# Masked alkynes for synthesis of threaded carbon chains

**DOI:** 10.1038/s41557-023-01374-z

**Published:** 2023-11-16

**Authors:** Connor W. Patrick, Yueze Gao, Prakhar Gupta, Amber L. Thompson, Anthony W. Parker, Harry L. Anderson

**Affiliations:** 1 Department of Chemistry, Chemistry Research Laboratory, University of Oxford, Oxford, UK; 2 Central Laser Facility, Research Complex at Harwell, Rutherford Appleton Laboratory, Didcot, UK

**Keywords:** Interlocked molecules, Synthetic chemistry methodology

## Abstract

Polyynes are chains of *sp*^1^ carbon atoms with alternating single and triple bonds. As they become longer, they evolve towards carbyne, the 1D allotrope of carbon, and they become increasingly unstable. It has been anticipated that long polyynes could be stabilized by supramolecular encapsulation, by threading them through macrocycles to form polyrotaxanes—but, until now, polyyne polyrotaxanes with many threaded macrocycles have been synthetically inaccessible. Here we show that masked alkynes, in which the C≡C triple bond is temporarily coordinated to cobalt, can be used to synthesize polyrotaxanes, up to the C_68_ [5]rotaxane with 34 contiguous triple bonds and four threaded macrocycles. This is the length regime at which the electronic properties of polyynes converge to those of carbyne. Cyclocarbons constitute a related family of molecular carbon allotropes, and cobalt-masked alkynes also provide a route to [3]catenanes and [5]catenanes built around cobalt complexes of cyclo[40]carbon and cyclo[80]carbon, respectively.

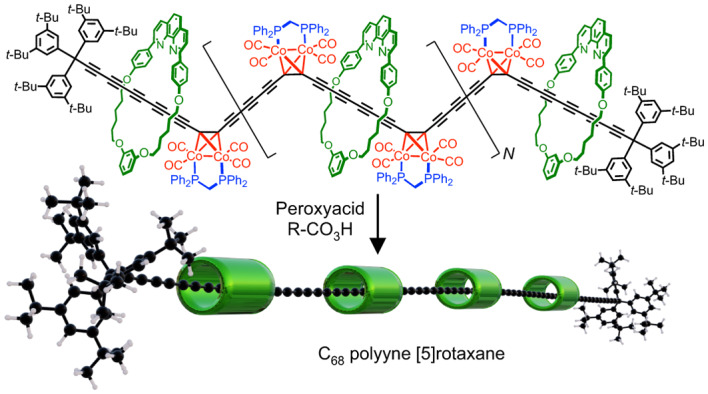

## Abstract (web summary)

Long polyynes have fascinating properties but they are difficult to synthesize as a consequence of their high reactivity. Now, it has been shown that cobalt carbonyl complexes can be used as masked alkyne equivalents, enabling the preparation of stable polyyne polyrotaxanes with up to 34 contiguous triple bonds.

## Main

**Fig. 1:**
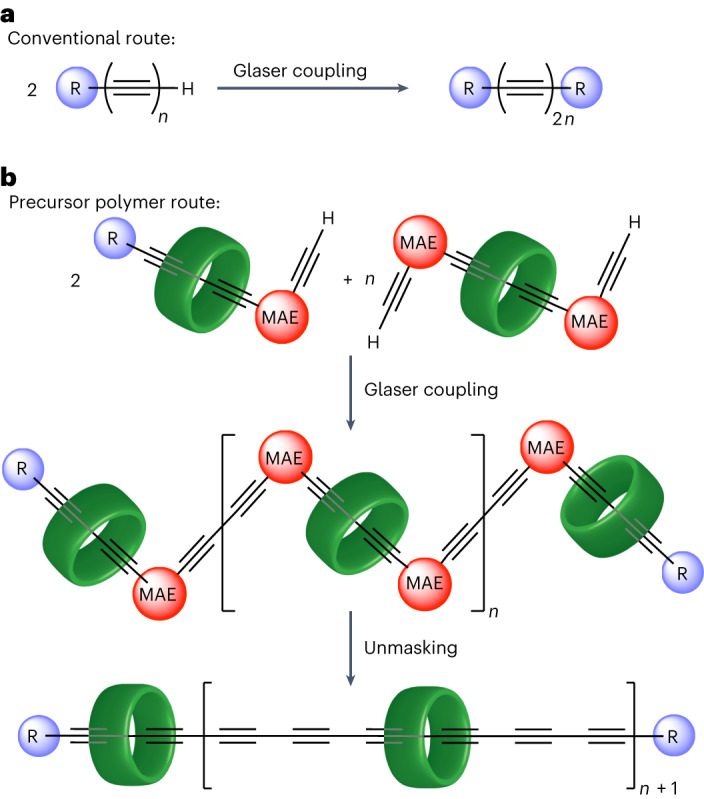
Strategies for synthesizing long polyynes. **a**, The classical approach to synthesize a polyyne preceded via a terminal polyyne with half as many carbon atoms in the chain^18–22^. **b**, In the precursor polymer route (this work), an MAE is used to conceal the reactivity of the alkynes until the whole backbone has been constructed.

The synthesis of new carbon allotropes is a rapidly evolving field^1–3^ that has already delivered technologically disruptive materials, such as fullerenes, carbon nanotubes and graphene. Recent advances include the synthesis of γ-graphyne^4,5^, covalent fullerene monolayers^6^, biphenylene networks^7^ and cyclo[18]carbon^8^. Allotropes composed entirely of *sp*^1^ two-coordinate carbon atoms, that is, carbyne and cyclo[*n*]carbons (the linear and cyclic forms, respectively), are under-explored as a consequence of their high reactivity. Linear chains of >50 carbon atoms have only been studied when encapsulated inside carbon nanotubes^9–11^, while structural studies of cyclocarbons have been limited to cryogenic temperatures^8,12,13^. Carbyne is predicted to be a 1D semiconductor^14^ with outstanding tensile strength^15,16^ and thermal conductivity^17^, and its properties have been deduced by extrapolation from experimental studies of monodisperse polyynes, which consist of chains of *sp*^1^-carbon atoms with bulky groups at both ends^18–22^. The longest previously reported polyynes of this type have chains of 48 *sp*^1^-carbon atoms^21^. These polyynes were synthesized using a strategy in which the final step is the oxidative Glaser of a fragile hydrogen-terminated polyyne intermediate, R-(C≡C)_*n*_-H, with half the length of the final product, R-(C≡C)_2*n*_-R (Fig. 1a). There are four major drawbacks to this conventional strategy for polyyne synthesis: (1) the instability of the terminal polyyne intermediate limits the efficiency of the final Glaser reactions, (2) the stepwise synthesis of this terminal polyyne intermediate can be laborious, (3) the final Glaser coupling step can be accompanied by loss of acetylenic units, resulting in shorter polyyne by-products that are difficult to separate from the desired product^18,21^, and (4) the stabilizing effect of the endgroups diminishes dramatically with increasing chain length.

Supramolecular encapsulation has previously been demonstrated to enhance the stability of polyyne dumbbells by threading them through macrocycles to form rotaxanes^23–25^. Polyyne [2]rotaxanes can be synthesized using active metal templates^24–31^, but there has been no method available for threading more than two macrocycles onto a polyyne chain. In this Article, we show that masked alkyne equivalents (MAEs)^25,30–32^ provided an efficient route to polyynes (Fig. 1b). This approach is analogous to the ‘precursor polymer’ routes used for synthesizing conjugated polymers^33,34^. Two types of masked building blocks are required to construct long polyyne polyrotaxanes: one with two reactive termini and one with a stopper at the end. We chose dicobalt tetracarbonyldiphenylphosphinomethane (Co_2_(CO)_4_dppm) as the masking group^32^, because it is bulky enough to act as a temporary stopper, to prevent unthreading of the macrocycles, and because it is unaffected by the reaction conditions of Glaser coupling. Here we show that these masked polyynes can be unmasked efficiently under mild conditions. This precursor polymer route circumvents all four of the drawbacks to the conventional route listed above, and it provides access to much longer polyynes than synthesized previously.

## Results and discussion

### Synthesis of [2]rotaxane intermediates

**Fig. 2:**
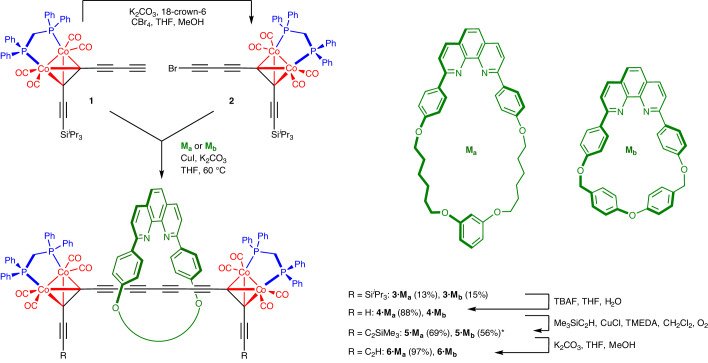
Synthesis of [2]rotaxane building blocks 6·M_a_ and 6·M_b_. Active metal template coupling of terminal butadiyne **1** and bromoalkyne **2** in the presence of macrocycle M_a_ or M_b_ was used to synthesize rotaxanes **3·M**_**a**_ and **3·M**_**b**_, which were then converted into **6·M**_**a**_ and **6·M**_**b**_. Asterisk indicates yield over two steps.

The cobalt alkyne complex **1** (Fig. 2) was prepared as reported previously^25^. Standard bromination conditions (for example, NBS/AgNO_3_) failed to convert **1** to the bromoalkyne **2**, probably because they oxidize the Co_2_(CO)_4_dppm group. Fortunately, this transformation can be achieved by treating **1** with carbon tetrabromide in the presence of potassium carbonate and 18-crown-6 in tetrahydrofuran/methanol. Bromoalkyne **2** is not stable as a solid at room temperature, but it can be handled as a solution. Two phenanthroline-based macrocycles **M**_**a**_ and **M**_**b**_ were chosen for this study^35,36^. The larger macrocycle **M**_**a**_ often gives higher yields of polyyne rotaxanes in active template coupling reactions^24–26,28–30^, whereas the smaller cavity of **M**_**b**_ is expected to protect the threaded polyyne more effectively. Active metal template Cadiot–Chodkiewicz coupling^37^ of a mixture of **1** and **2** in the presence of the macrocycles gave the symmetric [2]rotaxanes **3·M**_**a**_/**M**_**b**_ (Fig. 2). The [2]rotaxanes **3·M**_**a**_/**M**_**b**_ can also be isolated as by-products from the synthesis of rotaxanes **7·M**_**a**_/**M**_**b**_ by Cadiot–Chodkiewicz coupling of a mixture of **1** and supertrityl bromotriyne (for details, see Supplementary Section 2). Single crystal X-ray diffraction studies^38–43^ of rotaxanes **3·M**_**a**_ and **3·M**_**b**_ confirm that the macrocycle is threaded around the central octatetrayne thread (for more information, see details in Supplementary Section 4). De-protection of **3·M**_**a**_/**M**_**b**_ with tetrabutylammonium fluoride (TBAF) gives the terminal alkynes **4·M**_**a**_/**M**_**b**_ in high yield, but Glaser coupling of these alkynes is inefficient, probably as a result of steric hinderance, so both compounds were extended by coupling with excess trimethylsilyl acetylene to give **5·M**_**a**_/**M**_**b**_, and then treatment with potassium carbonate gave the bis-terminal alkynes **6·M**_**a**_/**M**_**b**_.

### Synthesis of polyrotaxanes

**Fig. 3:**
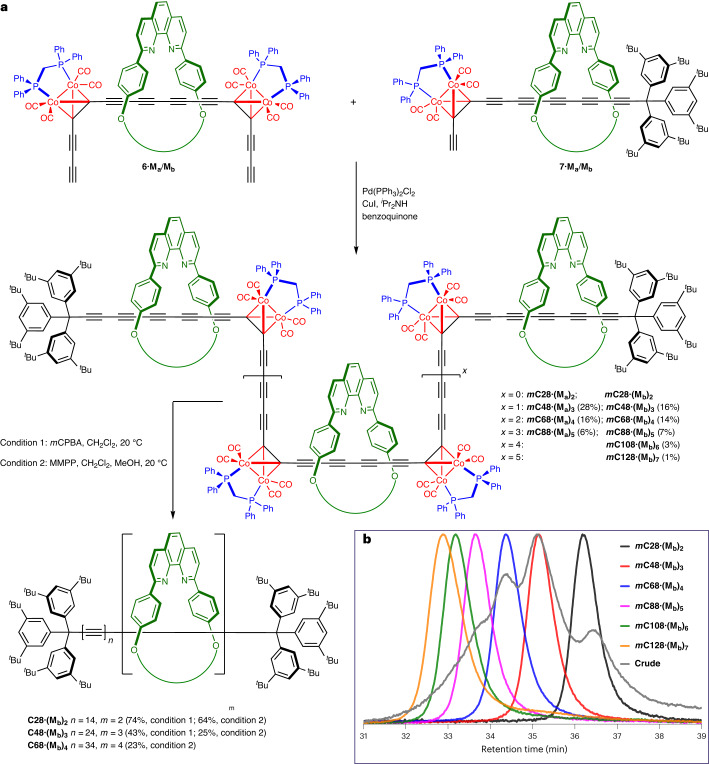
Synthesis of polyyne polyrotaxanes. **a**, Capped polymerization of **6·M**_**a**_ or **6·M**_**b**_, followed by unmasking, was used to prepare polyyne polyrotaxanes. **b**, Analytical GPC trace of masked polyrotaxanes precursors in the **M**_**b**_ series. TMEDA, tetramethylethylenediamine.

Palladium-catalysed oxidative alkyne coupling^44,45^ of the bis-functional [2]rotaxanes **6·M**_**a**_**/M**_**b**_, with mono-functional [2]rotaxanes **7·M**_**a**_**/M**_**b**_ as the capping agent, yielded a series of linear oligomers (Fig. 3), which can be separated by gel permeation chromatography (GPC). In the case of the larger macrocycle, coupling a 1:4 ratio of **6·M**_**a**_ and **7·M**_**a**_ gave [4]rotaxane ***m*C48·(M**_**a**_**)**_**3,**_ [5]rotaxane ***m*C68·(M**_**a**_**)**_**4**_ and [6]rotaxane ***m*C88·(M**_**a**_**)**_**5**_ in isolated yields of 28%, 16% and 6%, respectively, after the purification by recycling preparative GPC. For the other macrocycle, a 2:3 ratio of **6·M**_**b**_ and **7·M**_**b**_ gave ***m*C48·(M**_**b**_**)**_**3**_, ***m*C68·(M**_**b**_**)**_**4**_, ***m*C88·(M**_**b**_**)**_**5**_, ***m*C108·(M**_**b**_**)**_**6**_ and ***m*C128·(M**_**b**_**)**_**7**_ in 16%, 14%, 7%, 3% and 1% yields, respectively, under the same coupling conditions. These yields are based on **6·M**_**a**_/**M**_**b**_ as the limiting reagent. The [3]rotaxanes ***m*C28·(M**_**a**_**)**_**2**_ and ***m*C28·(M**_**b**_**)**_**2**_ were also formed, but we did not isolate them from these reactions because they are more easily prepared by homocoupling **7·M**_**a**_/**M**_**b**_ in the absence of **6·M**_**a**_/**M**_**b**_. The reference polyyne complexes ***m*C48** and ***m*C68** (that is, the dumbbells without any threaded macrocycles) were prepared in 51% and 18% yields, respectively, by coupling a 1:3 mixture of **6** and **7**.

### Unmasking of polyrotaxanes

The unmasking of Co_2_(CO)_4_dppm-polyynes complexes has previously been achieved using iodine, although this reaction is inefficient and unreliable; for example, unmaking ***m*C28·(M**_**a**_**)**_**2**_ with iodine gave the tetradecayne [3]rotaxane **C28·(M**_**a**_**)**_**2**_ in a poorly reproducible yield of 20–36% (refs. ^25,32,46^). After screening a range of oxidants, we found that *meta*-chloroperoxybenzoic acid (*m*CPBA) rapidly removes the Co_2_(CO)_4_dppm group. For example, treating [3]rotaxane ***m*C28·(M**_**b**_**)**_**2**_ with *m*CPBA (ten equivalents) in CH_2_Cl_2_ at 20 °C for 5 min gives the desired tetradecayne [3]rotaxane **C28·(M**_**b**_**)**_**2**_ in 74% yield. However, these reaction conditions are not compatible with the larger phenanthroline-based macrocycles **M**_**a**_, and ***m*C28·(M**_**a**_**)**_**2**_ reacts with *m*CPBA to give a complex mixture of products containing both the desired [3]rotaxane **C28·(M**_**a**_**)**_**2**_ and also the tetradecayne dumbbell **C28** (which appears to be formed via cleavage of the **M**_**a**_ macrocycle). Magnesium monoperoxyphthalate hexahydrate (MMPP) was tested as a milder oxidant and found to be very effective. Slow addition of MMPP (20 equivalents) in methanol to a solution of ***m*C28·(M**_**a**_**)**_**2**_ in CH_2_Cl_2_ over 1.5–2 h gave **C28·(M**_**a**_**)**_**2**_ in 59% isolated yield. These mild conditions are also effective with the **M**_**b**_ rotaxanes, giving **C28·(M**_**b**_**)**_**2**_ in 64% yield. The discovery of this unmasking method encouraged us to pursue longer polyyne rotaxanes. After subjecting the [4]rotaxane ***m*C48·(M**_**b**_**)**_**3**_ to *m*CPBA and MMPP conditions (separately), we isolated the tetracosayne [4]rotaxane **C48·(M**_**b**_**)**_**3**_ in 43% and 25% yields, respectively, after purification on silica gel. This polyyne [4]rotaxane **C48·(M**_**b**_**)**_**3**_ is an orange-red solid, and it is stable for weeks at room temperature without decomposition, both in solution and in the solid state, as confirmed by monitoring with thin layer chromatography or ultraviolet–visible (UV–vis) spectroscopy. The [4]rotaxane **C48·(M**_**a**_**)**_**3**_ and the naked polyyne dumbbell **C48** could be prepared from ***m*C48·(M**_**a**_**)**_**3**_ using MMPP (in 19% yield) and from ***m*C48** using *m*CPBA (in 56% yield).

**Fig. 4:**
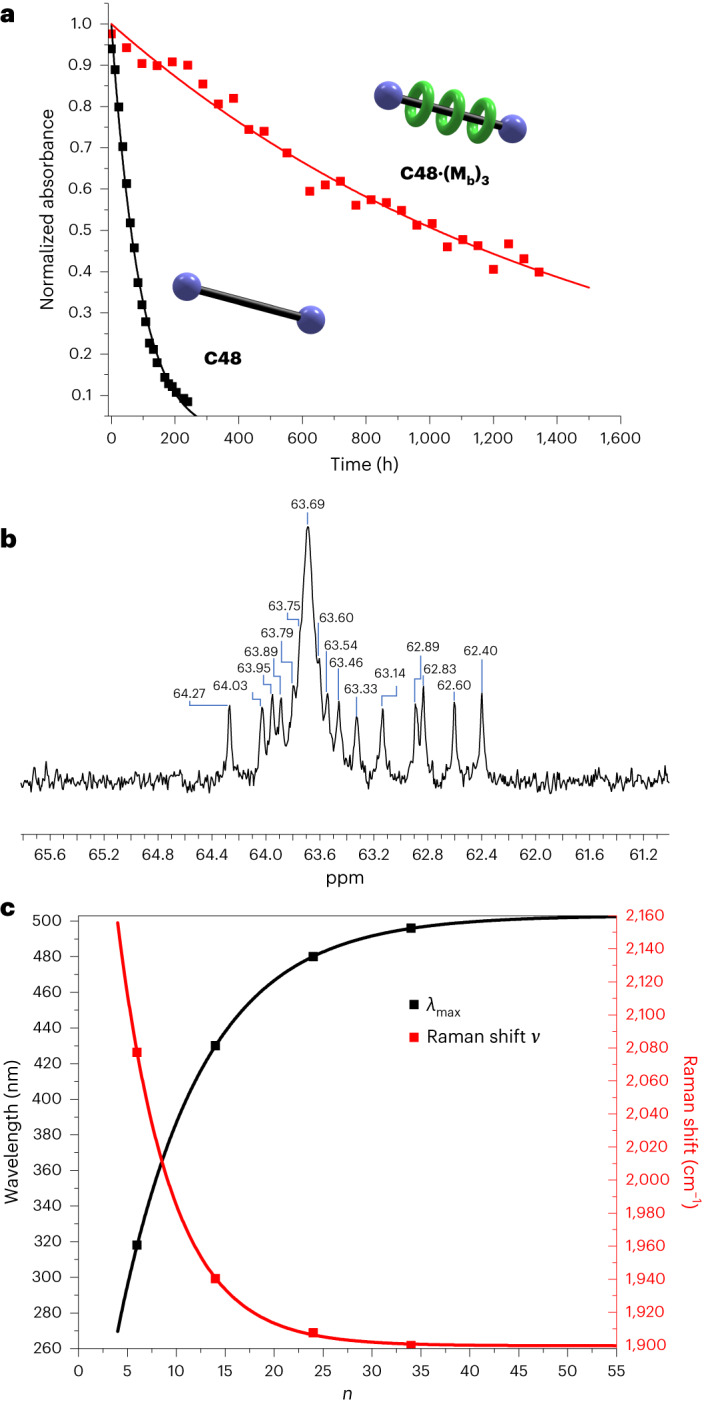
Characterization of polyyne rotaxane C48·(M_b_)_3_. **a**, Plot showing thermal decomposition of polyyne dumbbell **C48** and polyyne rotaxane **C48·(M**_**b**_**)**_**3**_ in the solid state at 30 °C. Decomposition in the solid state was monitored by dissolving samples of **C48** and **C48·(M**_**b**_**)**_**3**_ in CH_2_Cl_2_ and measuring the absorbance at 471 and 480 nm, respectively. Data were fitted to a first-order decay model, *A*(*t*) = exp(–*kt*), with *k* = 0.011 h^–1^ and 0.00068 h^–1^ for **C48** and **C48·(M**_**b**_**)**_**3**_, respectively. **b**, A partial ^13^C NMR spectrum of polyyne [4]rotaxane **C48·(M**_**b**_**)**_**3**_ showing selected *sp*-carbon chemical shifts (151 MHz, CD_2_Cl_2_, 298 K). **c**, A plot of absorption wavelength of the absorption maximum *λ*_max_ and Raman shift *ν* as a function of molecular length in the **M**_**b**_ series fitted to equation (1) gives an *λ*_∞_ = 503 nm and *v*_∞_ = 1,900 cm^–1^. The data are listed in Table 1. *n* is the number of C≡C triple bonds in the polyyne. Source data

The thermal stability of **C48·(M**_**b**_**)**_**3**_ in the solid state, in the dark under air at 30 °C, was compared with that of the corresponding polyyne dumbbell **C48** (for more information, see details in Fig. 4a and Supplementary Section 8). Both compounds decompose gradually with first-order kinetics, with a half-life of 1,022 h for **C48·(M**_**b**_**)**_**3**_ compared with 62 h for **C48**, which illustrates the substantial increase in stability conferred by supramolecular encapsulation. It is surprising that **C48** decomposes so slowly in the solid state, even without any threaded macrocycles.

The synthesis of the polyyne dumbbell **C48** via ***m*C48** illustrates the utility of MAEs in polyyne synthesis. The polyyne [4]rotaxanes **C48·(M**_**a**_**)**_**3**_ and **C48·(M**_**b**_**)**_**3**_ and dumbbell **C48** were fully characterized by ^1^H nuclear magnetic resonance (NMR), UV–vis and Raman spectroscopy and high-resolution mass spectrometry. We also recorded the ^13^C NMR spectrum of [4]rotaxane **C48·(M**_**b**_**)**_**3**_, and 19 out of the 24 *sp*-carbon resonances were resolved. The longest polyyne previously characterized by ^13^C NMR has a chain of 44 *sp*-carbons and shows a convergence of the *sp*-carbon resonance at 63.7 ppm^20^. In the ^13^C NMR spectrum of [4]rotaxane **C48·(M**_**b**_**)**_**3**_, with 48 *sp*-carbons, an envelope of overlapping signals is observed in the region 63.6–63.8 ppm (Fig. 4b), consistent with previous work.

Unmasking of the [5]rotaxane ***m*C68·(M**_**b**_**)**_**4**_ using MMPP (120 equivalents) gave the polyyne [5]rotaxane **C68·(M**_**b**_**)**_**4**_ in a 23% yield, after purification by silica gel chromatography and washing with ethanol, whereas this compound could not be prepared by unmasking ***m*C68·(M**_**b**_**)**_**4**_ with *m*CPBA. This polyyne [5]rotaxane is an orange-red solid, and it is stable in solution over several days on storing at −20 °C, but some decomposition is observed during chromatography. The C68 polyyne chain of this [5]rotaxane makes it longer than any previously reported polyyne^21^. It was fully characterized by ^1^H NMR, UV–vis and Raman spectroscopy and high-resolution mass spectrometry. We also prepared **C68·(M**_**a**_**)**_**4**_ but this [5]rotaxane is less stable than the version with the smaller macrocycle, which prevented characterization by NMR spectroscopy.

### UV–vis and Raman spectra

Polyynes exhibit characteristic electronic absorption bands, with sharp vibronic fine structure, which shift to longer wavelength as the length of the polyyne chain increases^19–22^. The absorption maxima (*λ*_max_) of the polyyne dumbbells and polyyne rotaxanes synthesized in this study are summarized in Table 1. The presence of threaded macrocycles has little effect on the absorption spectra, and the *λ*_max_ values of the naked polyyne dumbbells are similar to those of the corresponding polyyne rotaxanes, although the spectra of the rotaxanes are red-shifted by 3–9 nm, due to the different solvent environment around the threaded polyyne chain^24^, while the type of macrocycle (**M**_**a**_ versus **M**_**b**_) has no noticeable effect on the spectra. The variation in *λ*_max_ with the number of triple bonds, *n*, is plotted in Fig. 4c for the series **C12·M**_**b**_, **C28·(M**_**b**_**)**_**2**_, **C48·(M**_**b**_**)**_**3**_ and **C68·(M**_**b**_**)**_**4**_. These data fit well to the Meier equation^47^, equation (1), as reported by Chalifoux and Tykwinski for a series of shorter polyynes^20^,

1 $${\lambda }_{(n)}={\lambda }_{\infty }-({\lambda }_{\infty }-{\lambda }_{1}){{\rm{e}}}^{-k(n-1)}$$

where *λ*_∞_, *λ*_1_ and *k* are empirical parameters that reflect the *λ*_max_ values at *n* = ∞ and 1, respectively, and the rate of saturation. The data for our polyrotaxanes give *λ*_∞_ = 503 nm, *λ*_1_ = 172 nm and *k* = 0.116. These values are similar to those deduced by Chalifoux and Tykwinski for their supertrityl polyynes (*λ*_∞_ = 486 nm, *λ*_1_ = 175 nm and *k* = 0.116)^20^.

**Table 1:** Absorption maxima wavelengths (*λ*_max_) and peak Raman frequencies (*ν*)^a^

	C12	C12·M_b_	C28	C28·(M_a_)_2_	C28·(M_b_)_2_	C48	C48·(M_a_)_3_	C48·(M_b_)_3_	C68·(M_a_)_4_	C68·(M_b_)_4_
*λ*_max_ (nm)	315	318	423	430	430	471	479	480	492	496
*ν* (cm^−1^)	2,077	2,077	1,944	1,940	1,940	1,913	1,909	1,907	1,900	1,900

^a^All data were measured in CH_2_Cl_2_ at 25 °C, at concentrations of ~1 × 10^–5^ M for UV–vis and 1 × 10^–3^ M for Raman. Excitation wavelength of 1,064 nm, continuous-wave laser; spectral resolution of 4 cm^−1^.

The Raman spectra of polyynes are dominated by an intense band at around 1,900–2,100 cm^–1^, arising from in-phase stretching of the C≡C triple bonds, and the frequency of this vibration (*ν*) is sensitive to the degree of bond-length alternation in the polyyne chain^16,21,48^. The Raman frequencies of the polyyne dumbbells and rotaxanes are listed in Table 1, and plotted against the number of triple bonds, *n*, for the **M**_**b**_ rotaxanes in Fig. 4c. The presence of a threaded macrocycle has a negligible effect on the Raman frequency. This contrasts with the situation when a polyyne is encapsulated inside a double-walled carbon nanotube, where the Raman frequency is governed by the chirality of the inner tube and reduces with decreasing inner tube diameter^9–11^. The reduction in frequency of 177 cm^–1^ from **C12·M**_**b**_ (*ν* = 2,077 cm^–1^) to **C68·(M**_**b**_**)**_**4**_ (*ν* = 1,900 cm^–1^) reflects a reduction in bond-length alternation with elongation of the polyyne chain^48^. Fitting the Meier equation to the Raman frequencies plotted in Fig. 4c (that is, using equation (1) with *ν* instead of *λ*) gives *ν*_∞_ = 1,900 cm^–1^, *ν*_1_ = 2,343 cm^–1^ and *k* = 0.183. This value of the predicted Raman frequency of infinite carbyne (1,900 cm^–1^) is slightly higher than that deduced by Gao et al. from the Meier fit of a series of shorter pyridine-terminated polyynes (*ν*_∞_ = 1,886 cm^–1^) (ref. ^21^). The frequency for the [5]rotaxane **C68·(M**_**b**_**)**_**4**_ happens to equal the asymptotic limit, which illustrates that the Raman frequencies have already converged to that of carbyne. Carbyne chains inside double-walled carbon nanotube have lower Raman frequencies (1,770–1,850 cm^–1^; refs. ^9–11^), and in this case, the presence of the carbon nanotube shifts the resonance to lower frequency by about 100 cm^–1^ (ref. ^11^).

### Synthesis of catenanes

**Fig. 5:**
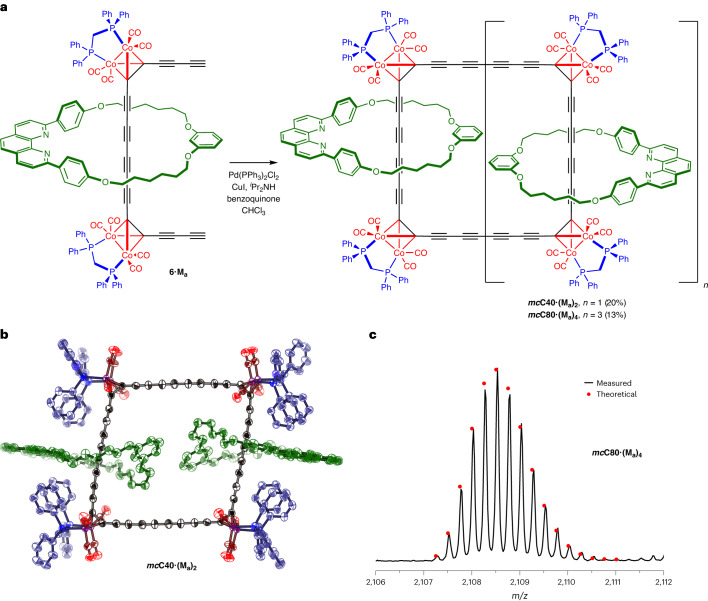
Synthesis of [3]catenane and [5]catenane. **a**, Reaction scheme for catenane synthesis. **b**, Solid-state structure of the [3]catenane ***mc*C40·(M**_**a**_**)**_**2**_. Thermal ellipsoids plotted at the 40% level. The macrocycles are coloured green, the phosphene ligands are blue and the oxygen atoms of the carbonyl ligands are red. **c**, nESI mass spectrum of the [5]rotaxane ***mc*C80·(M**_**a**_**)**_**4**_ compared with the calculated isotopomer distribution for [M + 4H]^4+^. Source data

Palladium-catalysed oxidative homocoupling of the bis-functional [2]rotaxane **6·M**_**a**_ yields a series of cyclic oligomers (Fig. 5), which can be separated by GPC. We isolated the masked cyclo[40]carbon [3]catenane ***mc*C40·(M**_**a**_**)**_**2**_ (20% yield) and the masked cyclo[80]carbon [5]catenane ***mc*C80·(M**_**a**_**)**_**4**_ (13% yield). These catenanes were fully characterized by ^1^H, ^13^C and ^31^P NMR spectroscopy, and analytical GPC, but none of these techniques provides definitive information on the number of repeat units. At first, attempts at recording matrix-assisted laser desorption/ionization time-of-flight mass spectra of these two cyclic oligomers were unsuccessful and we tentatively assumed that they were the [3] and [4]catenane. Fortunately, nanoelectrospray ionization (nESI) mass spectrometry gave intense well-resolved molecular ions for both compounds, proving that the larger structure is the [5]catenane, as shown by the excellent fit between the calculated and observed isotopomer pattern (Fig. 5c). Surprisingly, we were unable to detect any formation of the [4]catenane. Single crystals of the [3]catenane were grown by diffusion of cyclohexane vapour into a solution of ***mc*C40·(M**_**a**_**)**_**2**_ in tetrahydrofuran. Single crystal X-ray diffraction studies confirmed the interlocked structure (Fig. 5b). In the solid state, the catenane has *C*_i_ symmetry, with half a molecule in the asymmetric unit. Both the diphenylphosphinomethane (dppm) units point towards the **M**_**a**_ macrocycles, in contrast to the conformations adopted in the crystal structures of **3·M**_**a**_ (one dppm in and one out) and **3·M**_**b**_ (both dppm units out, that is, away from the **M**_**b**_ macrocycle; Supplementary Section 4). These dppm complexes are conformationally dynamic in solution (in fast exchange by NMR at room temperature)^49^, and the arrangement in the solid state is probably influenced by crystal packing. Unmasking of these two catenanes was attempted using *m*CPBA in CH_2_Cl_2_ at room temperature; however, this reaction gave a complex mixture of products. We have not yet attempted to synthesize catenanes derived from **3·M**_**b**_, and it seems likely that a more bulky version of **M**_**b**_ will be required to achieve the synthesis of a stable unmasked cyclocarbon catenane.

## Conclusions

The results presented here reveal that Co_2_(CO)_4_dppm is an excellent masking group for the synthesis of long polyynes, and particularly polyyne polyrotaxanes, because it is stable to Glaser-type oxidative coupling conditions and it can be removed efficiently by peracid oxidants, such as *m*CPBA and MMPP. When synthesizing conjugated polymers, it is often desirable to use a ‘precursor polymer route’. In this strategy, a non-conjugated precursor polymer is synthesized and then converted into the target conjugated polymer through an elimination step, so that the extended π-system is only revealed after construction of the covalent backbone^33,34^. The precursor route presented here (Fig. 1b) is advantageous, even for the synthesis of polyynes with no threaded macrocycles, as illustrated by the synthesis of **C48** from ***m*C48**, because it avoids the need for a long terminal polyyne intermediates and it allows the polymer backbone to be created before unmasking the whole polyyne chain. The use of Co_2_(CO)_4_dppm masking groups is particularly attractive for the synthesis of polyyne polyrotaxanes, because these organometallic complexes act as temporary stoppers, to prevent the macrocycles from unthreading. We have demonstrated this chemistry by preparing long masked polyynes up to the [8]rotaxane ***m*C128·(M**_**b**_**)**_**7**_, which is a precursor to a C_128_ polyyne, but we have not yet prepared this compound on a sufficient scale to test its unmasking. The longest unmasked polyyne polyrotaxane that we have synthesized during this study is the [5]rotaxane **C68·(M**_**b**_**)**_**4**_ with 34 contiguous alkyne units. The UV–vis absorption maxima and Raman frequencies for the homologous series of oligomers **C12·M**_**b**_, **C28·(M**_**b**_**)**_**2**_, **C48·(M**_**b**_**)**_**3**_ and **C68·(M**_**b**_**)**_**4**_ fit well to the Meier equation, indicating that an infinite carbyne chain would have an absorption maximum of *λ*_∞_ = 503 nm, and a Raman frequency of *ν*_∞_ = 1,900 cm^–1^. These asymptotic values match closely with those for **C68·(M**_**b**_**)**_**4**_ (*λ*_max_ = 496 nm; *ν* = 1,900 cm^–1^) showing that the spectroscopic behaviour has essentially saturated at this chain length. We have not yet fully explored the application of this chemistry to the synthesis of cyclocarbon catenanes, but we have demonstrated the synthesis of the masked [3]catenane ***mc*C40·(M**_**a**_**)**_**2**_ and [5]catenane ***mc*C80·(M**_**a**_**)**_**4**_.

## Online content

Any methods, additional references, Nature Portfolio reporting summaries, source data, extended data, supplementary information, acknowledgements and peer review information; details of author contributions and competing interests; and statements of data and code availability are available at 10.1038/s41557-023-01374-z.

### Supplementary information

Supplementary Information Supplementary Figs. 1–104, Tables 1–4, experimental procedures and discussion.

Supplementary Data 1 Crystallographic information file for **3·Ma**; CCDC reference 2224109.

Supplementary Data 2 Crystallographic structure factors for **3·Ma**; CCDC reference 2224109.

Supplementary Data 3 Crystallographic information file for **3·Mb**; CCDC reference 2224110.

Supplementary Data 4 Crystallographic structure factors for **3·Mb**; CCDC reference 2224110.

Supplementary Data 5 Crystallographic information file for ***mc*C40·(Ma)**_**2**_; CCDC reference 2224111.

Supplementary Data 6 Crystallographic structure factors for ***mc*C40·(Ma)**_**2**_; CCDC reference 2224111.

### Source data

Source Data Fig. 4a Plots of normalized absorption versus time and first-order fits.

Source Data Fig. 4b Partial 13C NMR spectrum.

Source Data Fig. 4c Absorption wavelength and Raman frequency versus *n* and fits.

Source Data Fig. 5c Mass spectrum.

## Data Availability

Relevant data are available within the paper and its Supplementary Information files. The NMR and mass spectra are presented in detail in the main Supplementary Information file, and the raw data for these spectra are available on reasonable request from the authors. Crystallographic data for the structures reported in this Article have been deposited at the Cambridge Crystallographic Data Centre, under deposition numbers CCDC 2224109 (**3·M**_**a**_), 2224110 (**3·M**_**b**_) and 2224111 (***mc*****C40·(M**_**a**_**)**_**2**_). Copies of the data can be obtained free of charge via https://www.ccdc.cam.ac.uk/structures/. Source data are provided with this paper.
