# Supplementary material for: Masked alkynes for synthesis of threaded carbon chains
Source: Nat Chem. 2023 Nov 16;16(2):193–200. doi: 10.1038/s41557-023-01374-z (PMC10849957; doi:10.1038/s41557-023-01374-z)
Supplement: Supplementary file 1 — Supplementary Figs. 1–104, Tables 1–4, experimental procedures and discussion. [file 41557_2023_1374_MOESM1_ESM.pdf]

nature chemistry

Article https://doi.org/10.1038/s41557-023-01374-z

Masked alkynes for synthesis of threaded carbon chains

In the format provided by the authors and unedited

Table of Contents

Section 1: General Methods **2–4**

Section 2: Synthetic Protocols **5–28**

Section 3: GPC Traces **29–30**

Section 4: X-Ray Crystal Structures **31–34**

Section 5: UV-Visible Absorption Spectra **35–39**

Section 6: Raman Spectra **40–43**

Section 7: Comparison of NMR Spectra **44**

Section 8: Solid-State Thermal Stability **45–46**

Section 9: NMR Spectra **47–82**

Section 10: Mass Spectra **83–93**

Section 11: References **94**

Section 1: General Methods

Commercially available reagents were used as received. Dry solvents (THF, CH_2Cl_2, $CHCl_3$ and MeCN) for reactions were prepared using a MBraun MB-SPS-5 bench-top solvent purification system (SPS) under nitrogen (H_2O content < 20 ppm). All other solvents were HPLC grade and were used without further purification. In the synthetic procedures, the solvents described as 'dry solvent' were purified using the SPS; if 'dry' is not stated, this means HPLC grade. Petroleum ether had a boiling point range of 40–60 °C. *N,N,N′,N′*-Tetramethylethylenediamine (TMEDA, 99%), diisopropylamine (99.5%), 3-chloroperbenzoic acid (*m*CPBA, ≤ 77%) and magnesium monoperoxyphthalate hexahydrate (MMPP, 80%) were purchased from Sigma-Aldrich and used as received. EDTA/NH_3 solution was prepared by saturating a 1:1 solution of water/aqueous 35% NH_3 solution with tetrasodium EDTA. Copper(I) chloride was prepared according to the published procedure[1] because CuCl prepared in this way has a finer particle size than commercial material, which is beneficial for Glaser coupling reactions.

Thin layer chromatography (TLC) was carried out on aluminum-backed silica gel plates with 0.2 mm thick silica gel 60 F254 (Merck) and visualized by UV irradiation at either 254 nm or 366 nm. Preparative flash column chromatography was carried out using flash silica gel 60 (230-400 mesh) obtained from Sigma-Aldrich, or on a Biotage Isolera One with a 200–400 nm UV detector.

Size exclusion chromatography (SEC) was carried out using Bio-Beads S-X1 or S-X3, 40–80 µm (Bio Rad). Analytical GPC was carried out on a VWR system equipped with a JAIGEL H-P pre-column, a JAIGEL-3H-A (8 × 500 mm) and a JAIGEL-4H-A (8 × 500 mm) column in series with THF + 1% pyridine as eluent with a flow rate of 1.0 mL/min. Semipreparative GPC was carried out on a Shimadzu recycling GPC system equipped with a LC-20 AD pump, SPD-20A UV detector and a JAIGEL H-P pre-column, a JAIGEL 3H (20 × 600 mm) and a JAIGEL 4H (20 × 600 mm) column in series with THF as the eluent at a flow rate of 3.5 mL/min.

^{1}H, ^{13}C, and ^{31}P NMR spectra were recorded on Bruker NEO 400, Bruker AVIII HD 500, Bruker AVIII HD 600 (Prodigy broadband cryoprobe), Avance NEO 600 (broadband helium cryoprobe), and AVIII 700 (^{1}H/^{13}C/^{15}N TCI cryoprobe) spectrometers at 400 MHz, 500 MHz, 600 MHz, and 700 MHz (^{1}H),100 MHz, 126 MHz, 151 MHz, and 175 MHz (^{13}C), and 162 MHz, 202 MHz, and 243 MHz (^{31}P), respectively at 298 K. All chemical shifts are reported in ppm, coupling constants are reported in Hz and ^{1}H multiplicities are reported in accordance with the following: s = singlet; br s = broad singlet; d = doublet; t = triplet; q = quartet; and m = multiplet. ^{1}H and ^{13}C NMR chemical shifts are reported relative to $SiMe_4$ ($\delta = 0$). ^{31}P NMR spectra were externally referenced to an 85 % aqueous solution of H_3PO_4 ($\delta_P = 0.0$ ppm) using the spectrometer reference values. ^{1}H NMR spectra were referenced to the residual solvent signal ($CDCl_3$: 7.26 ppm, CD_2Cl_2: 5.32 ppm) and ^{1}H NMR assignments were made using the information provided by coupling constants and/or 2D NMR methods (COSY, NOESY, HSQC, HMBC). ^{13}C NMR spectra were referenced to the solvent signal (CD_2Cl_2: 53.84 ppm, $CDCl_3$: 77.16 ppm). ^{13}C NMR spectra of cobalt carbonyl complex of alkynes often showed fewer than the expected number of peaks due to the broadness and low intensity (from the slow relaxation) of the carbons bound to cobalt and the overlap between the signals from the many carbon environments.

High-resolution mass spectrometry measurements were carried out by either: (i) electrospray ionization (ESI) mass spectrometry from 10 nM solutions of sample in 1:1 CH_2Cl_2:MeOH on a Thermo Scientific Q Exactive Hybrid Quadrupole-Orbitrap mass spectrometer using 90:10 MeOH:H_2O (+0.1% formic acid) as the

mobile phase by the mass spectrometry service at the University of Oxford; or (ii) nanoelectrospray ionization (nESI) mass spectrometry from 10 nM solutions of sample in 4:4:1 CH_2Cl_2:MeOH:formic acid on a Waters Synapt G2-S spectrometer equipped with an Advion Biosciences Nanomate Triversa chip-based nanospray system in positive mode using MassLynx software (v4.1) by Mark F. Wyatt (Wyatt Analytical Ltd) and Paul J. Gates at the University of Bristol. The Nanomate was set to aspirate 5 μL of sample solution. MALDI-ToF spectra were measured on a Bruker MALDI Autoflex Speed mass spectrometer using *trans*-2-[3-(4-*tert*-butylphenyl)-2-methyl-2-propenylidene]malononitrile (DCTB) as the matrix.

UV-vis spectra were recorded in solution on a Perkin-Elmer Lambda 20 or Perkin-Elmer Lambda 25 spectrometer at 298 K, in fused silica cuvettes with a pathlength of 1 cm. Molar absorption coefficients (ε, with units M^{-1} cm^{-1}) were determined from absorption spectra of solutions measured at appropriate concentrations to give an absorption in the range 0.5–1.5 with a path length of 1 cm.

Infrared spectra were recorded on a Bruker Tensor 27 FT-IR spectrometer with internal calibration range of 4000-600 cm^{-1} and the sample being prepared as a thin film on a diamond ATR module.

Raman spectra were recorded in CH_2Cl_2 solutions (typically 1 mM concentration) using a Bruker MultiRAM FT-Raman spectrometer. A continuous-wave Nd:YAG laser working at 1064 nm was employed for excitation. Raman scattering radiation was collected in a back-scattering configuration with a spectral resolution of 4 cm^{-1}, and samples were held in a stationary fused silica NMR tube. Laser power was typically 50 mW and kept low to avoid sample degradation, spectra accumulation times were typically 64 accumulations.

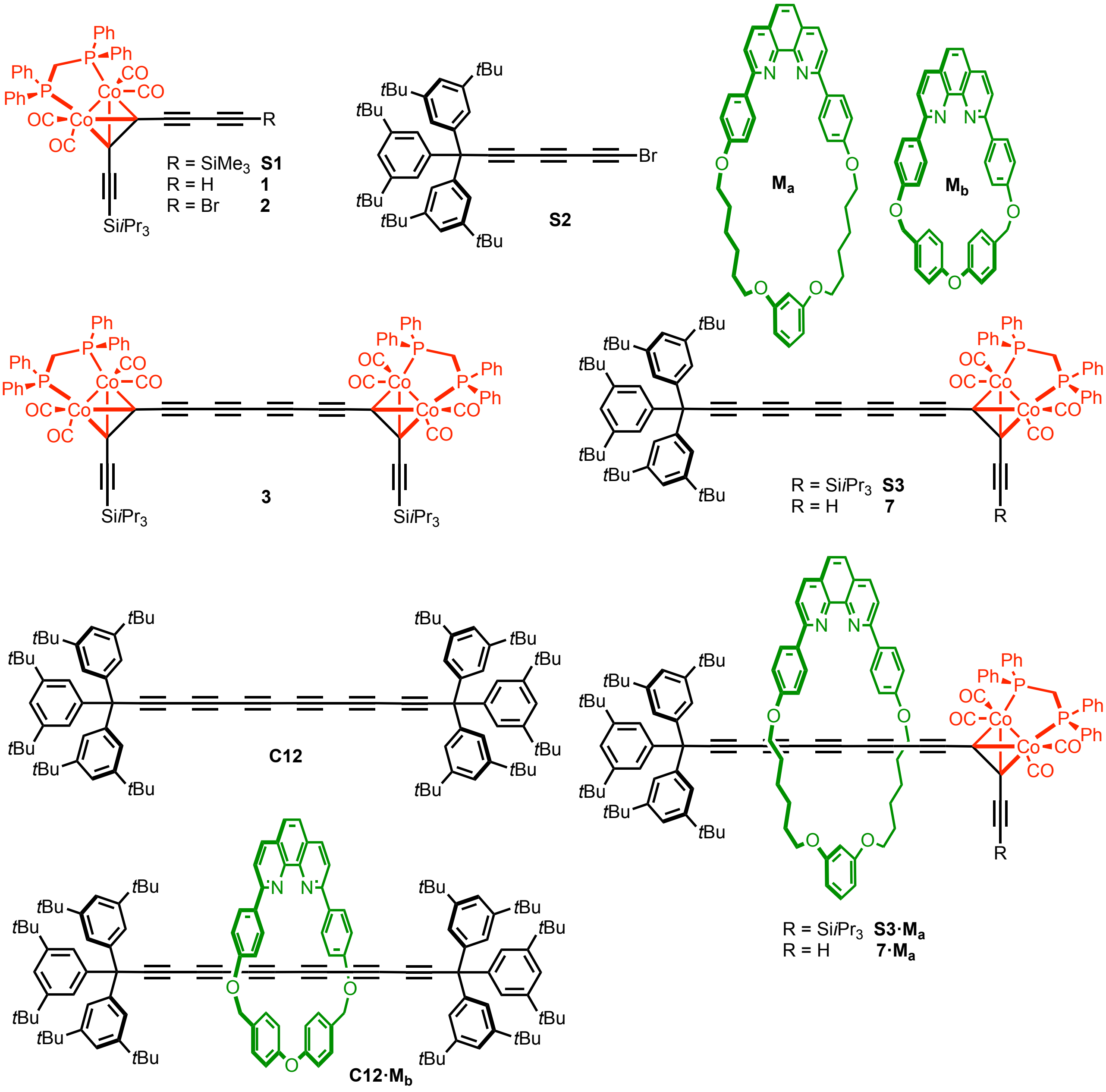

Figure 1. Previously reported molecular structures (except for compound **2**) used in this work.

The supertrityl (Tr*) triyne bromide compound **S2**,[2] dicobalt masked tetrayne **S1** and **1**, phenanthroline macrocycles $\mathbf{M_a}$[3] and $\mathbf{M_b}$,[4] tetracobalt masked octayne **3**,[5] compounds **S3**, $\mathbf{S3{\cdot}M_a}$, **7**, and $\mathbf{7{\cdot}M_a}$,[6] and polyyne rotaxane $\mathbf{C12{\cdot}M_b}$[7] and polyyne dumbbell **C12**[2] were synthesized as reported previously.

Unless otherwise noted in the individual procedures, complexes $\mathbf{M_a}$·CuI[6] and $\mathbf{M_b}$·CuI[8] were prepared as described in the literature. A typical procedure was described as follows: To a solution of macrocycle $\mathbf{M_b}$ (400 mg, 0.716 mmol) in CH_2Cl_2 (20 mL) was added a solution of copper(I) iodide (136 mg, 0.716 mmol) in MeCN (20 mL), and the solution was stirred for 1 h at 20 °C. Solvent removal gave $\mathbf{M_b}$·CuI as a dark red solid. The complexes were used in the next reactions without further purification.

Section 2: Synthetic Protocols

CAUTION: Some polyynes have been reported to be explosive. We have not experienced problems of this type, but all polyynes should be handled with care and not prepared on a large scale.

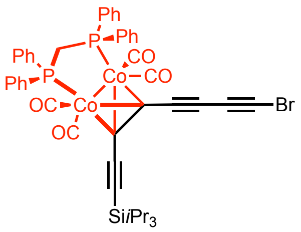

Compound 2: Potassium carbonate (0.18 g, 1.3 mmol) and 18-crown-6 (0.20 g, 0.77 mmol) were added to a solution of compound **S1** (0.24 g, 0.26 mmol) in THF (2 mL) and MeOH (2 mL), and the solution was stirred for 5 min at 20 °C. To the resulting solution was added a solution of CBr_4 (169 mg, 0.51 mmol) in THF (2 mL) and MeOH (2 mL). The mixture was stirred for 10 min at 20 °C and the reaction was monitored using TLC. Petroleum ether (10 mL) was added to the mixture and the solution passed through a short silica column (petroleum ether then petroleum ether/CH_2Cl_2 9:1) to give compound **2** as a red solution. *As this compound decomposes rapidly upon drying, the yield of the reaction was determined by ^1H NMR spectroscopy: Compound **S1** (34 mg, 0.036 mmol) was treated with the procedure described above, and the yield of compound **2** (24.6 mg, 26.0 µmol, 72 % yield) was determined by NMR using an internal reference of ethyl acetate in $CDCl_3$. The NMR solution was dried completely and resulted in a partially/completely decomposed solid (32 mg), assuming this mass was the intact compound **2**, the yield would be 94%. The difference between the two yields may be due to the decomposition when preparing the NMR sample as an insoluble solid was observed on the wall of the NMR tube.*

^{1}H NMR (600 MHz, $CDCl_3$) δ_H 7.42 (q, J = 6.2 Hz, 4H), 7.37 – 7.21 (m, 12H), 7.16 (d, J = 7.7 Hz, 4H), 3.51–3.32 (m, 2H, $PC\underline{H}_2$), 1.17–1.06 (m, 19H, iPr$_3$).**^{13}C{^{1}H} NMR** (151 MHz, $CDCl_3$) δ_C 203.4 (br, C=O), 201.5 (br, C=O), 136.6 (br), 134.0 (br), 132.4 (t, $J_{C–P}$ = 6.6 Hz), 131.5 (t, $J_{C–P}$ = 6.2 Hz), 130.1, 129.9, 128.6 (t, $J_{C–P}$ = 5.0 Hz), 128.4 (t, $J_{C–P}$ = 5.0 Hz), 108.7, 100.4, 81.0, 78.3, 67.9, 45.2 (C-Br), 35.9 (t, $J_{C–P}$ = 22.5 Hz, $P\underline{C}H_2$), 18.9 ($SiCH(\underline{C}H_3)_2$), 11.6 ($Si\underline{C}H(CH_3)_2$). **^{31}P{^{1}H} NMR** (243 MHz, $CDCl_3$) δ_P 38.66. **ESI HRMS *m/z:*** calcd for $C_{46}H_{44}{}^{79}BrCo_2O_4P_2Si$ ($[M + H]^+$) 947.0326, found 947.0320.

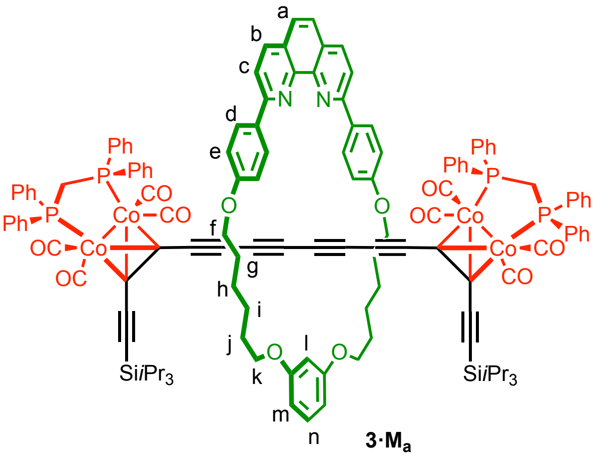

3·M$_a$

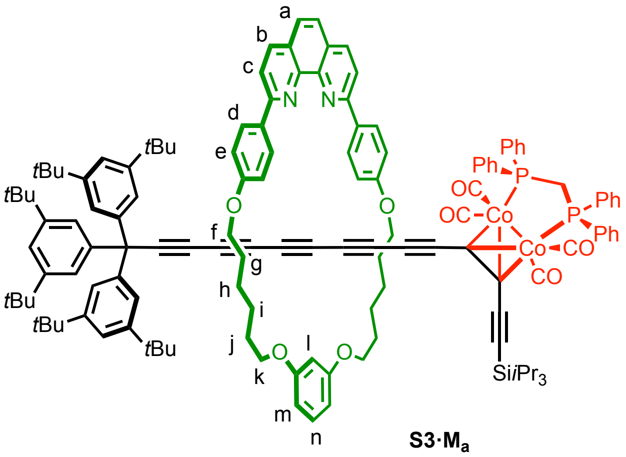

S3·M$_a$

Compound 3·M$_a$: **Method 1.** Saito macrocycle **M$_a$** (212 mg, 1.05 eq., 332 µmol) and copper(I) iodide (60.3 mg, 1.00 eq., 317 µmol) were combined and dissolved in a solution of dryMeCN (10.5 mL) and dry CH_2Cl_2 (15.0 mL) and stirred for 1 h under argon at 20 °C. The solvent was removed under reduced pressure and the residual solid dissolved in dry THF (25 mL). Deprotected cobalt-masked tetrayne **1** (275 mg, 1.00 eq., 317 µmol), supertrityl triyne bromide **S2** (232 mg, 1.00 eq., 317 µmol) and potassium carbonate (219 mg, 5.00 eq., 1.58 mmol) were combined and evacuated of air and flushed with argon three times. The solids were cooled in liquid nitrogen, before adding the Cu-macrocycle complex **M$_a$**·CuI, along with additional dry THF

(25 mL). The reaction mixture was thoroughly degassed (3 freeze-pump-thaw cycles) before warming to 60 °C and stirring under argon for 18 h. EDTA/NH_3 solution (40 mL) was added to the cooled reaction mixture and left to stir for 1 h. Et_2O (50 mL) was added and the organic layer extracted. The aqueous layer was washed with Et_2O (2 × 20 mL) and the combined organic extracts dried over Na_2SO_4 before removing the solvent under reduced pressure. The crude product was subject to SiO_2 chromatography (petroleum ether/EtOAc, gradient elution from 0 to 33%), then size-exclusion chromatography (Bio-Rad S-X3, toluene) to yield Tr* [2]rotaxane **S3·M$_a$** (240 mg, 110 μmol, 35%)[6] and tetracobalt [2]rotaxane **3·M$_a$** (101 mg, 42.5 μmol, 27%) as dark brown solids.

Method 2. To a solution of compound **S1** (50 mg, 0.053 mmol) in THF/MeOH (5 mL/5 mL) was added K_2CO_3 (73.4 mg, 0.531 mmol) at 20 °C, and the solution was stirred for 10 min. This solution was passed through a silica plug (CH_2Cl_2/petroleum ether 1:1), which resulted in a solution of compound **1**. Without further purification, the resulting solution was concentrated to ca. 2 mL, and dry THF (ca. 10 mL) was added; then this resulting solution was again concentrated, and dry THF was added; repeating this three times gave a solution of compound **1** in THF (3 mL). Separately, compound **2** (0.064 mmol, assuming 100% yield) in dry THF (2 mL) was prepared from compound **S1** (50 mg, 0.053 mmol) as described above. Complex **M$_a$**·CuI (48.5 mg, 0.059 mmol) and K_2CO_3 (36.7 mg, 0.266 mmol) were added to a solution of compound **2** (0.064 mmol assuming 100% yield) and compound **1** (0.053 mmol, assuming 100% yield) in dry THF (5 mL), and the mixture was stirred at 60 °C for 20 h under an argon atmosphere. After cooling to room temperature, saturated aqueous EDTA/NH_3 solution (5 mL) was added and stirred for 1 h. CH_2Cl_2 (10 mL) and H_2O (10 mL) were added, the layers were separated, and the aqueous phase was extracted with Et_2O (2 × 20 mL). The organic phases were combined, washed with brine (20 mL), dried ($MgSO_4$), and filtered. Solvent removal and purification by column chromatography (silica gel, petroleum ether/CH_2Cl_2, 3:1, then petroleum ether/ethyl acetate, 4: 1) afforded compound **3·Ma** (15 mg, 6.3 μmol, 13% based on compound **S1**) as a black solid.

^{1}H NMR (700 MHz, $CDCl_3$) δ_H 8.52 (d, J = 8.5 Hz, 4H, H$_d$), 8.20 (d, J = 8.4 Hz, 2H, H$_b$), 8.05 (d, J = 8.4 Hz, 2H, H$_c$), 7.69 (s, 2H, H$_a$), 7.31 (s, 8H, Co Ar−H), 7.26 – 7.21 (m, 8H, Co Ar−H), 7.21 – 7.07 (m, 20H, Co Ar−H), 7.01 (t, J = 7.6 Hz, 8H, H$_e$ & Co Ar−H), 6.73 (t, J = 2.4 Hz, 1H, H$_l$), 6.51 (dd, J = 8.1, 2.3 Hz, 2H, H$_m$), 4.26 (t, J = 7.4 Hz, 4H, H$_{f/k}$), 4.14 (t, J = 6.7 Hz, 4H, H$_{k/f}$), 3.34 (s, 4H, P-CH$_2$), 1.98 (p, J = 6.7 Hz, 4H, H$_{g/j}$), 1.90 (p, J = 6.7 Hz, 4H, H$_{j/g}$), 1.69 (d, J = 3.9 Hz, 8H, H$_{h,i}$), 1.01 (s, 42H, Si-^{i}Pr). **^{13}C{^{1}H} NMR** (176 MHz, $CDCl_3$) δ_C 160.93 (O-C-C$_e$), 160.77, 156.61 (N-C-C-N), 146.22 (C$_a$-C-C$_b$), 136.41 (C$_b$), 133.56 (br, Ar-C), 132.20 (br, Ar-C), 131.82, 131.43 (br, Ar-C), 130.21, 129.83 (C$_n$), 129.62 (C$_d$), 129.20, 128.51 (t, J_{C-P} = 4.8 Hz, Ar-C), 128.39 (t, J_{C-P} = 4.8 Hz, Ar-C), 127.29, 125.36 (C$_a$), 118.94 (C$_c$), 115.08 (C$_e$), 108.57 (C≡C), 108.13 (C$_m$), 101.35 (C≡C), 99.44 (C$_l$), 83.49 (C≡C), 82.08 (C≡C), 70.80 (C≡C), 68.35 (C$_{f/k}$), 67.87 (C$_{k/f}$), 67.30 (C≡C), 35.81 (P-CH$_2$), 29.74 (C$_{j/g}$), 29.39 (C$_{g/j}$), 25.89 (C$_{h/i}$), 25.84 (C$_{i/h}$), 18.86 (CH(CH$_3$)$_3$), 11.56 (CH(CH$_3$)$_3$) *(3 carbons not distinguished)*. **ESI HRMS** *m/z* = 1187.2710 $[M+2H]^{2+}$ ($C_{134}H_{130}Co_4N_2O_{12}P_4Si_2^{2+}$ requires 1187.2715).

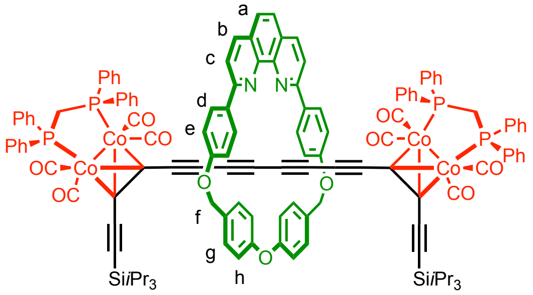

Compound 3·M$_b$: **Method 1.** To a solution of compound **S1** (270 mg, 0.287 mmol) in THF/MeOH (30 mL/10 mL) was added K_2CO_3 (397 mg, 2.87 mmol) at 20 °C, and the solution

was stirred for 30 min. This solution was concentrated to ca. 5 mL and passed through a silica plug (CH_2Cl_2/petroleum ether 1:1), which resulted in a solution of compound **1**. Without further purification, the resulting solution was concentrated to ca. 5 mL, and dry THF (ca. 50 mL) was added; then this resulting solution was again concentrated, and dry THF was added; repeating this for three times gave a solution of compound **1** in THF (45 mL). Compound **S2** (180 mg, 0.246 mmol), complex $\mathbf{M_b}$·CuI (203 mg, 0.271 mmol), and K_2CO_3 (170 mg, 1.23 mmol) were added into a Schlenk tube, and this mixture was vacuumed and refilled with a N_2 gas, repeating for three times. Compound **1** (0.287 mmol, assuming 100% yield) in the THF (45 mL) solution was transferred into the Schlenk tube, without further deoxygenation, this solution was stirred at a N_2 atmosphere at 65 °C for 18 h. After cooling to room temperature, saturated aqueous EDTA/NH_3 (10 mL) was added and stirred for 2 h. CH_2Cl_2 (50 mL) and H_2O (20 mL) were added, the layers were separated, and the aqueous phase was extracted with CH_2Cl_2 (2 × 20 mL). The organic phases were combined, washed with brine (20 mL), dried ($MgSO_4$), and filtered. Solvent was removed, and a short column chromatography (silica gel, petroleum ether/CH_2Cl_2 1:1) was used to remove the less polar by-products, then the eluting solvent of ethyl acetate was used to give a mixture containing the desired products. Then a SEC (SX3, toluene) was used to remove the free macrocycle. Solvent removal and a final purification by column chromatography (silica gel, ethyl acetate/ petroleum ether 1:5 to 1:1) afforded compound $\mathbf{3{\cdot}M_b}$ (55 mg, 24 μmol, 17% based on compound **S1**) and $\mathbf{S3{\cdot}M_b}$ (58 mg, 28 μmol, 11% based on compound **S2**) as black solids.

Method 2. To a solution of compound **S1** (200 mg, 0.213 mmol) in THF/MeOH (5 mL/2 mL) was added K_2CO_3 (147 mg, 1.06 mmol), and the solution was stirred for 10 min at 20 °C. This solution was passed through a silica plug (CH_2Cl_2/petroleum ether 1:1), which resulted in a solution of compound **1**. Without further purification, the resulting solution was concentrated to ca. 5 mL, and dry THF (ca. 20 mL) was added; then this resulting solution was again concentrated, and dry THF was added; repeating this three times gave a solution of compound **1** in THF (20 mL). Separately, compound **2** (0.213 mmol, assuming 100% yield) in dry THF (20 mL) was prepared from compound **S1** (200 mg, 0.213 mmol) as described in the synthetic procedure. Complex $\mathbf{M_b}$·CuI (191 mg, 0.256 mmol) and K_2CO_3 (147 mg, 1.06 mmol) were then added to the solution of compound **2** (0.213 mmol, assuming 100% yield) and compound **1** (0.213 mmol, assuming 100% yield) in dry THF (40 mL), and the mixture was stirred at 60 °C for 20 h under a N_2 atmosphere. After cooling to room temperature, saturated aqueous EDTA/NH_3 solution (20 mL) was added and stirred for 1 h. CH_2Cl_2 (50 mL) and H_2O (20 mL) were added, the layers were separated, and the aqueous phase was extracted with CH_2Cl_2 (2 × 20 mL). The organic phases were combined, washed with brine (20 mL), dried ($MgSO_4$), and filtered. Solvent removal and purification by column chromatography (silica gel, petroleum ether/CH_2Cl_2, 3:1, then petroleum ether/ethyl acetate, 4: 1) afforded compound $\mathbf{3{\cdot}M_b}$ (73 mg, 32 μmol, 15% based on compound **S1**) as a black solid.

IR (ATR): 2943 (w, C–H), 2864 (w, C–H), 2150 (w, C≡C), 2104 (w, C≡C), 2036 (m, C=O), 2013 (s, C=O), 1988 (m, C=O) cm^{-1}. ^{1}**H NMR** (600 MHz, CD_2Cl_2) δ_H 8.21 (d, J = 8.3 Hz, 2H; H_b), 8.08 (d, J = 8.7 Hz, 4H; H_d), 7.92 (d, J = 8.3 Hz, 2H; H_c), 7.73 (s, 2H; H_a), 7.40–7.36 (m, 15H), 7.31–7.24 (m, 13H), 7.18–7.14 (m, 8H), 7.10–7.07 (m, 4H), 7.03–7.01 (m, 12H), 5.30 (s, 4H; H_f), 3.43–3.33 (m, 4H; PCH_2), 1.03–1.02 (m, 42H; *i*Pr_3). ^{13}**C{**^{1}**H} NMR** (151 MHz, CD_2Cl_2) δ_C 204.2, 201.7, 160.0, 158.6, 158.1, 147.1, 137.2 (br), 136.3, 134.6, 134.2, 133.5 (br), 132.7 (t, $J_{C\text{–}P}$ = 6.1 Hz), 131.5 (t, $J_{C\text{–}P}$ = 6.1 Hz), 130.6, 130.1, 128.9 (t, $J_{C\text{–}P}$ = 4.9 Hz), 128.7 (t, $J_{C\text{–}P}$ = 4.9 Hz), 127.6, 127.5, 125.8, 122.8, 120.4, 116.8, 108.9, 101.3, 83.3, 82.5, 72.6, 71.0, 68.2, 67.9,

19.0, 11.9 (3 signals coincident or not observed). **^{31}P{^{1}H} NMR** (202 MHz, CD_2Cl_2) δ_P 38.2. **ESI HRMS *m/z*:** calcd for $C_{130}H_{113}Co_4N_2O_{11}P_4Si_2$ ([M + H]$^+$) 2293.4156, found 2293.4133.

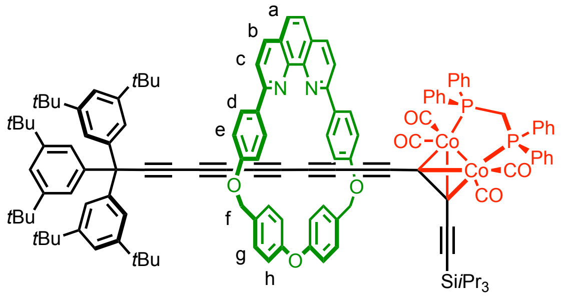

Compound S3·M$_b$: The titled compound **S3·M$_b$** (58 mg, 28 μmol, 11%) was obtained as a black solid as described in the above procedure using **Method 1**.

IR (ATR): 2960 (m, C–H), 2864 (w, C–H), 2034 (m, C=O), 2016 (s, C=O), 1991 (m, C=O) cm^{-1}. **^{1}H NMR** (600 MHz, CD_2Cl_2) δ_H 8.22 (d, J = 8.3 Hz, 2H; H$_b$), 7.98 (d, J = 8.8 Hz, 4H; H$_d$), 7.90 (d, J = 8.3 Hz, 2H; H$_c$), 7.74 (s, 2H; H$_a$), 7.40–7.37 (m, 4H; PPh_2-H), 7.34–7.33 (m, 4H; H$_g$), 7.30–7.26 (m, 13H; H$_h$, *para*-Tr* and PPh_2-H), 7.17–7.14 (m, 4H; PPh_2-H), 7.10–7.07 (m, 2H; PPh_2-H), 7.03–7.01 (m, 4H; PPh_2-H), 6.96 (d, J = 8.8 Hz, 4H; H$_e$), 6.93 (d, J = 1.7 Hz, 6H; *ortho*-Tr*), 5.30, 5.27 (ABq, J = 15.0 Hz, 4H; H$_f$), 3.47–3.32 (m, 2H; PCH_2), 1.18 (s, 54H; *t*Bu), 1.04–1.03 (m, 21H; *i*Pr$_3$). **^{13}C{^{1}H} NMR** (151 MHz, CD_2Cl_2) δ_C 159.8, 158.5, 158.2, 150.7, 147.0, 144.0, 137.1 (br), 136.4, 134.5, 134.3, 133.3 (br), 132.7 (t, $J_{C–P}$ = 6.1 Hz), 131.5 (t, $J_{C–P}$ = 6.1 Hz), 130.6, 130.2, 130.1, 128.9 (t, $J_{C–P}$ = 4.9 Hz), 128.7 (t, $J_{C–P}$ = 4.9 Hz), 127.7, 127.6, 125.8, 124.0, 122.5, 120.9, 120.6, 116.6, 108.7, 101.6, 86.0, 82.0, 71.2, 70.7, 70.3, 68.2, 66.9, 65.2, 64.64, 64.55, 63.8, 57.8, 35.2, 31.5, 19.0, 11.9 (5 signals coincident or not observed). **^{31}P{^{1}H} NMR** (202 MHz, CD_2Cl_2) δ_P 38.3. **ESI HRMS *m/z*:** calcd for $C_{133}H_{133}Co_2N_2O_7P_2Si$ ([M + H]$^+$) 2078.8048, found 2078.8008.

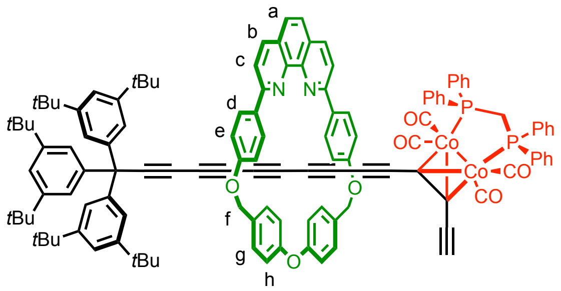

Compound 7·M$_b$: To compound **S3·M$_b$** (45 mg, 0.022 mmol) in THF/H_2O (10/0.1 mL) was added TBAF (0.22 mL, 0.22 mmol, 1.0 M in THF). The solution was stirred at 20 °C, and the reaction was monitored by TLC analysis until deemed complete; ca. 1.5 h. H_2O (10 mL) and CH_2Cl_2 (20 mL) were then added, the layers were separated, and the aqueous phase was extracted with CH_2Cl_2 (2 × 10 mL). The organic phases were combined, washed with brine (20 mL), dried ($MgSO_4$), and filtered. Solvent removal and purification by column chromatography (silica gel, ethyl acetate/petroleum ether 1:2) afforded compound **7·M$_b$** (31 mg, 16 μmol, 75%) as a black solid.

^{1}H NMR (400 MHz, CD_2Cl_2) δ_H 8.22 (d, J = 8.3 Hz, 2H; H$_b$), 7.99 (d, J = 8.8 Hz, 4H; H$_d$), 7.91 (d, J = 8.3 Hz, 2H; H$_c$), 7.74 (s, 2H; H$_a$), 7.38–7.34 (m, 8H; H$_g$ and PPh_2-H), 7.31–7.26 (m, 13H; H$_h$, *para*-Tr* and PPh_2-H), 7.21–7.18 (m, 4H; PPh_2-H), 7.10–7.07 (m, 6H; PPh_2-H), 6.98 (d, J = 8.8 Hz, 4H; H$_e$), 6.95 (d, J = 1.8 Hz, 6H; *ortho*-Tr*), 5.31 (s, 4H; H$_f$), 3.88 (t, J = 1.0 Hz, 1H; C≡CH), 3.51–3.28 (m, 2H; PCH_2), 1.19 (s, 54H; *t*Bu). **^{13}C{^{1}H} NMR** (100 MHz, CD_2Cl_2) δ_C 159.8, 158.5, 158.1, 150.7, 147.0, 144.0, 136.4, 134.5, 134.3, 132.4 (t, $J_{C–P}$ = 6.1 Hz), 131.7 (t, $J_{C–P}$ = 6.1 Hz), 130.6, 130.4, 130.1, 128.84 (t, $J_{C–P}$ = 4.9 Hz), 128.79 (t, $J_{C–P}$ = 4.9 Hz), 127.7, 127.6, 125.9, 123.9, 122.5, 120.9, 120.6, 116.6, 86.1, 86.0, 85.7, 82.2, 81.7, 71.1, 70.7, 70.3, 66.9, 65.1, 64.5, 63.9, 57.8, 35.2, 31.5 (8 signals coincident or not observed). **^{31}P{^{1}H} NMR** (162 MHz, CD_2Cl_2) δ_P 39.1. **ESI HRMS *m/z*:** calcd for $C_{124}H_{113}Co_2N_2O_7P_2$ ([M + H]$^+$) 1922.6715, found 1922.6645.

Compound 4: Tetracobalt tetrayne thread **3** (0.18 g, 1.0 eq., 0.10 mmol) was dissolved in dry THF (30 mL) containing water (0.30 mL). TBAF (1.0 M in THF, 1.0 mL, 10 eq., 1.0 mmol) was added dropwise and the mixture stirred at 20 °C for 30 min.

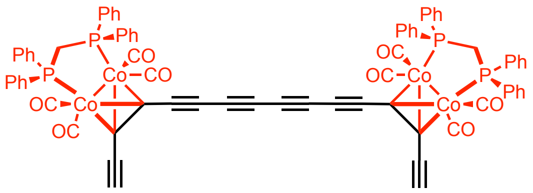

The reaction was quenched with addition of aqueous NH_4Cl solution (20 mL), then Et_2O (20 mL) was added, and the organic layer extracted. The aqueous layer was washed with Et_2O (2 × 10 mL) and the combined extracts washed with brine (20 mL), then dried over Na_2SO_4. The solvent was removed under reduced pressure and the crude material purified by silica chromatography (petroleum ether/CH_2Cl_2, gradient elution from 0 to 100%) to yield deprotected tetracobalt tetrayne thread **4** (0.14 g, 0.98 mmol, 94%) as a red-brown solid.

^{1}H NMR (500 MHz, CD_2Cl_2) δ_H 7.42 – 7.37 (m, 8H, Ar-H), 7.37 – 7.30 (m, 16H, Ar-H), 7.30 – 7.22 (m, 16H, Ar-H), 3.89 (t, J = 1.8 Hz, 2H, C≡CH), 3.47 (t, J = 10.5 Hz, 4H, P-CH_2). **^{13}C{^{1}H} NMR** (126 MHz, CD_2Cl_2) δ_C 202.79 (C≡O), 135.68 (t, $J_{C–P}$ = 21.1 Hz, Ar-C), 134.50 (t, $J_{C–P}$ = 20.4 Hz, Ar-C), 132.29 (t, $J_{C–P}$ = 6.4 Hz, Ar-C), 131.95 (t, $J_{C–P}$ = 6.1 Hz, Ar-C), 130.66 (Ar-C), 130.49 (Ar-C), 128.88 (t, $J_{C–P}$ = 6.4 Hz, Ar-C), 85.81 (C≡CH), 85.69, 83.41, 82.28, 72.07 (Co-C), 70.66, 66.92, 65.24 (Co-C), 37.43 (t, $J_{C–P}$ = 21.3 Hz, P-CH_2). **^{31}P{^{1}H} NMR** (202 MHz, CD_2Cl_2) δ_P 39.24. **ESI HRMS** *m/z* = 1420.9410 $[M+H]^+$ ($C_{74}H_{46}Co_4O_8P_4^+$ requires 1420.9398).

Compound 4·Ma: Tetracobalt [2]rotaxane **3·Ma** (35 mg, 1.0 eq., 15 μmol) was dissolved in THF (15 mL) containing water (0.15 mL). TBAF solution (1.0 M in THF, 0.15 mL, 10 eq., 0.15 mmol) was added dropwise and the mixture stirred at 20 °C for 30 min. The reaction was quenched with addition of aqueous NH_4Cl solution (15 mL), then Et_2O (10 mL) was added, and the organic layer extracted. The aqueous layer was washed with Et_2O (2 × 5 mL) and the combined extracts washed with brine (10 mL), then dried over Na_2SO_4. The solvent was removed under reduced pressure and the crude material purified by silica chromatography (petroleum ether/EtOAc, gradient elution from 0–40%) to yield deprotected tetracobalt [2]rotaxane **4·Ma** (27 mg, 13 μmol, 88%) as a dark brown solid.

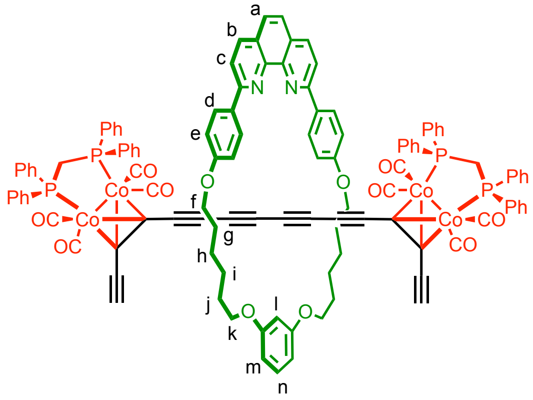

^{1}H NMR (500 MHz, CD_2Cl_2) δ_H 8.52 (d, J = 8.9 Hz, 4H, H_d), 8.26 (d, J = 8.4 Hz, 2H, H_b), 8.10 (d, J = 8.5 Hz, 2H, H_c), 7.74 (s, 2H, H_a), 7.35 – 7.29 (m, 8H, Co Ar−H), 7.28 – 7.23 (m, 8H, H_e & Co Ar−H), 7.23 – 7.18 (m, 16H, Co Ar−H), 7.17 – 7.13 (m, 4H, Co Ar−H), 7.11 – 7.05 (m, 9H, H_n & Co Ar−H), 6.74 (t, J = 2.4 Hz, 1H, H_l), 6.48 (dd, J = 8.2, 2.4 Hz, 2H, H_m), 4.26 (t, J = 7.4 Hz, 4H, $H_{f/k}$), 4.14 (t, J = 6.8 Hz, 4H, $H_{k/f}$), 3.84 (s, 2H, C≡CH), 3.37 (dt, J = 22.4, 11.8 Hz, 4H, P-CH_2), 1.98 (t, J = 7.2 Hz, 4H, $H_{g/j}$), 1.91 (t, J = 6.8 Hz, 4H, $H_{j/g}$), 1.74 – 1.66 (m, 8H, $H_{h,i}$). **^{13}C{^{1}H} NMR** (126 MHz, CD_2Cl_2) δ_C 161.18 (O-C-C_e), 161.14, 156.40 (N-C-C-N), 146.51 (C_a-C-C_b), 136.82 (C_b), 135.91 (t, J_{C-P} = 25.5 Hz, Ar-C), 134.12 (t, J_{C-P} = 20.3 Hz, Ar-C), 132.25 (t, J_{C-P} = 6.5 Hz, Ar-C), 131.99, 131.79 (t, J_{C-P} = 5.9 Hz, Ar-C), 130.63 (Ar-C), 130.32 (Ar-C), 129.82 (C_n), 129.32 (C_d), 128.77 (t, J_{C-P} = 5.2 Hz, Ar-C), 127.75, 125.76 (C_a), 119.17 (C_c), 115.36 (C_e), 108.13 (C_m), 99.90 (C_l), 86.28 (C≡CH), 85.61 (C≡C), 83.81 (C≡C), 82.21 (C≡C), 70.93 (C≡C), 68.68 ($C_{k/f}$), 68.30 ($C_{f/k}$), 67.38 (C≡C),

37.14 (t, J_{C-P} = 21.1 Hz, P-$\underline{C}H_2$), 30.10 ($C_{j/g}$), 29.67 ($C_{g/j}$), 26.29 ($C_{h/i}$), 26.19 ($C_{i/h}$) *(3 carbons not distinguished)*. **^{31}P{^{1}H} NMR** (202 MHz, CD_2Cl_2) δ_P 39.20. **ESI HRMS** *m/z* = 2061.2693 $[M+H]^+$ ($C_{116}H_{89}Co_4N_2O_{12}P_4^+$ requires 2061.2688).

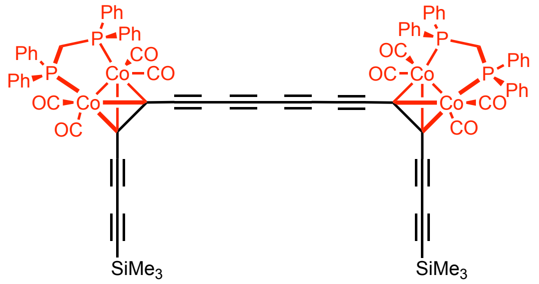

Compound 5: Deprotected tetracobalt thread **4** (240 mg, 1.0 eq., 0.17 mmol) was dissolved in dry CH_2Cl_2 (250 mL). Trimethylsilyl acetylene (1.2 mL, 1.7 g, 50 eq., 8.5 mmol) and copper(I) chloride (0.75 g, 45 eq., 76 mmol) were added and O_2 bubbled through the vigorously-stirred solution. TMEDA (1.0 mL, 40 eq., 6.8 mmol) was added to the reaction mixture. Additional trimethylsilyl acetylene (1.2 mL, 50 eq., 8.5 mmol) was added over a period of 30 min via syringe pump. The reaction was monitored by TLC and, upon completion, was quenched by addition of water (100 mL). The organic layer was extracted and washed with water (2 × 150 mL), then brine (150 mL) before drying over Na_2SO_4. The solvent was removed under reduced pressure and the crude material initially purified by a SiO_2 plug, flushing first with petroleum ether to remove the bis-TMS butadiyne, then 1:1 petroleum ether/CH_2Cl_2. The crude material was then subject to SiO_2 chromatography (petroleum ether/EtOAc, gradient elution from 0 to 40%), to yield the TMS-protected extended thread **5** (0.18 g, 0.11 mmol, 66%) as a red solid. R_f (petroleum ether/EtOAc, 33%) = 0.60.

IR (ATR): 2958 (w, C–H), 2149 (m, C≡C), 2037 (s, C=O), 2015 (s, C=O), 1992 (s, C=O) cm^{-1}. **^{1}H NMR** (600 MHz, CD_2Cl_2) δ_H 7.39 – 7.31 (m, 24H, Ar-$\underline{H}$), 7.31 – 7.25 (m, 16H, Ar-$\underline{H}$), 3.59 – 3.39 (m, 4H, P-C$\underline{H_2}$), 0.26 (s, 18H, $SiMe_3$). **^{13}C{^{1}H} NMR** (151 MHz, CD_2Cl_2) δ_C 202.78 ($\underline{C}$≡O), 202.12 ($\underline{C}$≡O), 134.87 (q, J_{C-P} = 23.0 Hz, Ar-$\underline{C}$), 132.13 (t, J_{C-P} = 6.2 Hz, Ar-$\underline{C}$), 130.68 (d, J_{C-P} = 9.5 Hz, Ar-$\underline{C}$), 129.01 (dt, J_{C-P} = 6.7, 3.2 Hz, Ar-$\underline{C}$), 93.86, 89.85, 83.40, 83.09, 82.75, 80.30, 71.04, 69.84 (br, Co-$\underline{C}$), 67.07, 66.36 (br, Co-$\underline{C}$), 38.50 (t, J_{C-P} = 22.0 Hz, P-$\underline{C}H_2$), -0.21 ($SiMe_3$). **^{31}P{^{1}H} NMR** (243 MHz, CD_2Cl_2) δ_P 39.63. **ESI HRMS** *m/z* = 1615.0386 $[M+H]^+$ ($C_{84}H_{63}Co_4O_8P_4Si_2^+$ requires 1615.0335).

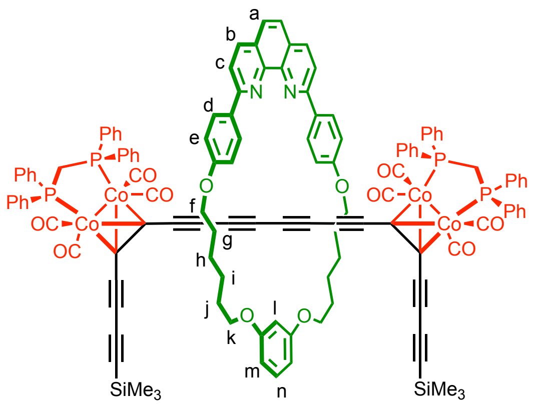

Compound 5·M$_a$: Deprotected tetracobalt [2]rotaxane **4·M$_a$** (30 mg, 1.0 eq., 15 µmol) was dissolved in dry CH_2Cl_2 (20 mL). TMS-acetylene (0.10 mL, 50 eq., 0.72 mmol) and copper(I) chloride (65 mg, 45 eq., 0.68 mmol) were added and the solution stirred vigorously in air for 2 min. TMEDA (68 mg, 87 µL, 40 eq., 0.74 mmol) was added to the reaction mixture. The remaining TMS-acetylene (72 mg, 0.10 mL, 50 eq., 1.46 mmol) was added over a period of 30 min via syringe pump. Saturated aqueous EDTA/NH_3 (5 mL) was added and stirred for 20 min. The organic layer was extracted and washed with water (2 × 20 mL), then brine (20 mL) before drying over Na_2SO_4. The solvent was removed under reduced pressure and the crude material purified by SiO_2 chromatography (petroleum ether/EtOAc, gradient elution from 0 to 33%), to yield the extended tetracobalt [2]rotaxane **5·M$_a$** (23 mg, 10 µmol, 69%) as a red-brown solid. R_f (petroleum ether/EtOAc, 50%) = 0.68.

IR (ATR): 2981 (s, C–H), 2972 (s, C–H), 2889 (m, C–H), 2149 (m, C≡C), 2036 (s, C=O), 2017 (s, C=O), 1993 (s, C=O) cm^{-1}. ^{1}**H NMR** (500 MHz, CD_2Cl_2) δ_H 8.52 (d, J = 8.8 Hz, 4H, H_d), 8.25 (d, J = 8.5 Hz, 2H, H_b), 8.09 (d, J = 8.5 Hz, 2H, H_c), 7.73 (s, 2H, H_a), 7.30 – 7.24 (m, 16H, H_e & Ar-$\underline{H}$), 7.24 – 7.18 (m, 16H, Ar-$\underline{H}$), 7.18 – 7.14 (m, 4H, Ar-$\underline{H}$), 7.13 – 7.08 (m, 9H, H_n & Ar-$\underline{H}$), 6.71 (t, J = 2.4 Hz, 1H, H_l), 6.49 (dd, J = 8.1, 2.3 Hz, 2H, H_m), 4.28 (t, J = 7.3 Hz, 4H, $H_{f/k}$), 4.13 (t, J = 6.6 Hz, 4H, $H_{k/f}$), 3.38 (s, 4H, P-C$\underline{H}_2$), 2.01 (dd, J = 10.6, 3.8 Hz, 4H, $H_{g/j}$), 1.93 (dd, J = 8.2, 4.7 Hz, 4H, $H_{j/g}$), 1.73 (t, J = 3.5 Hz, 8H, $H_{h,i}$), 0.25 (s, 18H, $SiMe_3$). ^{13}**C{^{1}H} NMR** (126 MHz, CD_2Cl_2) δ_C 202.31 ($\underline{C}$≡O, br), 161.24 (O-$\underline{C}$-C_e), 161.12, 156.36 (N-$\underline{C}$-$\underline{C}$-N), 146.50 (C_a-$\underline{C}$-C_b), 136.80 (C_b), 135.04 (t, $J_{C\text{-}P}$ = 22.3 Hz, Ar-$\underline{C}$), 134.41 (t, $J_{C\text{-}P}$ = 21.4 Hz, Ar-$\underline{C}$), 131.99 (t, $J_{C\text{-}P}$ = 6.1 Hz, Ar-$\underline{C}$), 130.68 (Ar-$\underline{C}$), 130.49 (C_n), 129.85 (Ar-$\underline{C}$), 129.26 (C_d), 128.89 (t, $J_{C\text{-}P}$ = 5.0 Hz, Ar-$\underline{C}$), 127.75, 125.76 (C_a), 119.17 (C_c), 115.39 (C_e), 107.93 (C_m), 100.16 (C_l), 93.62, 89.95, 83.62, 83.10, 80.29, 71.29, 68.76 ($C_{k/f}$), 68.26 ($C_{f/k}$), 67.48, 60.63, 38.36 (t, $J_{C\text{-}P}$ = 22.0 Hz, P-$\underline{C}H_2$), 30.11 ($C_{j/g}$), 29.70 ($C_{g/j}$), 26.35 ($C_{h/i}$), 26.29 ($C_{i/h}$), 14.41, -0.20 ($SiMe_3$) (*2 carbons not distinguished*). ^{31}**P{^{1}H} NMR** (202 MHz, CD_2Cl_2) δ_P 39.61. **ESI HRMS** m/z = 2253.3412 [M+H]$^+$ ($C_{126}H_{105}Co_4N_2O_{12}P_4Si_2^+$ requires 2253.3479).

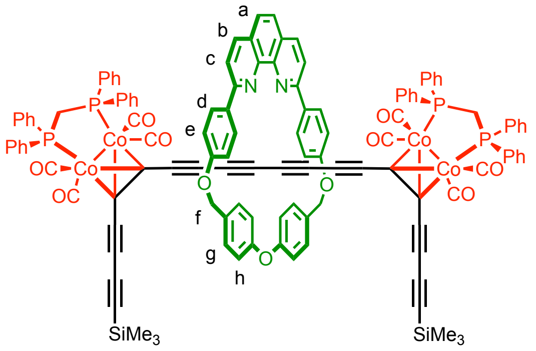

Compound 5·M$_b$: To compound **3·M$_b$** (100 mg, 0.0436 mmol) in THF/H_2O (20/0.2 mL) was added TBAF (0.436 mL, 0.436 mmol, 1.0 M in THF). The solution was stirred at 20 °C, and the reaction was monitored by TLC analysis until deemed complete; ca. 2.5 h. H_2O (10 mL) and CH_2Cl_2 (20 mL) were then added, the layers were separated, and the aqueous phase was extracted with CH_2Cl_2 (2 × 10 mL). The organic phases were combined, washed with brine (20 mL), dried ($MgSO_4$), and filtered. Solvent removal and a silica column plug (ethyl acetate/petroleum ether 1:2) afforded deprotected intermediate **4·M$_b$** as a black solid, without further purification **4·M$_b$** was carried out through the next step. The Glaser-Hay catalyst was prepared by adding CuCl (170 mg, 1.74 mmol) to a solution of TMEDA (252 mg, 2.17 mmol) in CH_2Cl_2 (2 mL), and the solution was stirred for 5 min at 20 °C. To a solution of **4·M$_b$** (0.043 mmol, assuming 100% yield) and trimethylsilyl acetylene (426 mg, 4.34 mmol) in CH_2Cl_2 (60 mL) was added the prepared Glaser-Hay catalyst, and the reaction mixture was stirred at 20 °C, during which a solution of trimethylsilyl acetylene (426 mg, 4.34 mmol) in CH_2Cl_2 (20 mL) was added to the above resulting solution at 20 °C over 3 h via a syringe pump. Saturated aqueous EDTA/NH_3 (5 mL) was added and stirred for 30 min. H_2O (20 mL) was added, the layers were separated, and the aqueous phase was extracted with CH_2Cl_2 (2 × 20 mL). The organic phases were combined, washed with brine (20 mL), dried ($MgSO_4$), and filtered. Solvent removal and purification by column chromatography (silica gel, ethyl acetate/petroleum ether 1:5 to 1:1) afforded compound **5·M$_b$** (53 mg, 24 μmol, 56%) as a black solid.

IR (ATR): 2958 (w, C–H), 2153 (m, C≡C), 2036 (s, C=O), 2015 (s, C=O), 1991 (s, C=O) cm^{-1}. ^{1}**H NMR** (500 MHz, CD_2Cl_2) δ_H 8.22 (d, J = 8.4 Hz, 2H; H_b), 8.10 (d, J = 8.8 Hz, 4H; H_d), 7.93 (d, J = 8.4 Hz, 2H; H_c), 7.73 (s, 2H; H_a), 7.40 (d, J = 8.7 Hz, 4H; H_g), 7.36 (d, J = 8.7 Hz, 4H; H_h), 7.32–7.28 (m, 13H), 7.25–7.19 (m, 17H), 7.11–7.10 (m, 10H), 7.05 (d, J = 8.8 Hz, 4H; H_e), 5.36 (s, 4H; H_f), 3.44–3.35 (m, 4H; PCH_2), 0.26 (s, 18H; $SiCH_3$). ^{13}**C{^{1}H} NMR** (126 MHz, CD_2Cl_2) δ_C 160.0, 158.5, 158.0, 147.0, 136.3, 135.4, 135.2, 135.1, 134.6, 134.4, 134.2, 132.1 (t, $J_{C\text{–}P}$ = 6.3 Hz), 131.9 (t, $J_{C\text{–}P}$ = 6.2 Hz), 130.6, 130.4, 128.9–128.8 (m), 127.54, 127.48,

125.8, 122.7, 121.1, 120.4, 117.3, 116.8, 93.6, 90.0, 84.2, 82.9, 82.3, 80.5, 72.6, 71.0, 67.8, 38.8 (br), –0.2 (2 signals coincident or not observed). **^{31}P{^{1}H} NMR** (202 MHz, CD_2Cl_2) δ_P 39.3. **ESI HRMS *m/z*:** calcd for $C_{122}H_{89}Co_4N_2O_{11}P_4Si_2$ ($[M + H]^+$) 2173.2278, found 2173.2286.

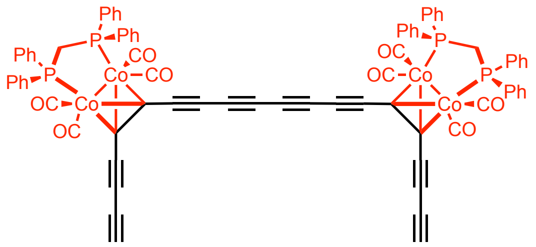

Compound 6: **Method 1**. Extended tetracobalt thread **5** (30 mg, 1.0 eq., 19 µmol) and K_2CO_3 (39 mg, 15 eq., 0.28 mmol) were dissolved in a 1:1 solution of THF (5 mL) and MeOH (5 mL). Water (0.1 mL) was added and the solution stirred at 20 °C for 20 min before addition of water (15 mL) and Et_2O (15 mL). The organic layer was separated, washed with water (10 mL) then brine (10 mL). The organic extract was dried over Na_2SO_4 and the solvent removed under reduced pressure to yield deprotected extended tetracobalt thread **6** (8.3 mg, 5.6 µmol, 30%) as a red-brown solid.; R_f (petroleum ether/EtOAc, 33%) = 0.45.

Method 2. Extended tetracobalt thread **5** (65 mg, 1.0 eq., 40 µmol) was dissolved in THF (25 mL) and water (0.25 mL, 1% v/v). TBAF solution (1.0 M in THF, 40 µL, 1.0 eq., 40 µmol) solution was added and the mixture stirred at 20 °C for 5 min before addition of aqueous sat. aqueous NH_4Cl (25 mL). Et_2O (10 mL) was added and the organic layer extracted, washed with water (20 mL), then brine (20 mL). The combined organic extracts were dried over Na_2SO_4 and the solvent reduced to minimal volume (~1–2 mL) under reduced pressure. $CHCl_3$ (25 mL) was added, then the solvent reduced to minimal volume under reduced pressure (this process was repeated twice more). The solution of deprotected extended tetracobalt tetrayne thread **6** (55 mg, 38 µmol, 94%) in $CHCl_3$ was used directly in the next step. *(Note: This compound is unstable as a dry solid, but stable both in solution and on silica. This compound should be handled in solution.* ***Method 2*** *allows a solution of thread* ***6*** *in $CHCl_3$ to be obtained prior to the coupling step.)*

^{1}H NMR (600 MHz, CD_2Cl_2) δ_H 7.40 – 7.32 (m, 24H, Ar-H), 7.31 – 7.25 (m, 16H, Ar-H), 3.55 – 3.42 (m, 4H, P-CH_2), 2.93 (s, 2H, C≡CH). **^{13}C{^{1}H} NMR** (151 MHz, CD_2Cl_2) δ_C 202.86 (C≡O), 134.77 (br, Ar-C), 132.12 (t, $J_{C\text{-}P}$ = 6.2 Hz, Ar-C), 130.71 (d, $J_{C\text{-}P}$ = 7.7 Hz, Ar-C), 129.01 (q, $J_{C\text{-}P}$ = 3.8 Hz, Ar-C), 83.32, 81.71, 78.88, 74.14 (C≡CH), 71.06, 70.09, 67.05, 38.55 (br, P-CH_2). **^{31}P{^{1}H} NMR** (243 MHz, CD_2Cl_2) δ_P 39.68. **ESI HRMS** *m/z* = 1468.9429 $[M - H]^-$ ($C_{78}H_{45}Co_4O_8P_4^-$ requires 1468.9425).

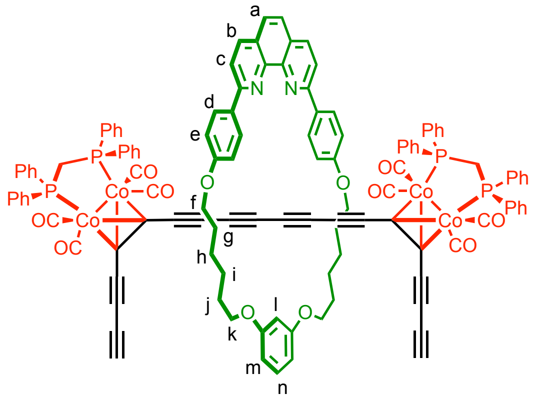

Compound 6·M$_a$: Extended tetracobalt [2]rotaxane **5·M$_a$** (22 mg, 1.0 eq., 9.8 µmol) and potassium carbonate (20 mg, 15 eq., 0.15 mmol) were dissolved in a 1:1 solution of THF (5 mL) and MeOH (5 mL). Water (0.05 mL) was added and the solution stirred at 20 °C for 20 min. The solvent was evaporated under reduced pressure and the crude material purified by SiO_2 chromatography (petroleum ether/EtOAc, gradient elution from 0 to 50%) to yield deprotected extended tetracobalt [2]rotaxane **6·M$_a$** (20 mg, 9.5 µmol, 97%) as a red-brown solid. R_f (petroleum ether/EtOAc, 50%) = 0.49.

^{1}H NMR (600 MHz, CD_2Cl_2) δ_H 8.52 (d, J = 8.8 Hz, 4H, H_d), 8.25 (d, J = 8.4 Hz, 2H, H_b), 8.09 (d, J = 8.4 Hz, 2H, H_c), 7.73 (s, 2H, H_a), 7.30 – 7.26 (m, 14H, H_e & Ar-H), 7.25 – 7.20 (m, 17H, Ar-H), 7.19 – 7.16 (m, 4H, Ar-H), 7.14 – 7.10 (m, 10H, H_n & Ar-H), 6.72 (t, J = 2.3 Hz, 1H, H_l), 6.49 (dd, J = 8.1, 2.3 Hz, 2H, H_m), 4.27 (t, J = 7.3 Hz, 4H, $H_{f/k}$), 4.13 (t, J = 6.7 Hz, 4H, $H_{k/f}$), 3.38 (s, 4H, P-CH_2), 2.87 (s, 2H, C≡CH), 2.00 (t, J = 9.1 Hz, 4H, $H_{g/j}$), 1.93 (t, J = 6.6 Hz, 4H, $H_{j/g}$), 1.73 (p, J = 3.5 Hz, 8H, $H_{h,i}$). **^{13}C{^{1}H} NMR** (151 MHz, CD_2Cl_2) δ_C 202.55 (C≡O, br), 161.24 (O-C-C_e), 161.15, 156.39 (N-C-C-N), 146.53 (C_a-C-C_b), 136.81 (C_b), 135.01 (t, $J_{C\text{-}P}$ = 21.9 Hz, Ar-C), 134.40 (t, $J_{C\text{-}P}$ = 21.3 Hz, Ar-C), 132.00 (t, $J_{C\text{-}P}$ = 6.0 Hz, Ar-C), 130.72 (Ar-C), 130.54 (C_n), 129.86 (Ar-C), 129.28 (C_d), 128.91 (td, $J_{C\text{-}P}$ = 5.0, 2.3 Hz, Ar-C), 127.77, 125.77 (C_a), 119.18 (C_c), 115.40 (C_e), 107.97 (C_m), 100.18 (C_l), 83.59, 83.14, 82.02, 78.85, 74.03 (C≡CH), 71.35, 70.12, 68.77 ($C_{k/f}$), 68.29 ($C_{f/k}$), 67.51, 38.43 (t, $J_{C\text{-}P}$ = 21.8 Hz, P-CH_2), 30.11 ($C_{j/g}$), 29.71 ($C_{g/j}$), 26.36 ($C_{i/h}$), 26.30 ($C_{h/i}$) (*Two C-Co resonances not observed due to extremely long relaxation times*). **^{31}P{^{1}H} NMR** (243 MHz, CD_2Cl_2) δ_P 39.63. **ESI HRMS** *m/z* = 2109.2695 $[M + H]^+$ ($C_{120}H_{89}Co_4N_2O_{12}P_4^+$ requires 2109.2688).

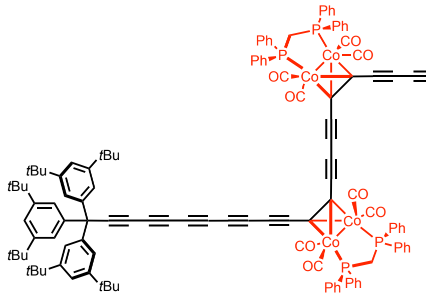

mC28

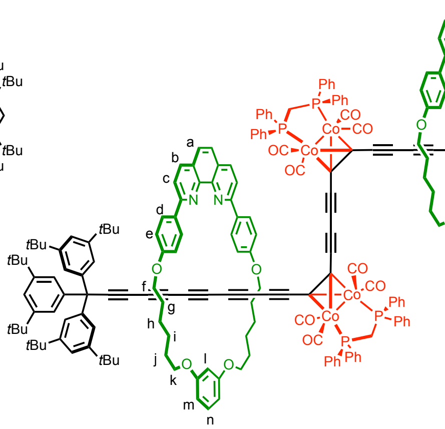

mC28·(M$_a$)$_2$

Compounds *m*C28 and ***m*C28·(M$_a$)$_2$** were synthesized as previously reported.[6]

Compound *m*C28·(M$_b$)$_2$: To compound **7·M$_b$** (50.0 mg, 0.026 µmol) in CH_2Cl_2 (10 mL) was added a solution of CuCl (26 mg, 0.26 mmol) and TMEDA (39 µL, 0.26 mmol) in CH_2Cl_2 (2 mL), and the solution was stirred at 20 °C for 40 min. Saturated aqueous EDTA/NH_3 (5 mL) was added and stirred for 20 min. H_2O (20 mL) was then added, the layers were separated, and the aqueous phase was extracted with CH_2Cl_2 (2 × 10 mL). The organic phases were combined, washed with brine (20 mL), dried ($MgSO_4$), and filtered. Solvent removal and purification by column chromatography (silica gel, ethyl acetate/petroleum ether 1:5 to 1:1) afforded compound ***m*C28·(M$_b$)$_2$** (34 mg, 8.8 µmol, 68%) as a black solid.

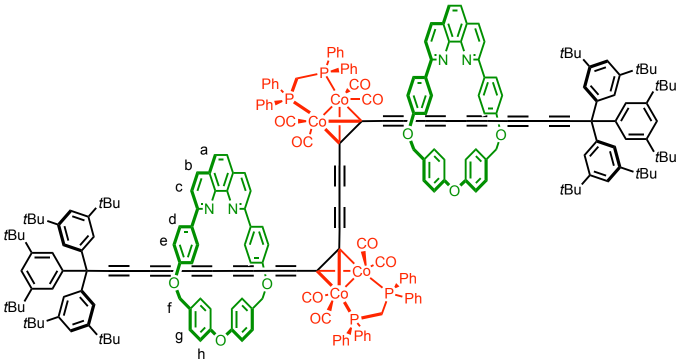

IR (ATR): 2962 (m, C–H), 2927 (m, C–H), 2871 (w, C–H), 2160 (w, C≡C), 2033 (m, C=O), 2016 (s, C=O), 1991 (m, C=O) cm^{-1}. **UV-vis** ($CHCl_3$) λ_{max} (ε): 284 (193000), 318 (195000), 414 (104000), 582 (sh, 6510) nm. **^{1}H NMR** (500 MHz, CD_2Cl_2) δ_H 8.15 (d, J = 8.3 Hz, 4H; H_b), 7.98 (d, J = 8.8 Hz, 8H; H_d), 7.85 (d, J = 8.3 Hz, 4H; H_c), 7.68 (s, 4H; H_a), 7.31 (d, J = 8.6 Hz, 8H; H_g), 7.29 (t, J = 1.8 Hz, 6H; *para*-Tr*), 7.26 (d, J = 8.6 Hz, 8H; H_h), 7.25–7.21 (m, 18H; PPh_2-H), 7.16–7.11 (m, 22H; PPh_2-H), 6.97–6.94 (m, 20H; H_e and *ortho*-Tr*),

5.26, 5.22 (ABq, J_{AB} = 15.6 Hz, 8H; H_f), 3.51–3.23 (m, 4H; PCH_2), 1.18 (s, 54H; *t*Bu). **^{13}C{^{1}H} NMR** (126 MHz, CD_2Cl_2) δ_C 159.8, 158.5, 158.1, 150.7, 147.0, 144.0, 136.4, 135.3, 135.1, 134.9, 134.4, 134.3, 132.3 (t, J_{C-P} = 5.7 Hz), 131.7 (t, J_{C-P} = 5.7 Hz), 130.6, 130.5, 130.1, 129.00 (t, J_{C-P} = 4.9 Hz), 128.85 (t, J_{C-P} = 4.9 Hz), 127.6, 127.5, 125.8, 123.9, 122.5, 120.9, 120.6, 116.6, 88.2, 86.0, 85.9, 82.7, 82.0, 71.7, 70.6, 70.3, 67.2, 65.1, 64.6, 64.0, 60.6, 57.8, 35.2, 31.5 (2 signals coincident or not observed). **^{31}P{^{1}H} NMR** (202 MHz, CD_2Cl_2) δ_P 39.6. **ESI HRMS *m/z*:** calcd for $C_{248}H_{223}Co_4N_4O_{14}P_4$ ([M + H]$^+$) 3842.3201, found 3842.3034. **GPC** (analytical, THF/pyridine 1% v/v) retention time: 36.18 min.

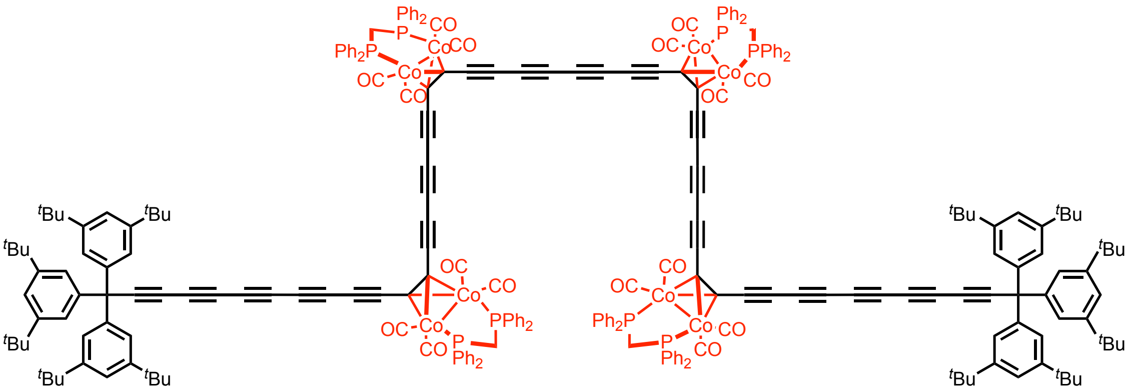

Compound *m*C48: Dry $CHCl_3$ (25 mL) and diisopropylamine (110 mg, 0.15 mL, 80 eq., 1.1 mmol) was added to bis(triphenylphosphine)palladium(II) dichloride (25 mg, 2.7 eq., 36 µmol), copper(I) iodide (34 mg, 13 eq., 0.18 mmol) and benzoquinone (44 mg, 30 eq., 0.41 mmol). The mixture was stirred in air before addition of a mixture of deprotected Tr* cobalt thread **7** (56 mg, 3.0 eq., 41 µmol) and extended tetracobalt thread **6** (20 mg, 1.0 eq., 14 µmol) in dry $CHCl_3$ (5 mL). A drying tube was fitted and the reaction was stirred at 20 °C in air for 2 h. Water (30 mL) was added then the organic layer extracted and washed with water (30 mL) then brine (30 mL). The organic extract was dried over Na_2SO_4, then the solvent removed under reduced pressure. The crude material was purified by silica chromatography (petroleum ether/ CH_2Cl_2). The crude material was purified by recycling GPC (THF) to yield octacobalt ***m*C48** (29 mg, 6.9 µmol, 51% with respect to compound **6**) and dodecacobalt ***m*C68** (7.0 mg, 1.2 µmol, 18% with respect to compound **6**) as dark-brown solids.

IR (ATR): 2981 (s, C–H), 2971 (s, C–H), 2889 (m, C–H), 2152 (m, C≡C), 2019 (s, C=O), 1996 (s, C=O) cm^{-1}. **^{1}H NMR** (600 MHz, CD_2Cl_2) δ_H 7.41 – 7.33 (m, 46H, Co Ar-H & Tr* Ar-H), 7.33 – 7.28 (m, 30H, Co Ar-H), 7.28 – 7.24 (m, 10H, Co Ar-H), 6.97 (d, J = 1.8 Hz, 12H, Tr* Ar-H), 3.66 – 3.34 (m, 8H, P-CH2), 1.22 (s, 108H, ^{t}Bu). **^{13}C{^{1}H} NMR** (151 MHz, CD_2Cl_2) δ_C 202.34 (br, C≡O), 150.80 (Tr* Ar-C), 143.83 (Tr* Ar-C), 135.13 – 134.22 (m, Co Ar-C), 132.29 – 131.91 (m, Co Ar-C), 130.94 – 130.65 (m, Co Ar-C), 129.14 – 128.90 (m, Co Ar-C), 123.99 (Tr* Ar-C), 121.01 (Tr* Ar-C), 87.03, 84.81, 84.73, 84.37, 84.30, 83.66, 83.36, 82.54, 82.17, 71.62, 71.42, 71.34, 70.16, 69.82, 67.29, 66.50, 64.54, 63.78, 63.69, 63.64, 57.84 (CAr_3), 38.87 – 38.21 (m, P-CH_2), 35.17 ($C(CH_3)_3$), 31.51 ($C(CH_3)_3$) *(13 carbons not distinguished and ^{31}P–^{13}C couplings are not resolved due to overlapping signals)*. **^{31}P{^{1}H} NMR** (243 MHz, CD_2Cl_2) δ_P 39.63.

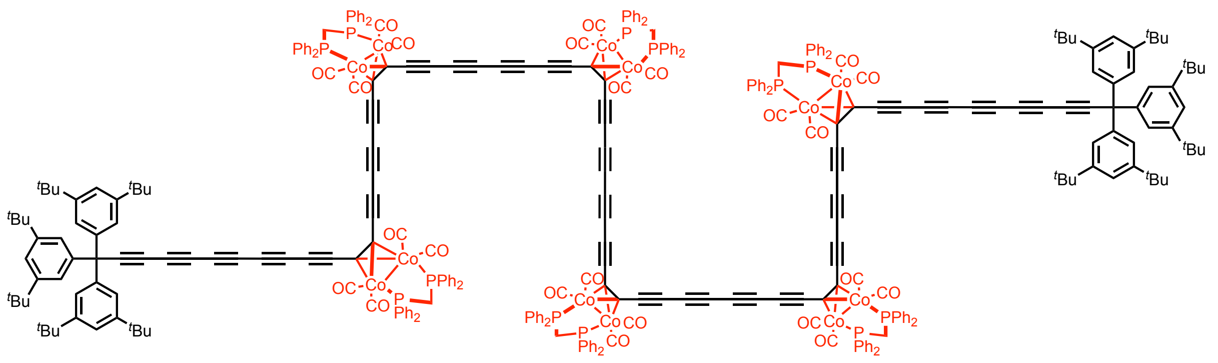

Compound *m*C68: The titled compound ***m*C68** (7.0 mg, 1.2 µmol, 18%) was obtained as a dark-brown solid as described in the above procedure.

IR (ATR): 2980 (m, C–H), 2970 (m, C–H), 2903 (w, C–H), 2149 (m, C≡C), 2017 (s, C=O), 1995 (s, C=O) cm^{-1}. **^{1}H NMR** (600 MHz, CD_2Cl_2) δ_H 7.40 – 7.33 (m, 69H, Co Ar-H & Tr* Ar-H), 7.32 – 7.24 (m, 57H, Co Ar-H), 6.96 (d, J = 1.8 Hz, 12H, Tr* Ar-H), 3.62 – 3.35 (m, 12H, P-CH$_2$), 1.21 (s, 108H, ^{t}Bu). **^{13}C{^{1}H} NMR** (151 MHz, CD_2Cl_2) δ_C 201.12 (br, C≡O), 150.80 (Tr* Ar-C), 143.82 (Tr* Ar-C), 135.13 – 134.22 (m, Co Ar-C), 132.35 – 131.86 (m, Co Ar-C), 130.97 – 130.62 (m, Co Ar-C), 129.25 – 128.78 (m, Co Ar-C), 123.99 (Tr* Ar-C), 121.01 (Tr* Ar-C), 87.02, 84.80, 84.74, 84.37, 84.30, 83.74, 83.63, 83.54, 83.45, 83.38, 83.35, 82.54, 71.61, 71.52, 71.48, 71.35, 70.16, 69.83, 67.35, 67.26, 67.18, 66.49, 64.53, 63.78, 63.68, 63.64, 57.84, 38.84 – 38.34 (m, P-CH$_2$), 35.17 (C(CH$_3$)$_3$), 31.51 (C(CH$_3$)$_3$) *(24 carbons not distinguished and ^{31}P–^{13}C couplings are not resolved due to overlapping signals)*. **^{31}P{^{1}H} NMR** (243 MHz, CD_2Cl_2) δ_P 39.66.

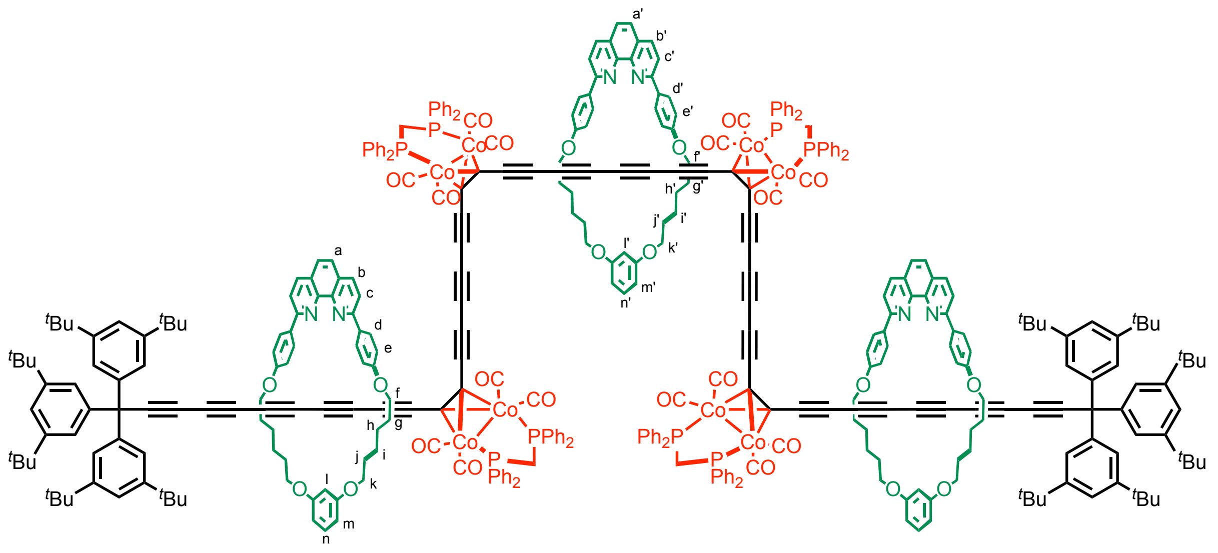

Compound *m*C48·(M$_a$)$_3$: Dry $CHCl_3$ (75 mL) and diisopropylamine (140 mg, 0.19 mL, 80 eq., 1.4 mmol) was added to bis(triphenylphosphine)palladium(II) dichloride (32 mg, 2.7 eq., 45 µmol), copper(I) iodide (43 mg, 13 eq., 0.22 mmol) and benzoquinone (55 mg, 30 eq., 0.51 mmol). The mixture was stirred in air before addition of a mixture of deprotected Tr* [2]rotaxane **7·M$_a$** (140 mg, 4.0 eq., 67 µmol) and deprotected extended tetracobalt [2]rotaxane **6·M$_a$** (36 mg, 1.0 eq., 17 µmol) in dry $CHCl_3$ (15 mL) over 1 hour via syringe pump. After complete addition, the reaction was stirred at 20 °C in air for a further 1 hour. EDTA/NH_3 solution (75 mL) was then added, and the mixture stirred for 1 h. The organic layer was extracted, washed with water (50 mL) then brine (50 mL), before being dried over Na_2SO_4 and the solvent removed under reduced pressure.

The crude material was purified by recycling GPC (THF) to yield octacobalt [4]rotaxane ***m*C48·(M$_a$)$_3$** (29.4 mg, 4.8 μmol, 28%), dodecacobalt [5]rotaxane ***m*C68·(M$_a$)$_4$** (11.1 mg, 1.34 μmol, 16%) and hexadecacobalt [6]rotaxane ***m*C88·(M$_a$)$_5$** (3.3 mg, 0.32 μmol, 6%) as dark-brown solids. *(Note: tetracobalt [3]rotaxane **mC28·(M$_a$)$_2$** is also formed, but not isolated.)*

IR (ATR) (only selected signals) 2961 (C–H), 2866 (C–H), 2149 (C≡C), 2019 (s, C=O), 1995 (s, C=O) cm^{-1}. **UV-vis** ($CHCl_3$) λ_{max} (ε) 589 (15200), 416 (153000), 319 (260000), 291 (292000) nm. **^{1}H NMR** (600 MHz, CD_2Cl_2) δ_H 8.49 (d, J = 8.3 Hz, 4H, H$_{d'}$), 8.46 (d, J = 8.3 Hz, 8H, H$_d$), 8.22 (d, J = 8.5 Hz, 4H, H$_b$), 8.19 (d, J = 8.4 Hz, 2H, H$_{b'}$), 8.09 – 7.99 (m, 6H, H$_{c,c'}$), 7.71 (s, 4H, H$_a$), 7.68 (s, 2H, H$_{a'}$), 7.31 – 7.27 (m, 16H, Cr Ar-H), 7.26 – 7.17 (m, 65H, H$_{e,e'}$, Co & Tr* Ar-H), 7.15 – 7.10 (m, 17H, Co Ar-H), 7.09 – 7.05 (m, 3H, H$_{n,n'}$), 6.91 (d, J = 1.8 Hz, 12H, Tr* Ar-H), 6.73 (t, J = 2.4 Hz, 1H, H$_{l'}$), 6.64 (t, J = 2.4 Hz, 2H, H$_l$), 6.48 (dd, J = 8.1, 2.2 Hz, 2H, H$_{m'}$), 6.46 (dd, J = 8.1, 2.3 Hz, 4H, H$_m$), 4.29 – 4.11 (m, 16H, H$_{f,k}$), 4.07 (t, J = 6.6 Hz, 8H, H$_{f',k'}$), 3.64 – 3.16 (m, 8H, P-CH$_2$), 2.01 – 1.86 (m, 24H, H$_{g,j}$ & H$_{g'j'}$), 1.75 – 1.70 (m, 8H, H$_{h',i'}$), 1.66 (d, J = 5.5 Hz, 16H, H$_{h,i}$), 1.14 (s, 108H, ^{t}Bu). **^{13}C{^{1}H} NMR** (151 MHz, CD_2Cl_2) δ_C 201.65 (br, C≡O), 161.23, 161.16, 161.13, 161.07, 156.37, 150.70, 146.50, 143.81, 136.81, 134.97 (t, J_{C-P} = 22.3 Hz), 134.61 – 134.02 (m), 132.25 – 131.67 (m), 130.75, 130.64, 130.60, 129.90, 129.09 – 128.84 (m), 127.76, 127.73, 125.77, 123.93, 120.94, 119.21, 115.41, 115.32, 107.98, 107.89, 100.33, 100.26, 86.88, 85.14, 84.72, 84.25, 83.89, 83.54, 82.84, 82.26, 77.96, 77.75, 77.54, 74.51, 74.25, 71.77, 71.68, 71.38, 70.45, 69.98, 68.77, 68.63, 68.31, 68.22, 67.78, 66.92, 64.82, 64.29, 64.08, 63.83, 57.84, 38.34 (br), 31.47, 30.15, 30.05, 29.74, 29.64, 26.40, 26.32, 26.28 *(4 carbonyl resonances not distinguished due to overlap and broadening; 12 Ar-C resonances not observed due to overlap)*. **^{31}P{^{1}H} NMR** (243 MHz, CD_2Cl_2) δ_P 39.54. **ESI HRMS** m/z = 3053.9266 $[M+2H]^{2+}$ ($C_{376}H_{342}Co_8N_6O_{28}P_8^{2+}$ requires 3053.9034). **GPC** (analytical, THF/pyridine 1% v/v) retention time: 35.4 min.

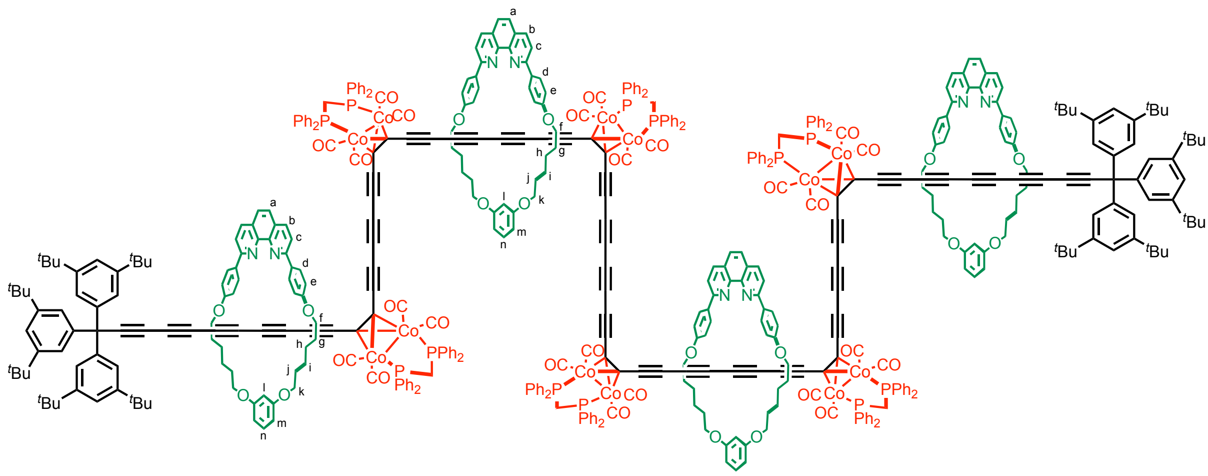

Compound *m*C68·(M$_a$)$_4$: The titled compound ***m*C68·(M$_a$)$_4$** (11.1 mg, 1.34 μmol, 16%) was obtained as a dark-brown solid as described in the above procedure.

IR (ATR) (only selected signals) 2951 (C–H), 2865 (C–H), 2148 (C≡C), 2019 (s, C=O), 1995 (s, C=O) cm^{-1}. **UV-vis** ($CHCl_3$) λ_{max} (ε) 592 (31500), 449 (286000), 422 (301000), 346 (417000), 322 (462000), 291 (520000) nm. **^{1}H NMR** (600 MHz, CD_2Cl_2) δ_H 8.50 (d, J = 8.8 Hz, 8H, H$_d$), 8.45 (d, J = 8.9 Hz, 8H, H$_d$), 8.21 (d, J = 8.4 Hz, 4H, H$_b$), 8.18 (d, J = 8.4 Hz, 4H, H$_b$), 8.04 (t, J = 8.0 Hz, 8H, H$_c$), 7.70 (s, 4H, H$_a$), 7.67 (s, 4H, H$_a$), 7.31 – 7.16 (m, 118H, H$_e$, Tr* Ar-H & Co Ar-H), 7.16 – 7.05 (m, 28H, H$_n$ & Co Ar-H), 6.90 (d, J = 1.8 Hz, 12H, Tr* Ar-H), 6.73 (t, J = 2.4 Hz, 2H, H$_l$), 6.64 (t, J = 2.4 Hz, 2H, H$_l$), 6.49 (dd, J = 8.1, 2.3 Hz, 4H, H$_m$), 6.46 (dd, J = 8.1, 2.3 Hz, 4H, H$_m$), 4.27 (t, J = 7.3 Hz, 8H, H$_{f/k}$), 4.22 – 4.16 (m, 8H, H$_{f/k}$), 4.13 (t, J = 6.6 Hz,

8H, $H_{k/f}$), 4.06 (t, J = 6.6 Hz, 8H, $H_{k/f}$), 3.58 – 3.26 (m, 12H, P-C$\underline{H}_2$), 2.03 – 1.84 (m, 32H, $H_{g,j}$), 1.78 – 1.61 (m, 32H, $H_{h,i}$), 1.13 (s, 108H, ^{t}Bu). **^{13}C{^{1}H} NMR** (151 MHz, CD_2Cl_2) δ_C 161.22, 161.16, 161.13, 161.06, 156.36, 150.69, 146.50, 143.80, 136.81, 135.15 – 134.72 (m), 134.63 – 134.16 (m), 132.36 – 131.67 (m), 131.03 – 130.48 (m), 129.90, 129.29, 129.23, 129.14 – 128.77 (m), 127.75, 125.77, 123.92, 120.93, 119.21, 115.40, 115.31, 107.97, 107.88, 100.31, 100.26, 86.86, 85.12, 84.71, 84.61, 84.25, 83.98, 83.70, 83.47, 83.31, 77.96, 77.75, 77.54, 73.97, 71.99, 71.85, 71.69, 71.48, 71.37, 70.99, 70.42, 69.97, 68.76, 68.62, 68.31, 68.21, 67.85, 67.22, 67.15, 66.91, 64.81, 64.30, 64.07, 63.83, 57.83, 38.35, 37.25, 35.10, 31.46, 30.15, 30.05, 29.73, 29.64, 26.41, 26.35, 26.31, 26.27, 25.99 *(9 Ar-C resonances not observed due to overlap; 6 Co-C resonances not observed due to their extremely long relaxation times and ^{31}P–^{13}C couplings are not resolved due to overlapping signals)*. **^{31}P{^{1}H} NMR** (243 MHz, CD_2Cl_2) δ_P 39.60. **ESI HRMS** *m/z* = 2738.3530 $[M+3H]^{3+}$ ($C_{496}H_{429}Co_{12}N_8O_{40}P_{12}^{3+}$ requires 2738.3533). **GPC** (analytical, THF/pyridine 1% v/v) retention time: 34.6 min.

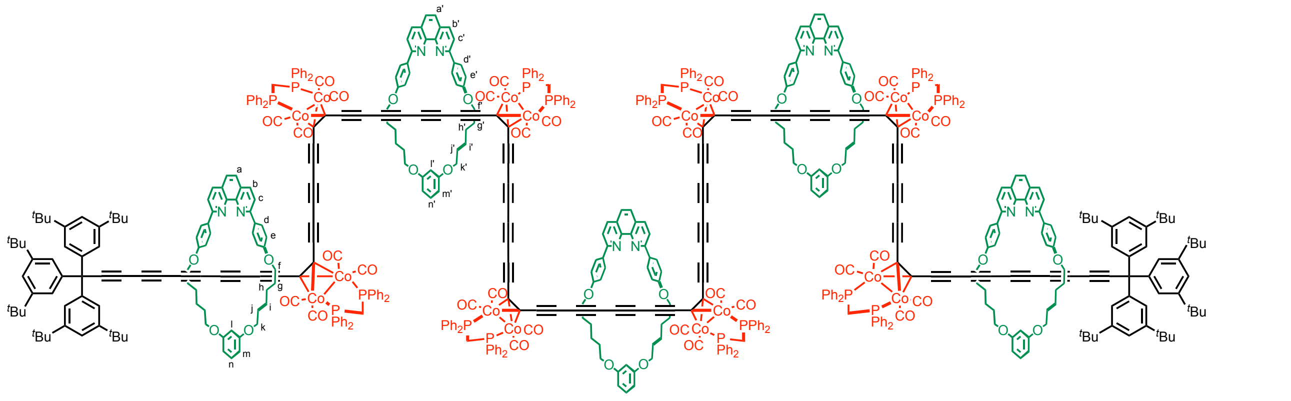

Compound *m*C88·(M$_a$)$_5$: The titled compound ***m*C88·(M$_a$)$_5$** (3.3 mg, 0.32 µmol, 6%) was obtained as a dark-brown solid as described in the above procedure.

IR (ATR) (only selected signals) 2952 (C–H), 2866 (C–H), 2147 (C≡C), 2018 (s, C=O), 1995 (s, C=O) cm^{-1}. **UV-vis** ($CHCl_3$) λ_{max} (ε) 592 (48200), 450 (454000), 424 (461000), 344 (620000), 323 (667000), 291 (741000) nm. **^{1}H NMR** (600 MHz, CD_2Cl_2) δ_H 8.59 – 8.48 (m, 12H, H_d), 8.45 (d, J = 8.6 Hz, 8H, H_d), 8.20 (dd, J = 18.8, 8.4 Hz, 10H, $H_{b,b'}$), 8.10 – 8.00 (m, 10H, $H_{c,c'}$), 7.70 (d, J = 2.4 Hz, 4H, H_a), 7.66 (d, J = 3.3 Hz, 6H, $H_{a'}$), 7.31 – 7.16 (m, 153H, $H_{e,e'}$, Tr* Ar-$\underline{H}$ & Co Ar-$\underline{H}$), 7.10 (ddd, J = 31.4, 15.1, 7.8 Hz, 38H, $H_{n,n'}$ & Co Ar-$\underline{H}$), 6.90 (d, J = 1.8 Hz, 12H, Tr* Ar-$\underline{H}$), 6.77 – 6.71 (m, 3H, $H_{l'}$), 6.64 (t, J = 2.3 Hz, 2H, H_l), 6.49 (ddd, J = 8.3, 6.5, 2.3 Hz, 6H, $H_{m'}$), 6.46 (dd, J = 8.1, 2.3 Hz, 4H, H_m), 4.31 – 4.25 (m, 10H, $H_{f,k/f',k'}$), 4.24 – 4.16 (m, 10H, $H_{f,k/f',k'}$), 4.14 (td, J = 6.7, 3.7 Hz, 10H, $H_{k,f/k',f'}$), 4.06 (t, J = 6.6 Hz, 10H, $H_{k,f/k',f'}$), 3.69 – 3.16 (m, 16H, P-C$\underline{H}_2$), 2.03 – 1.85 (m, 40H, $H_{g,j}$ & $H_{g'j'}$), 1.77 – 1.63 (m, 40H, $H_{h,i}$ & $H_{h'i'}$), 1.13 (s, 108H, ^{t}Bu). **^{13}C{^{1}H} NMR** (151 MHz, CD_2Cl_2) δ_C 201.89 (br, $\underline{C}$≡O), 161.21, 161.16, 161.12, 161.06, 156.34, 150.69, 146.50, 143.80, 136.80, 135.12 – 134.63 (m), 134.61 – 134.04 (m), 132.10 – 131.96 (m), 131.91, 131.86, 130.76, 130.74, 130.67, 130.61, 129.90, 129.28, 129.23, 129.02 – 128.92 (m), 127.75, 125.76, 123.92, 120.93, 119.20, 115.40, 115.31, 107.96, 107.88, 100.31, 100.26, 86.97, 86.86, 85.33, 85.13, 84.72, 84.24, 83.97, 83.77, 83.70, 83.51, 83.37, 82.84, 82.23, 77.96, 77.75, 77.53, 71.98, 71.88, 71.78, 71.69, 71.48, 71.38, 70.43, 69.97, 68.76, 68.61, 68.31, 68.20, 67.86, 67.82, 67.77, 67.71, 67.68, 67.53, 67.24, 66.91, 64.81, 64.29, 64.07, 63.83, 57.83, 38.95 – 38.13 (m), 35.10, 31.46, 30.15, 30.05, 29.74, 29.64, 26.41, 26.35, 26.31, 26.28 *(carbonyl and 11 Ar-C resonances not distinguished due to overlap; 8 Co-C resonances not observed due to their extremely long*

relaxation times; ³¹P–¹³C couplings are not resolved due to overlapping signals). **^{31}P{^{1}H} NMR** (243 MHz, CD_2Cl_2) δ_P 39.63. **ESI HRMS** *m/z* = 2064.6663 $[M+5H]^{5+}$ $C_{616}H_{517}Co_{16}N_{10}O_{52}P_{16}^{5+}$ requires 2064.6641). **GPC** (analytical, THF/pyridine 1% v/v) retention time: 34.0 min.

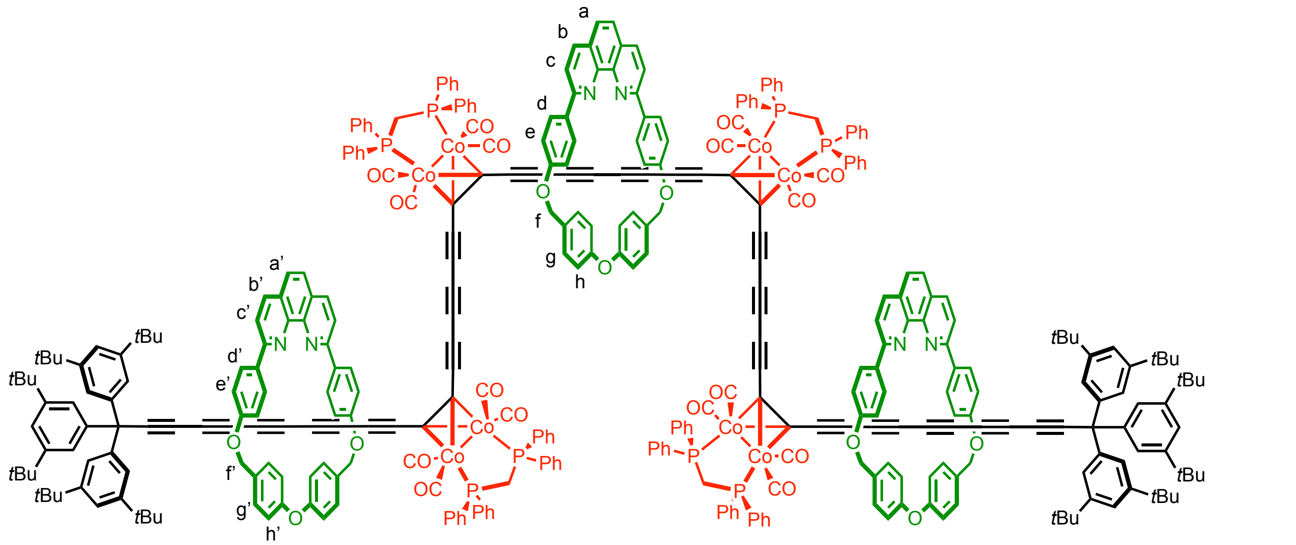

Compound *m*C48·(M$_b$)$_3$: To compound **5·M$_b$** (45 mg, 0.021 mmol) in THF/MeOH (10/10 mL) was added K_2CO_3 (28.6 mg, 0.21 mmol), and the solution was stirred at rt for 1 h. H_2O (20 mL) and CH_2Cl_2 (30 mL) were then added, the layers were separated, and the aqueous phase was extracted with CH_2Cl_2 (2 × 10 mL). The organic phases were combined, washed with brine (20 mL), dried ($MgSO_4$), and filtered. Solvent removal and purification by silica plug (ethyl acetate/petroleum ether 1:2) afforded a black solid **6·M$_b$**, which was redissolved in $CHCl_3$ (20 mL). To this resulting solution was added compound **7·M$_b$** (60 mg, 0.031 mmol). Then, a solution of $Pd(PPh_3)_2Cl_2$ (44 mg, 0.062 mmol), CuI (59 mg, 0.31 mmol), benzoquinone (45 mg, 0.42 mmol), and diisopropylamine (0.24 mL, 1.7 mmol) in $CHCl_3$ (10 mL) was added to the resulting solution, and this reaction mixture was stirred for 2 h at 20 °C. Saturated aqueous EDTA/NH_3 (25 mL) was added and stirred for 30 min. H_2O (20 mL) was added, the layers were separated, and the aqueous phase was extracted with CH_2Cl_2 (2 × 20 mL). The organic phases were combined, washed with brine (20 mL), dried ($MgSO_4$), and filtered. Solvent removal and purification by recycling GPC (THF) afforded compound ***m*C48·(M$_b$)$_3$** (19 mg, 3.2 μmol, 16%), ***m*C68·(M$_b$)$_4$** (11 mg, 1.4 μmol, 14%), ***m*C88·(M$_b$)$_5$** (5 mg, 0.5 μmol, 7%), ***m*C108·(M$_b$)$_6$** (2.0 mg, 0.17 μmol, 3%), and ***m*C128·(M$_b$)$_7$** (0.8 mg, 0.06 μmol, 1%) as black solids. *(Note: tetracobalt [3]rotaxane **m*C28·(M$_b$)$_2$** is also formed, but not isolated.)*

IR (ATR): 2963 (m, C–H), 2907 (w, C–H), 2868 (w, C–H), 2150 (w, C≡C), 2036 (m, C=O), 2018 (s, C=O), 1994 (m, C=O) cm^{-1}. **UV-vis** ($CHCl_3$) λ_{max} (ε): 284 (304000), 318 (298000), 422 (183000), 582 (sh, 17900) nm. **^{1}H NMR** (400 MHz, CD_2Cl_2) δ_H 8.18 (d, *J* = 8.4 Hz, 4H; $H_{b'}$), 8.17 (d, *J* = 8.4 Hz, 2H; H_b), 8.10 (d, *J* = 8.7 Hz, 4H; H_d), 7.98 (d, *J* = 8.8 Hz, 8H; $H_{d'}$), 7.90 (d, *J* = 8.4 Hz, 2H; H_c), 7.88 (d, *J* = 8.4 Hz, 4H; $H_{c'}$), 7.70 (s, 4H; $H_{a'}$), 7.67 (s, 2H; H_a), 7.41 (s, 6H), 7.35–7.27 (m, 42H), 7.24–7.23 (m, 38H), 7.15–7.11 (m, 24H), 7.05 (d, *J* = 8.7 Hz, 4H; H_e), 6.97 (d, *J* = 8.8 Hz, 8H; $H_{e'}$), 6.93 (d, *J* = 1.7 Hz, 12H; *ortho*-Tr*), 5.33 (s, 4H; H_f), 5.30 (s, 8H; $H_{f'}$), 3.53–3.31 (m, 8H; PCH_2), 1.17 (s, 108H; *t*Bu). **^{13}C{^{1}H} NMR** (151 MHz, CD_2Cl_2) δ_C 160.1, 159.8, 159.3, 158.6, 158.5, 158.1, 158.0, 150.73, 150.70, 147.01, 146.98, 144.0, 143.8 (br), 138.1 (br), 136.4, 135.1, 134.6, 134.5, 134.3, 132.2 (t, J_{C-P} = 6.3 Hz), 132.1, 131.94 (t, J_{C-P} = 6.2 Hz), 131.89, 131.85, 131.7, 130.64, 130.59, 130.2, 130.1, 129.01, 128.97, 128.9, 128.6, 128.5, 127.63, 127.55, 125.83, 125.78, 125.1,

123.9, 122.8, 122.5, 120.9, 120.6, 120.4, 116.9, 116.6, 116.2, 86.2, 84.8, 84.4, 83.4, 82.6, 81.7, 73.1, 71.9, 71.5, 71.0, 70.7, 70.3, 69.4, 68.2, 67.2, 65.1, 64.5, 64.1, 57.8, 35.1, 31.5 (12 signals coincident or not observed). **^{31}P{^{1}H} NMR** (202 MHz, CD_2Cl_2) δ_P 39.4. **ESI HRMS *m/z*:** calcd for $C_{364}H_{295}Co_8N_6O_{25}P_8$ ($[M + 3H]^{3+}$) 1957.4891, found 1957.5133. **GPC** (analytical, THF/pyridine 1% v/v) retention time: 35.15 min.

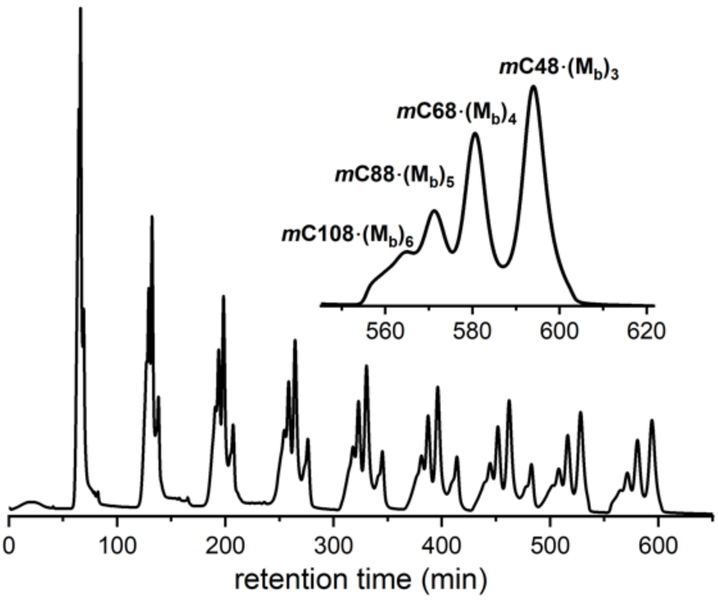

A typical recycling GPC trace of the crude reaction containing ***m*C28·(M$_b$)$_2$**, ***m*C48·(M$_b$)$_3$**, ***m*C68·(M$_b$)$_4$**, ***m*C88·(M$_b$)$_5$**, ***m*C108·(M$_b$)$_6$**, and ***m*C128·(M$_b$)$_7$** (eluting with THF at a flow rate of 3.5 mL/min, λ = 480 nm).

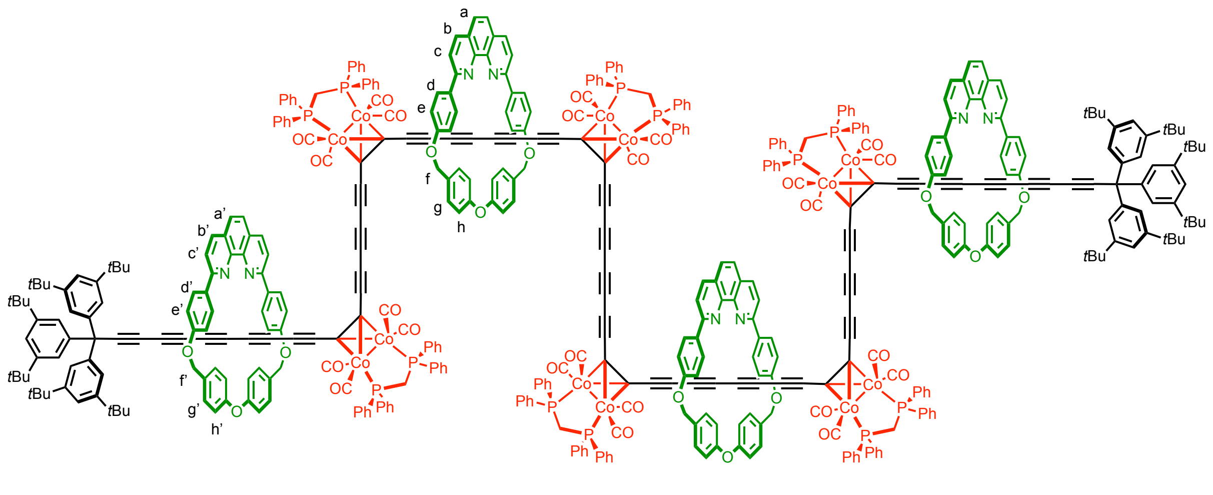

Compound *m*C68·(M$_b$)$_4$: The titled compound ***m*C68·(M$_b$)$_4$** (11 mg, 1.4 µmol, 14%) was obtained as a black solid as described in the above procedure.

IR (ATR): 2962 (w, C–H), 2928 (w, C–H), 2869 (w, C–H), 2151 (w, C≡C), 2036 (m, C=O), 2018 (s, C=O), 1994 (m, C=O) cm^{-1}. **UV-vis** ($CHCl_3$) λ_{max} (ε): 284 (386000), 318 (376000), 422 (260000), 582 (sh, 27500) nm. **^{1}H NMR** (500 MHz, CD_2Cl_2) δ_H 8.18 (d, J = 8.3 Hz, 4H; $H_{b'/b}$), 8.17 (d, J = 8.3 Hz, 4H; $H_{b/b'}$), 8.10 (d, J = 8.8 Hz, 8H; H_d), 7.99 (d, J = 8.7 Hz, 8H; $H_{d'}$), 7.90 (d, J = 8.3 Hz, 4H; $H_{c/c'}$), 7.88 (d, J = 8.4 Hz, 4H; $H_{c'/c}$), 7.70 (s, 4H; $H_{a'/a}$), 7.68 (s, 4H; $H_{a/a'}$), 7.43–7.38 (m, 16H), 7.36–7.34 (m, 8H), 7.32–7.22 (m, 98H), 7.15–7.11 (m, 36H), 7.05 (d, J = 8.8 Hz, 8H; H_e), 6.97 (d, J = 8.7 Hz, 8H; $H_{e'}$), 6.94 (d, J = 1.8 Hz, 12H; *ortho*-Tr*), 5.35 (s, 8H; H_f), 5.30 (s, 8H; $H_{f'}$), 3.53–3.27 (m, 12H; PCH_2), 1.18 (s, 108H; *t*Bu). **^{13}C{^{1}H} NMR** (151 MHz,

CD_2Cl_2) δ_C 160.0, 159.8, 159.3, 158.6, 158.5, 158.1, 158.0, 150.7, 146.99, 146.96, 144.0, 136.4, 136.3, 135.2 (br), 135.0 (br), 134.9 (br), 134.6, 134.5, 134.44, 134.40, 134.29, 134.25, 132.20, 132.16, 132.11, 132.05, 132.01, 131.97, 131.93, 131.88, 131.8, 130.7, 130.64, 130.58, 130.2, 130.1, 129.01, 128.98, 128.95, 128.9, 127.6, 127.5, 125.82, 125.78, 123.9, 122.7, 122.5, 120.9, 120.6, 120.4, 116.8, 116.6, 86.1, 84.90, 84.87, 84.8, 84.6, 84.4, 83.7, 83.3, 82.7, 82.3, 81.7, 73.2, 73.0, 71.9, 71.5, 71.3, 71.2, 71.0, 70.7, 70.3, 68.2, 67.3, 67.1, 65.1, 64.5, 64.0, 57.8, 35.1, 31.5 (14 signals coincident or not observed *and* ^{31}P–^{13}C *couplings are not resolved due to overlapping signals*). **^{31}P{^{1}H} NMR** (202 MHz, CD_2Cl_2) δ_P 39.4. **ESI HRMS *m/z*:** calcd for $C_{480}H_{366}Co_{12}N_8O_{36}P_{12}$ ($[M + 4H]^{4+}$) 1975.1510, found 1975.1534. **GPC** (analytical, THF/pyridine 1% v/v) retention time: 34.37 min.

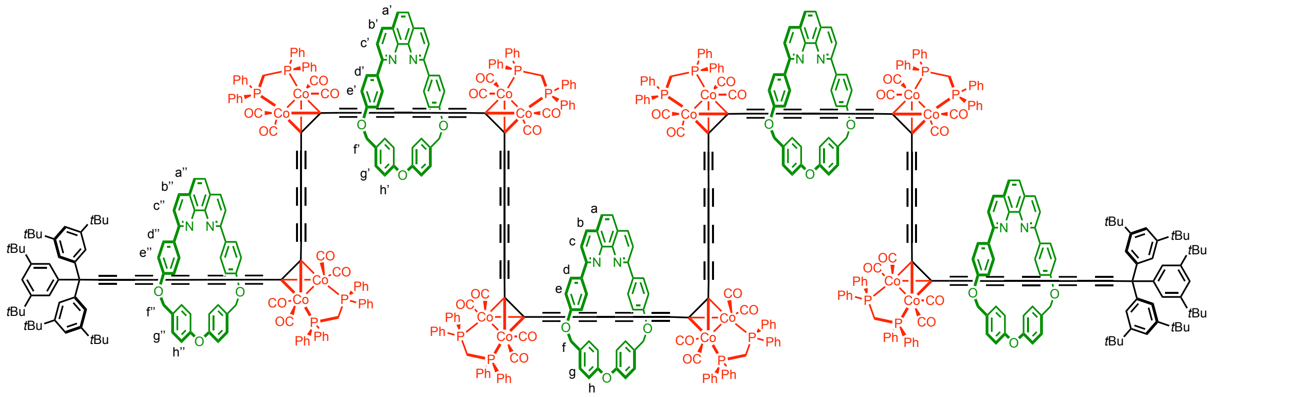

Compound *m*C88·(M$_b$)$_5$: The titled compound ***m*C88·(M$_b$)$_5$** (5 mg, 0.5 μmol, 7%) was obtained as a black solid as described in the above procedure.

^{1}H NMR (500 MHz, CD_2Cl_2) δ_H 8.18 (d, J = 8.3 Hz, 2H; H_b), 8.18 (d, J = 8.3 Hz, 4H; $H_{b'/b''}$), 8.17 (d, J = 8.3 Hz, 4H; $H_{b''/b'}$), 8.09 (d, J = 8.7 Hz, 8H; $H_{d'}$), 8.08 (d, J = 8.8 Hz, 4H; H_d), 7.98 (d, J = 8.7 Hz, 8H; $H_{d''}$), 7.91 (d, J = 8.3 Hz, 2H; H_c), 7.90 (d, J = 8.3 Hz, 4H; $H_{c'/c''}$), 7.87 (d, J = 8.3 Hz, 4H; $H_{c''/c'}$), 7.693 (s, 4H; $H_{a'/a''}$), 7.687 (s, 2H; H_a), 7.68 (s, 4H; $H_{a''/a'}$), 7.40–7.35 (m, 40H, $H_{g+g'+g''+h+h'+h''}$), 7.32–7.22 (m, 134H), 7.14–7.11 (m, 48H), 7.05 (d, J = 8.8 Hz, 12H; $H_{e+e'}$), 6.97 (d, J = 8.7 Hz, 8H; $H_{e''}$), 6.93 (d, J = 1.8 Hz, 12H; *ortho*-Tr*), 5.35 (s, 4H; H_f), 5.34 (s, 8H; $H_{f'}$), 5.30 (s, 8H; $H_{f''}$), 3.51–3.32 (m, 16H; PCH_2), 1.17 (s, 108H; *t*Bu). **^{13}C{^{1}H} NMR** (151 MHz, CD_2Cl_2) δ_C 160.0, 159.8, 158.6, 158.53, 158.51, 158.1, 158.0, 150.7, 147.00, 146.96, 144.0, 136.4, 136.3, 134.9, 134.8, 134.7, 134.60, 134.57, 134.5, 134.29, 134.27, 134.25, 132.20, 132.16, 132.10, 132.05, 132.01, 131.97, 131.93, 131.88, 131.84, 131.80, 130.7, 130.2, 130.1, 129.01, 128.98, 128.95, 128.9, 127.6, 127.5, 125.82, 125.78, 123.9, 122.7, 122.6, 122.5, 120.9, 120.6, 120.4, 116.8, 116.6, 84.9, 84.6, 84.4, 83.7, 71.0, 70.7, 70.3, 68.2, 67.3, 57.8, 35.1, 31.5 (74 signals coincident or not observed *and* ^{31}P–^{13}C *couplings are not resolved due to overlapping signals*). **^{31}P{^{1}H} NMR** (202 MHz, CD_2Cl_2) δ_P 39.4. **ESI HRMS *m/z*:** calcd for $C_{596}H_{437}Co_{16}N_{10}O_{47}P_{16}$ ($[M + 5H]^{5+}$) 1985.7480, found 1985.9485; calcd for $C_{596}H_{436}Co_{16}N_{10}O_{47}P_{16}$ ($[M + 4H]^{4+}$) 2481.9332, found 2481.8789; calcd for $C_{596}H_{435}Co_{16}N_{10}O_{47}P_{16}$ ($[M + 3H]^{3+}$) 3308.9085, found 3308.7858. **GPC** (analytical, THF/pyridine 1% v/v) retention time: 33.66 min.

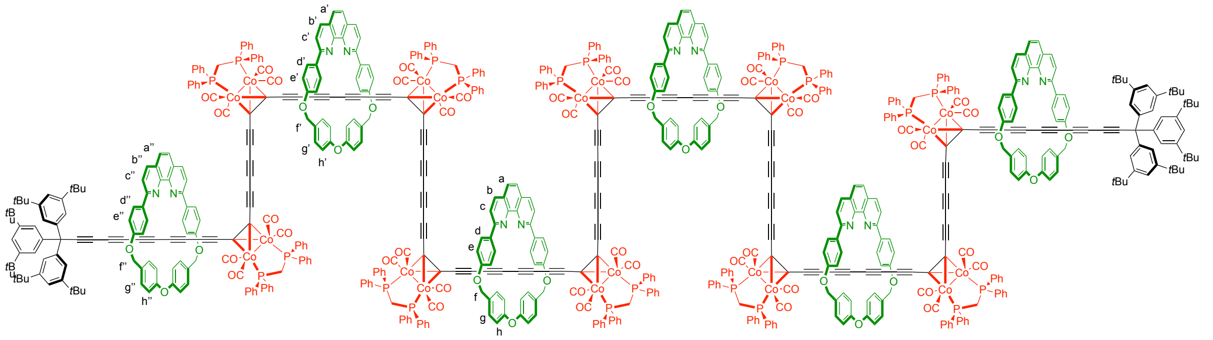

Compound *m*C108·(M$_b$)$_6$: The titled compound ***m*C108·(M$_b$)$_6$** (2.0 mg, 0.17 μmol, 3%) was obtained as a black solid as described in the above procedure. **^{1}H NMR** (600 MHz, CD_2Cl_2) δ_H 8.18 (d, J = 8.4 Hz, 4H; $H_{b/b'/b''}$), 8.18 (d, J = 8.3 Hz, 4H; $H_{b/b'/b''}$), 8.17 (d, J = 8.2 Hz, 4H; $H_{b/b'/b''}$), 8.09 (d, J = 8.8 Hz, 8H; $H_{d/d'}$), 8.09 (d, J = 8.8 Hz, 8H; $H_{d/d'}$), 7.98 (d, J = 8.6 Hz, 8H; $H_{d''}$), 7.91 (d, J = 8.4 Hz, 4H; $H_{c/c'/c''}$), 7.90 (d, J = 8.3 Hz, 4H; $H_{c/c'/c''}$), 7.87 (d, J = 8.3 Hz, 4H; $H_{c/c'/c''}$), 7.69 (s, 4H; $H_{a/a'/a''}$), 7.68 (s, 4H; $H_{a/a'/a''}$), 7.67 (s, 4H; $H_{a/a'/a''}$), 7.42–7.23 (m, 194H), 7.14–7.13 (m, 60H), 7.05–7.04 (m, 16H, $H_{e+e'}$), 6.97 (d, J = 8.7 Hz, 8H; $H_{e''}$), 6.93 (d, J = 1.8 Hz, 12H; *ortho*-Tr*), 5.35 (s, 8H; H_f), 5.34 (s, 8H; $H_{f'}$), 5.29 (s, 8H; $H_{f''}$), 3.51–3.33 (m, 20H; PCH_2), 1.17 (s, 108H; *t*Bu). **^{31}P{^{1}H} NMR** (243 MHz, CD_2Cl_2) δ_P 39.4. **ESI HRMS *m/z*:** calcd for $C_{712}H_{508}Co_{20}N_{12}O_{58}P_{20}$ ($[M + 6H]^{6+}$) 1992.9799, found 1992.9747; calcd for $C_{712}H_{507}Co_{20}N_{12}O_{58}P_{20}$ ($[M + 5H]^{5+}$) 2391.3744, found 2391.3302. **GPC** (analytical, THF/pyridine 1% v/v) retention time: 33.18 min.

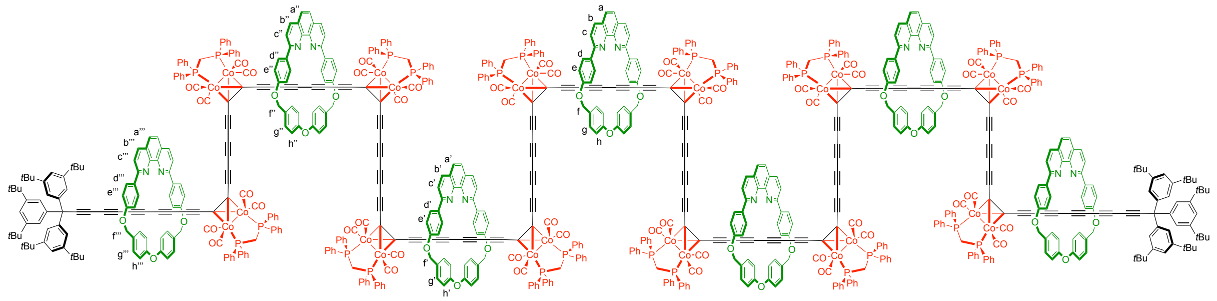

Compound *m*C128·(M$_b$)$_7$: The titled compound ***m*C128·(M$_b$)$_7$** (0.8 mg, 0.06 μmol, 1%) was obtained as a black solid as described in the above procedure. **^{1}H NMR** (600 MHz, CD_2Cl_2) δ_H 8.18–8.16 (m, 14H; $H_{b+b'+b''+b'''}$), 8.10–8.08 (m, 20H; $H_{d+d'+d''}$), 7.98 (d, J = 8.5 Hz, 8H; $H_{d'''}$), 7.91–7.86 (m, 14H; $H_{c+c'+c''+c'''}$), 7.69–7.67 (m, 14H; $H_{a+a'+a''+a'''}$), 7.41–7.22 (m, 230H), 7.13 (m, 72H), 7.05–7.04 (m, 20H, $H_{e+e'+e''}$), 6.96 (d, J = 8.6 Hz, 8H; $H_{e'''}$), 6.93 (d, J = 1.8 Hz, 12H; *ortho*-Tr*), 3.49–3.34 (m, 24H; PCH_2), 1.17 (s, 108H; *t*Bu) (CH_2 from M$_b$ overlapped with the solvent signal). **^{31}P{^{1}H} NMR** (202 MHz, CD_2Cl_2) δ_P 39.4. **ESI HRMS *m/z*:** calcd for $C_{828}H_{578}Co_{24}N_{14}O_{69}P_{24}$ ($[M + 6H]^{6+}$) 2330.8347, found 2330.7774; calcd for $C_{828}H_{577}Co_{24}N_{14}O_{69}P_{24}$ ($[M + 5H]^{5+}$) 2796.8002, found 2796.6333. **GPC** (analytical, THF/pyridine 1% v/v) retention time: 32.88 min.

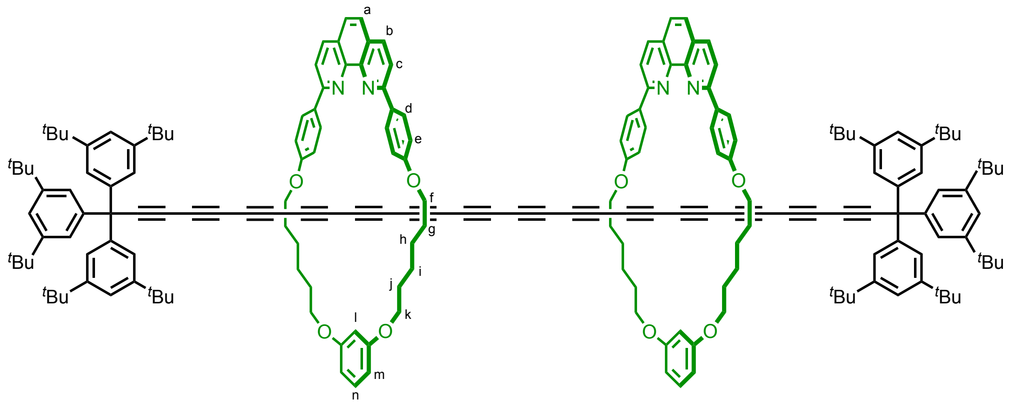

Compound C28·(M$_a$)$_2$: A solution of magnesium monoperoxyphthalate hexahydrate (93 mg, 80 wt%, 20 eq., 0.15 mmol) in MeOH (5.0 mL) was added dropwise using a syringe pump over a period of 2 h to a solution of cobalt-masked [3]rotaxane ***m*C28·(M$_a$)$_2$** (30 mg, 1.0 eq., 7.5 µmol) in CH_2Cl_2 (15 mL). The reaction was monitored using TLC and, upon reaching completion, was quenched using a sat aqueous $NaHCO_3$ solution (20 mL). The organic layer was washed with water and brine, then dried over Na_2SO_4 and the solvent was removed under reduced pressure. The crude mixture was purified by silica gel column chromatography (petroleum ether/EtOAc, gradient elution from 0 to 75%) to yield 14-yne [2]rotaxane **C28·(M$_a$)$_2$** (12 mg, 4.4 µmol, 59%) as a yellow solid.

IR (ATR) (only selected signals) 3006 (C–H), 2963 (C–H), 2200 (C≡C), 2130 (C≡C), 2013 (C≡C) cm^{-1}. **UV-vis** ($CHCl_3$) λ_{max} (ε) 432 (346000), 401 (323000), 375 (205000), 355 (136000), 336 (99100), 289 (126000) nm. **^{1}H NMR** (600 MHz, CD_2Cl_2) δ_H 8.38 (d, J = 8.8 Hz, 8H, H$_d$), 8.20 (d, J = 8.4 Hz, 4H, H$_b$), 8.01 (d, J = 8.3 Hz, 4H, H$_c$), 7.70 (s, 4H, H$_a$), 7.33 (t, J = 1.8 Hz, 6H, Tr* Ar-H), 7.09 (t, J = 8.8 Hz, 2H, H$_n$), 7.04 (d, J = 8.8 Hz, 8H, H$_e$), 6.94 (d, J = 1.8 Hz, 12H, Tr* Ar-H), 6.45 – 6.41 (m, 4H, H$_{l,m}$), 4.05 – 3.98 (m, 8H, H$_f$), 3.93 – 3.86 (m, 8H, H$_k$), 1.88 – 1.82 (m, 8H, H$_g$), 1.80 – 1.73 (m, 8H, H$_j$), 1.55 – 1.50 (m, 16H, H$_{h,i}$), 1.22 (s, 108H, ^{t}Bu). **^{13}C{^{1}H} NMR** (151 MHz, CD_2Cl_2) δ_C 160.87, 160.81, 156.03 (C$_c$-C-N), 150.87, 146.41 (C$_a$-C-C$_b$), 143.47, 136.81 (C$_b$), 132.16 (N-C-C-C$_d$), 130.02 (C$_k$), 129.13 (C$_d$), 127.78 (N-C-C-N), 125.82 (C$_a$), 123.93, 121.13, 119.10 (C$_c$), 115.05 (C$_e$), 107.38 (C$_j$), 100.81 (C$_l$), 87.45, 69.17, 68.41 (C$_{f/k}$), 68.08 (C$_{k/f}$), 64.55, 64.27, 64.17, 64.06, 63.83, 63.60, 63.38, 63.17, 62.91, 62.74, 62.54, 62.25, 57.82 (CAr$_3$), 35.15 ($\underline{C}(CH_3)_3$), 31.46 ($C(\underline{C}H_3)_3$), 29.95 (C$_{j/g}$), 29.52 (C$_{g/j}$), 26.34 (C$_{h/i}$), 26.31 (C$_{i/h}$). **ESI HRMS** m/z = 2772.6213 [M+H]$^+$ ($C_{198}H_{211}N_4O_8^+$ requires 2772.6221). **Raman** (CH_2Cl_2, only most intense signal) 1940 (C≡C) cm^{-1}.

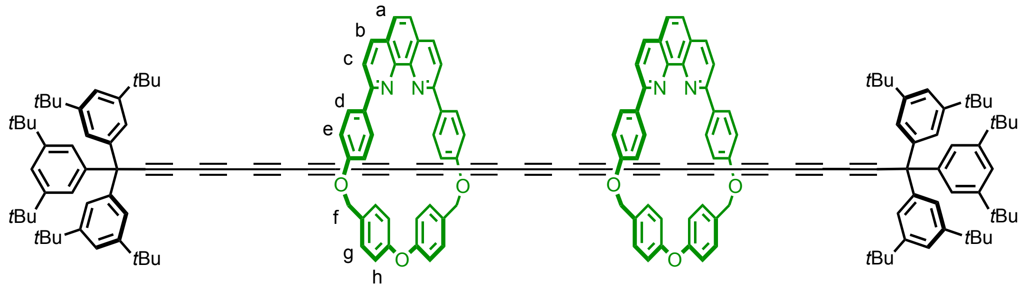

Compound C28·(M$_b$)$_2$: **Method 1**. To compound ***m*C28·(M$_b$)$_2$** (28 mg, 0.0073 mmol) in CH_2Cl_2 (30 mL) was added *m*CPBA (18 mg, 77 wt%, 0.080 mmol), and the solution was stirred at 20 °C for 5 min. Saturated aqueous $NaHCO_3$ (5 mL) was added, and the solution was stirred for 5 min. H_2O (20 mL) was added, the layers were separated, and the aqueous phase was extracted with CH_2Cl_2 (2 × 10 mL). The organic phases were combined, washed with brine (20 mL), dried ($MgSO_4$), and filtered. Solvent removal and purification by

column chromatography (silica gel, ethyl acetate/petroleum ether 1:10 to 1:5) afforded compound **C28·(M$_b$)$_2$** (14 mg, 5.4 µmol, 74%) as a yellow-orange solid.

Method 2. To compound ***m*C28·(M$_b$)$_2$** (15 mg, 0.0039 mmol) in CH_2Cl_2 (6 mL) was added a solution of magnesium monoperoxyphthalate hexahydrate (97 mg, 80 wt%, 0.16 mmol) in MeOH (3 mL) via syringe pump over 1.5 h, while the solution was stirred at 20 °C. Saturated aqueous $NaHCO_3$ (2 mL) was added, and the solution was stirred for 5 min. H_2O (10 mL) and CH_2Cl_2 (10 mL) were added, the layers were separated, and the aqueous phase was extracted with CH_2Cl_2 (2 × 10 mL). The organic phases were combined, washed with brine (20 mL), dried ($MgSO_4$), and filtered. Solvent removal and purification by column chromatography (silica gel, ethyl acetate/petroleum ether 1:10 to 1:5) afforded compound **C28·(M$_b$)$_2$** (6.5 mg, 2.5 µmol, 64%) as a yellow-orange solid.

IR (ATR): 2963 (s, C–H), 2927 (m, C–H), 2867 (m, C–H), 2201 (w, C≡C), 2131 (w, C≡C), 2081 (w, C≡C), 2015 (w, C≡C), 1937 (w, C≡C) cm^{-1}. **UV-vis** ($CHCl_3$) λ_{max} (ε): 275 (sh, 103000), 285 (118000), 297 (90400), 308 (78700), 320 (77100), 337 (83100), 354 (123000), 376 (224000), 401 (351000), 431 (386000) nm. **^{1}H NMR** (500 MHz, CD_2Cl_2) δ_H 8.18 (d, *J* = 8.3 Hz, 4H; H$_b$), 7.84 (d, *J* = 8.3 Hz, 4H; H$_c$), 7.78 (d, *J* = 8.7 Hz, 8H; H$_d$), 7.71 (s, 4H; H$_a$), 7.29 (t, *J* = 1.8 Hz, 6H; *para*-Tr*), 7.03 (d, *J* = 8.4 Hz, 8H; H$_g$), 6.91 (d, *J* = 1.8 Hz, 12H; *ortho*-Tr*), 6.89 (d, *J* = 8.4 Hz, 8H; H$_h$), 6.68 (d, *J* = 8.7 Hz, 8H; H$_e$), 4.98, 4.89 (ABq, J_{AB} = 15.0 Hz, 8H; H$_f$), 1.18 (s, 108H; *t*Bu). **^{13}C{^{1}H} NMR** (126 MHz, CD_2Cl_2) δ_C 159.6, 158.4, 157.8, 150.9 (Tr*), 146.8, 143.5 (Tr*), 136.6, 134.5, 134.1, 129.9, 127.8, 127.4, 126.0, 123.9 (Tr*), 122.3, 121.1 (Tr*), 120.6, 116.5, 87.1, 70.5, 69.3, 64.1, 63.93, 63.85, 63.80, 63.78, 63.7, 63.58, 63.4, 63.2, 62.9, 62.8, 62.6, 57.8, 35.2, 31.5. **ESI HRMS *m/z*:** calcd for $C_{190}H_{179}N_4O_6$ ([M + H]$^+$) 2614.3885, found 2614.3861.

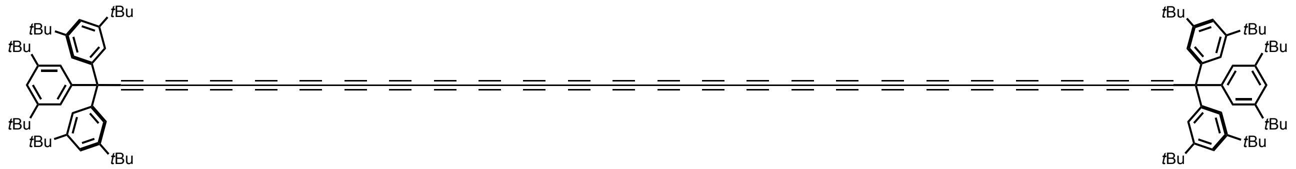

Compound C48: A solution of *m*CPBA (16 mg, 77 wt%, 24 eq., 70 µmol) in CD_2Cl_2 (noted CH_2Cl_2 also works, 1 mL) was added to a solution of octacobalt thread **13** (12 mg, 1.0 eq, 3.0 µmol) in CD_2Cl_2 (noted CH_2Cl_2 also works, 2 mL). Solvent removal and purification by column chromatography (silica gel, CH_2Cl_2/pentane 1:4) afforded compound **C48** (2.9 mg, 1.7 µmol, 56%) as an orange-red solid.

IR (ATR) (only selected signals) 2962 (m, C–H), 2925 (m, C–H), 2854 (w, C–H), 2201 (vw, C≡C), 2096 (vw, C≡C), 2025 (vw, C≡C), 1942 (vw, C≡C), 1933 (vw, C≡C) cm^{-1}. **UV-vis** (CH_2Cl_2) λ_{max}: 471, 438, 410, 387, 371, 356, 339, 326, 279 nm. **^{1}H NMR** (500 MHz, CD_2Cl_2) δ_H 7.27 (s, 6H, Tr* Ar-H), 6.94 (s, 12H, Tr* Ar-H), 1.18 (s, 108H, ^{t}Bu). **MALDI HRMS** (DCTB matrix) 1735.371 [M]$^-$ ($C_{134}H_{126}$ requires 1734.986). **Raman** (CH_2Cl_2, only most intense signal) 1913 (C≡C) cm^{-1}.

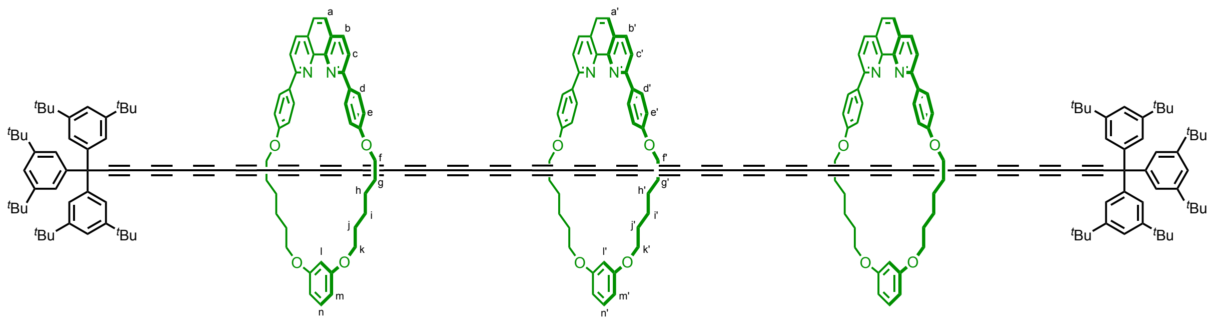

Compound C48·(M$_a$)$_3$: To a solution of cobalt-masked [4]rotaxane ***m*C48·(M$_a$)$_3$** (8.8 mg, 1.0 eq, 1.4 µmol) in CH_2Cl_2 (5.0 mL) was added dropwise a solution of magnesium monoperoxyphthalate hexahydrate (89 mg, 80 wt%, 100 eq., 144 µmol) in MeOH (4.0 mL) via syringe pump over a period of 2 h. The reaction was monitored using TLC and, once complete, was quenched using a sat. aqueous $NaHCO_3$ solution (~10 mL). The organic layer was washed with water and brine, then dried over Na_2SO_4 and the solvent removed under reduced pressure until a minimal amount (~0.5 mL) of solvent remained. The crude mixture was purified using silica gel column chromatography (petroleum ether/EtOAc, gradient elution from 0 to 75%) to yield 24-yne [4]rotaxane **C48·(M$_a$)$_3$** (1.0 mg, 0.27 µmol, 19%) as a red-orange solid after a quick removal of solvents. *(Note: the mass of the solid was determined as ca. 1.0 mg, 19% yield, but the solid has decomposed to some extent during drying.)*

UV-vis (CH_2Cl_2) λ_{max}: 478, 443, 415, 392, 372, 360, 344, 329, 289 nm. **^{1}H NMR** (700 MHz, CD_2Cl_2) δ_H 8.45 – 8.24 (br m, 12H, $H_{d,d'}$), 8.20 – 8.10 (br m, 6H, $H_{b,b'}$), 8.04 – 7.91 (br m, 6H, $H_{c,c'}$), 7.69 – 7.60 (br m, 6H, $H_{a,a'}$), 7.30 (t, J = 1.7 Hz, 6H, Tr* Ar-H), 7.10 – 7.05 (m, 3H, $H_{n,n'}$), 7.04 – 7.00 (m, 8H, H_e), 6.96 – 6.93 (m, 4H, $H_{e'}$), 6.91 (d, J = 1.8 Hz, 12H, Tr* Ar-H), 6.43 – 6.39 (m, 6H, $H_{n,n'}$), 6.36 (dd, J = 8.1, 2.4 Hz, 2H, H_l), 6.33 (br s, 1H, $H_{l'}$), 4.03 – 3.99 (m, 8H, H_f), 3.91 – 3.87 (m, 12H, $H_{k,f'}$), 3.79 (t, J = 6.4 Hz, 4H, $H_{k'}$), 1.86 – 1.82 (m, 8H, H_g), 1.78 – 1.74 (m, 12H, $H_{j,g'}$), 1.69 – 1.67 (m, 4H, $H_{j'}$), 1.57 (br s, 16H, $H_{h/h'/i/i'}$), 1.43 (br s, 8H, $H_{h/h'/i/i'}$), 1.18 (s, 108H, ^{t}Bu). **MALDI HRMS** (DCTB matrix) m/z = 1825.9779 $[M+2H]^{2+}$ ($C_{260}H_{254}N_6O_{12}^{2+}$ requires 1825.9719). **Raman** (CH_2Cl_2, only most intense signal) 1910 (C≡C) cm^{-1}.

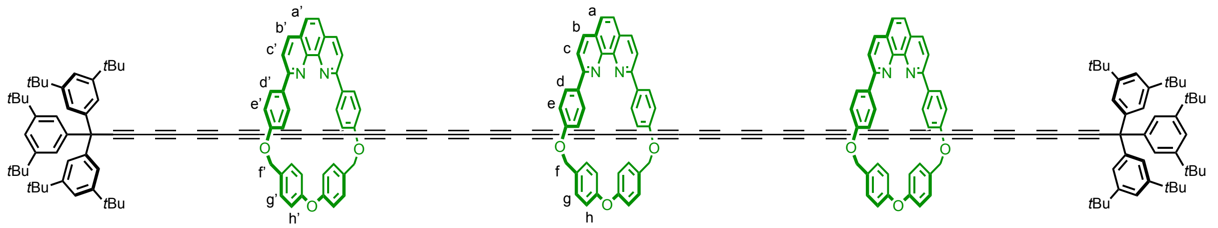

Compound C48·(M$_b$)$_3$: **Method 1**. To compound ***m*C48·(M$_b$)$_3$** (12 mg, 0.0020 mmol) in CH_2Cl_2 (12 mL) was added *m*CPBA (10 mg, 77 wt%, 0.045 mmol), and the solution was stirred at 20 °C for 10 min. Saturated aqueous $NaHCO_3$ (3 mL) was added, and the solution was stirred for 5 min. H_2O (20 mL) was added, the layers were separated, and the aqueous phase was extracted with CH_2Cl_2 (2 × 10 mL). The organic phases were combined, washed with brine (20 mL), dried ($MgSO_4$), and filtered. Solvent removal and purification by column chromatography (silica gel, ethyl acetate/petroleum ether 1:10 to 1:5) afforded compound **C48·(M$_b$)$_3$** (3.0 mg, 0.88 µmol, 43%) as an orange-red solid.

Method 2. To compound ***m*C48·(M$_b$)$_3$** (7.5 mg, 0.0013 mmol) in CH_2Cl_2 (4 mL) was added a solution of magnesium monoperoxyphthalate hexahydrate (47 mg, 80wt%, 0.076 mmol) in MeOH (2 mL) via syringe pump over 1.5 h, while the solution was stirred at 20 °C. Saturated aqueous $NaHCO_3$ (2 mL) was added, and

the solution was stirred for 5 min. H_2O (10 mL) and CH_2Cl_2 (10 mL) were added, the layers were separated, and the aqueous phase was extracted with CH_2Cl_2 (2 × 10 mL). The organic phases were combined, washed with brine (20 mL), dried ($MgSO_4$), and filtered. Solvent removal and purification by column chromatography (silica gel, ethyl acetate/petroleum ether 1:10 to 1:5) afforded compound **C48·(M$_b$)$_3$** (1.1 mg, 0.32 µmol, 25%) as an orange-red solid.

IR (ATR): 2958 (m, C–H), 2925 (m, C–H), 2854 (m, C–H), 2200 (w, C≡C), 2099 (w, C≡C), 2026 (w, C≡C), 1982 (w, C≡C), 1937 (w, C≡C), 1898 (w, C≡C), 800 (s) cm^{-1}. **UV-vis** ($CHCl_3$) λ_{max} (ε): 283 (196000), 332 (188000), 344 (183000), 361 (170000), 394 (251000), 418 (438000), 447 (709000), 482 (706000) nm. **^{1}H NMR** (500 MHz, CD_2Cl_2) δ_H 8.23 (d, J = 8.3 Hz, 2H; H$_b$), 8.15 (d, J = 8.3 Hz, 4H; H$_{b'}$), 7.90 (d, J = 8.3 Hz, 2H; H$_c$), 7.82 (d, J = 8.7 Hz, 4H; H$_d$), 7.81 (d, J = 8.3 Hz, 4H; H$_{c'}$), 7.79 (d, J = 8.7 Hz, 8H; H$_{d'}$), 7.74 (s, 2H; H$_a$), 7.67 (s, 4H; H$_{a'}$), 7.30 (t, J = 1.8 Hz, 6H; *para*-Tr*), 7.05 (d, J = 8.4 Hz, 8H; H$_{g'}$), 6.92 (d, J = 1.8 Hz, 12H; *ortho*-Tr*), 6.87 (d, J = 8.4 Hz, 8H; H$_{h'}$), 6.82 (d, J = 8.3 Hz, 4H; H$_g$), 6.67 (d, J = 8.7 Hz, 8H; H$_{e'}$), 6.54 (d, J = 8.3 Hz, 4H; H$_h$), 6.53 (d, J = 8.7 Hz, 4H; H$_e$), 4.93, 4.89 (ABq, J_{AB} = 15.0 Hz, 8H; H$_{f'}$), 4.54 (s, 4H; H$_f$), 1.19 (s, 108H; *t*Bu). **^{13}C{^{1}H} NMR** (151 MHz, CD_2Cl_2) δ_C 159.8, 159.6, 158.4, 158.3, 157.7, 157.5, 150.9 (Tr*), 146.9, 146.8, 143.5 (Tr*), 136.8, 136.7, 134.64, 134.60, 134.1, 133.8, 129.8, 129.7, 127.9, 127.8, 127.4, 127.1, 126.1, 126.0, 124.0 (Tr*), 122.5, 122.4, 121.1 (Tr*), 120.6, 120.4, 116.7, 116.6, 87.4, 70.5, 69.2, 64.3, 64.03, 63.95, 63.89, 63.79, 63.75, 63.69, 63.60, 63.54, 63.46, 63.33, 63.14, 62.89, 62.83, 62.60, 62.40, 57.8, 35.2, 31.5 (6 sp^1-carbon signals coincident or not observed). **MALDI HRMS (DCTB) *m/z*:** calcd for $C_{248}H_{206}N_6O_9$ ($[M + 2H]^{2+}$) 1706.7952, found 1706.7972.

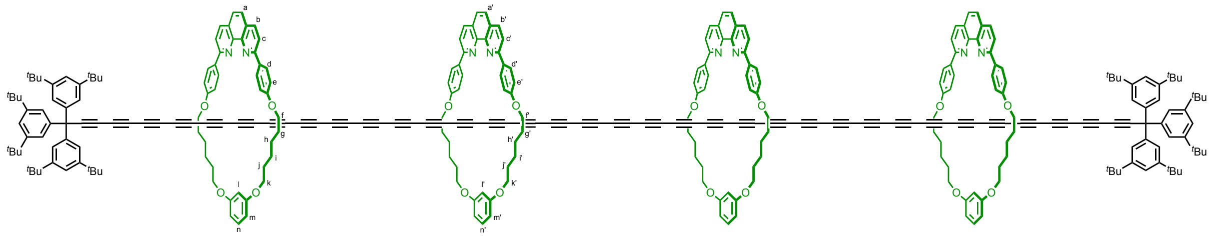

Compound C68·(M$_a$)$_4$: To a solution of cobalt-masked [5]rotaxane ***m*C68·(M$_a$)$_4$** (4.0 mg, 1.0 eq, 0.49 µmol) in CH_2Cl_2 (6.0 mL) was added dropwise a solution of magnesium 2-carboperoxybenzoate hexahydrate (36 mg, 80 wt%, 120 eq., 58 µmol) in MeOH (5.0 mL) via syringe pump over a period of 2 h. The reaction was quenched using a sat. aqueous $NaHCO_3$ solution (~10 mL). The organic layer was washed with water and brine, then dried over Na_2SO_4 and the solvent removed under reduced pressure until a minimal amount (~0.5 mL) of solvent remained. The crude mixture was passed through a quick silica gel plug (EtOAc) to yield 34-yne [5]rotaxane **C68·(M$_a$)$_4$** as an orange-red solution. *(Note: The compound shows significant decomposition upon complete solvent removal.)*

UV-vis (CH_2Cl_2) λ_{max}: 287, 329, 347, 369, 402, 425, 456, 492 nm. **MALDI HRMS** (DCTB) 4529.217 $[M+H]^+$ ($C_{322}H_{294}N_8O_{16}$ requires 4529.251). **Raman** (CH_2Cl_2, only most intense signal) 1904 (C≡C) cm^{-1}.

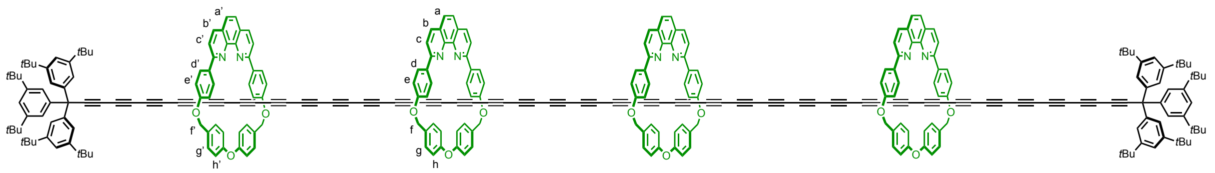

Compound C68·(M$_b$)$_4$: To compound ***m*C68·(M$_b$)$_4$** (2.8 mg, 0.355 µmol) in CH_2Cl_2 (2.5 mL) was added a solution of magnesium monoperoxyphthalate (26.3 mg, 80% w/w, 42.5 µmol) in MeOH (1 mL) via syringe pump over 1.5 h, while the solution was stirred at 20 °C. Saturated aqueous $NaHCO_3$ (2 mL) was added, and the solution was stirred for 5 min. H_2O (10 mL) and CH_2Cl_2 (10 mL) were added, the layers were separated, and the aqueous phase was extracted with CH_2Cl_2 (2 × 10 mL). The organic phases were combined, washed with brine (20 mL), dried ($MgSO_4$), and filtered. Solvent removal, purification by a short column chromatography (silica gel, ethyl acetate/petroleum ether1:5, then ethyl acetate/CH_2Cl_2/petroleum ether 1:2:2) and washing with ethanol (3 × 5 mL) afforded compound **C68·(M$_b$)$_4$** (0.35 mg, 0.083 µmol, 23%) as an orange-red solid.

UV-vis (CH_2Cl_2) λ_{max}: 496, 460, 429, 403 (sh), 377 (sh), 352, 321, 282 nm. **^{1}H NMR** (600 MHz, CD_2Cl_2) δ_H 8.21 (d, J = 8.2 Hz, 4H; H_b), 8.15 (d, J = 8.2 Hz, 4H; $H_{b'}$), 7.89 (d, J = 8.2 Hz, 4H; H_c), 7.84 (d, J = 8.4 Hz, 8H; H_d), 7.81 (d, J = 8.2 Hz, 4H; $H_{c'}$), 7.79 (d, J = 8.4 Hz, 8H; $H_{d'}$), 7.71 (s, 4H; H_a), 7.67 (s, 4H; $H_{a'}$), 7.30 (t, J = 1.8 Hz, 6H; *para*-Tr*), 7.06 (d, J = 8.2 Hz, 8H; $H_{g'}$), 6.92 (d, J = 1.8 Hz, 12H; *ortho*-Tr*), 6.87 (d, J = 8.2 Hz, 8H; $H_{h'}$), 6.84 (d, J = 8.1 Hz, 8H; H_g), 6.67 (d, J = 8.4 Hz, 8H; $H_{e'}$), 6.52 (d, J = 8.4 Hz, 4H; H_e), 6.50 (d, J = 8.1 Hz, 4H; H_h), 4.93, 4.90 (ABq, J_{AB} = 14.8 Hz, 8H; $H_{f'}$), 4.51, 4.46 (ABq, J_{AB} = 15.1 Hz, 8H; H_f), 1.19 (s, 108H; *t*Bu). **ESI HRMS *m/z*:** calcd for $C_{306}H_{234}N_8O_{12}$ ($[M + 4H]^{4+}$) 1053.7006, found 1053.7248; calcd for $C_{306}H_{233}N_8O_{12}$ ($[M + 3H]^{3+}$) 1404.5984, found 1404.5970. **MALDI HRMS (DCTB) *m/z*:** calcd for $C_{306}H_{230}N_8O_{12}$ ($[M]^+$) 4210.7721, found 4210.904.

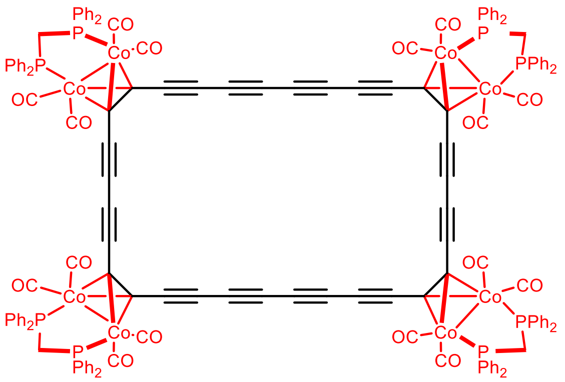

***mc*C32**: Dry $CHCl_3$ (100 mL) and diisopropylamine (130 mg, 180 µL, 20 eq., 1.3 mmol) were added to deprotected tetracobalt tetrayne thread **3** (90 mg, 1.0 eq., 63 µmol), bis(triphenylphosphine)palladium(II) dichloride (30 mg, 0.67 eq., 42 µmol), copper(I) iodide (40 mg, 3.3 eq., 21 µmol) and benzoquinone (93 mg, 14 eq., 0.86 mmol). A drying tube was fitted, and reaction stirred at 20 °C in air for 24 h. The mixture was passed through a short silica plug ($CHCl_3$) before removing the solvent under reduced pressure. The crude material was purified by recycling GPC, then recrystallized from $CHCl_3$/pentane to give the cyclic dimer ***mc*C32** (30 mg, 11 µmol, 35%) as a dark red crystalline solid.

IR (ATR) (only selected signals) 2923 (C–H), 2852 (C–H), 2148 (w, C≡C), 2035 (s, C=O), 2015 (s, C=O), 1990 (s, C=O) cm^{-1}. **UV-vis** (CH_2Cl_2) λ_{max} (ε) 557 (20900), 384 (180000), 326 (175000), 274 (181000) nm. **^{1}H NMR** (600 MHz, CD_2Cl_2) δ_H 7.59 – 7.36 (br m, 32H, Ar-H), 7.36 – 7.11 (br m, 48H, Ar-H), 3.72 – 3.25 (br m, 8H, P-CH$_2$). **^{13}C{^{1}H} NMR** (151 MHz, CD_2Cl_2) δ_C 202.67 (C≡O), 135.09 (br, Ar-C), 132.21, 130.63 (Ar-C), 128.97 (Ar-C), 88.34, 84.55 (br) 84.02, 82.62 (br), 70.58, 66.92, 38.52 (br, P-CH$_2$) *(2 carbons not distinguished and ^{31}P–^{13}C couplings are not resolved due to overlapping signals)*. **^{31}P{^{1}H} NMR** (243 MHz, CD_2Cl_2) δ_P 40.25. **GPC** (analytical, THF/pyridine 1% v/v) retention time = 43.02 min.

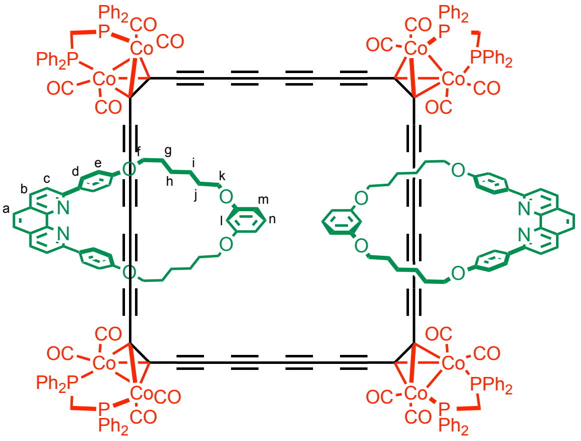

[3]Catenane *mc*C40·(M$_a$)$_2$: Dry $CHCl_3$ (40 mL) and diisopropylamine (29 mg, 40 µL, 40 eq., 0.28 mmol) was added to bis(triphenylphosphine)palladium(II) dichloride (6.6 mg, 1.3 eq., 9.5 µmol), copper(I) iodide (9.0 mg, 6.7 eq., 47 µmol) and benzoquinone (12 mg, 15 eq., 0.11 mmol). The mixture was stirred in air before addition of extended tetracobalt [2]rotaxane **6·(M$_a$)$_2$** (30 mg, 2.0 eq., 14 µmol) in dry $CHCl_3$ (10 mL) over 1 h via syringe pump. After complete addition, the reaction was stirred at 20 °C in air for a further 1 h. EDTA/NH_3 solution (25 mL) was then added and the mixture stirred for 1 h. The organic layer was extracted, washed with water (20 mL) The crude material was purified by recycling GPC (THF) to yield the octacobalt [3]catenane ***mc*C40·(M$_a$)$_2$** (6.0 mg, 1.4 µmol, 20% with respect to **6·(M$_a$)$_2$**) and hexadecacobalt [5]catenane ***mc*C80·(M$_a$)$_4$** (3.9 mg, 0.46 µmol, 13% with respect to **6·(M$_a$)$_2$**) as dark-brown solids.

IR (ATR) (only selected signals) 2935 (C–H), 2145 (C≡C), 2019 (s, C=O), 1995 (s, C=O) cm^{-1}. **UV-vis** ($CHCl_3$) λ_{max} (ε) 569 (24000), 406 (230000), 345 (246000), 327 (252000), 292 (291000) nm. **^{1}H NMR** (600 MHz, CD_2Cl_2) δ_H 8.54 (d, J = 8.3 Hz, 8H, H$_d$), 8.23 (d, J = 8.4 Hz, 4H, H$_b$), 8.08 (d, J = 8.6 Hz, 4H, H$_c$), 7.72 (s, 4H, H$_a$), 7.31 – 7.27 (m, 26H, H$_e$ & Ar-H), 7.27 – 7.20 (m, 38H, Ar-H), 7.20 – 7.16 (m, 8H, Ar-H), 7.16 – 7.07 (m, 18H, H$_n$ & Ar-H), 6.75 (s, 2H, H$_l$), 6.52 (dd, J = 8.2, 2.3 Hz, 4H, H$_m$), 4.26 (t, J = 7.3 Hz, 8H, H$_{f/k}$), 4.14 (d, J = 6.7 Hz, 8H, H$_{k/f}$), 3.55 – 3.24 (m, 8H, P-CH$_2$), 1.99 (t, J = 6.8 Hz, 8H, H$_{g/j}$), 1.93 (t, J = 6.5 Hz, 8H, H$_{j/g}$), 1.72 (br s, 16H, H$_{h,i}$). **^{13}C{^{1}H} NMR** (151 MHz, CD_2Cl_2) δ_C 202.14 (C≡O, br), 161.28 (O-C-C$_e$), 161.16, 156.31 (N-C-C-N), 146.51 (C$_a$-C-C$_b$), 136.82 (C$_b$), 134.84 (t, J_{C-P} = 21.7 Hz, Ar-C), 134.35 (t, J_{C-P} = 21.5 Hz, Ar-C), 132.03 (d, J_{C-P} = 6.5 Hz, Ar-C), 130.78 (Ar-C), 130.64 (C$_n$), 129.96 (Ar-C), 129.28 (C$_d$), 128.96 (t, J_{C-P} = 4.9 Hz, Ar-C), 127.78, 125.81 (C$_a$), 119.15 (C$_c$), 115.44 (C$_e$), 107.95 (C$_m$), 100.33 (C$_l$), 84.08, 83.71, 83.38, 83.21, 71.36, 71.11, 68.79 (C$_{k/f}$), 68.30 (C$_{f/k}$), 67.60, 66.97, 38.98 (t, J_{C-P} = 21.5 Hz, P-CH$_2$), 30.11 (C$_{j/g}$), 29.70 (C$_{g/j}$), 26.38 (C$_{h/i}$), 26.37 (C$_{i/h}$). **^{31}P{^{1}H} NMR** (243 MHz, CD_2Cl_2) δ_P 40.28. **ESI HRMS** m/z = 2107.2534 $[M+2H]^{2+}$ ($C_{240}H_{174}Co_8N_4O_{24}P_8^{2+}$ requires 2107.2532). **GPC** (analytical, THF/pyridine 1% v/v) retention time: 36.8 min.

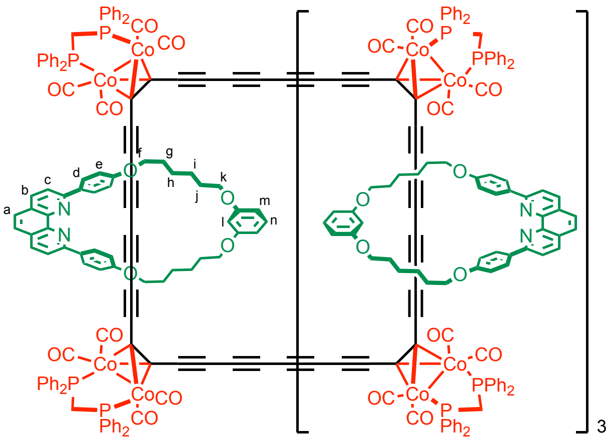

[5]Catenane *mc*C80·(M$_a$)$_4$: The titled compound ***mc*C80·(M$_a$)$_4$** (3.9 mg, 0.46 µmol, 13%) was obtained as a dark-brown solid as described in the above procedure.

IR (ATR) (only selected signals) 2961 (C–H), 2861 (C–H), 2146 (C≡C), 2018 (s, C=O), 1995 (s, C=O) cm^{-1}. **UV-vis** ($CHCl_3$) λ_{max} (ε) 592 (35000), 444 (333000), 417 (353000), 346 (379000), 328 (392000), 291 (435000) nm. **^{1}H NMR** (600 MHz, CD_2Cl_2) δ_H 8.51 (d, J = 8.3 Hz, 16H, H$_d$), 8.18 (d, J = 8.2 Hz, 8H, H$_b$), 8.04 (d, J = 8.3 Hz, 8H, H$_c$), 7.67 (s, 8H, H$_a$), 7.31 – 7.17 (m, 140H, H$_e$ & Ar-H), 7.17 – 7.10 (m, 36H, Ar-H), 6.75

(s, 4H, H_l), 6.51 (d, *J* = 8.1 Hz, 8H, H_m), 4.27 (t, *J* = 7.3 Hz, 16H, $H_{f/k}$), 4.15 (t, *J* = 6.7 Hz, 16H, $H_{k/f}$), 3.70 – 3.08 (m, 16H, P-C$\underline{H}_2$), 2.06 – 1.89 (m, 32H, $H_{j,g}$), 1.81 – 1.67 (m, 32H, $H_{h,i}$). **^{13}C{^{1}H} NMR** (151 MHz, CD_2Cl_2) δ_C 202.09 ($\underline{C}$≡O, br), 161.26 (O-$\underline{C}$-C_e), 161.16, 156.31 (N-$\underline{C}$-$\underline{C}$-N), 146.50 (C_a-$\underline{C}$-C_b), 136.81 (C_b), 135.05 – 134.02 (m, Ar-$\underline{C}$), 132.03 (d, $J_{C\text{-}P}$ = 7.3 Hz, Ar-$\underline{C}$), 130.77 (Ar-$\underline{C}$), 130.66 (C_n), 129.93 (Ar-$\underline{C}$), 129.29 (C_d), 128.99 (t, $J_{C\text{-}P}$ = 5.2 Hz, Ar-$\underline{C}$), 127.74, 125.79 (C_a), 119.17 (C_c), 115.41 (C_e), 108.00 (C_m), 100.23 (C_l), 83.78 (br), 83.32, 77.55, 71.76, 71.45, 68.77 ($C_{k/f}$), 68.32 ($C_{f/k}$), 67.77, 67.19, 38.75 (br, P-$\underline{C}H_2$), 30.14 ($C_{j/g}$), 29.73 ($C_{g/j}$), 26.40 ($C_{i/h}$), 26.36 ($C_{h/i}$). **^{31}P{^{1}H} NMR** (243 MHz, CD_2Cl_2) δ_P 39.84. **ESI HRMS** *m/z* = 2107.2834 $[M+4H]^{4+}$ ($C_{480}H_{348}Co_{16}N_8O_{48}P_{16}{}^{4+}$ requires 2107.2532). **GPC** (analytical, THF/pyridine 1% v/v) retention time: 35.9 min.

Section 3: GPC Trace

(a)

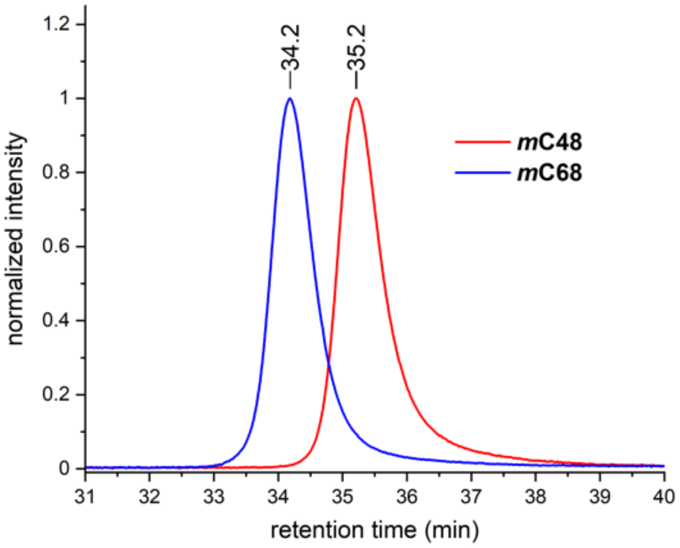

Figure 2. (a) Analytical GPC traces of masked thread compounds ***m*C48** and ***m*C68** (eluting with THF + 1% pyridine at a flow rate of 1.0 mL/min, λ = 525 nm).

(b)

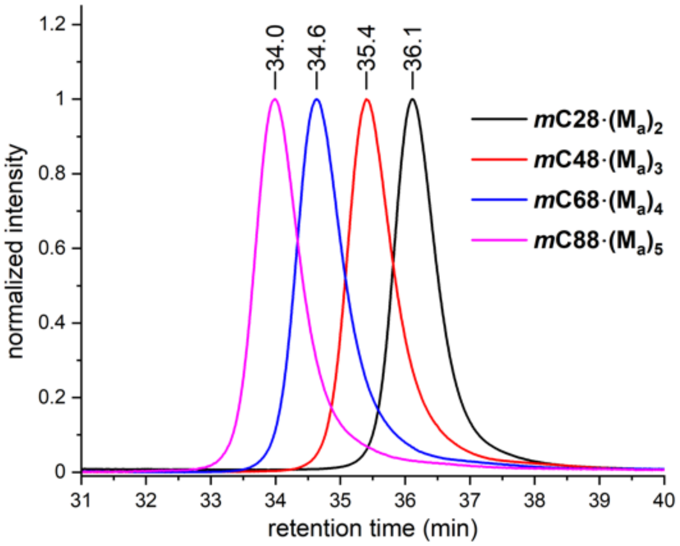

(b) Analytical GPC traces of masked polyrotaxanes ***m*C28·(M$_a$)$_2$**, ***m*C48·(M$_a$)$_3$**, ***m*C68·(M$_a$)$_4$**, and ***m*C88·(M$_a$)$_5$** (eluting with THF + 1% pyridine at a flow rate of 1.0 mL/min, λ = 525 nm).

(c)

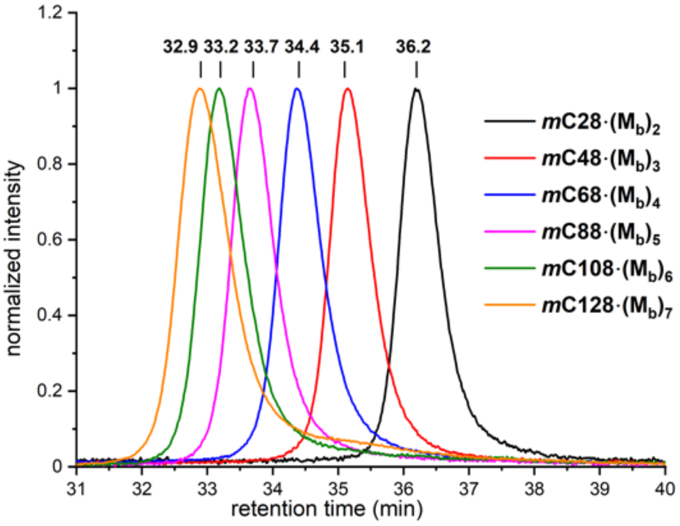

(c) Analytical GPC traces of masked polyrotaxanes ***m*C28·(M$_b$)$_2$**, ***m*C48·(M$_b$)$_3$**, ***m*C68·(M$_b$)$_4$**, ***m*C88·(M$_b$)$_5$**, ***m*C108·(M$_b$)$_6$**, and ***m*C128·(M$_b$)$_7$** (eluting with THF + 1% pyridine at a flow rate of 1.0 mL/min, λ = 480 nm).

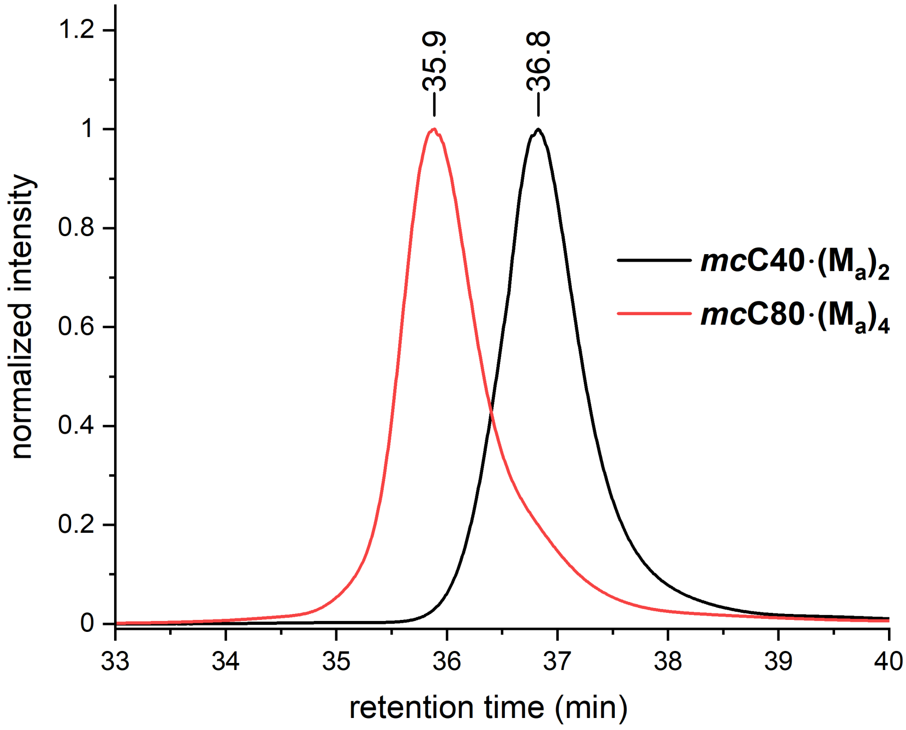

Figure 3. Analytical GPC traces of catenanes ***mc*C40·(M$_a$)$_2$** and ***mc*C80·(M$_a$)$_4$** (eluting with THF + 1% pyridine at a flow rate of 1.0 mL/min, λ = 525 nm).

Section 4: X-Ray Crystal Structures

Single crystal X-ray diffraction data for **3·M$_a$** and **3·M$_b$** were collected using beamline I19 at Diamond Light Source[9] and reduced using DIALS/XIA2.[10,11] Data for the [3]catenane ***mc*C40·(M$_a$)$_2$** were collected using a (Rigaku) Oxford Diffraction Super-Nova A diffractometer and were reduced or CrysAlisPro. Structures were solved using SuperFlip[12] and refined using CRYSTALS[13,14] as detailed in the CIF.

Crystallographic data have been deposited with the Cambridge Crystallographic Data Centre (CCDC 2224109–2224111) and can be obtained via www.ccdc.cam.ac.uk/data_request/cif

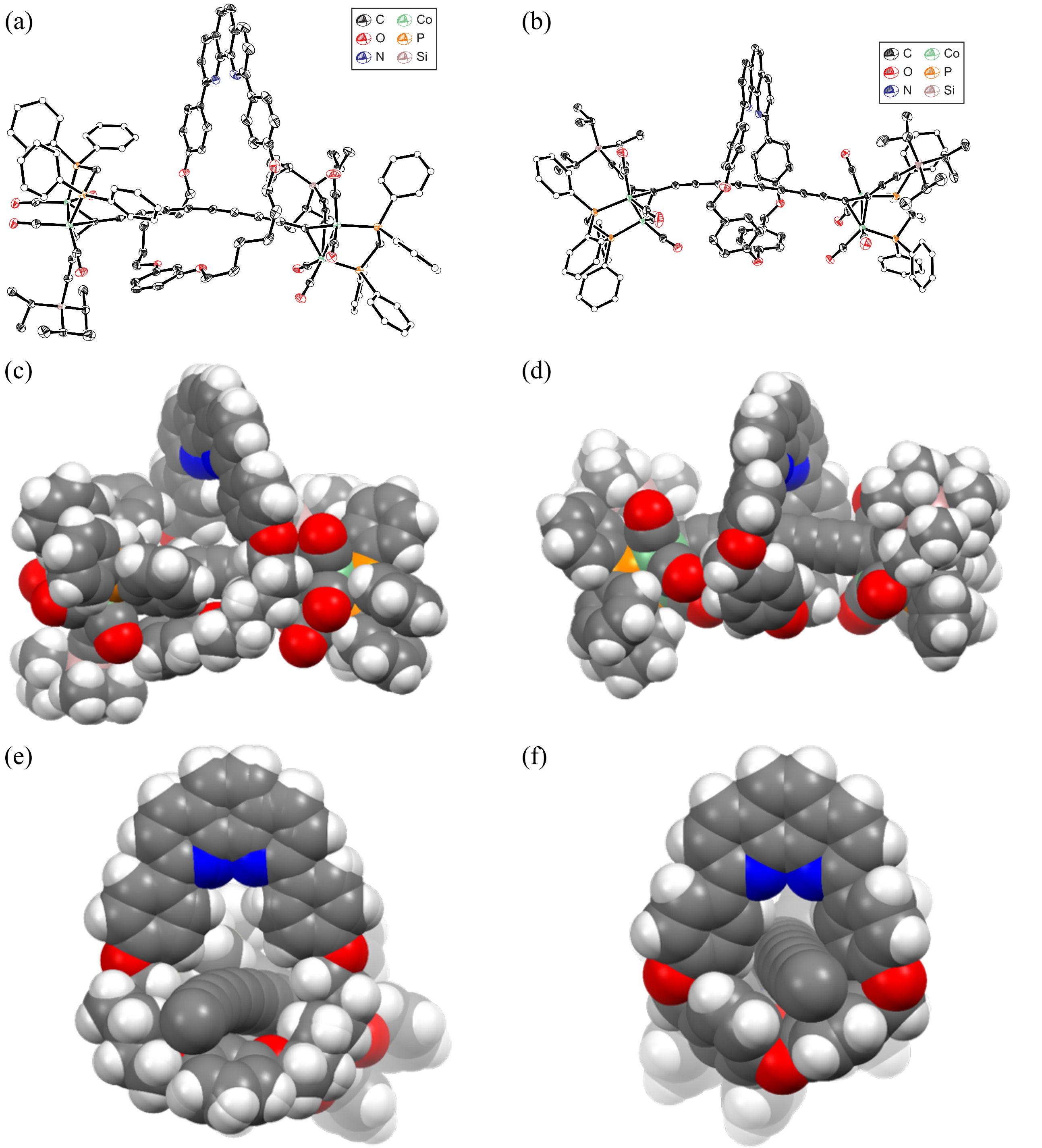

Figure 4. X-ray crystallographic structure of tetracobalt [2]rotaxanes **3·M$_a$** (left) and **3·M$_b$** (right). For (a-b) ellipsoids are plotted at 30% probability, hydrogen atoms omitted and dppm phenyl rings plotted as spheres for clarity. Space-filling models (c-f) are presented to highlight difference in macrocyclic cavity size between **M$_a$** and **M$_b$**.

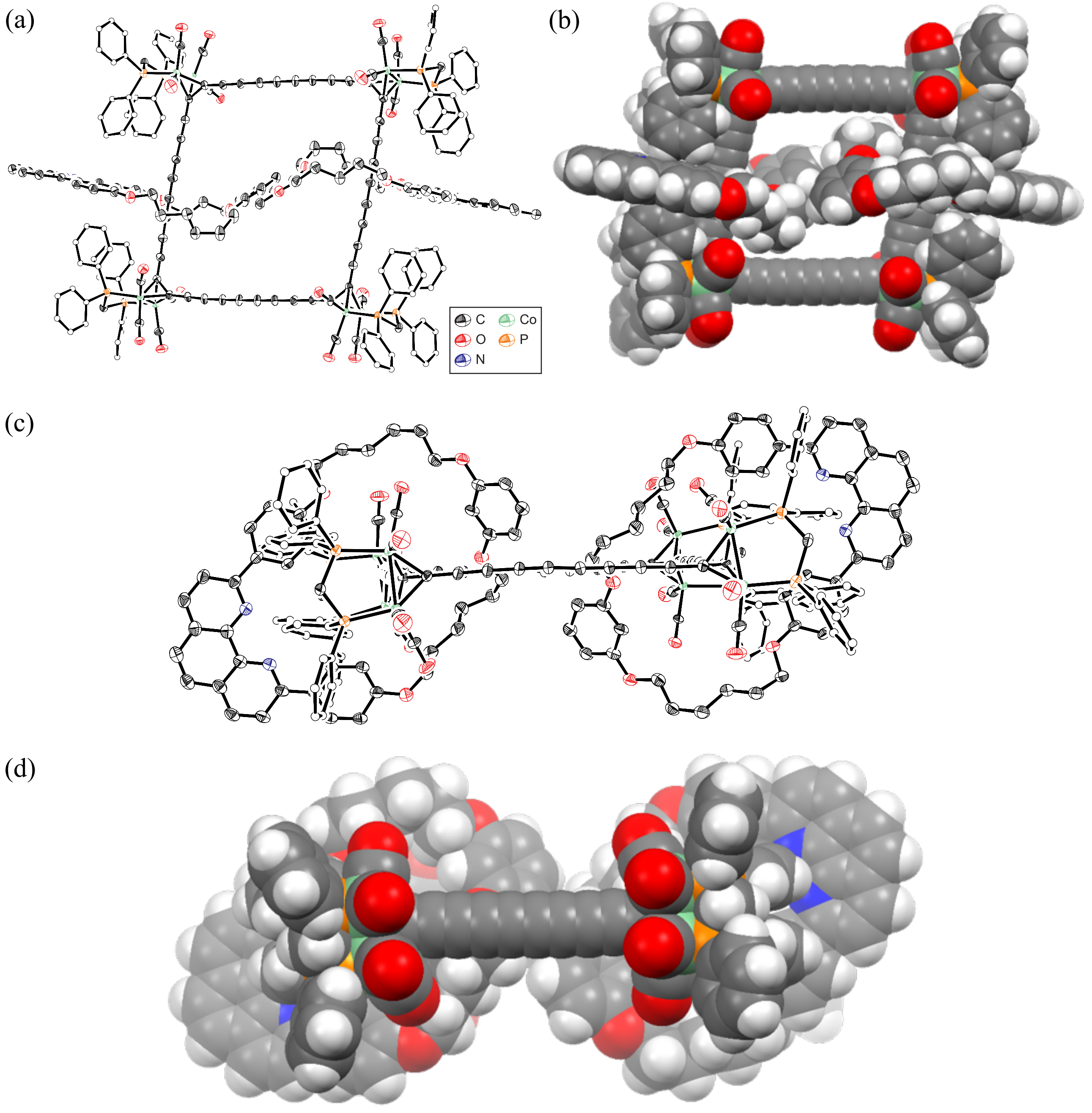

Figure 5. X-ray crystallographic structure of ***mc*C40·(M$_a$)$_2$**. For (a) and (c) ellipsoids are plotted at 30% probability, hydrogen atoms omitted and dppm phenyl rings plotted as spheres for clarity. Space-filling models are presented in (b) and (d).

checkCIF/PLATON report, alerts and responses

Tetracobalt [2]rotaxane **3·M$_a$** (CCDC 2224109)

`The following ALERTS were generated.`

`Alert level A`

`PLAT027_ALERT_3_A _diffrn_reflns_theta_full value (too) Low ......  21.30 Degree`

Author Response: The sample was weakly diffracting and failed give diffraction beyond around 22 degrees. Further, as the sample suffered radiation damage, it was not possible to increase the acquisition time further to increase the diffraction limit. Despite the limited resolution, the final result gives useful information and the data quality does not affect the conclusions drawn.

`Alert level B`

`PLAT029_ALERT_3_B _diffrn_measured_fraction_theta_full value Low .      0.958 Why?`

Author Response: Unfortunately, the sample suffered radiation damage so it was necessary to remove some of the data during processing leading to a reduced completeness. Despite the poor completeness, the final result gives useful information and the data quality does not affect the conclusions drawn.

`PLAT911_ALERT_3_B Missing FCF Refl Between Thmin & STh/L=    0.600       5800 Report`

Author Response: Because the sample suffered radiation damage, it was necessary to remove some of the data during processing. These reflections were missing from all areas of the data and led to a reduced completeness. Despite this, the final result gives useful information and the data quality does not affect the conclusions drawn.

Tetracobalt [2]rotaxane **3·M$_b$** (CCDC 2224110)

`The following ALERTS were generated.`

`Alert level B`

`PLAT021_ALERT_4_B Ratio Unique / Expected Reflections too High ...  1.555`

Author Response: These materials suffer from radiation damage, so although the sample was weakly diffracting it was not possible to increase the exposure as it would damage the integrity of the crystal. Despite the limited resolution, the final result gives useful information and the data quality does not affect the conclusions drawn.

`PLAT026_ALERT_3_B Ratio Observed / Unique Reflections (too) Low ..        35% Check`

Author Response: These materials suffer from radiation damage, so although the sample was weakly diffracting it was not possible to increase the exposure as it would damage the integrity of the crystal. Despite the limited resolution, the final result gives useful information and the data quality does not affect the conclusions drawn.

`PLAT430_ALERT_2_B Short Inter D...A Contact  O27      ..O27      .       2.79 Ang.`

Author Response: Although these are close they are in an interdigitated arrangement suggesting this is a preferred configuration.

`PLAT920_ALERT_1_B Theta(Max)  in CIF and FCF Differ by ..........       5.55 Degree`

Author Response: Data beyond the diffraction limit were removed using the Wilson plot. Although these reflections are flagged correctly in the FCF file, they are still included in the file although not in the calculation for theta(max) in the CIF.

```
PLAT934_ALERT_3_B Number of (Iobs-Icalc)/Sigma(W) > 10 Outliers ..          9 Check
```

Author Response: The data were a little noisy, thought to be the result of a combination of radiation damage, solvent modelling and crystal quality. The worst of the outlying reflections were removed, though nine remained.

---

[3]Catenane ***mc*C40·(M$_a$)$_2$** (CCDC 2224111)

```
The following ALERTS were generated.
```

Alert level B

```
PLAT920_ALERT_1_B Theta(Max)  in CIF and FCF Differ by ........... 8.30 Degree
```

Author Response: Data beyond the diffraction limit were removed using the Wilson plot. Although these reflections are flagged correctly in the FCF file, they are still included in the file although not in the calculation for theta(max) in the CIF.

Section 5: UV-Visible Absorption Spectra

(a)

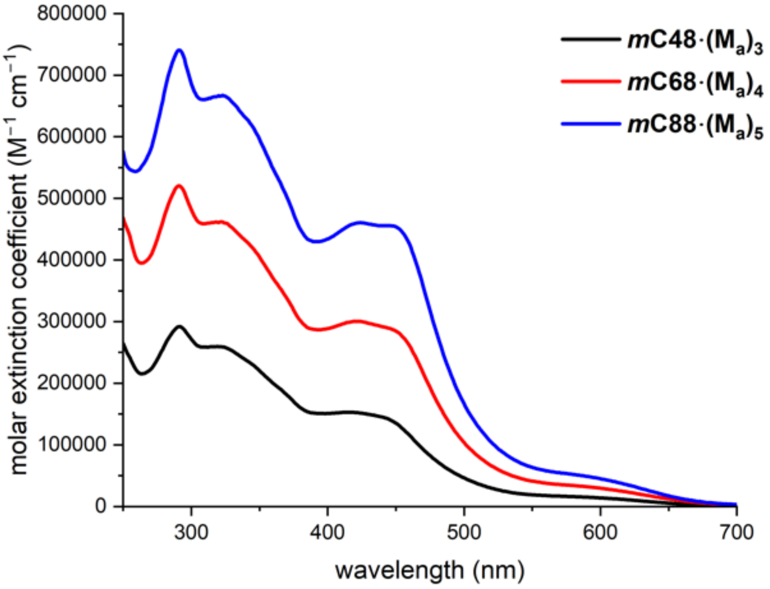

(b)

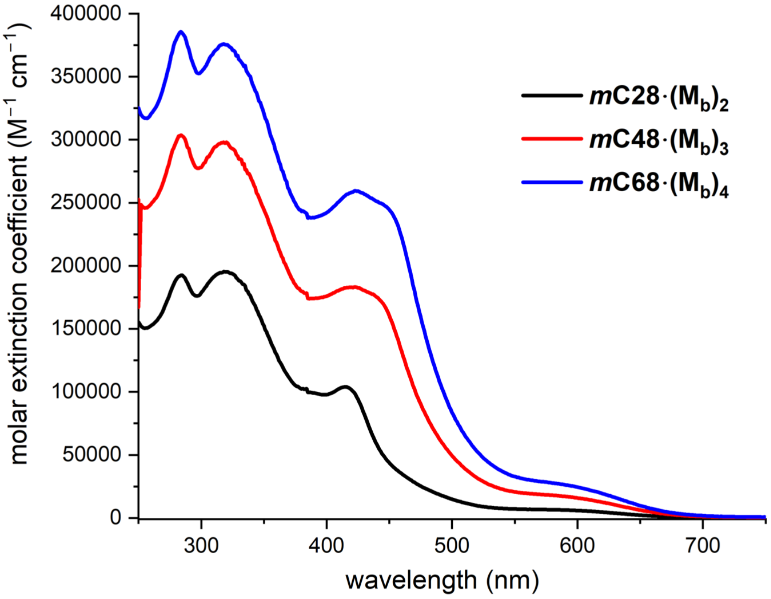

Figure 6. UV-vis spectra of masked polyrotaxanes (a) ***m*C48·(M$_a$)$_3$**, ***m*C68·(M$_a$)$_4$**, and ***m*C88·(M$_a$)$_5$** and (b) ***m*C28·(M$_b$)$_2$**, ***m*C48·(M$_b$)$_3$**, and ***m*C68·(M$_b$)$_4$** as measured in $CHCl_3$ at room temperature.

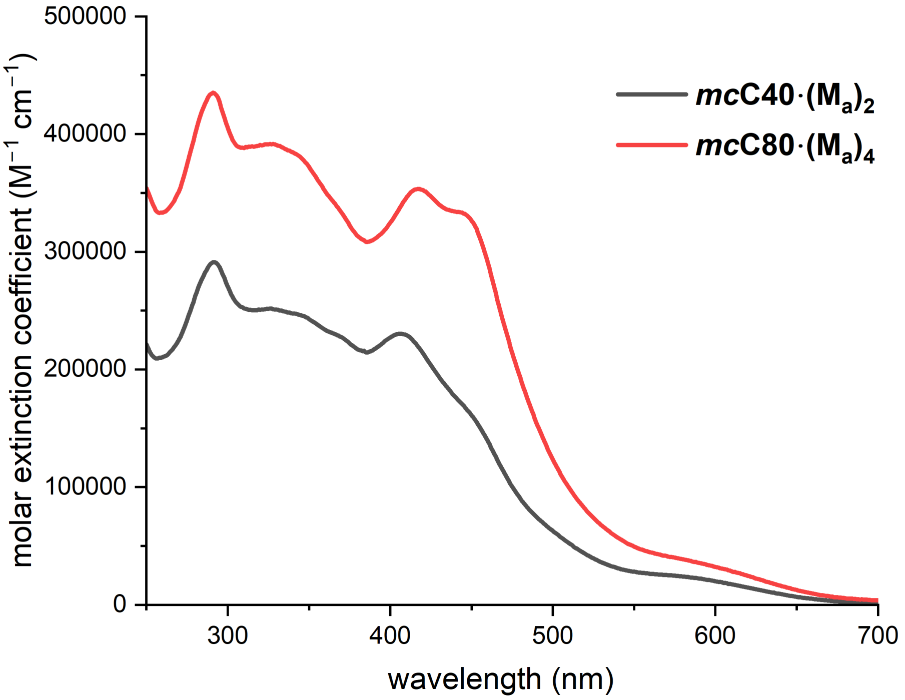

Figure 7. UV-vis spectra of masked catenanes ***mc*C40·(M$_a$)$_2$** and ***mc*C80·(M$_a$)$_4$** as measured in $CHCl_3$ at room temperature.

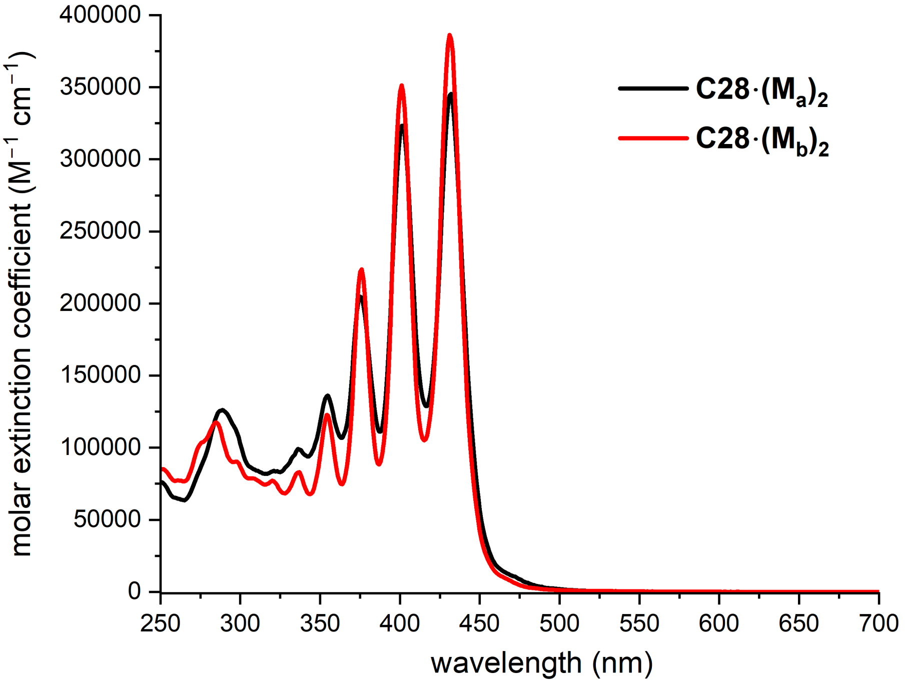

Figure 8. UV-vis spectra of polyyne [3]rotaxanes **C28·(M$_a$)$_2$** and **C28·(M$_b$)$_2$** as measured in $CHCl_3$ at room temperature.

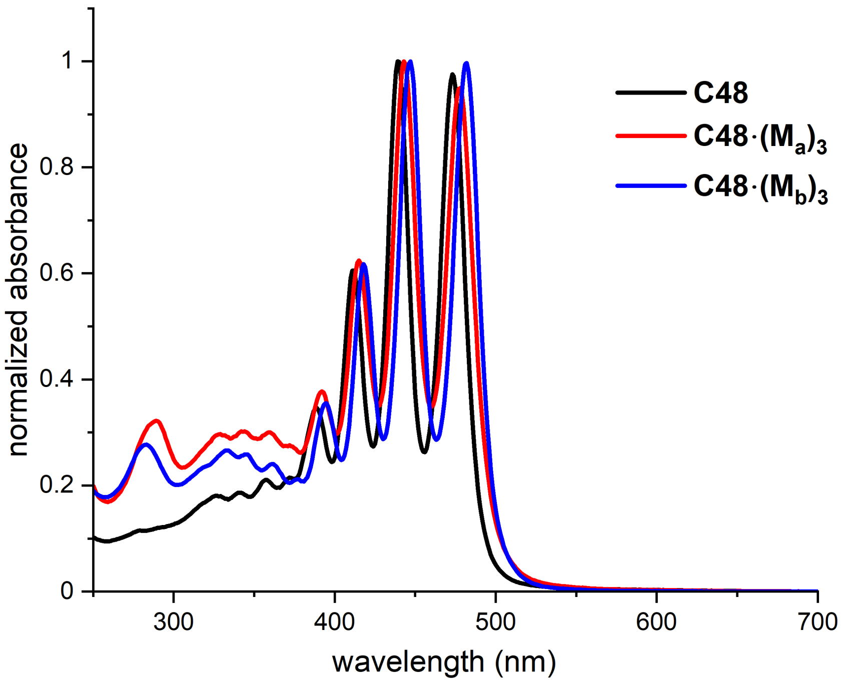

Figure 9. Normalized UV-vis spectra of polyyne **C48**, polyyne rotaxanes **C48·(M$_a$)$_3$** and **C48·(M$_b$)$_3$** as measured in $CHCl_3$ at room temperature.

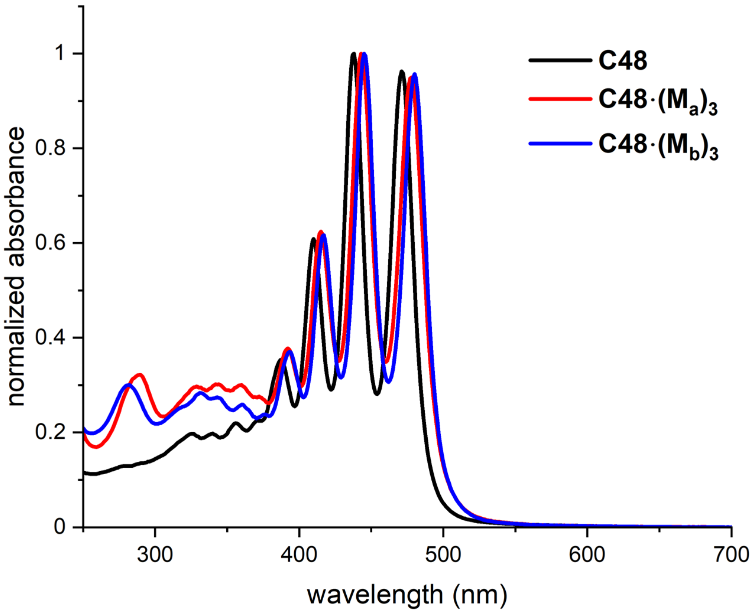

Figure 10. Normalized UV-vis spectra of polyyne **C48**, polyyne rotaxanes **C48·(M$_a$)$_3$** and **C48·(M$_b$)$_3$** as measured in CH_2Cl_2 at room temperature.

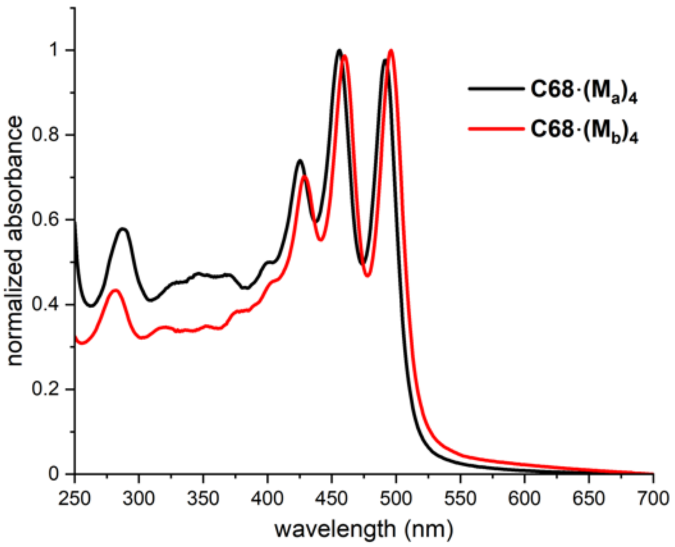

Figure 11. Normalized UV-vis spectra of polyyne rotaxanes **C68·(M$_a$)$_4$** and **C68·(M$_b$)$_4$** as measured in CH_2Cl_2 at room temperature.

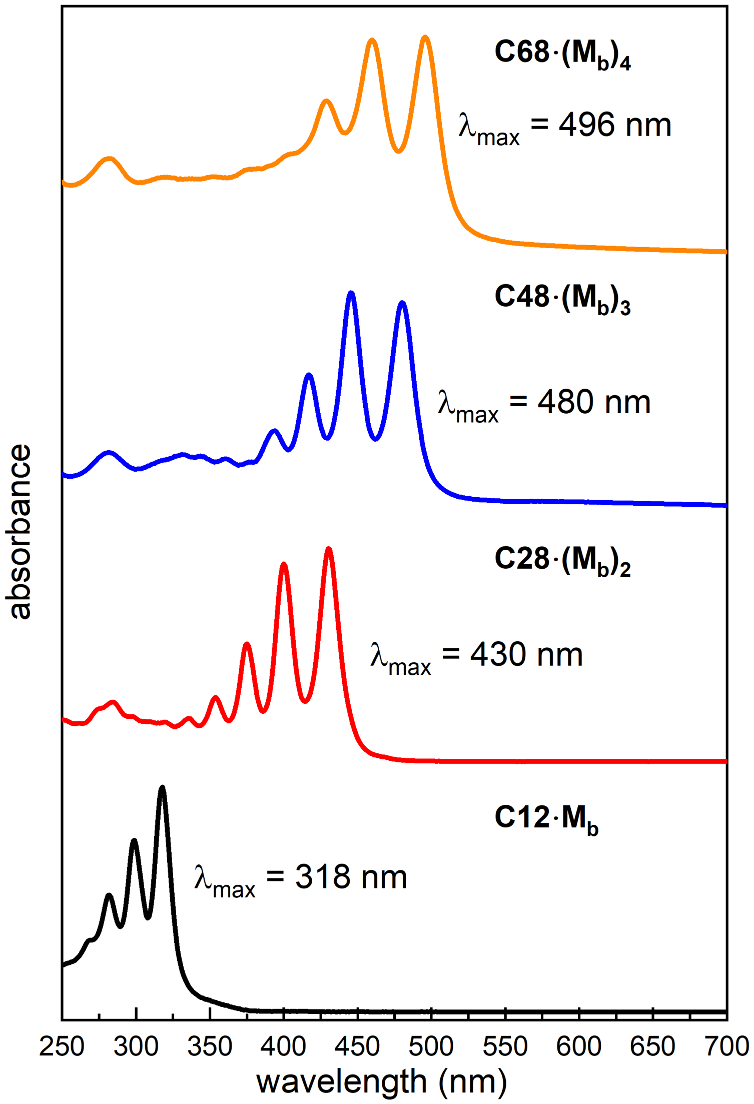

Figure 12. UV-vis spectra of **C12·M$_b$**, **C28·(M$_b$)$_2$**, **C48·(M$_b$)$_3$**, and **C68·(M$_b$)$_4$** as measured in CH_2Cl_2 at room temperature.

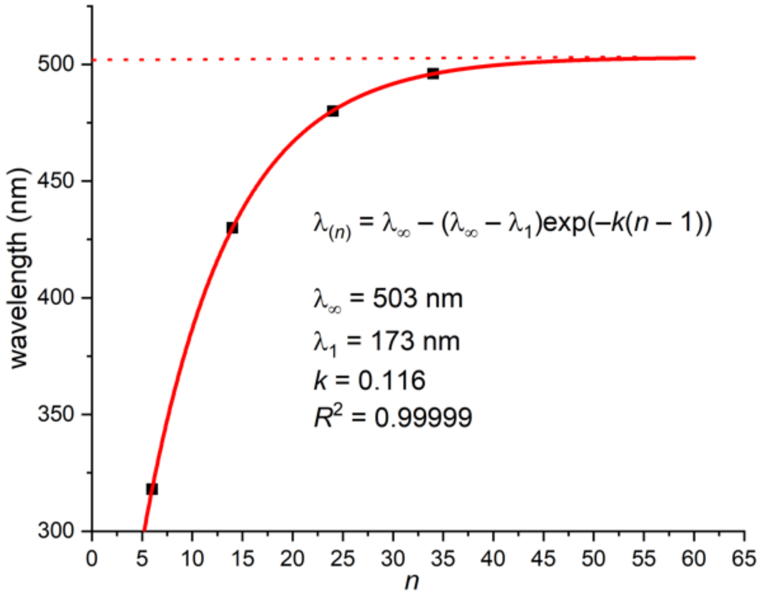

Figure 13. Convergence of λ_{max} for the series of polyyne rotaxanes **C12·M$_b$**, **C28·(M$_b$)$_2$**, **C48·(M$_b$)$_3$**, and **C68·(M$_b$)$_4$** fitted to Meier equation $\lambda(n) = \lambda_\infty - (\lambda_\infty - \lambda_1)e^{-k(n-1)}$.

Table 1. Data analysis for **Figure 13** in the series of polyyne rotaxane with **M$_b$**.

n	$\lambda_{max(exp)}$	$\lambda_{max(cal)}$
6	318	318.0
14	430	429.9
24	480	480.2
34	496	495.9

Table 2. Variables for equation $\lambda(n) = \lambda_\infty - (\lambda_\infty - \lambda_1)e^{-k(n-1)}$ in **Figure 13** in the series of polyyne rotaxane incorporating **M$_b$**.

**variable**	**value**
λ_∞	503.1 ± 0.3
λ_1	172.5 ± 0.9
k	0.1160 ± 0.0005
R^2	0.99999

Section 6: Raman Spectra

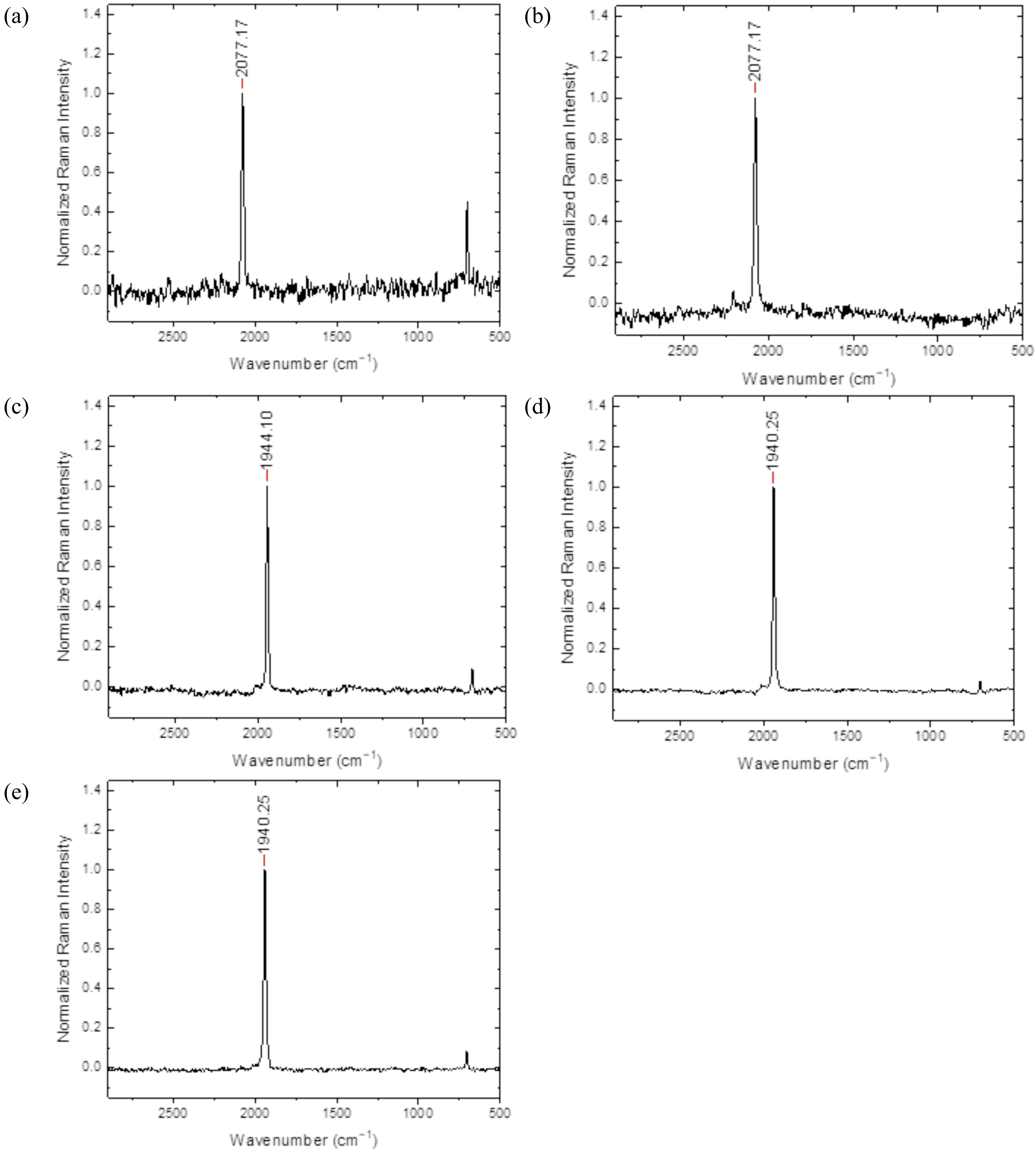

Figure 14. FT-Raman spectra of (a) polyyne dumbbell **C12**, laser power: 200 mW, 32 accumulations, (b) polyyne rotaxane **C12·M$_b$**, laser power: 200 mW, 32 accumulations, (c) polyyne dumbbell **C28**, laser power: 200 mW, 64 accumulations, (d) polyyne rotaxane **C28·(M$_a$)$_2$**, laser power: 50 mW, 64 accumulations, and (e) polyyne rotaxane **C28·(M$_b$)$_2$**, laser power: 50 mW, 64 accumulations; all measured in CH_2Cl_2 (ca. 1 mM) at room temperature; excitation: 1064 nm, spectral resolution: 4 cm^{-1}. Prior to analysis and normalization, the Raman spectra were baseline-corrected by subtracting a third order polynomial curve, using Origin software. The peak at 704 cm^{-1} can be assigned to the CH_2Cl_2 solvent.

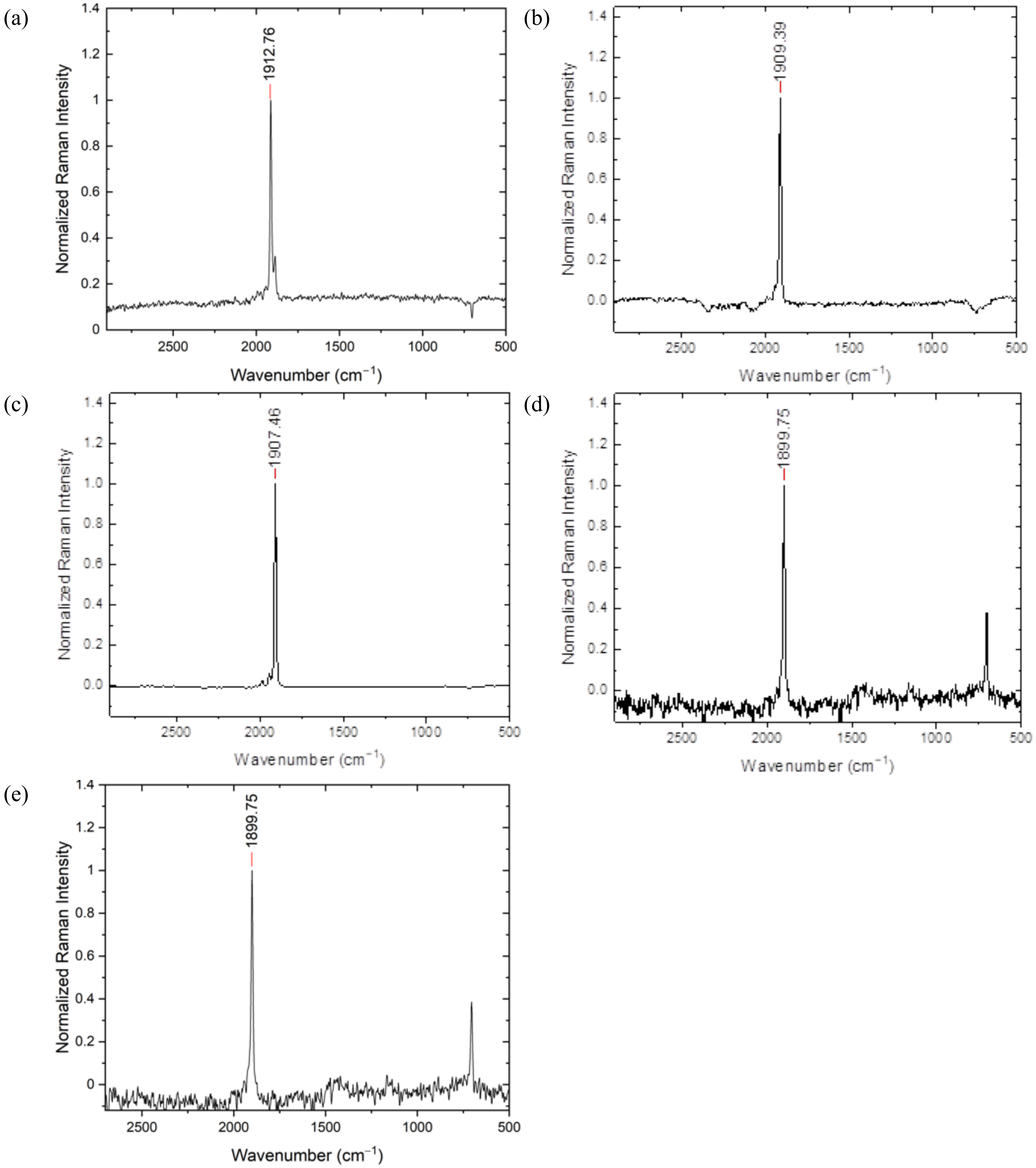

Figure 15. FT-Raman spectra of (a) **C48**, laser power: 100 mW, 32 accumulations; polyyne rotaxanes (b) **C48·(M$_a$)$_3$**, laser power: 100 mW, 32 accumulations, (c) **C48·(M$_b$)$_3$**, laser power: 50 mW, 64 accumulations, (d) **C68·(M$_a$)$_4$**, laser power: 50 mW, 64 accumulations and (e) **C68·(M$_b$)$_4$**, laser power: 100 mW, 128 accumulations; all measured in CH_2Cl_2 (ca. 1 mM) at room temperature; excitation: 1064 nm, spectral resolution: 4 cm^{-1}. Prior to analysis and normalization, the Raman spectra were baseline-corrected by subtracting a third order polynomial curve, using Origin software. The peak at 704 cm^{-1} can be assigned to the CH_2Cl_2 solvent.

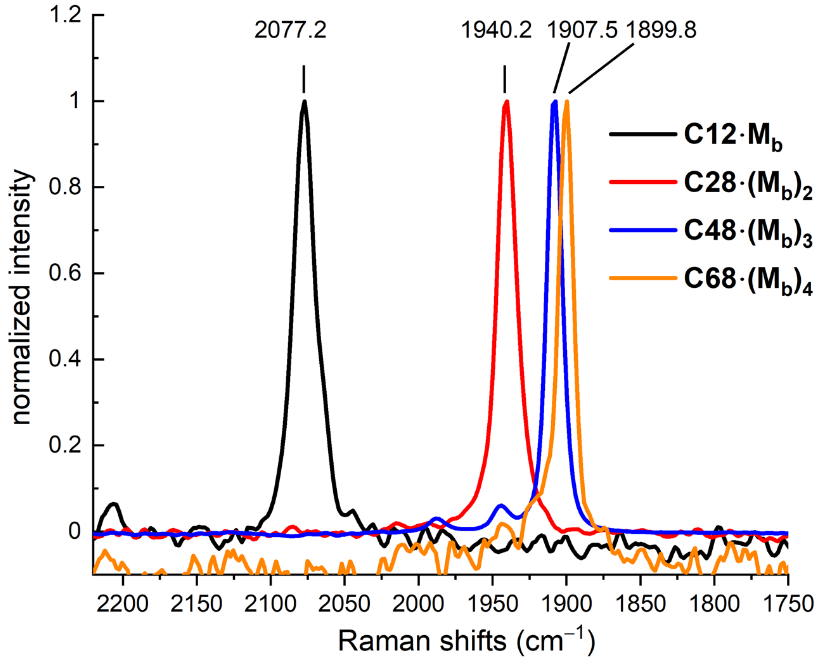

Figure 16. Normalized FT-Raman spectra of **C12·M$_b$**, **C28·(M$_b$)$_2$**, **C48·(M$_b$)$_3$**, and **C68·(M$_b$)$_4$** as measured in CH_2Cl_2 (ca. 1 mM) at room temperature; excitation: 1064 nm, spectral resolution: 4 cm^{-1}.

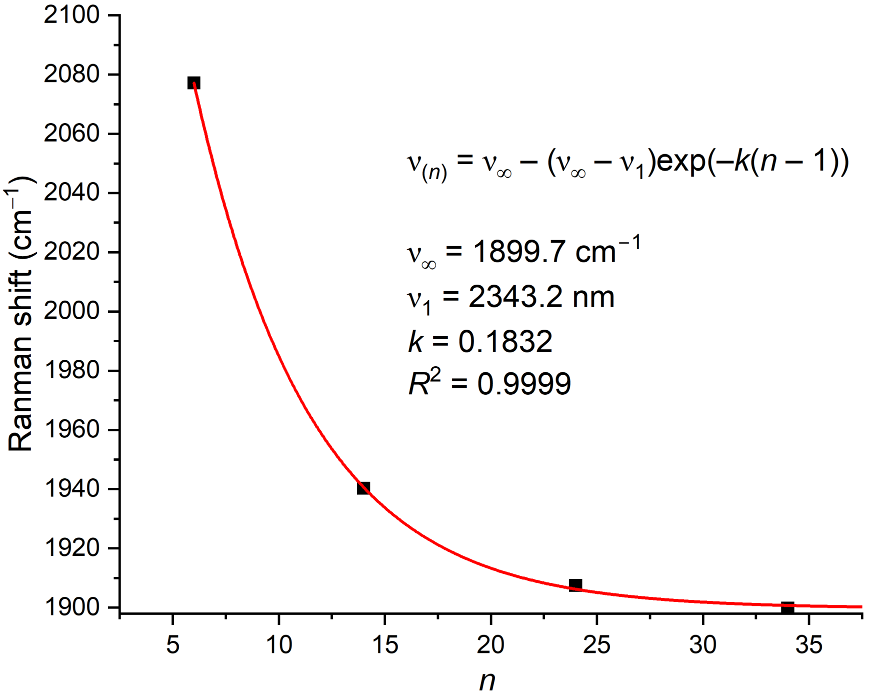

Figure 17. Convergence of Raman shifts for the series of polyyne rotaxane incorporating **M$_b$** by using the Meier equation $v(n) = v_\infty - (v_\infty - v_1)e^{-k(n-1)}$. The experimental Raman shift of compound **C68·(M$_b$)$_4$** happens to equal the predicated value of v_∞ 1900 cm^{-1} for infinite length, which illustrates the fact that the shift saturated at a length of ca. 68 sp-carbons.

Table 3. Data analysis for **Figure 17** in the series of polyyne rotaxane incorporating $\mathbf{M_b}$.

n	$v_{(exp)}$	$v_{(cal)}$
6	2077.2	2077.2
14	1940.3	1940.7
24	1907.5	1906.2
34	1899.7	1900.7

Table 4. Variables for equation $v(n) = v_\infty - (v_\infty - v_1)e^{-k(n-1)}$ in **Figure 17** in the series of polyyne rotaxane incorporating $\mathbf{M_b}$.

**variable**	**value**
v_∞	1899.7 ± 1.4
v_1	2343.2 ± 14.8
k	0.1832 ± 0.0067
R^2	0.99987

Section 7: Comparison of NMR Spectra

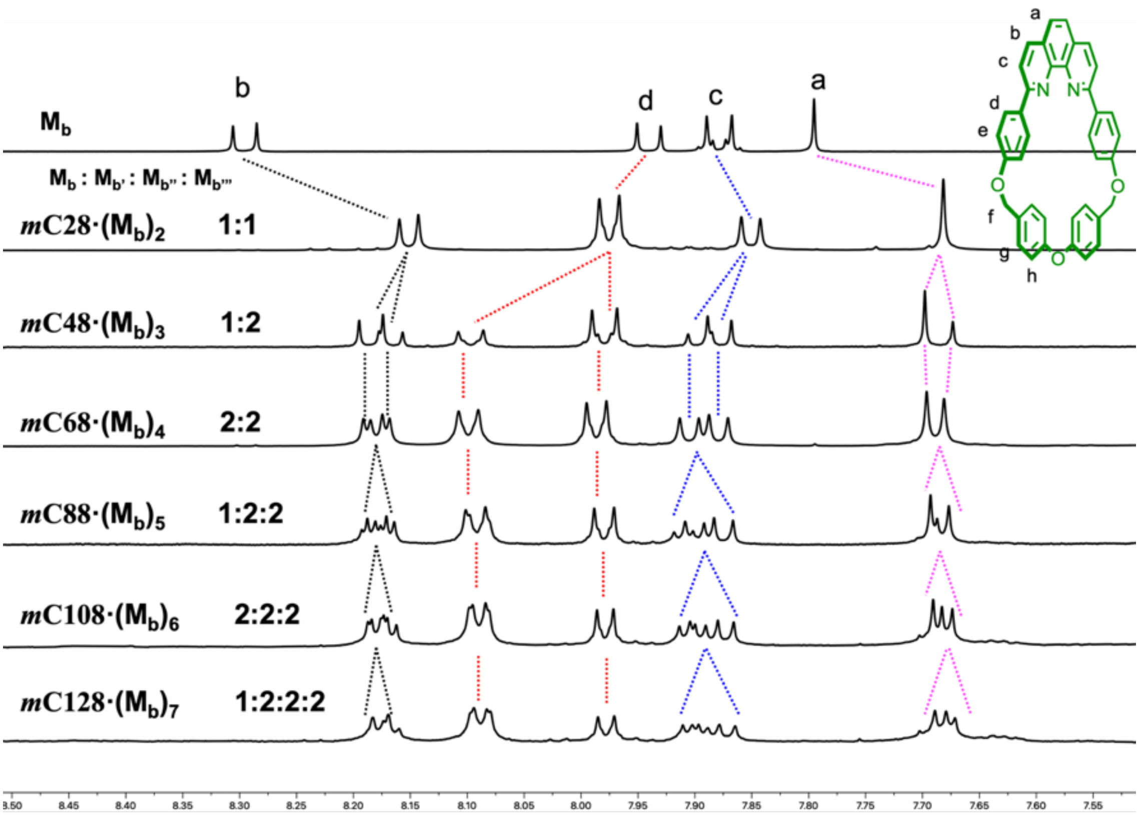

Figure 18. Comparison of partial ^{1}H NMR spectra (8.5–7.5 ppm) of **M**$_b$ (400 MHz) and masked poly[*n*]rotaxanes (*n* = 3–8) ***m*C28·(M**$_b$**)**$_2$ (500 MHz), ***m*C48·(M**$_b$**)**$_3$ (400 MHz), ***m*C68·(M**$_b$**)**$_4$ (500 MHz), ***m*C88·(M**$_b$**)**$_5$ (500 MHz), ***m*C108·(M**$_b$**)**$_6$ (600 MHz), and ***m*C128·(M**$_b$**)**$_7$ (600 MHz) in CD_2Cl_2.

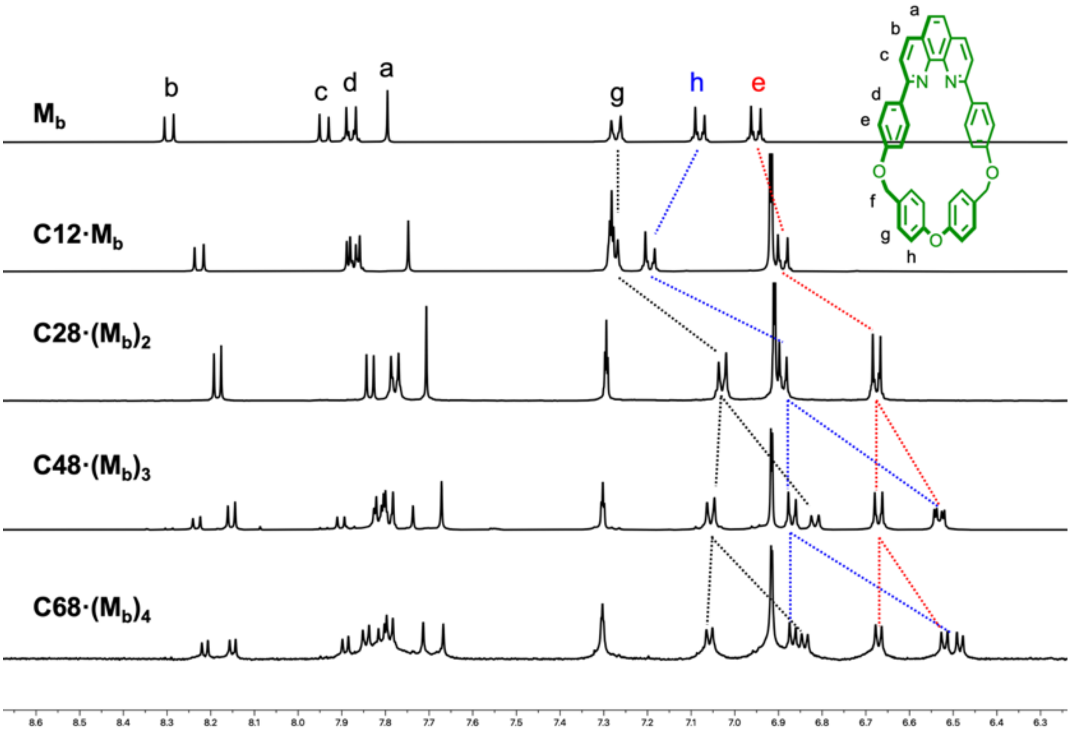

Figure 19. Comparison of partial ^{1}H NMR spectra (8.6–6.3 ppm) of macrocycle **M**$_b$ (400 MHz) and polyyne rotaxanes **C12·M**$_b$ (400 MHz), **C28·(M**$_b$**)**$_2$ (500 MHz), **C48·(M**$_b$**)**$_3$ (500 MHz), and **C68·(M**$_b$**)**$_4$ (600 MHz) in CD_2Cl_2.

Section 8: Solid-State Thermal Stability

Stability tests were performed for solid-state samples of the polyyne dumbbell **C48** and polyyne rotaxanes **C48·(M$_b$)$_3$** at 30 °C. To perform this experiment, concentrated solutions of **C48** and **C48·(M$_b$)$_3$** in CH_2Cl_2 were prepared, such that diluting 50 µL of the concentrated solution into 1.5 mL of CH_2Cl_2 in a cuvette with a pathlength of 10 mm gave a UV-vis absorption spectrum with an optical density of ca. 1 at λ_{max}. About 80 samples were prepared by transferring 50 µL of the concentrated solution into 80 vials. The solvent in each vial was evaporated and the residue was dried under high vacuum for ca. 10 min. These solid samples were then stored in an oven at 30 °C, wrapped with aluminum foil to shield from light. Three parallel measurements were recorded for each compound. **C48** and **C48·(M$_b$)$_3$** samples were dissolved in CH_2Cl_2 and analyzed by UV-vis absorption spectroscopy at each 12 h interval and 48 h interval, respectively. Data points were averaged over three parallel measurements, because the solid films were formed unevenly on the wall of glass vials, resulting in some variation in the rate of decomposition.

The thermal decomposition was fitted to a first-order decay model, $A(t) = a\cdot\exp(-kt)$. For the polyyne dumbbell **C48**, the fit is excellent with $R^2 = 0.99$, giving the decaying rate of $k = 0.01125$ h^{-1}; half-life of $t_{1/2} =$ 62 h (**Figure 20a**). For the polyyne rotaxane **C48·(M$_b$)$_3$**, the fit is excellent with $R^2 = 0.98$ giving $k = 0.000678$ h^{-1}; half lifetime of $t_{1/2} =$ 1022 h (**Figure 20b**). These results show that the polyyne rotaxane is 16.5 times more stable than the polyyne dumbbell. The plots in Figure 4 are the same as those in Figure 20a-c, except that in Figure 4 the absorbance is normalized, by dividing by *a*, to make it easier to compare the decay curves.

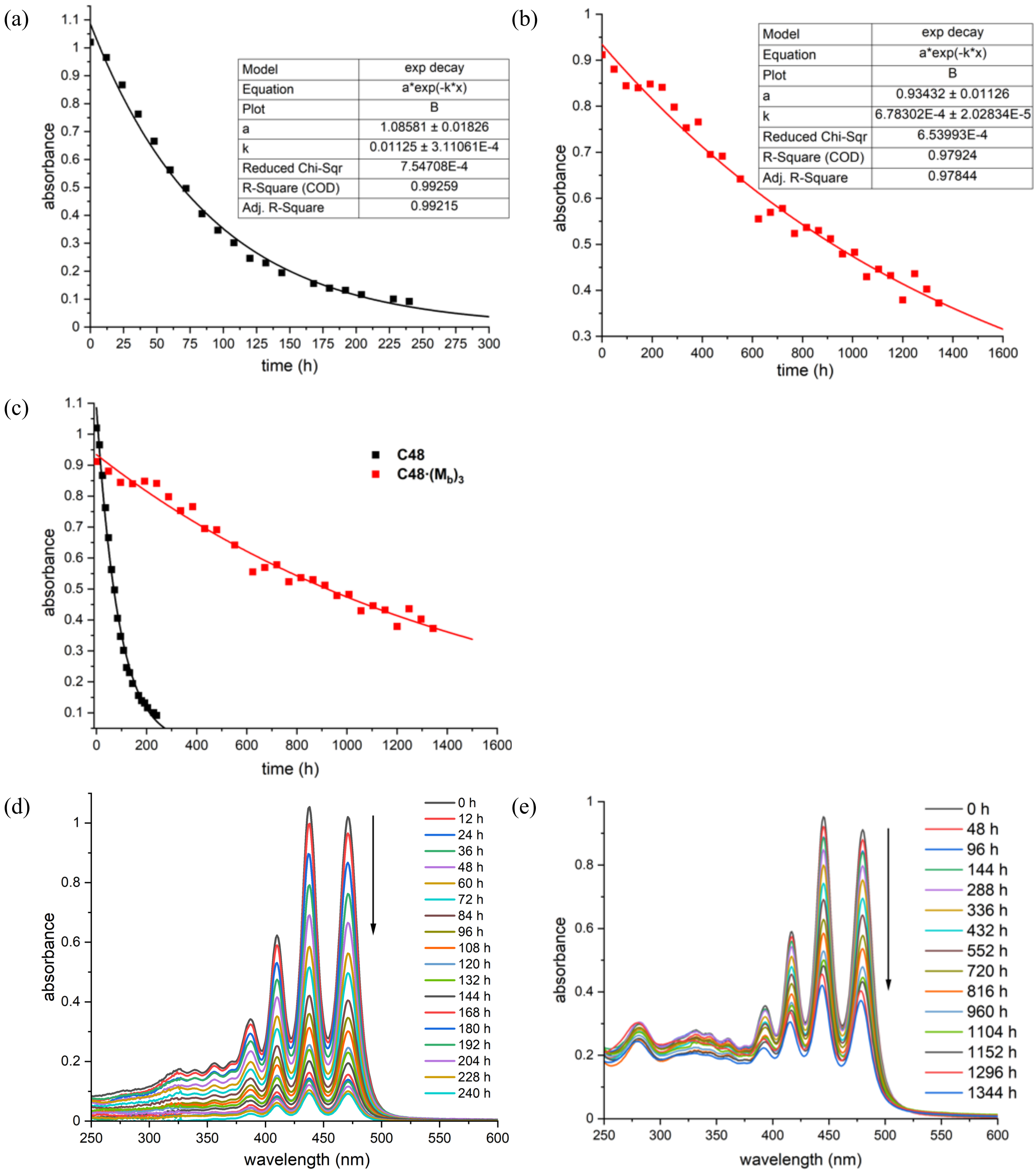

Figure 20. (a) Plot of UV-vis absorbance decay for solid-state polyyne dumbbell **C48** monitored at λ_{max} = 471 nm over a period of 240 h at 30 °C, as measured in CH_2Cl_2. (b) Plot of UV-vis absorbance decay for solid-state polyyne rotaxane **C48·(M$_b$)$_3$** monitored at λ_{max} = 480 nm over a period of 1344 h at 30 °C. An overlaid plot of absorbance of **C48** and **C48·(M$_b$)$_3$** from (a) and (b) is plotted in (c) to aid comparison. Selected UV-vis spectra during the decomposition process of (d) polyyne dumbbell **C48** and (e) polyyne rotaxane **C48·(M$_b$)$_3$**.

Section 9: NMR Spectra

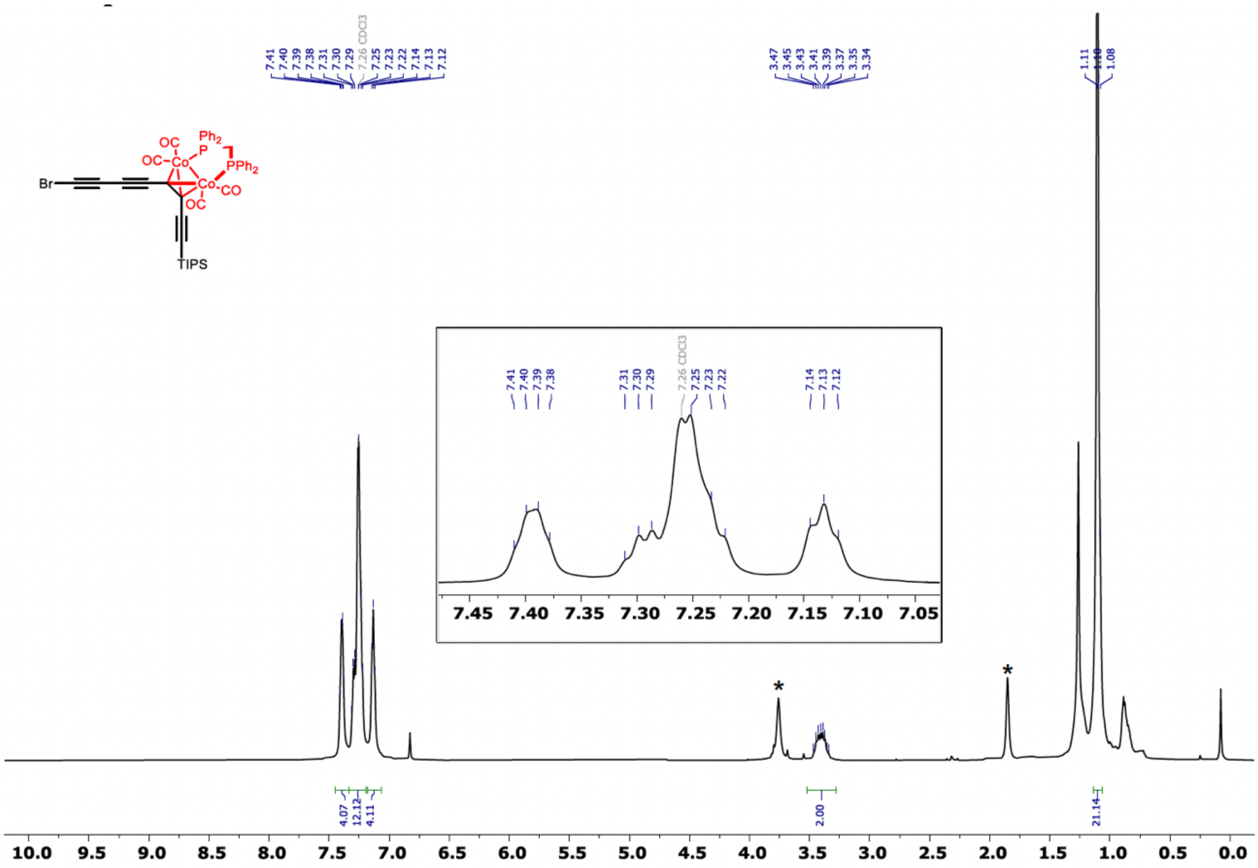

Figure 21. ^{1}H NMR (600 MHz) spectrum of compound **2** in $CDCl_3$ (*indicates residual THF solvent).

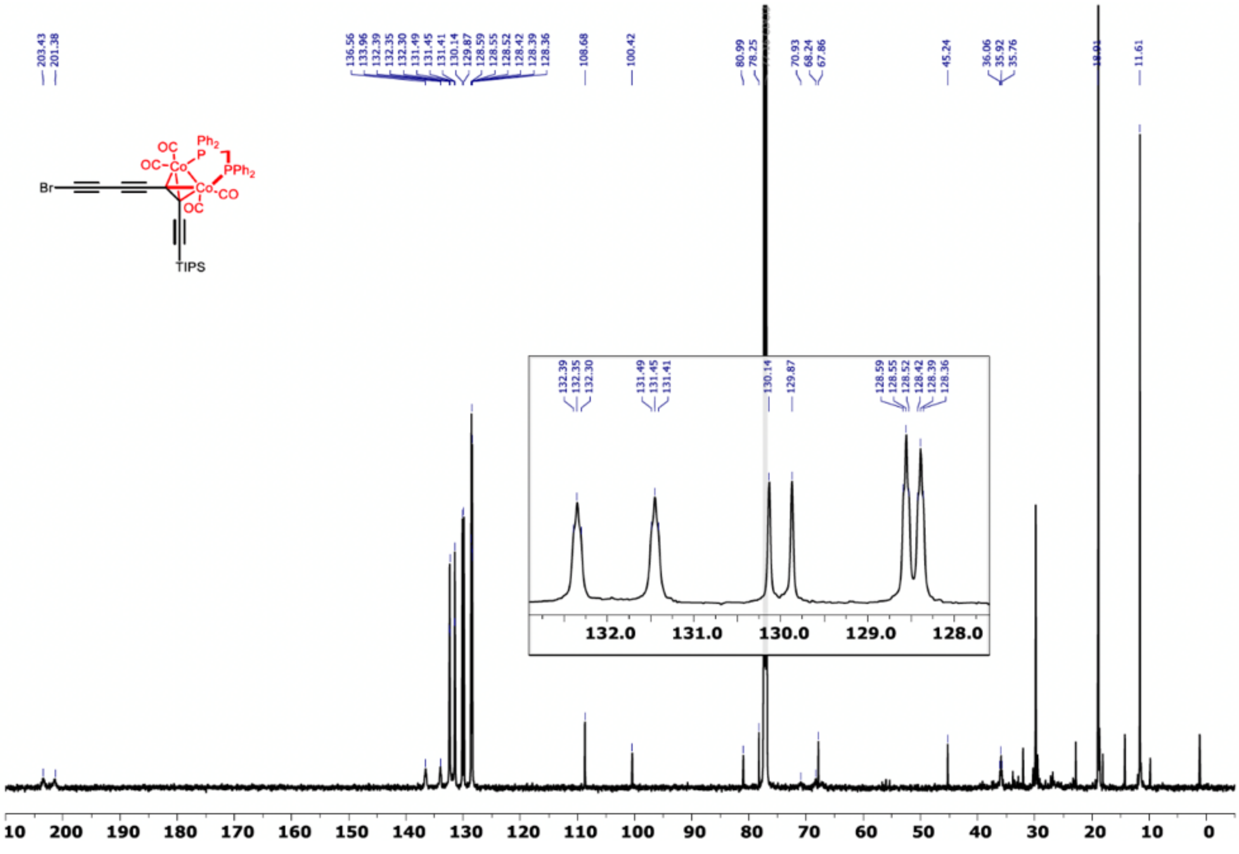

Figure 22. $^{13}C\{^{1}H\}$ NMR (151 MHz) spectrum of compound **2** in $CDCl_3$.

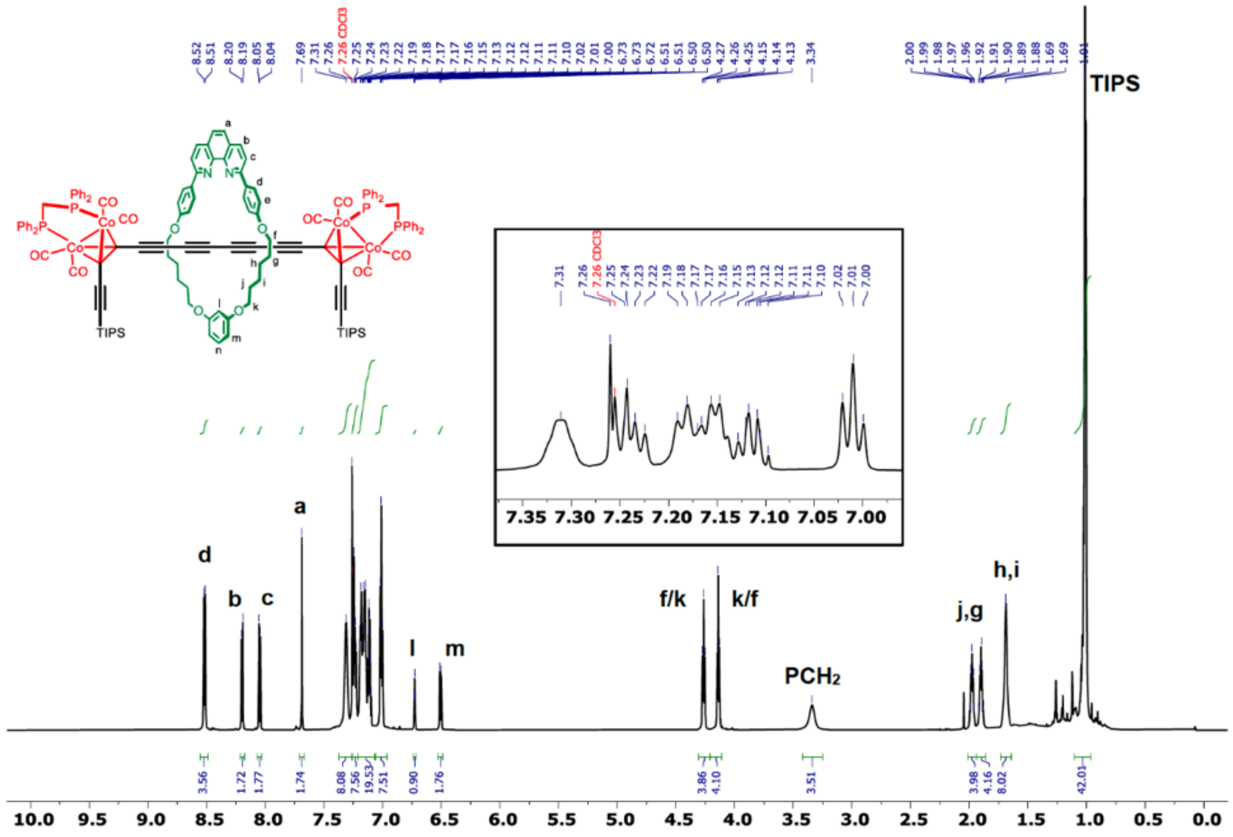

Figure 23. ^{1}H NMR (600 MHz) spectrum of compound **3·M$_a$** in CD_2Cl_2.

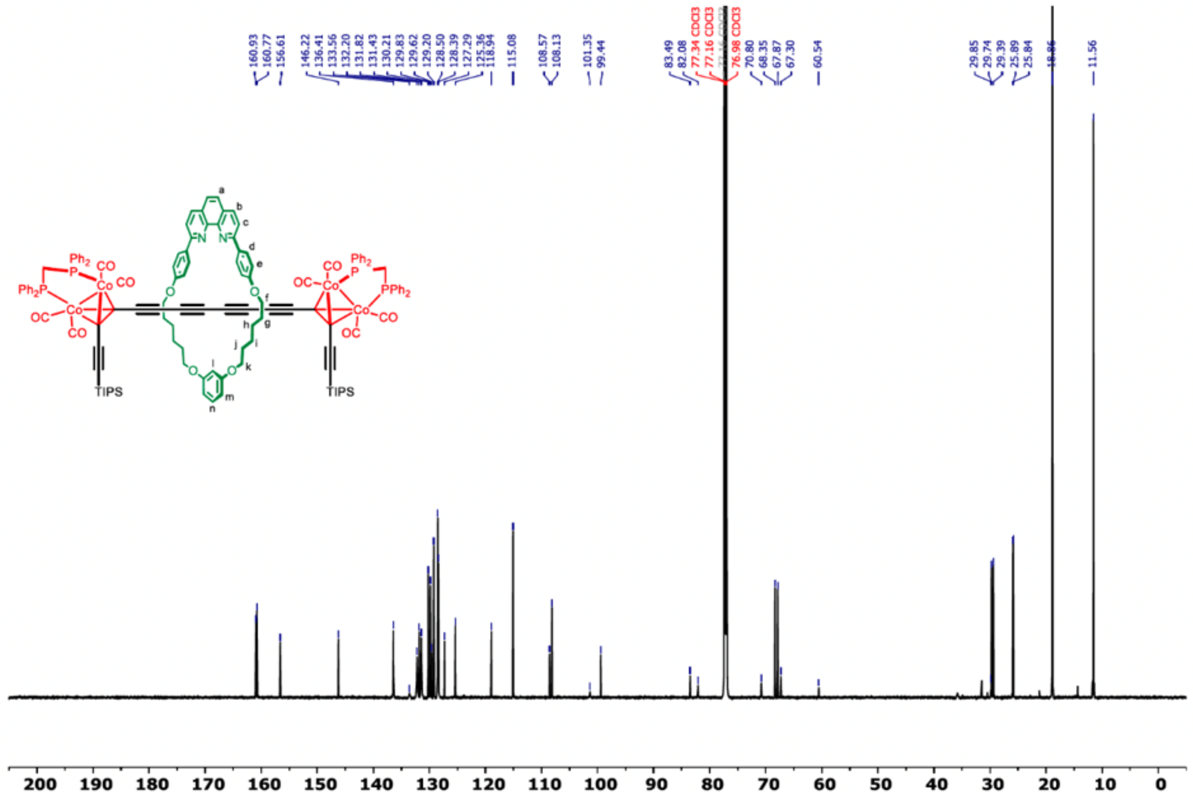

Figure 24. $^{13}C\{^{1}H\}$ NMR (MHz) spectrum of compound **3·M$_a$** in CD_2Cl_2.

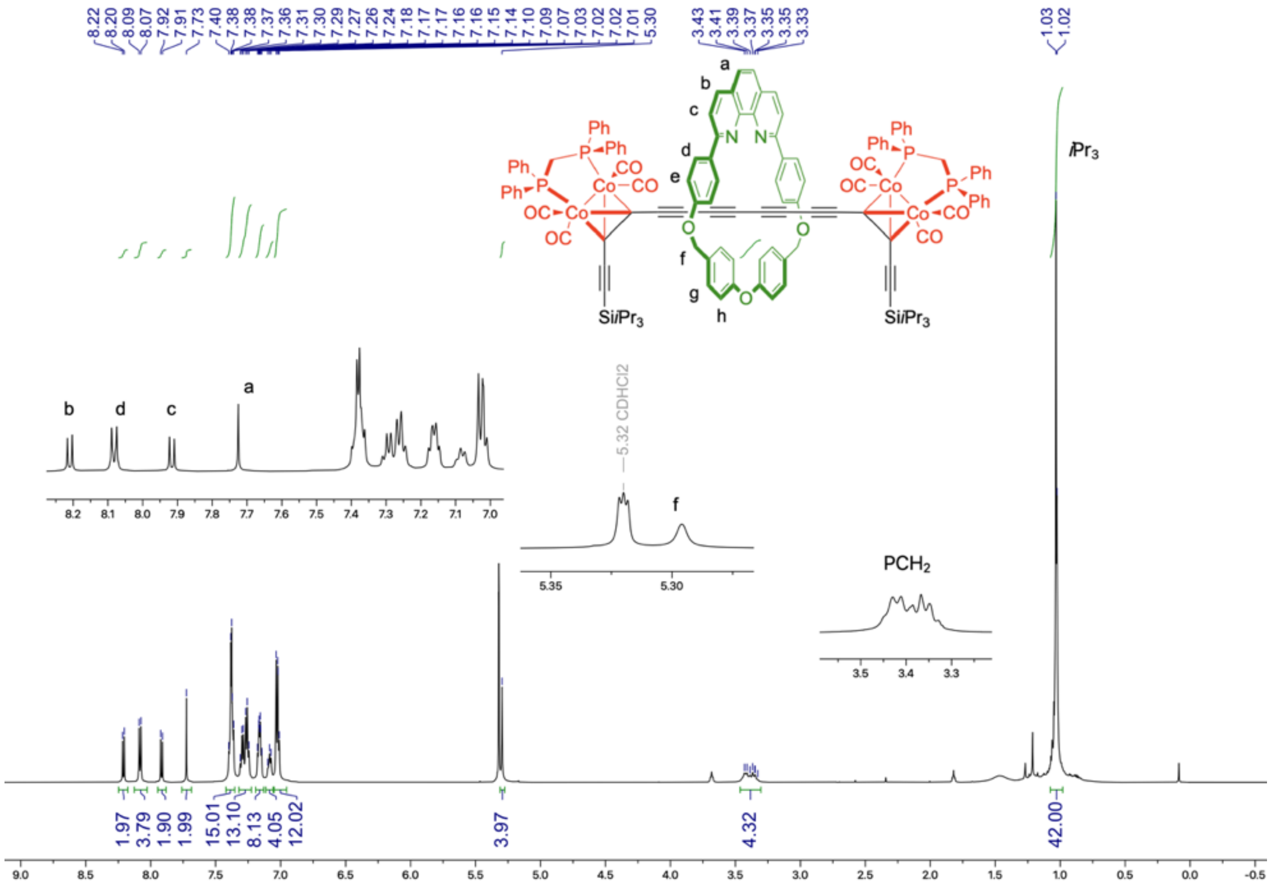

Figure 25. ^{1}H NMR (600 MHz) spectrum of compound **3·M$_b$** in CD_2Cl_2.

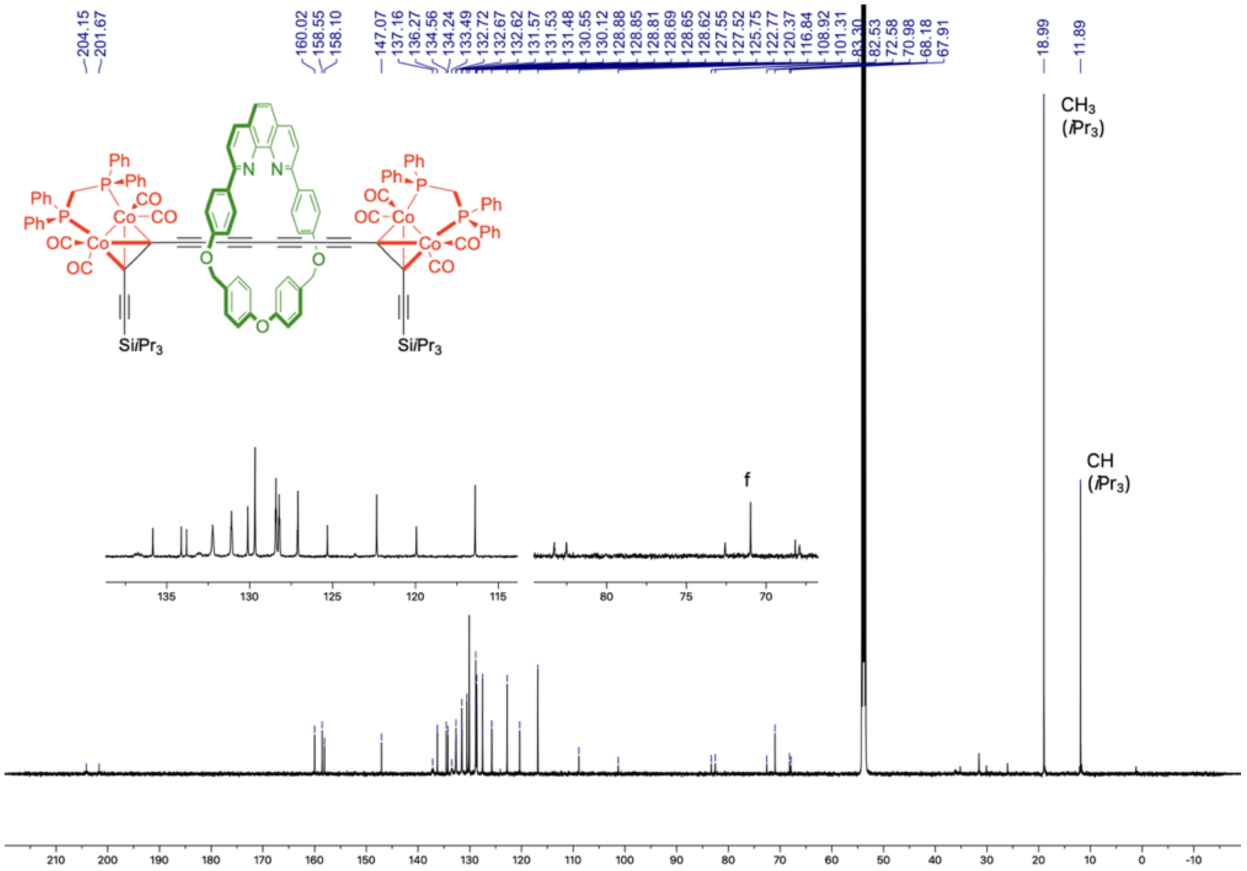

Figure 26. $^{13}C\{^{1}H\}$ NMR (151 MHz) spectrum of compound **3·M$_b$** in CD_2Cl_2.

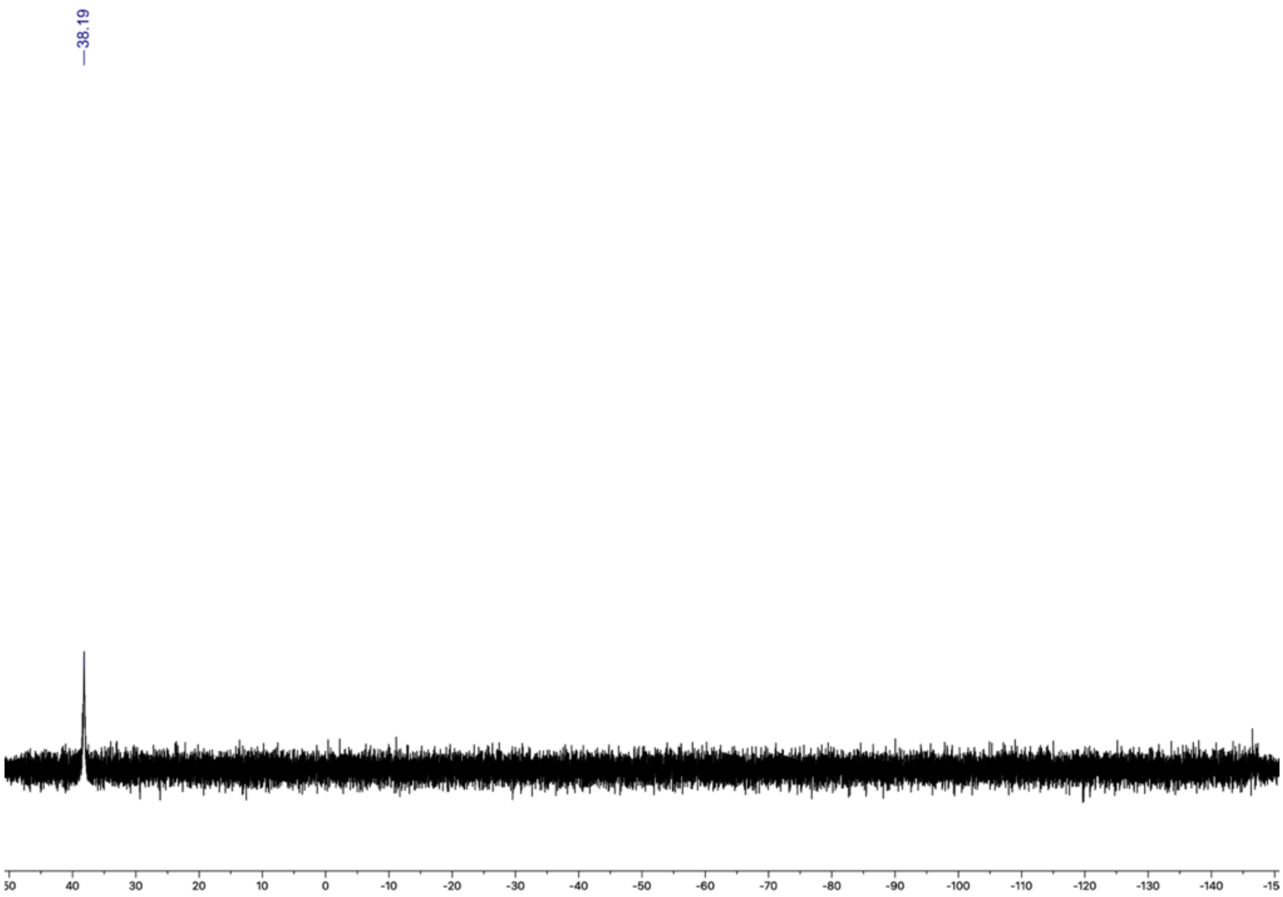

Figure 27. $^{31}P\{^1H\}$ NMR (202 MHz) spectrum of compound **3·M$_b$** in CD_2Cl_2.

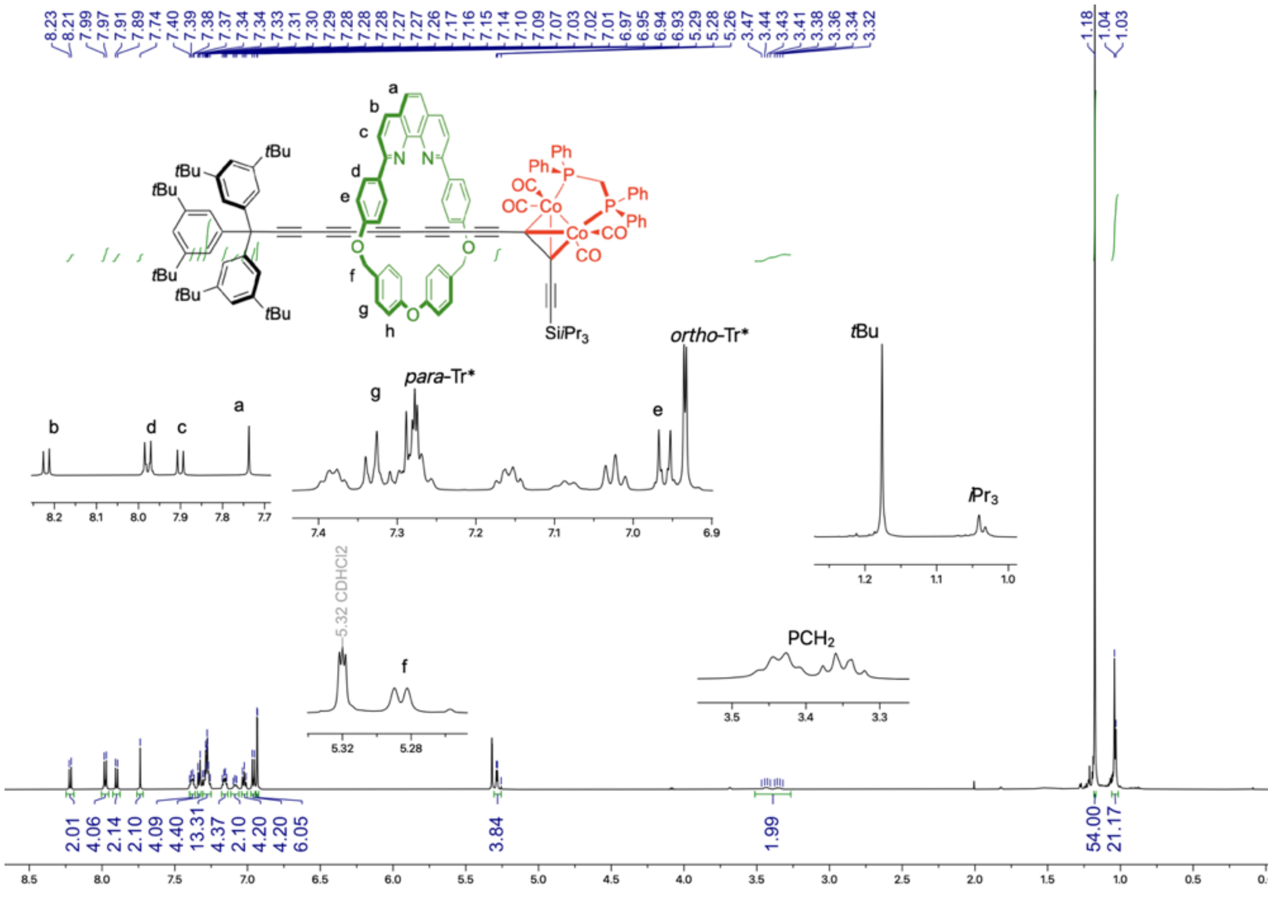

Figure 28. ^1H NMR (600 MHz) spectrum of compound **S3·M$_b$** in CD_2Cl_2.

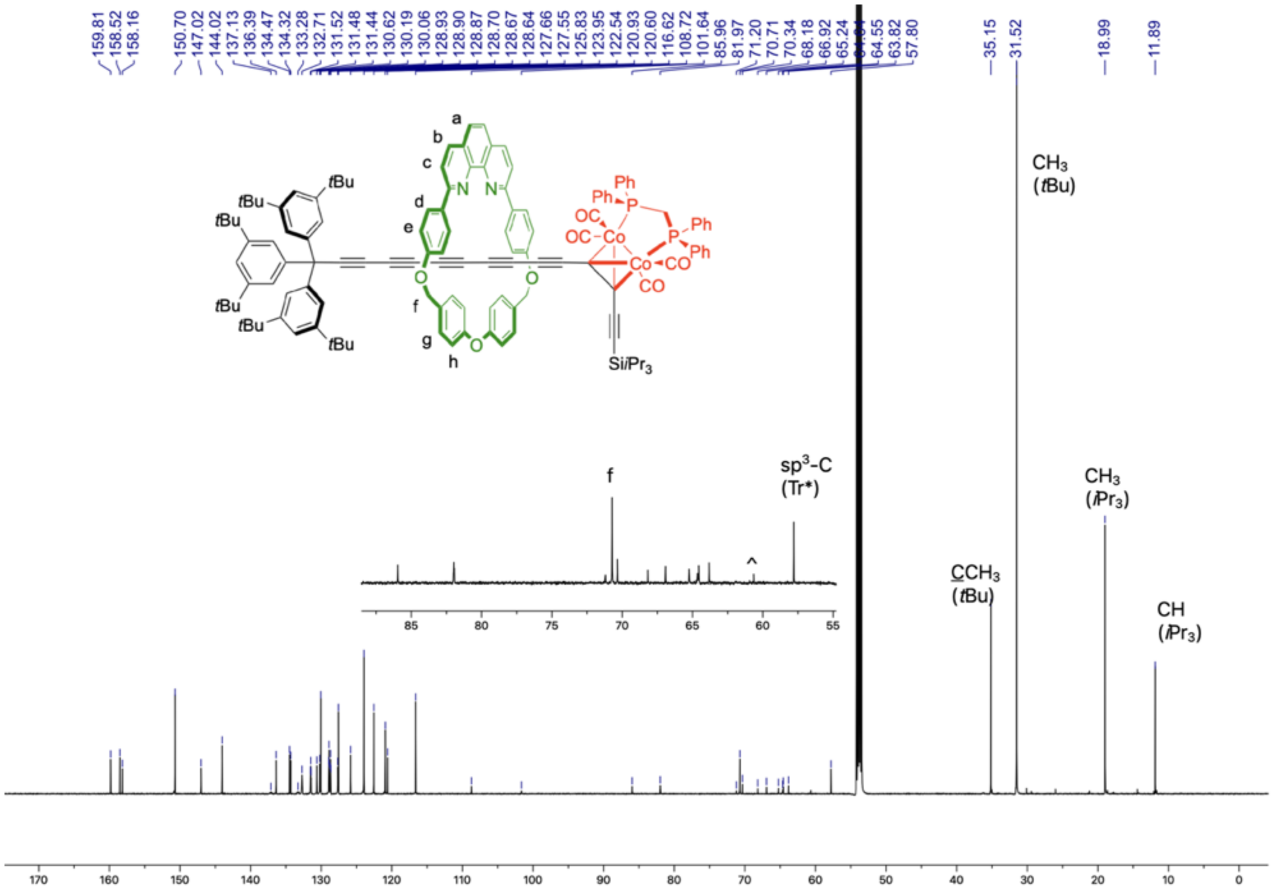

Figure 29. $^{13}C\{^{1}H\}$ NMR (151 MHz) spectrum of compound **S3·M$_b$** in CD_2Cl_2 (^indicates residual ethyl acetate).

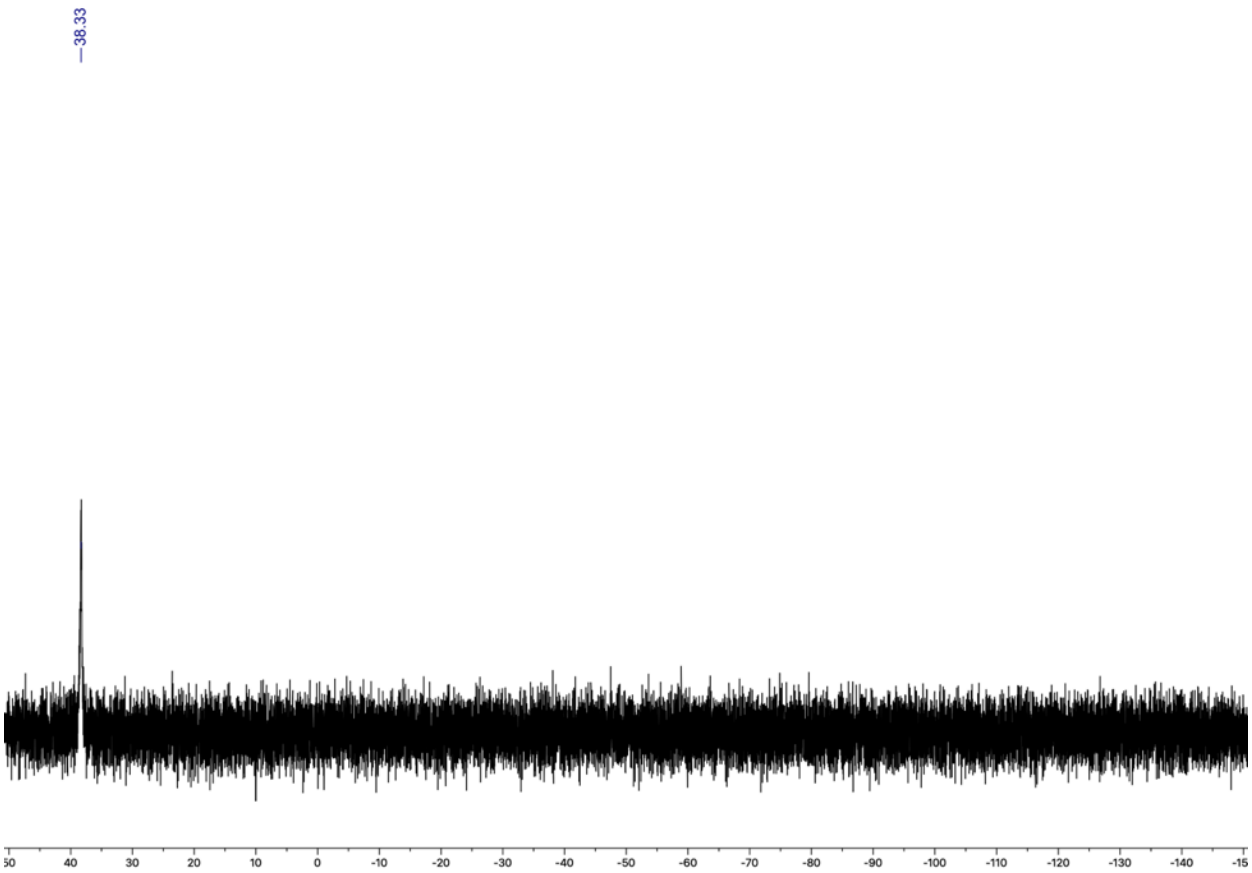

Figure 30. $^{31}P\{^{1}H\}$ NMR (202 MHz) spectrum of compound **S3·M$_b$** in CD_2Cl_2.

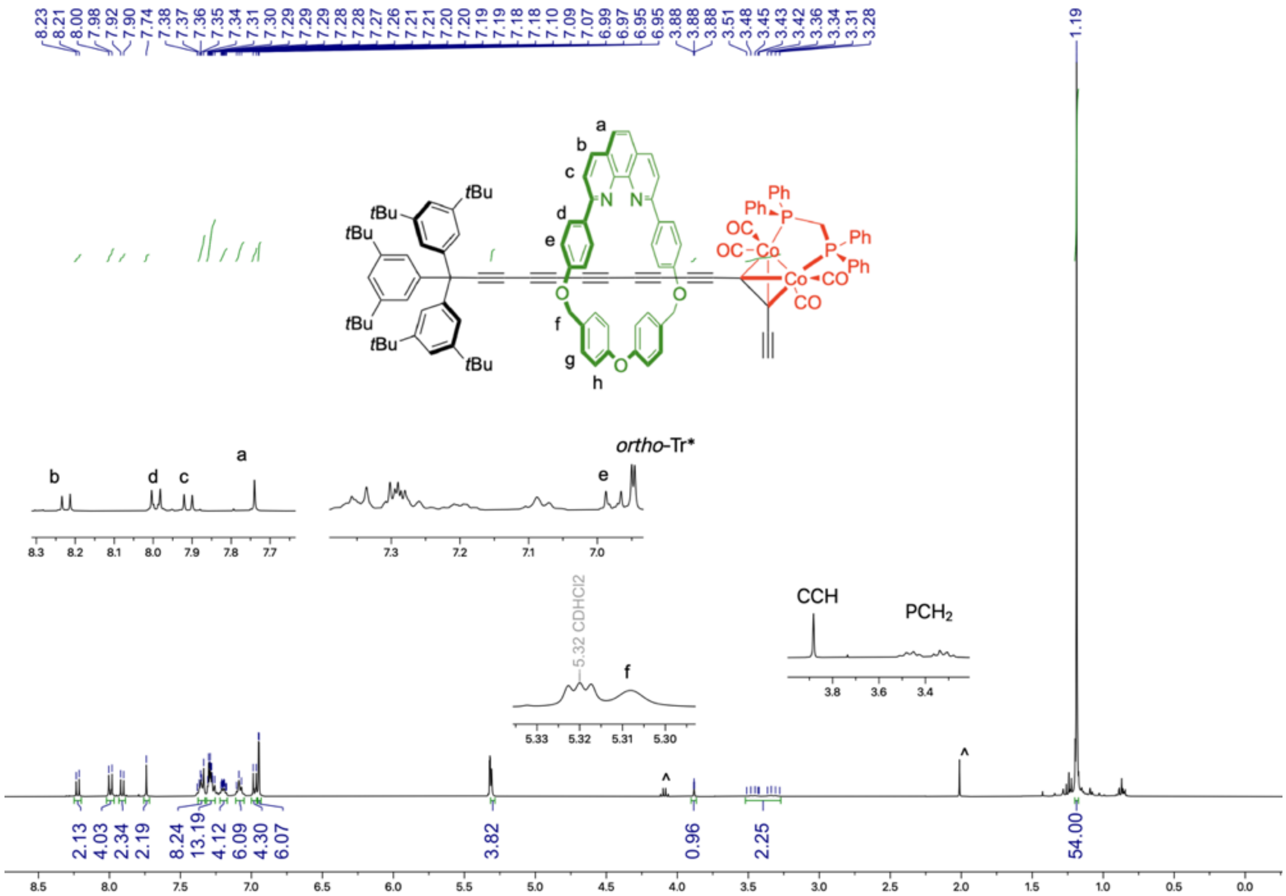

Figure 31. ^{1}H NMR (400 MHz) spectrum of compound **7·M$_b$** in CD_2Cl_2 (^indicates residual ethyl acetate).

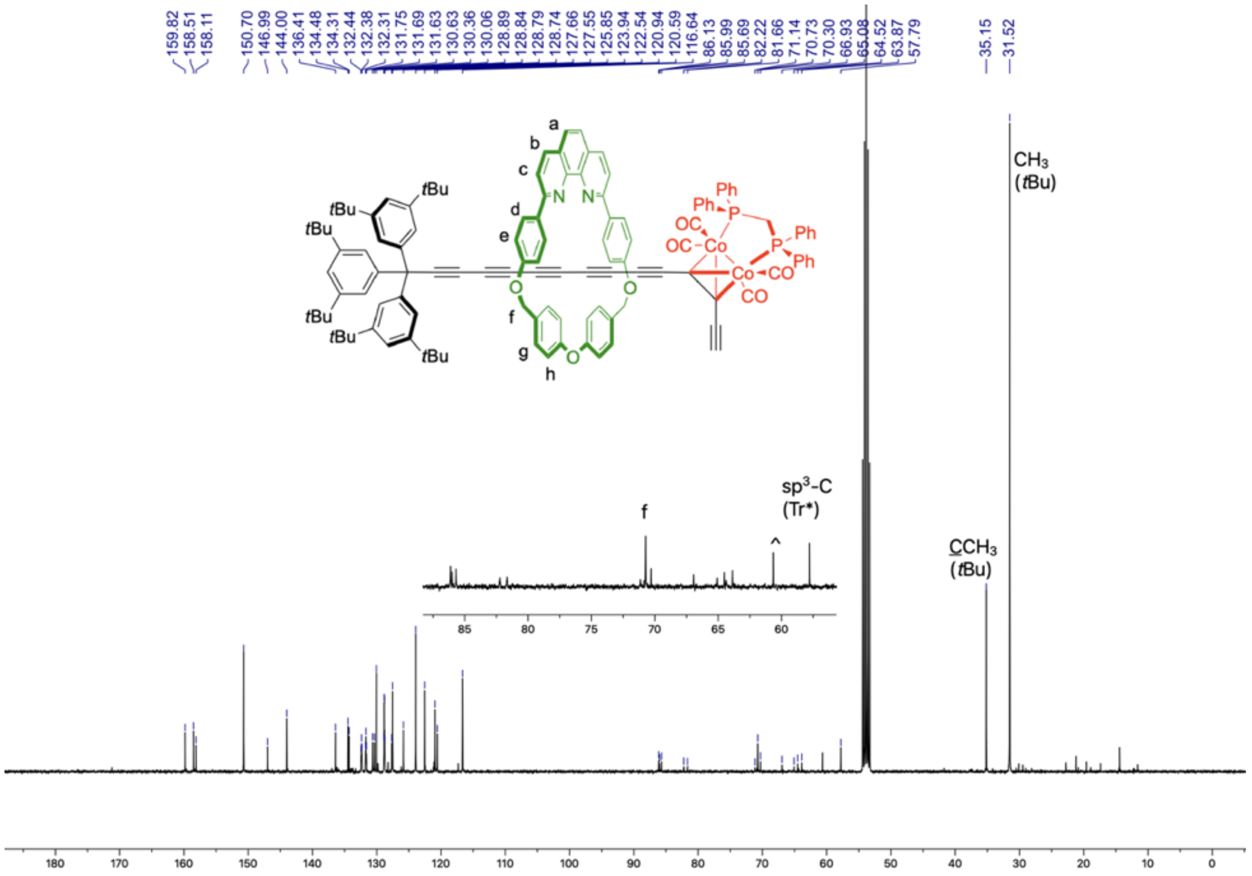

Figure 32. $^{13}C\{^{1}H\}$ NMR (100 MHz) spectrum of compound **7·M$_b$** in CD_2Cl_2 (^indicates residual ethyl acetate).

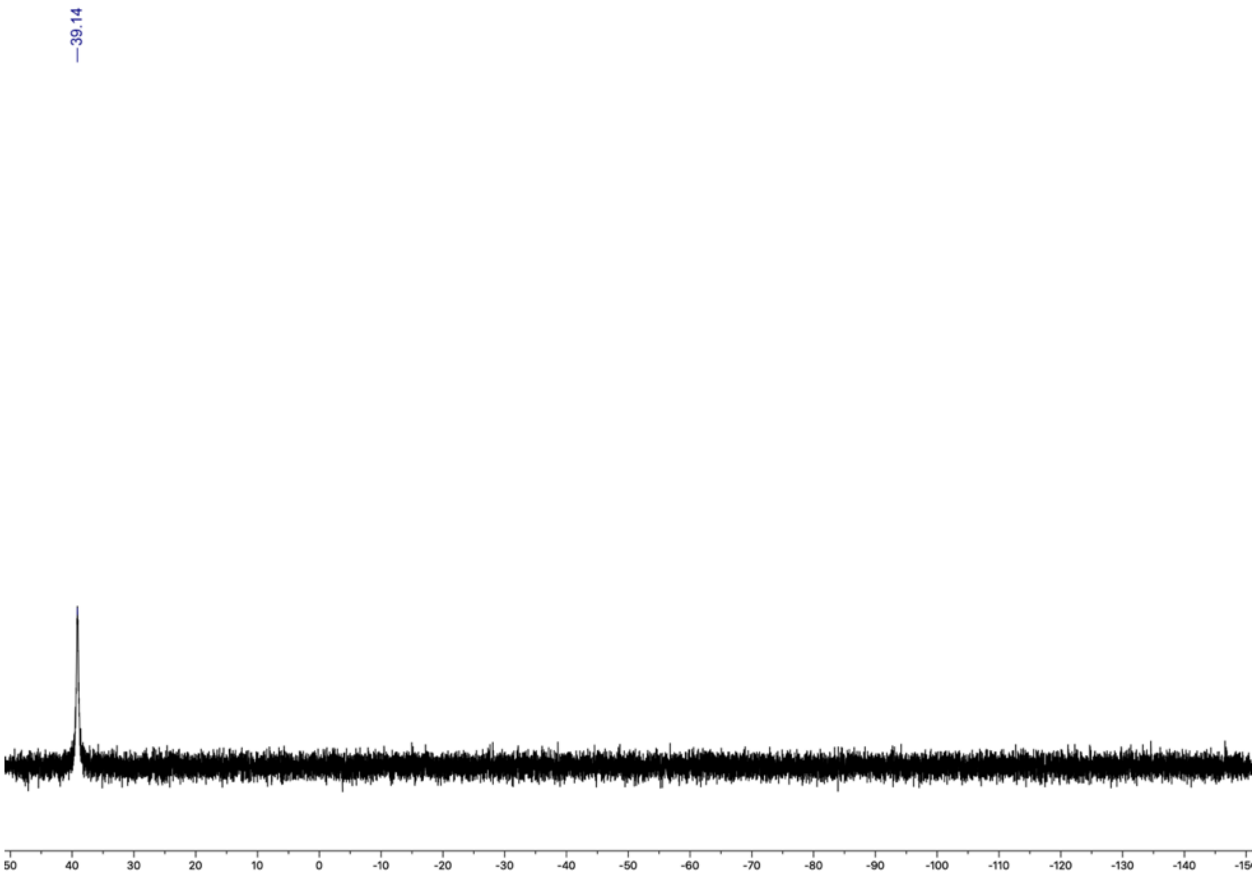

Figure 33. $^{31}P\{^{1}H\}$ NMR (162 MHz) spectrum of **7·M$_b$** in CD_2Cl_2.

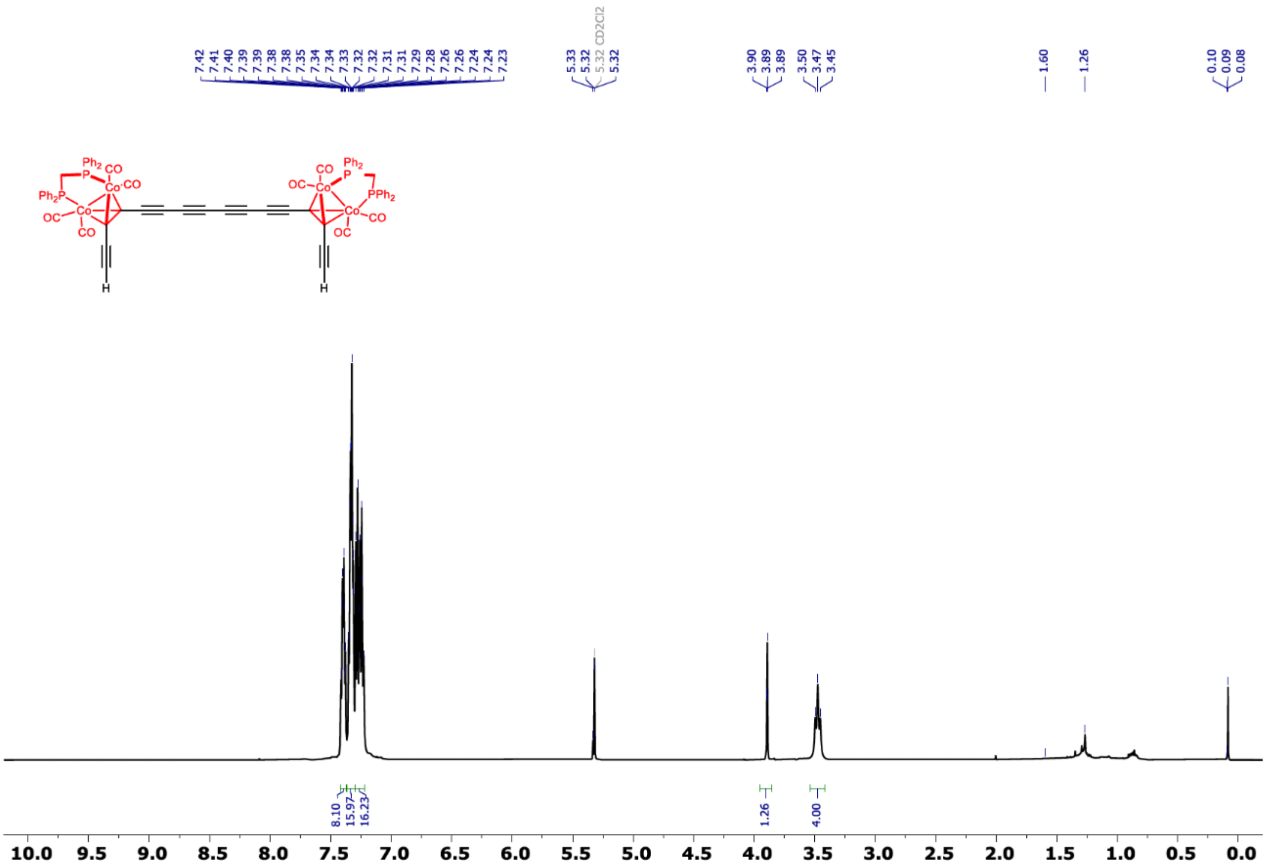

Figure 34. ^{1}H NMR (500 MHz) spectrum of compound **4** in CD_2Cl_2.

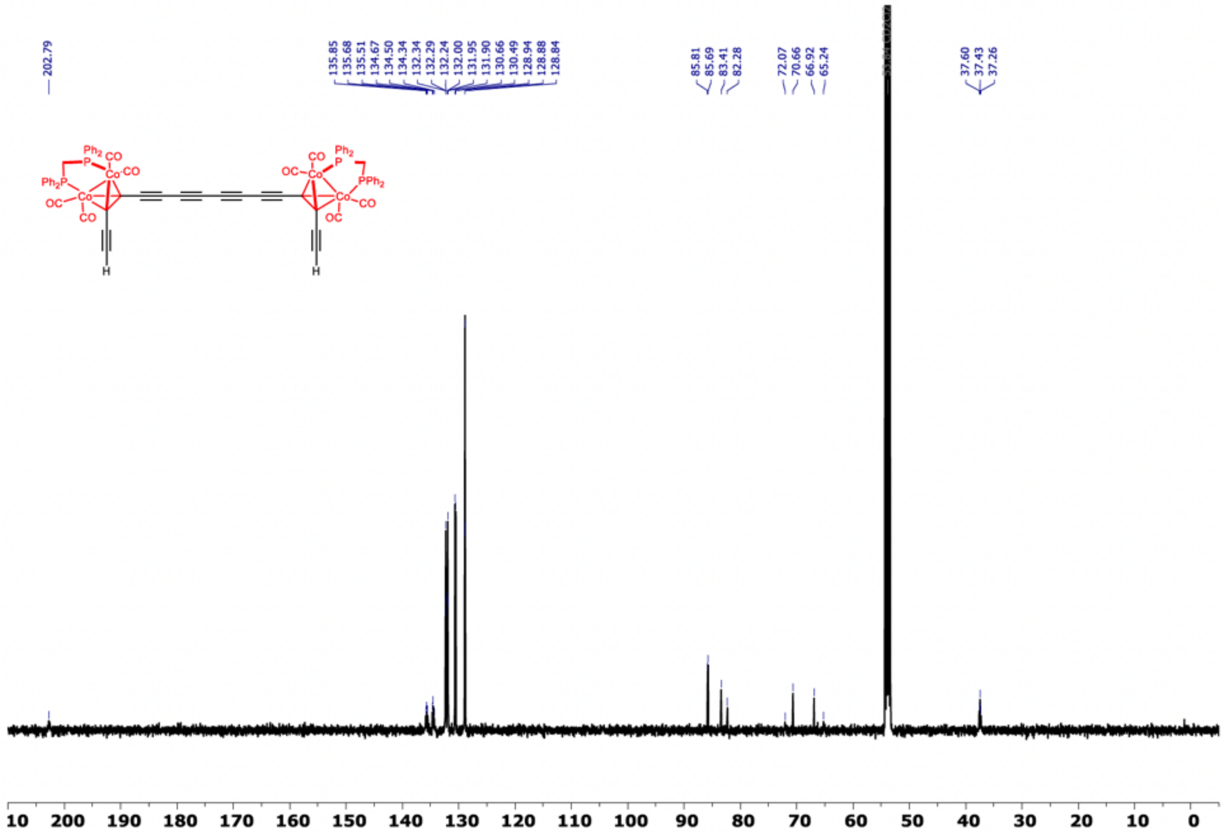

Figure 35. $^{13}C\{^{1}H\}$ NMR (126 MHz) spectrum of compound **4** in CD_2Cl_2.

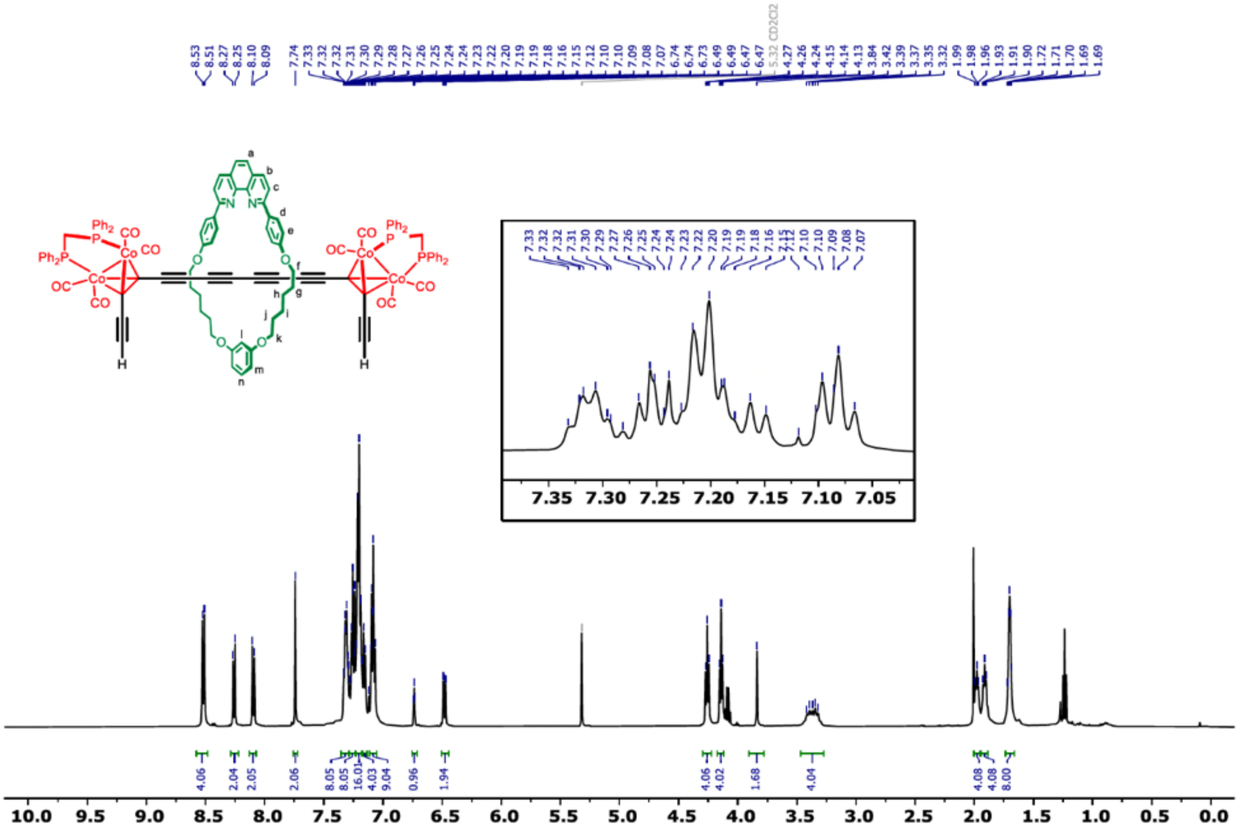

Figure 36. ^{1}H NMR (500 MHz) spectrum of compound $\mathbf{4{\cdot}M_a}$ in CD_2Cl_2.

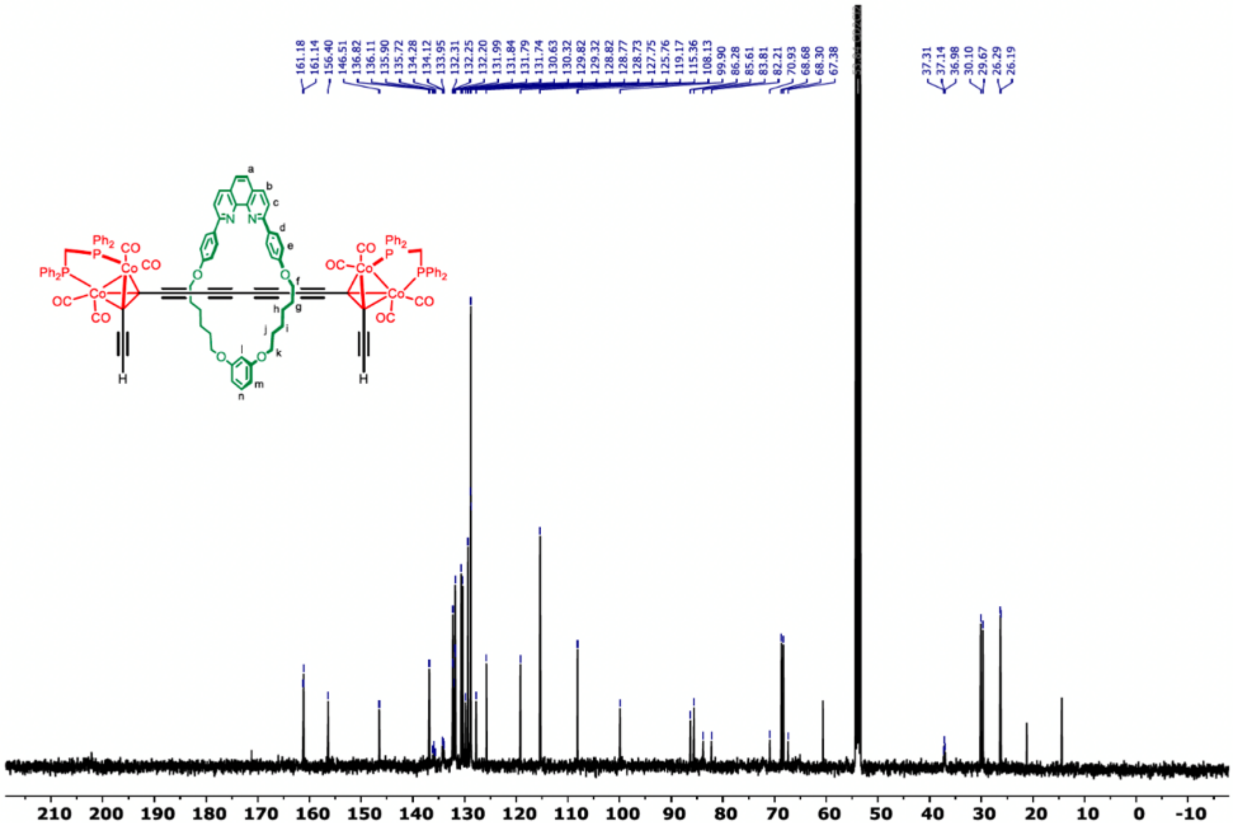

Figure 37. $^{13}C\{^{1}H\}$ NMR (126 MHz) spectrum of compound **4·M**

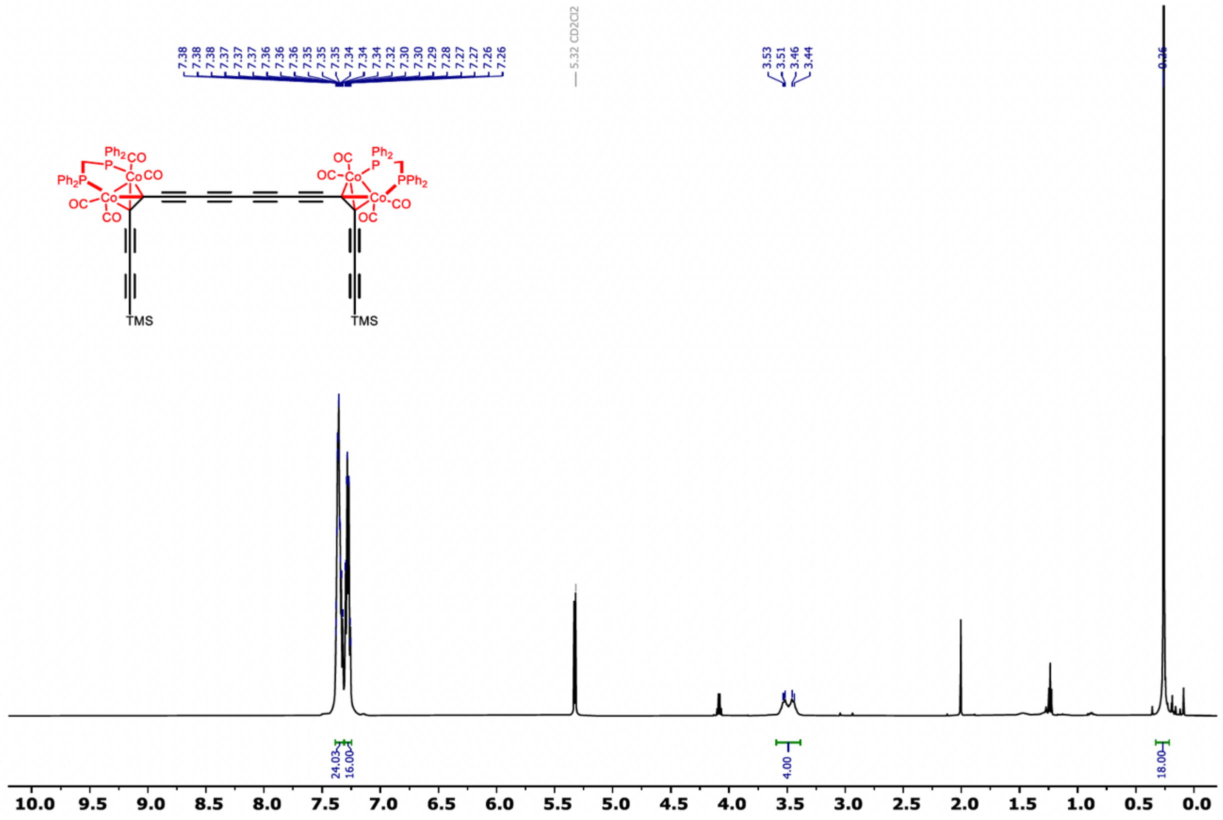

Figure 38. ^{1}H NMR (600 MHz) spectrum of compound **5** in CD_2Cl_2.

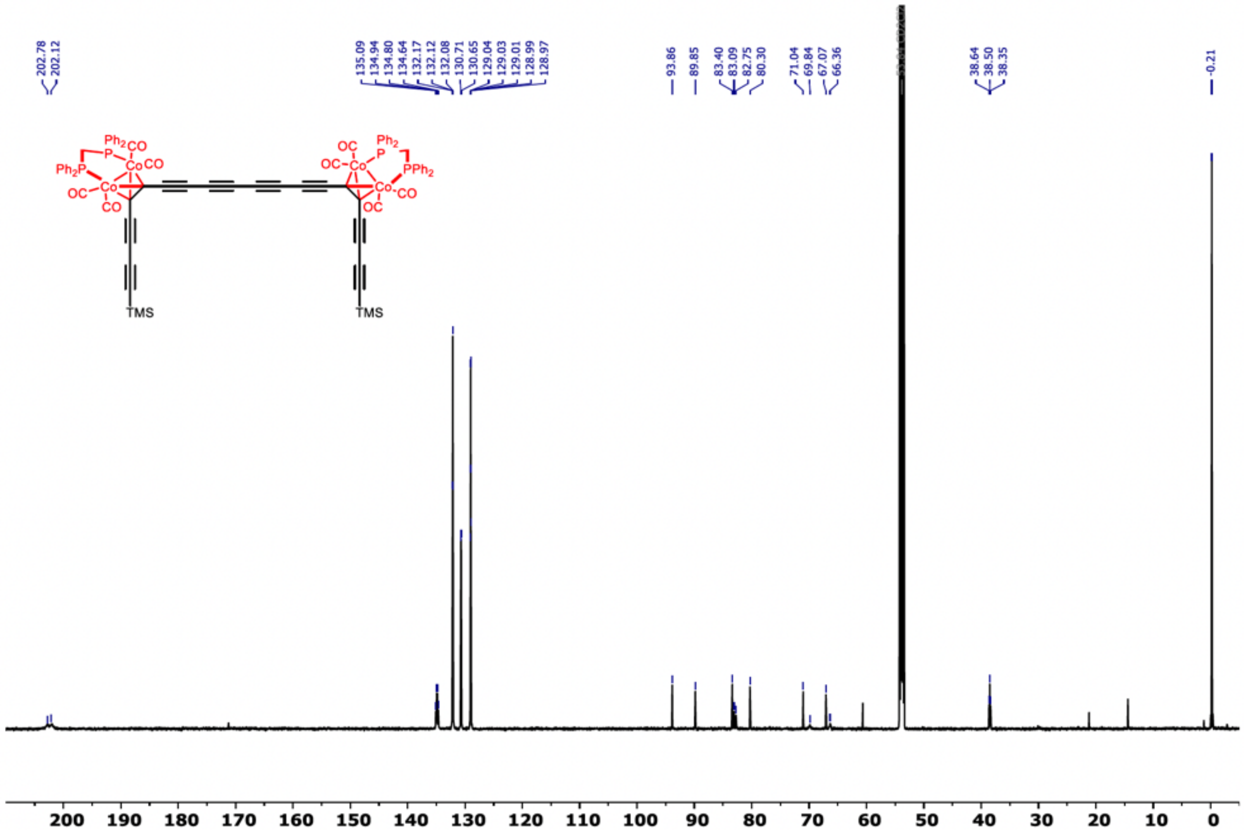

Figure 39. $^{13}C\{^{1}H\}$ NMR (151 MHz) spectrum of compound **5** in CD_2Cl_2.

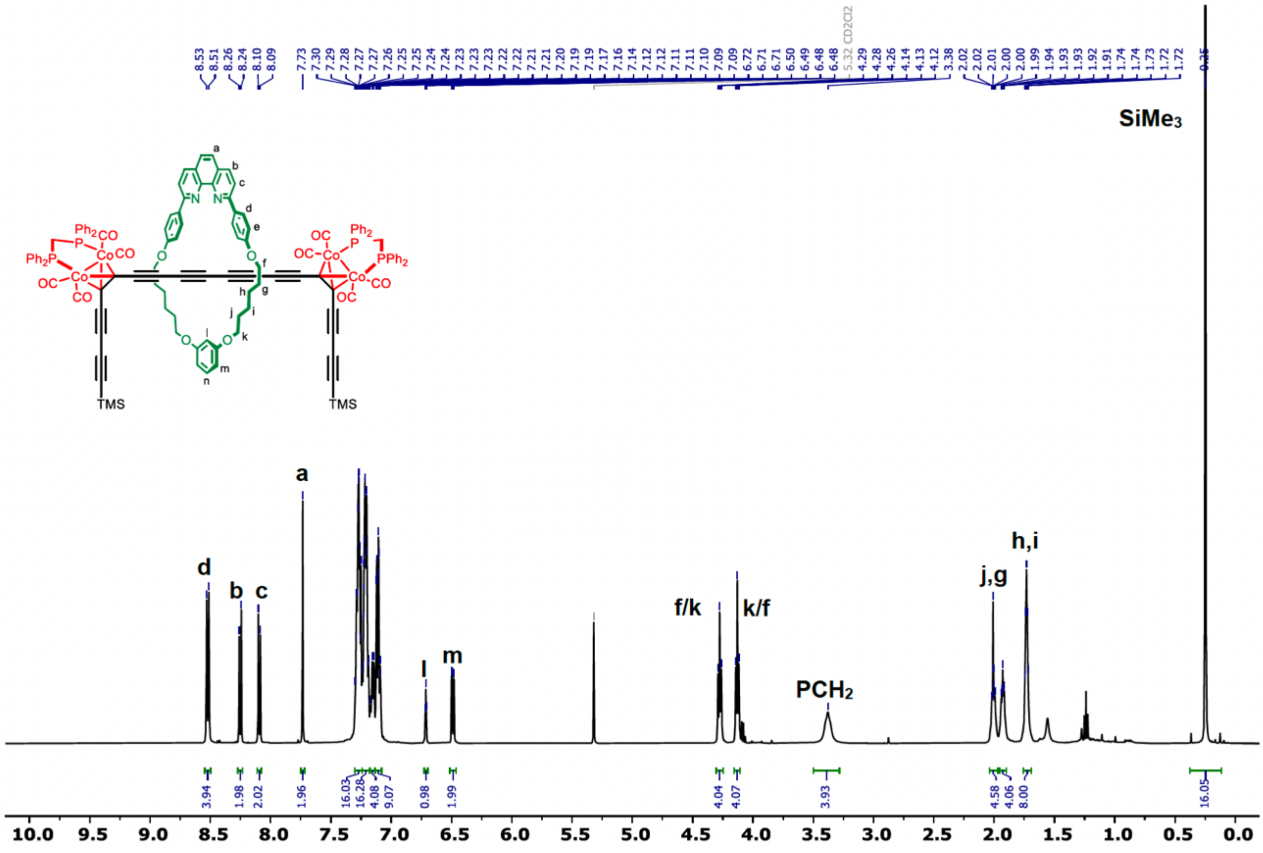

Figure 40. ^{1}H NMR (500 MHz) spectrum of compound $\mathbf{5{\cdot}M_a}$ in CD_2Cl_2.

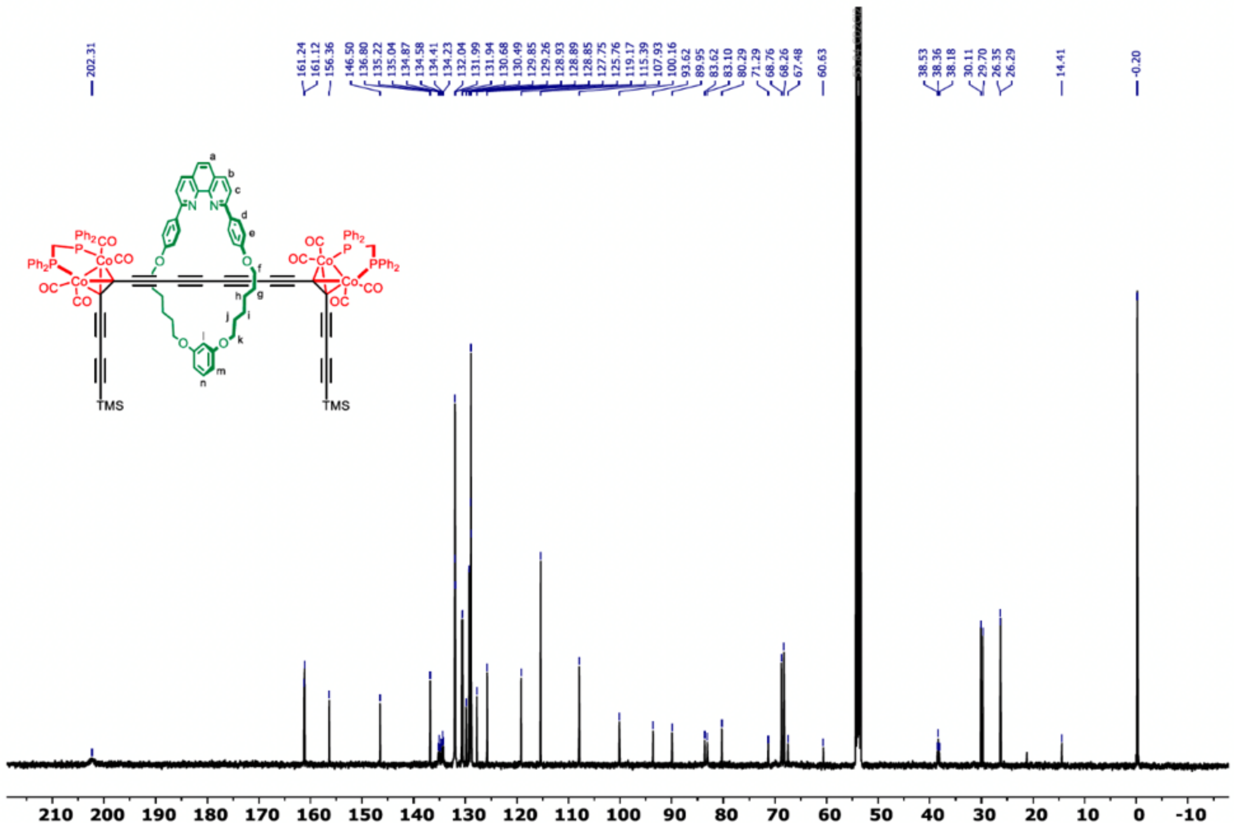

Figure 41. $^{13}C\{^{1}H\}$ NMR (126 MHz) spectrum of compound **5·M$_a$** in CD_2Cl_2.

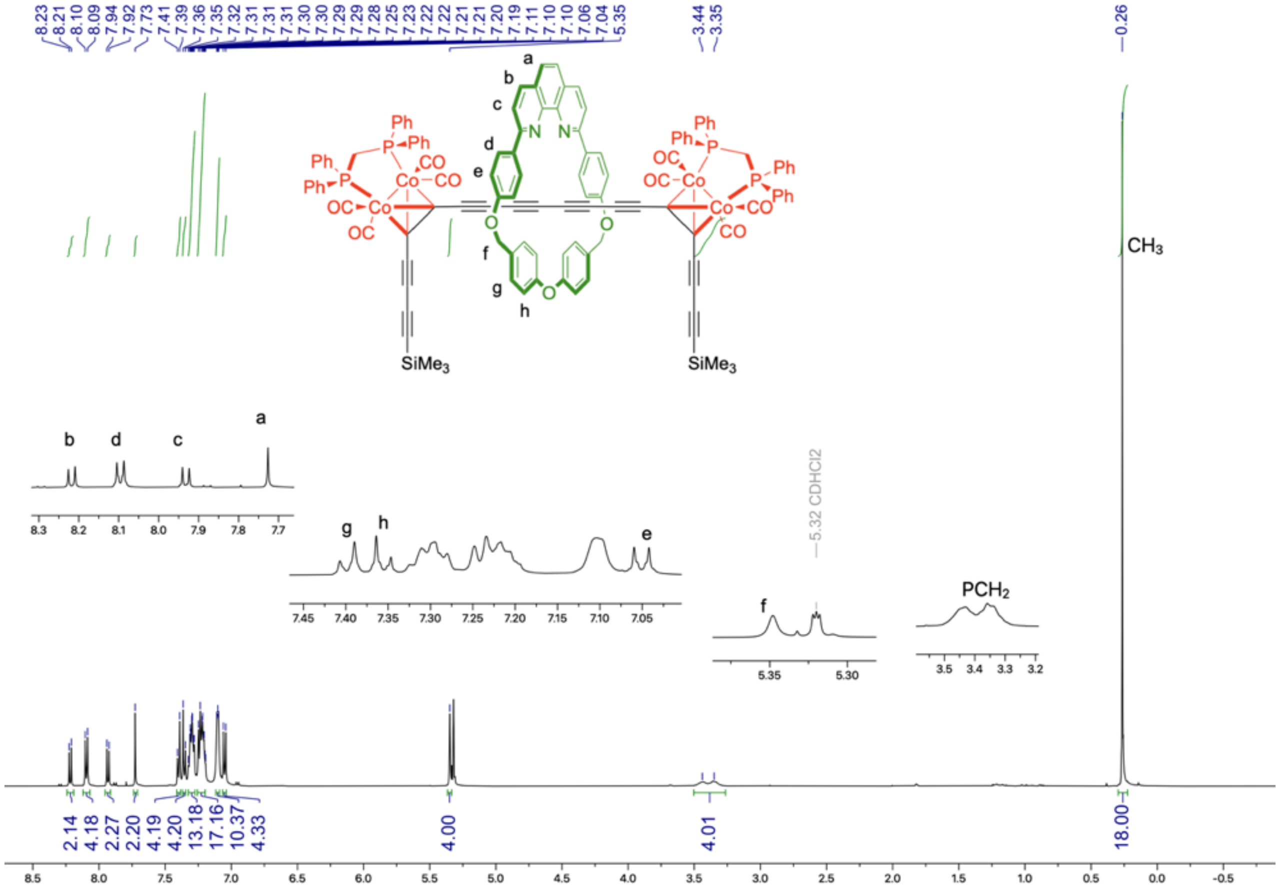

Figure 42. ^{1}H NMR (500 MHz) spectrum of compound **5·M$_b$** in CD_2Cl_2.

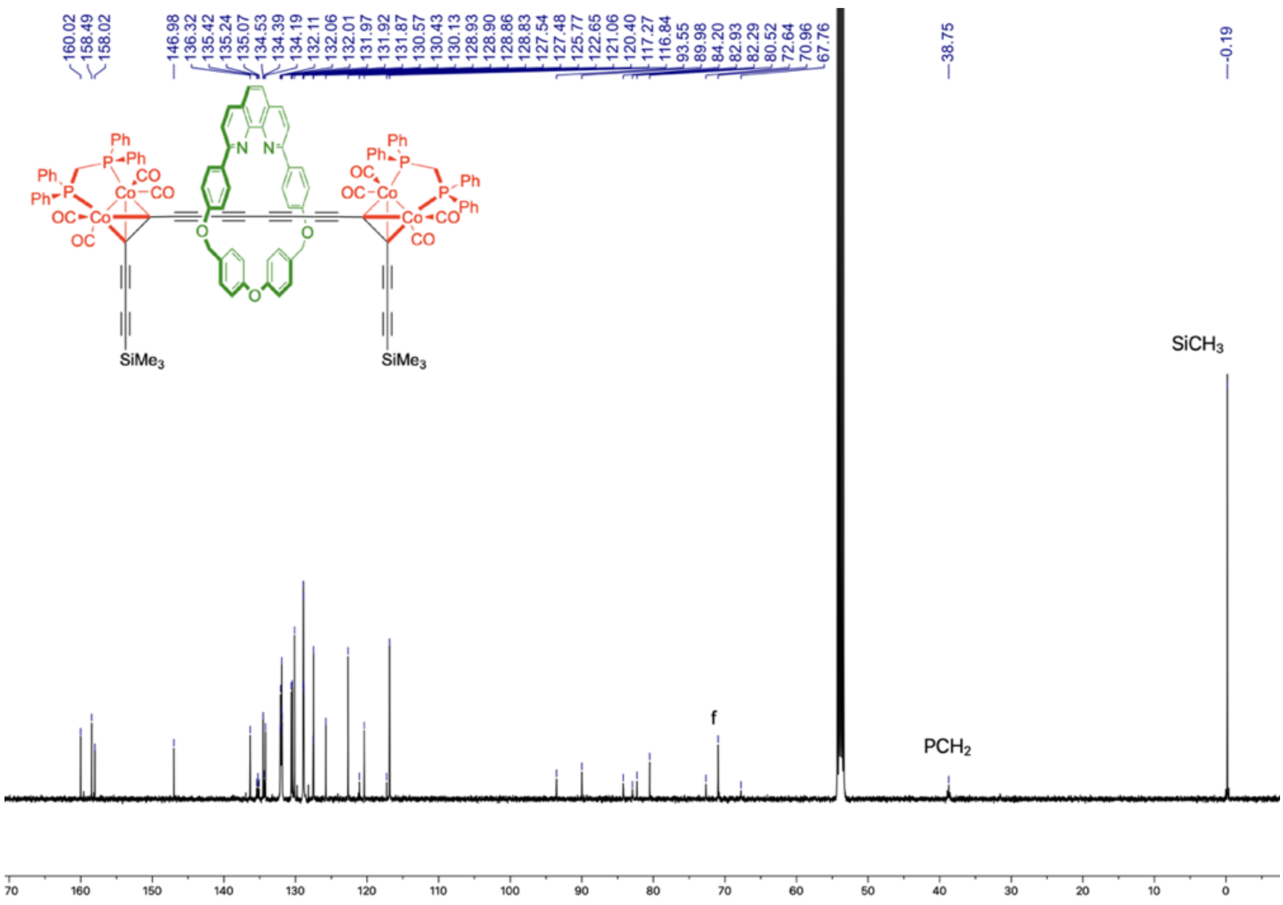

Figure 43. $^{13}C\{^{1}H\}$ NMR (126 MHz) spectrum of compound **5·M$_b$** in CD_2Cl_2.

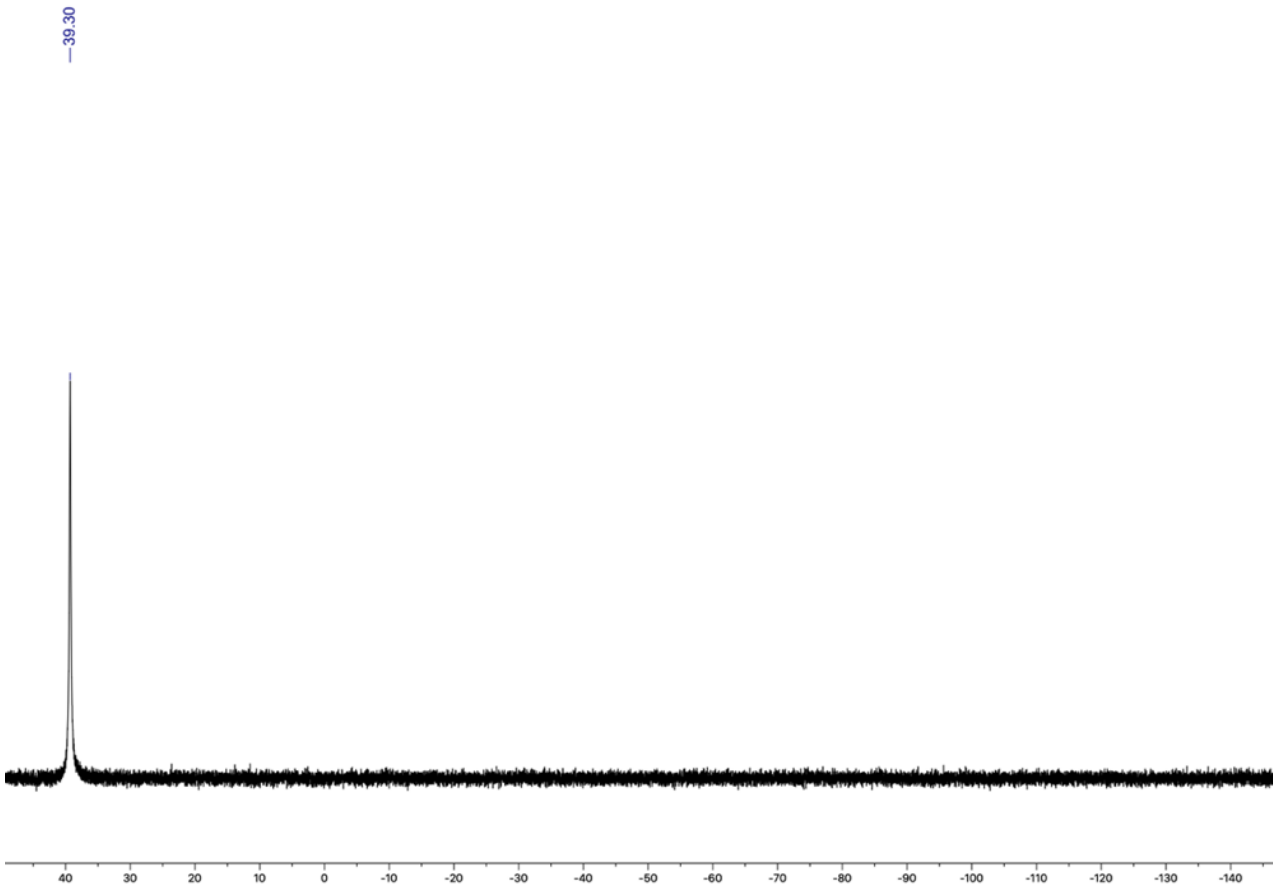

Figure 44. $^{31}P\{^{1}H\}$ NMR (202 MHz) spectrum of compound **5·M$_b$** in CD_2Cl_2.

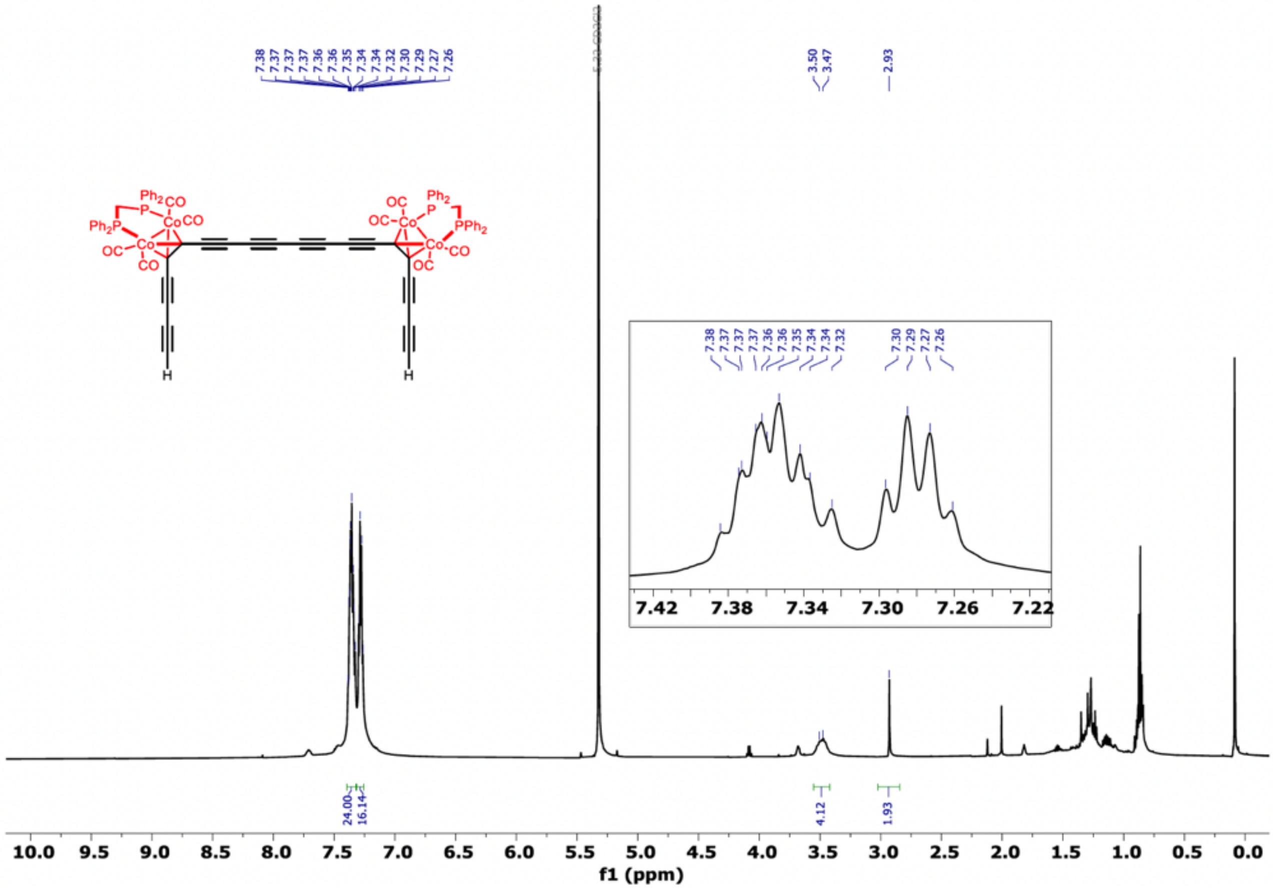

Figure 45. ^{1}H NMR (600 MHz) spectrum of compound **6** in CD_2Cl_2.

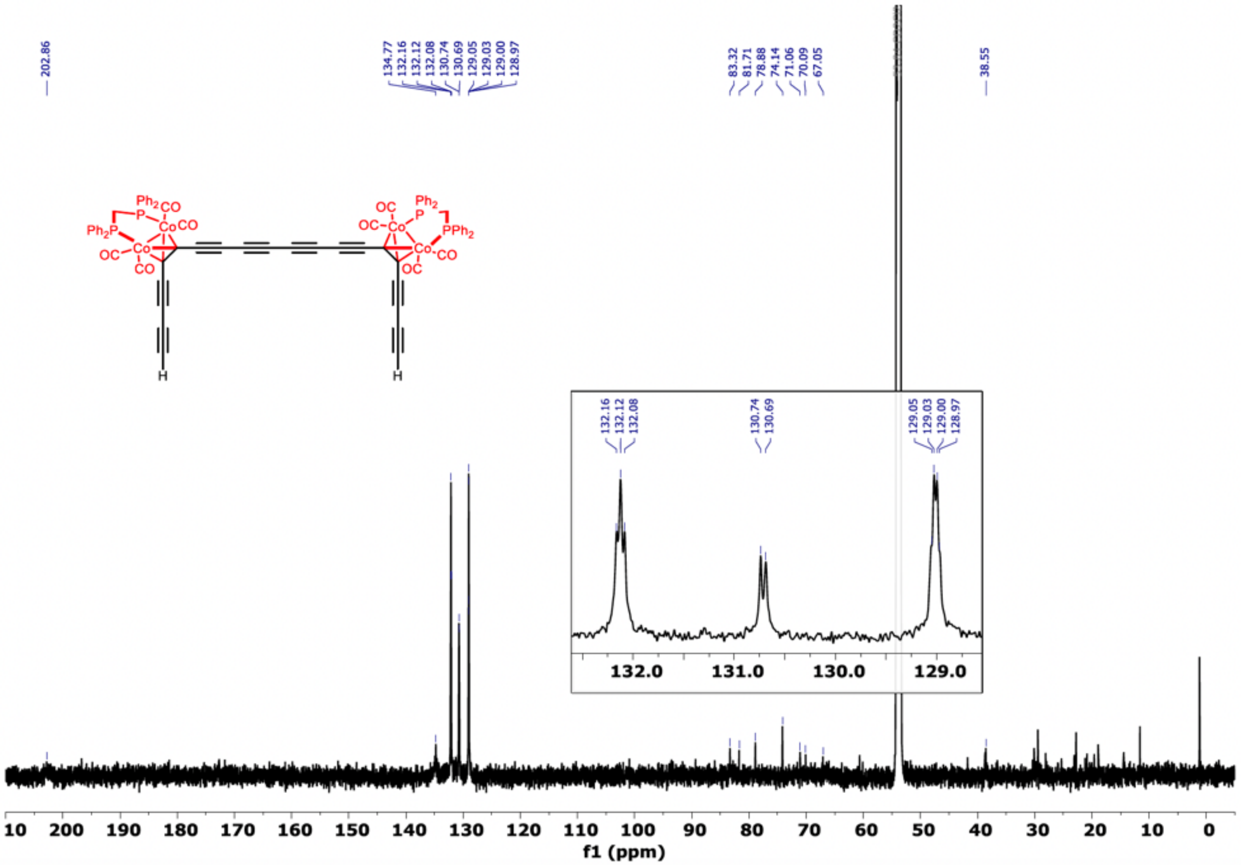

Figure 46. $^{13}C\{^{1}H\}$ NMR (151 MHz) spectrum of compound **6** in CD_2Cl_2.

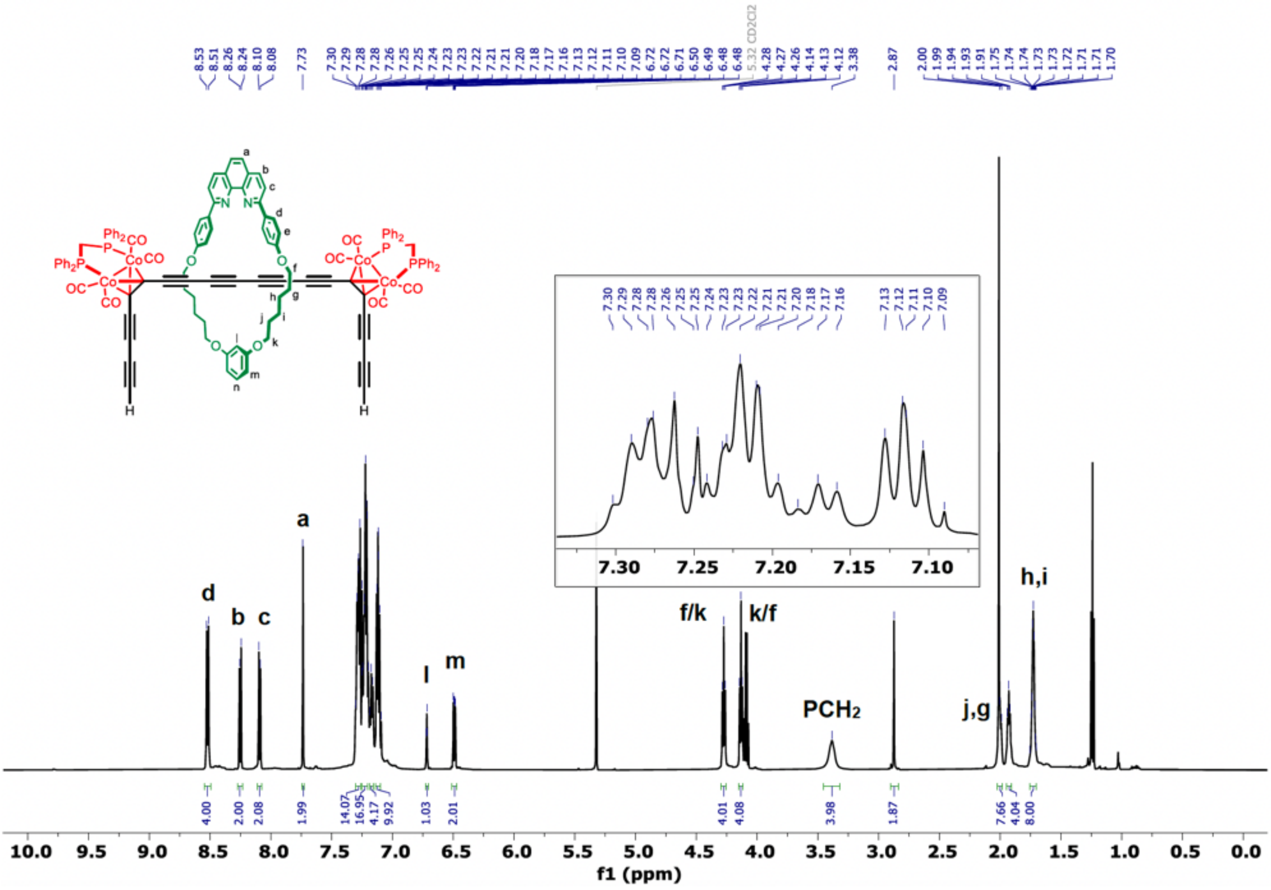

Figure 47. ^{1}H NMR (500 MHz) spectrum of compound **6·M$_a$** in CD_2Cl_2.

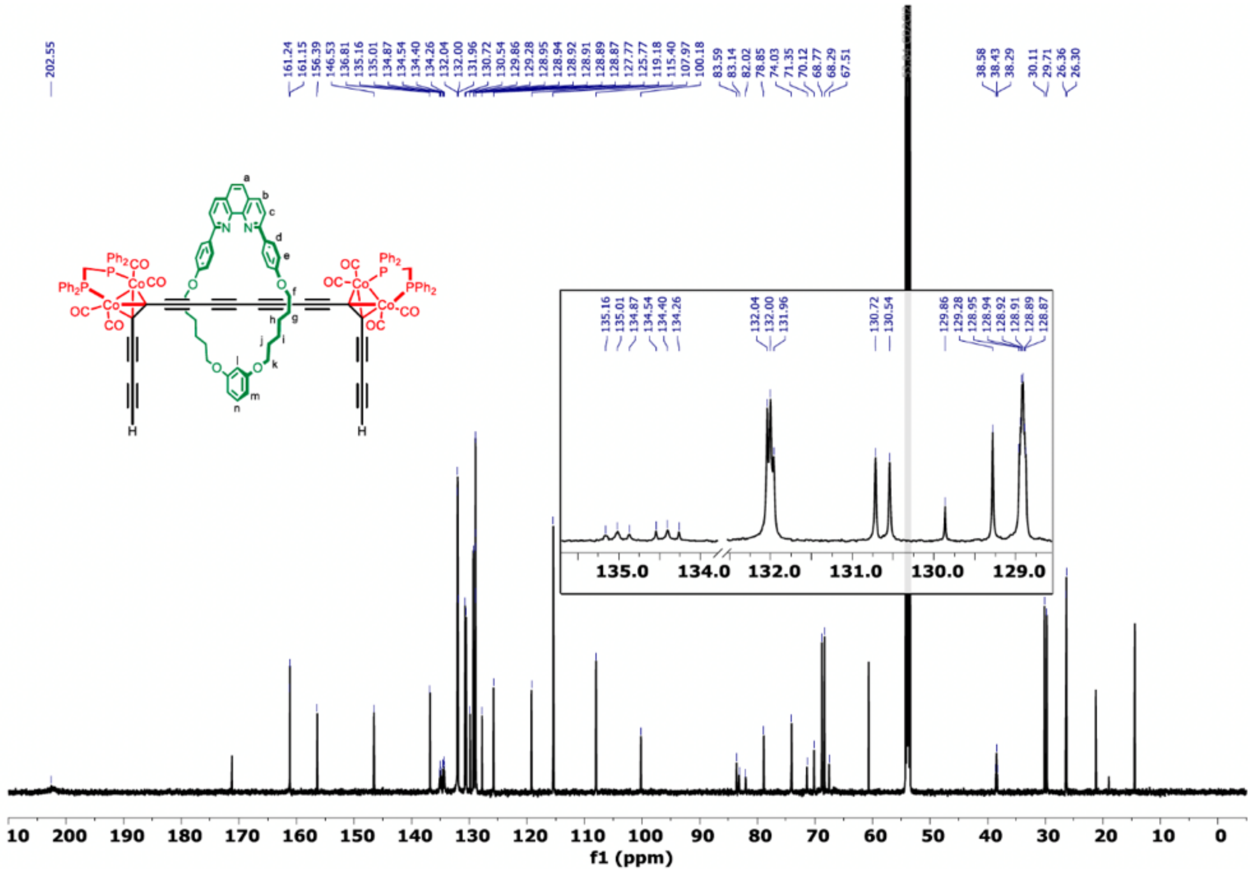

Figure 48. $^{13}C\{^{1}H\}$ NMR (126 MHz) spectrum of compound **6·M$_a$** in CD_2Cl_2.

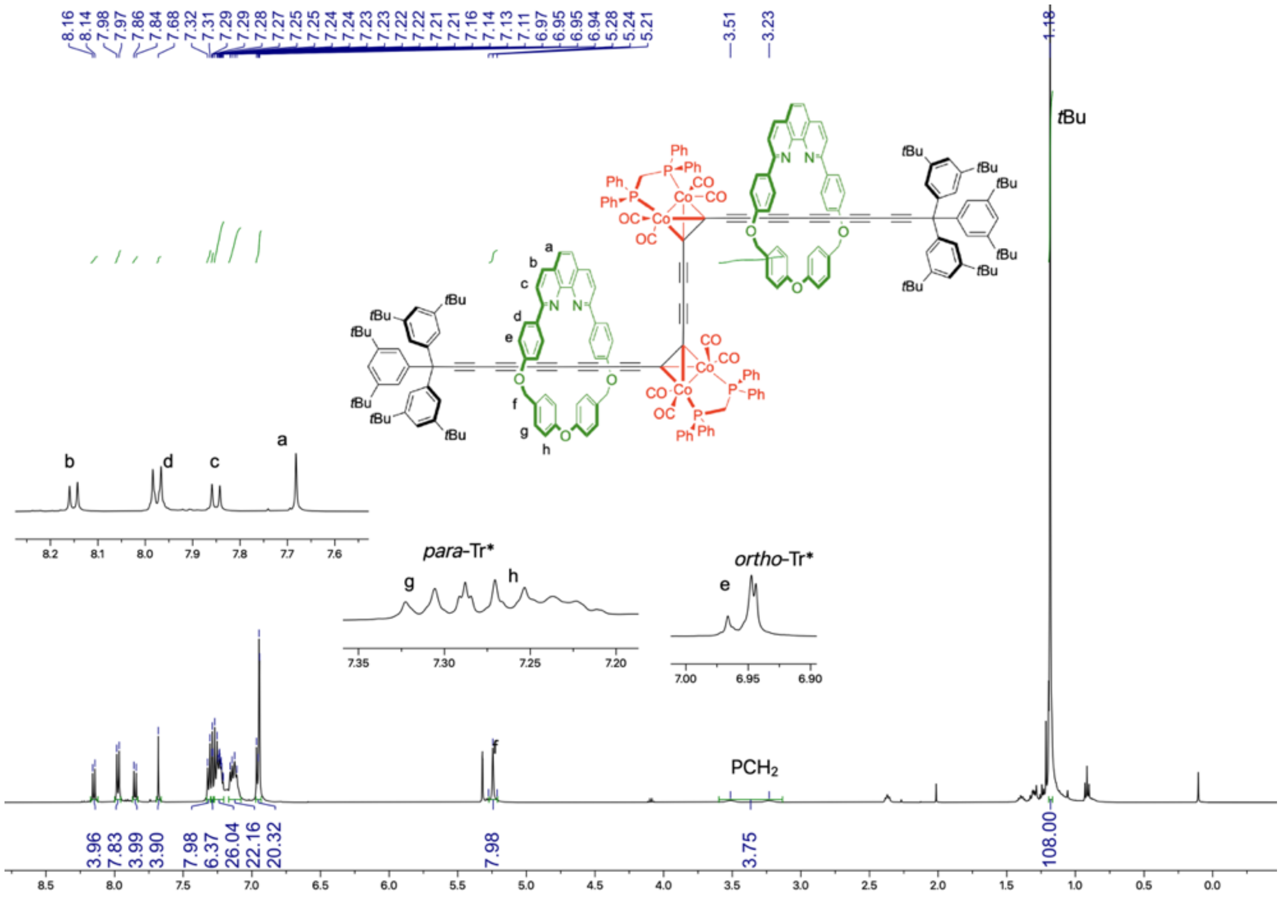

Figure 49. ^{1}H NMR (500 MHz) spectrum of compound ***m*C28·(M$_b$)$_2$** in CD_2Cl_2.

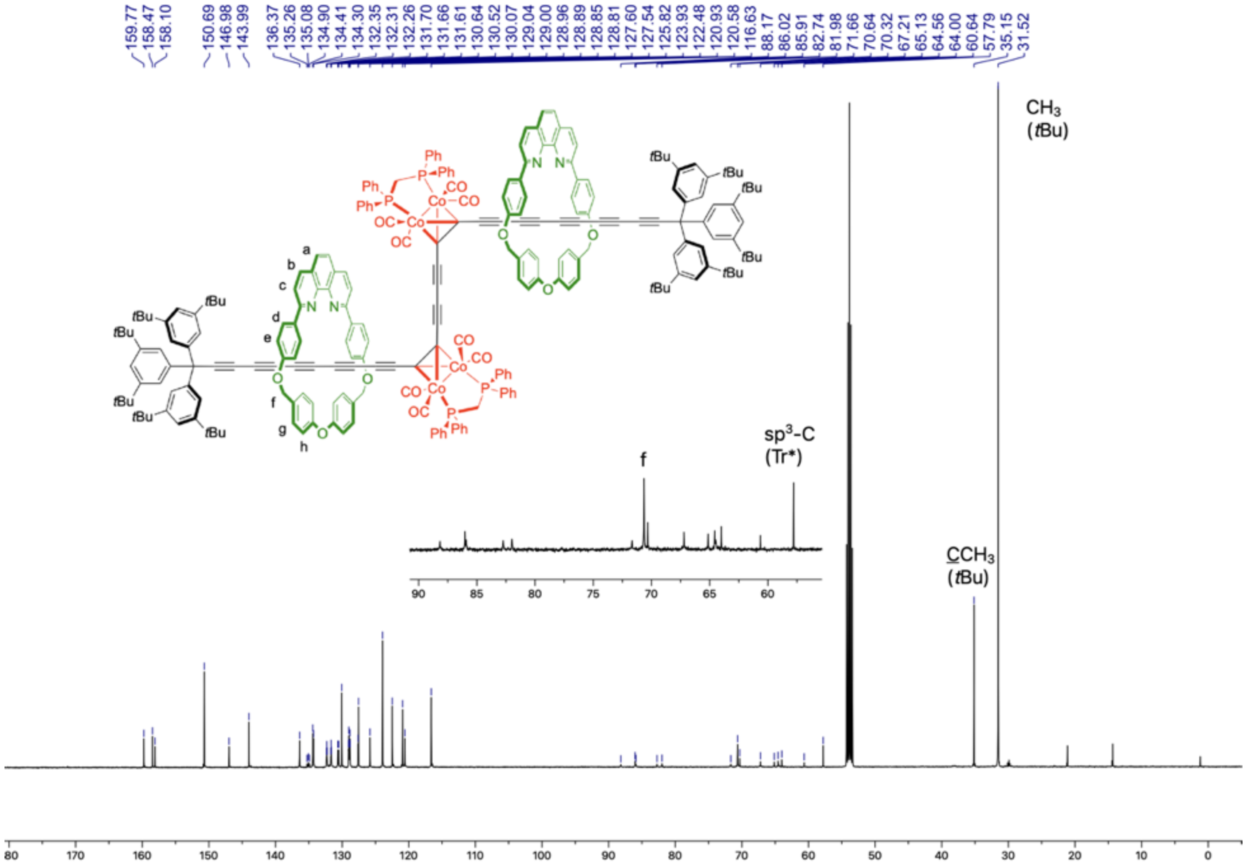

Figure 50. ^{13}C{^{1}H} NMR (126 MHz) spectrum of compound ***m*C28·(M$_b$)$_2$** in CD_2Cl_2.

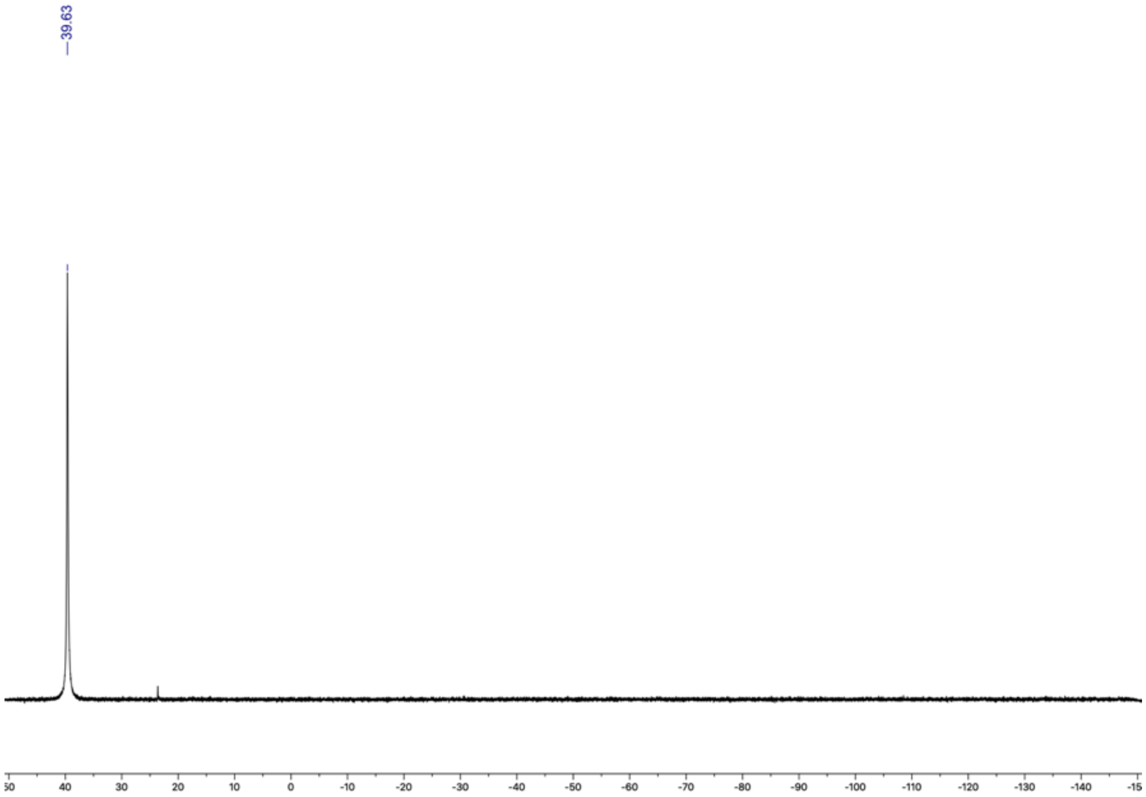

Figure 51. $^{31}P\{^{1}H\}$ NMR (202 MHz) spectrum of compound ***m*C28·(M$_b$)$_2$** in CD_2Cl_2.

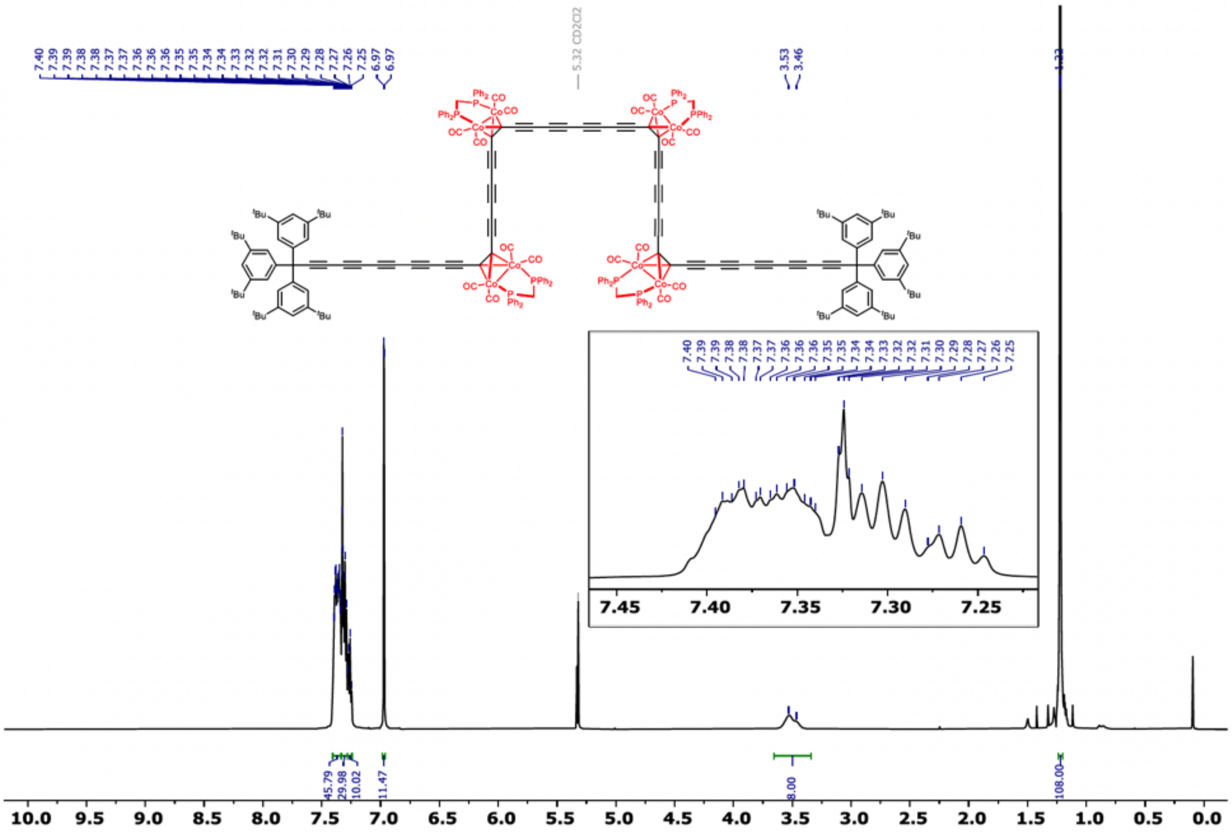

Figure 52. ^{1}H NMR (600 MHz) spectrum of compound ***m*C48** in CD_2Cl_2.

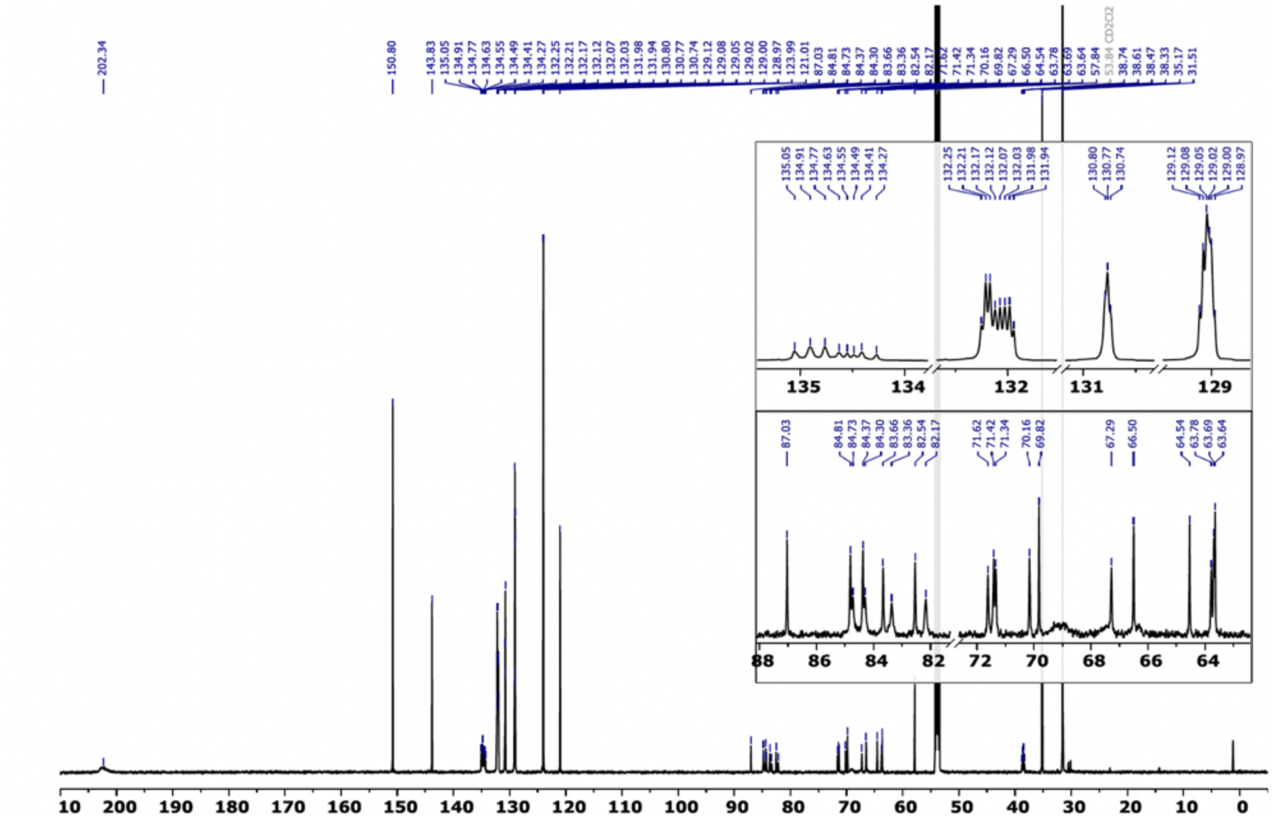

Figure 53. $^{13}C\{^{1}H\}$ NMR (151 MHz) spectrum of compound ***m*C48** in CD_2Cl_2.

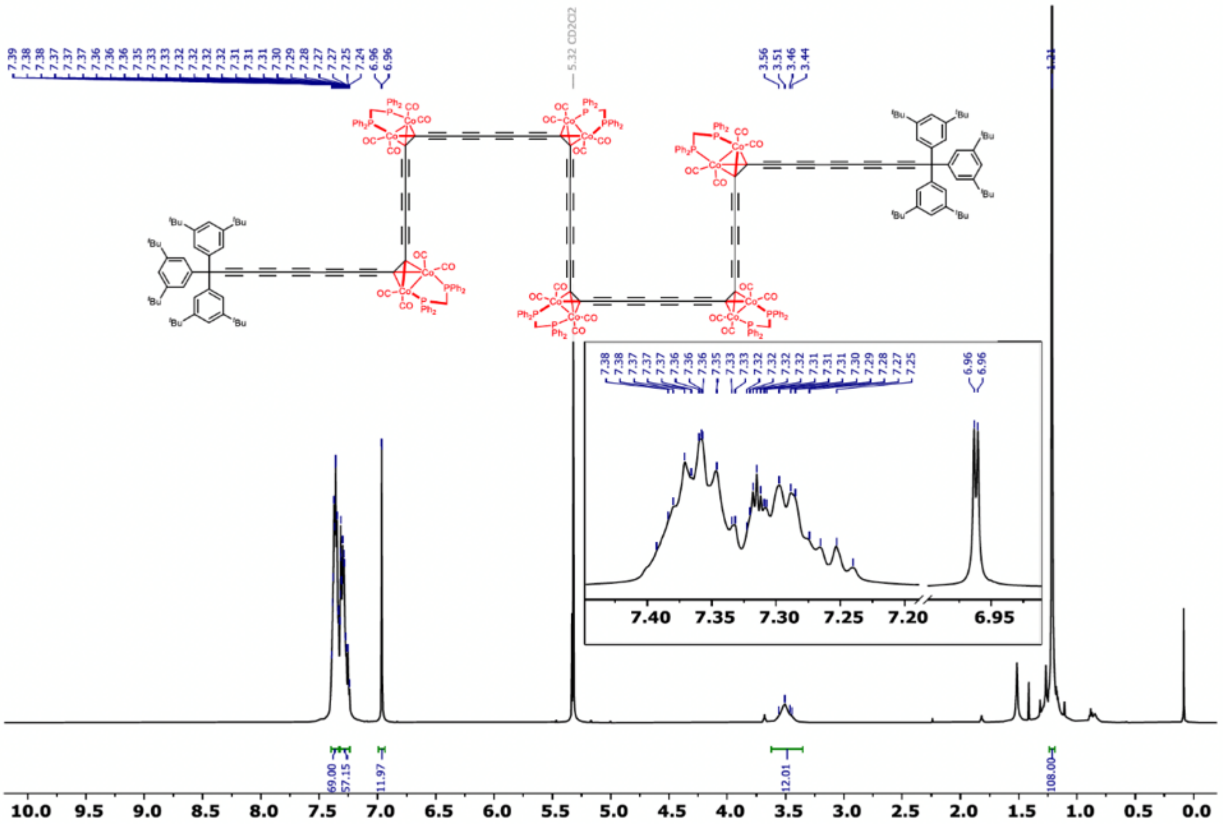

Figure 54. ^{1}H NMR (600 MHz) spectrum of compound ***m*C68** in CD_2Cl_2.

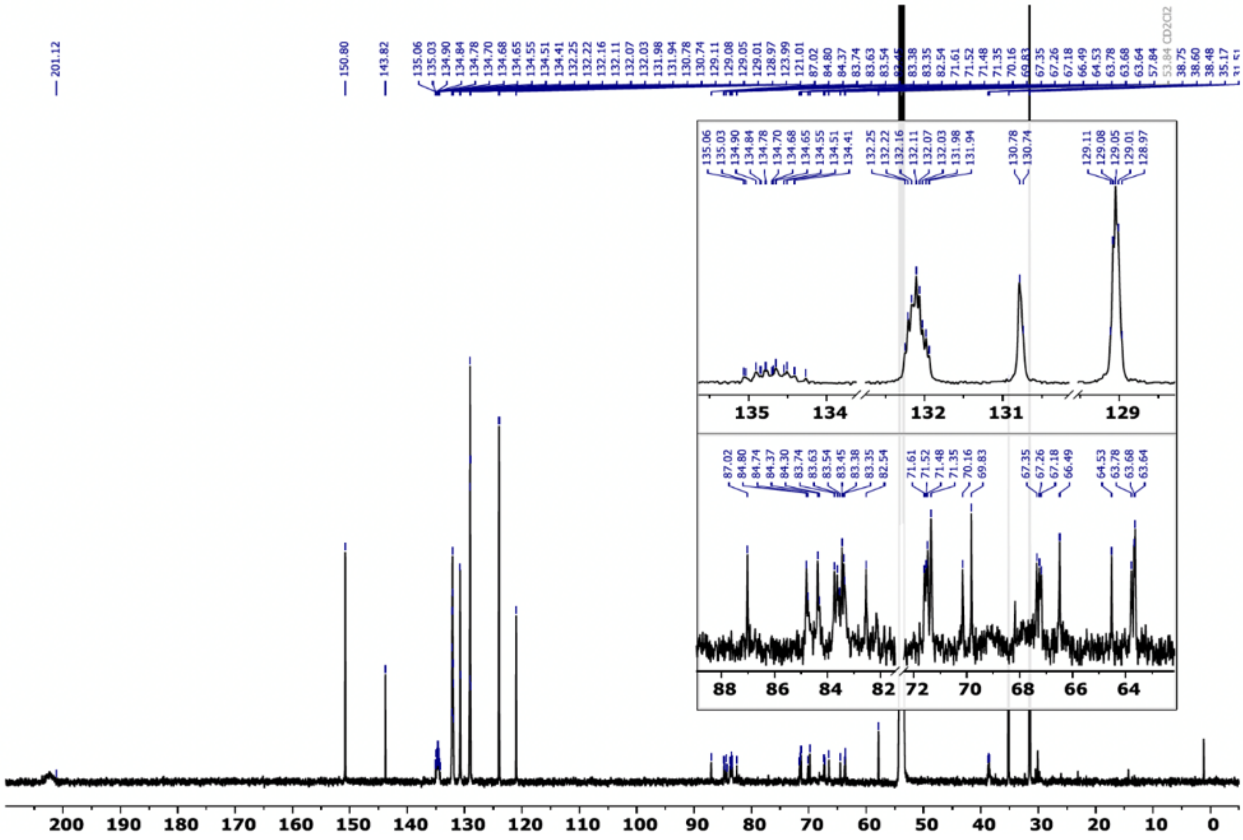

Figure 55. $^{13}C\{^{1}H\}$ NMR (151 MHz) spectrum of compound ***m*C68** in CD_2Cl_2.

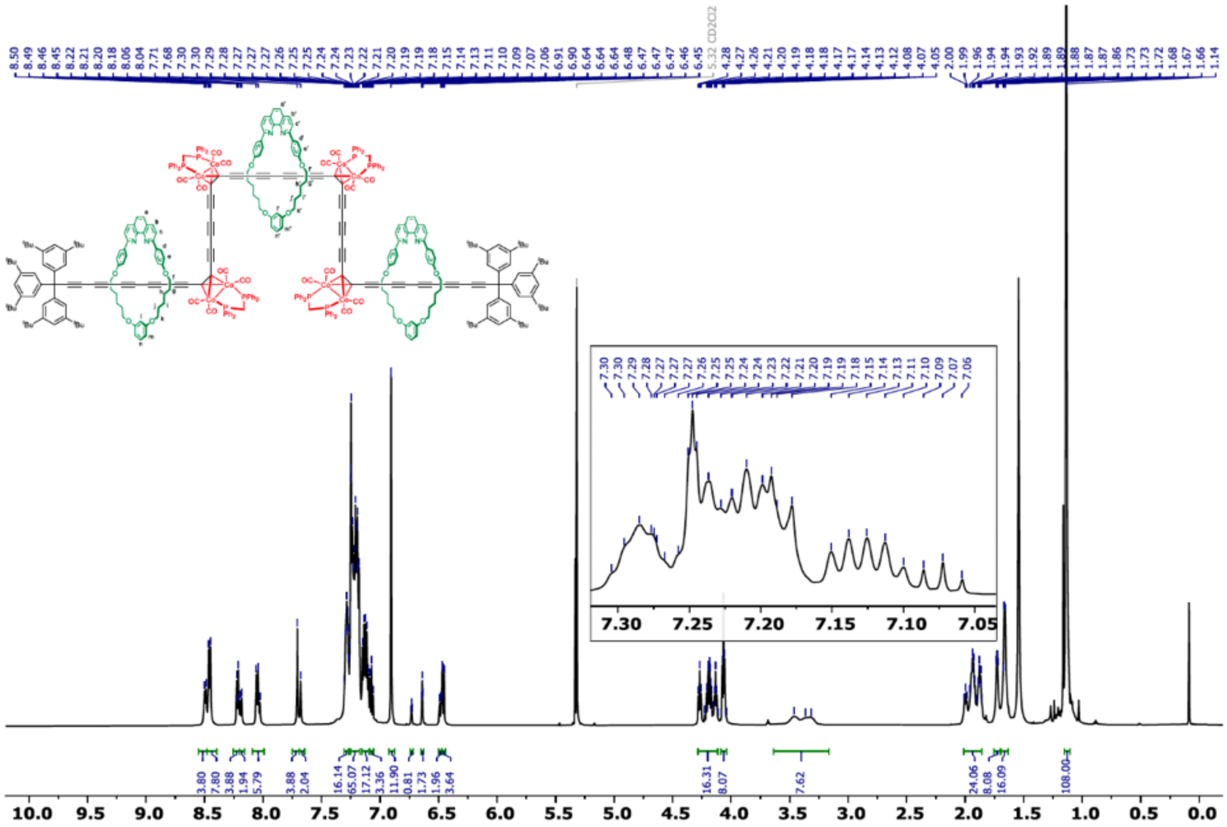

Figure 56. ^{1}H NMR (600 MHz) spectrum of compound ***m*C48·(M$_a$)$_3$** in CD_2Cl_2.

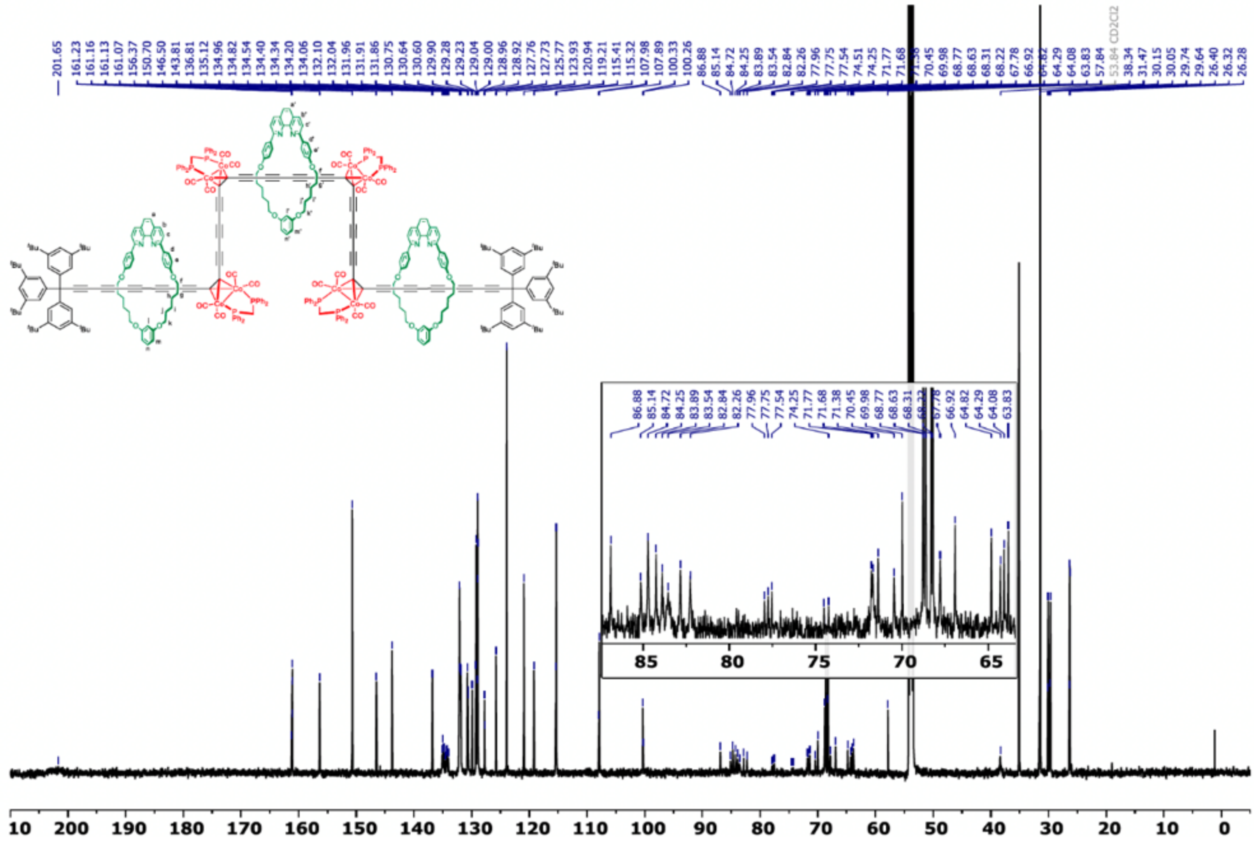

Figure 57. $^{13}C\{^{1}H\}$ NMR (151 MHz) spectrum of compound ***m*C48·(Mₐ)₃** in CD_2Cl_2.

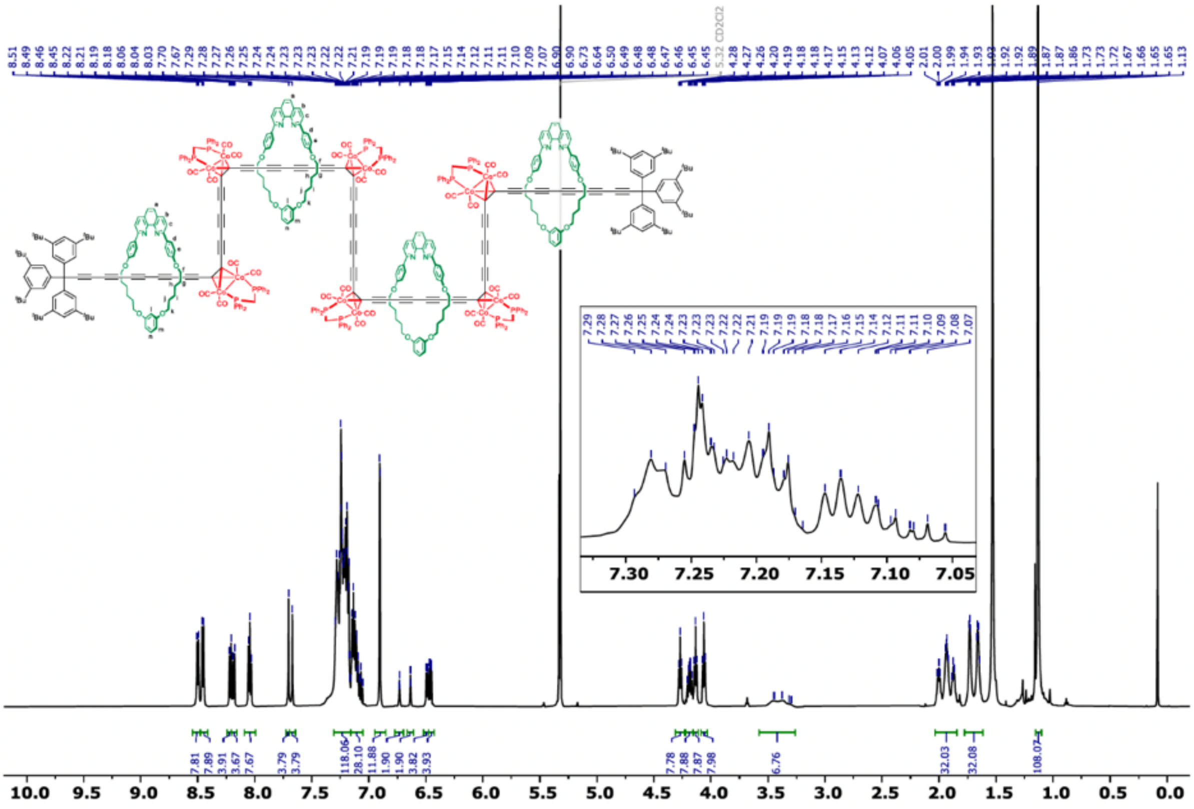

Figure 58. ^{1}H NMR (600 MHz) spectrum of compound ***m*C68·(Mₐ)₄** in CD_2Cl_2.

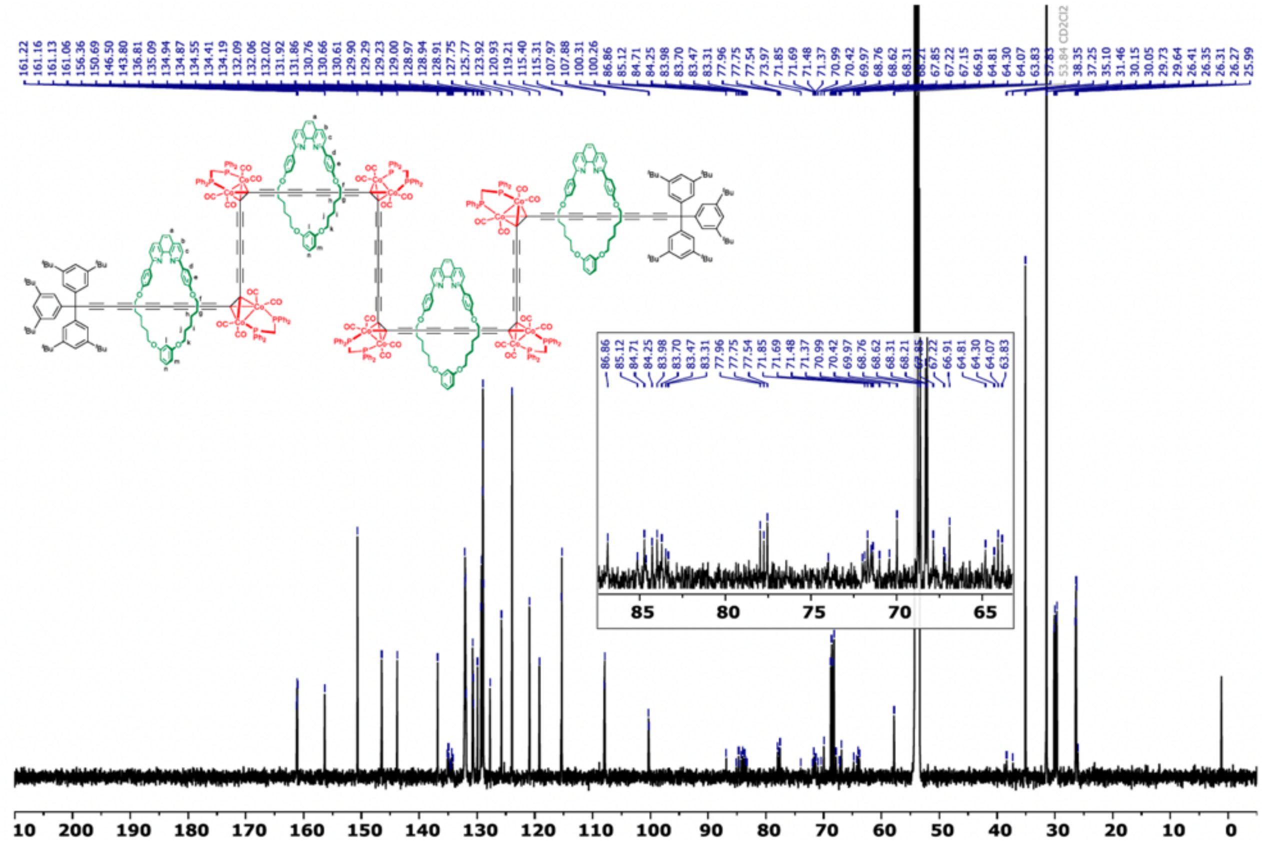

Figure 59. $^{13}C\{^{1}H\}$ NMR (151 MHz) spectrum of compound ***m*C68·(M$_a$)$_4$** in CD_2Cl_2.

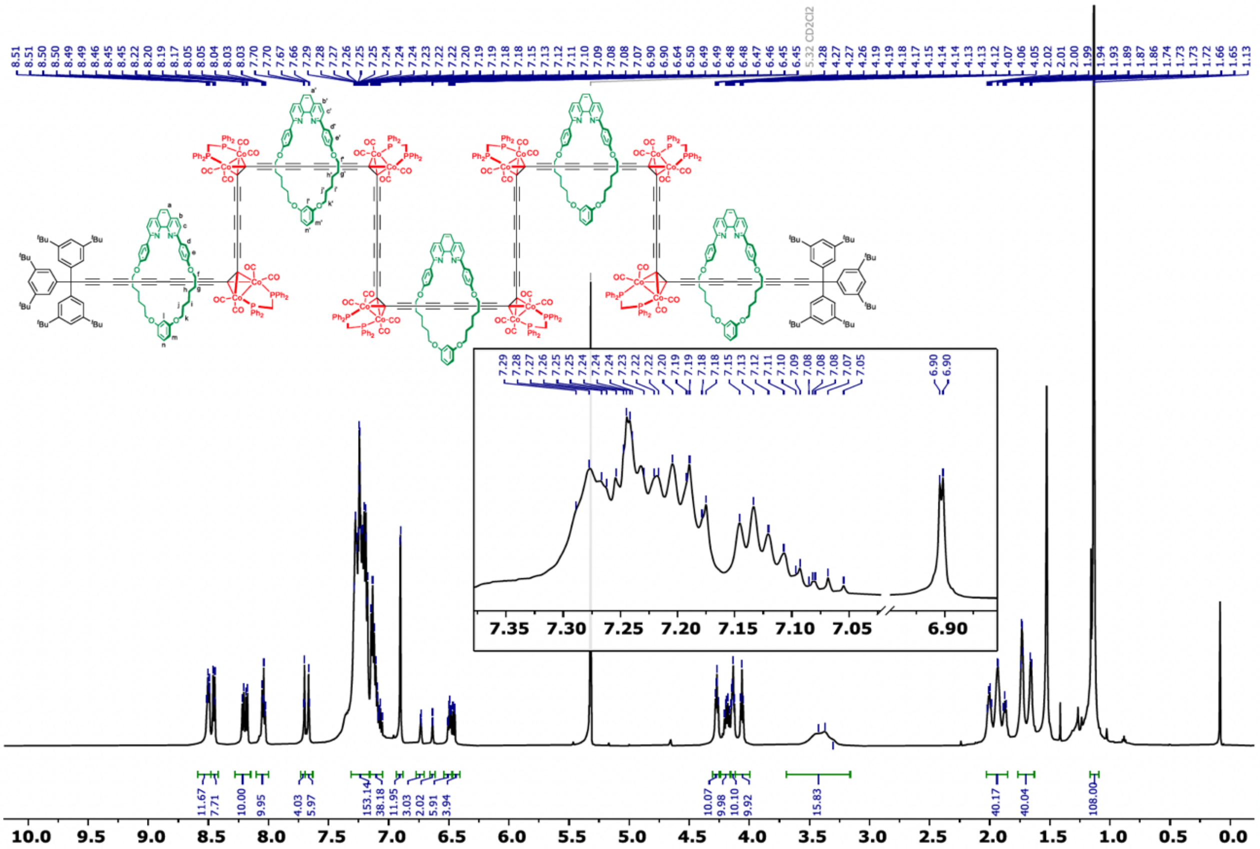

Figure 60. ^{1}H NMR (600 MHz) spectrum of compound ***m*C88·(M$_a$)$_5$** in CD_2Cl_2.

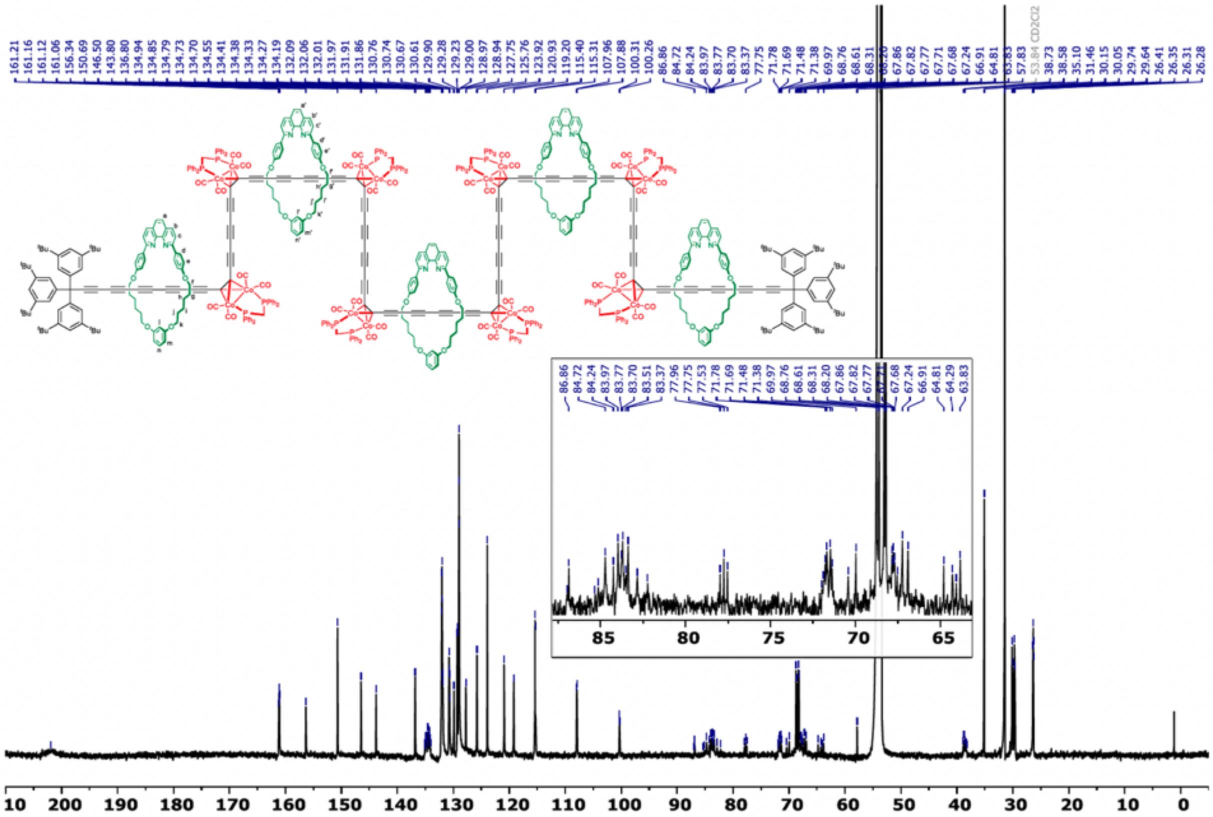

Figure 61. $^{13}C\{^{1}H\}$ NMR (151 MHz) spectrum of compound ***m*C88·(M$_a$)$_5$** in CD_2Cl_2.

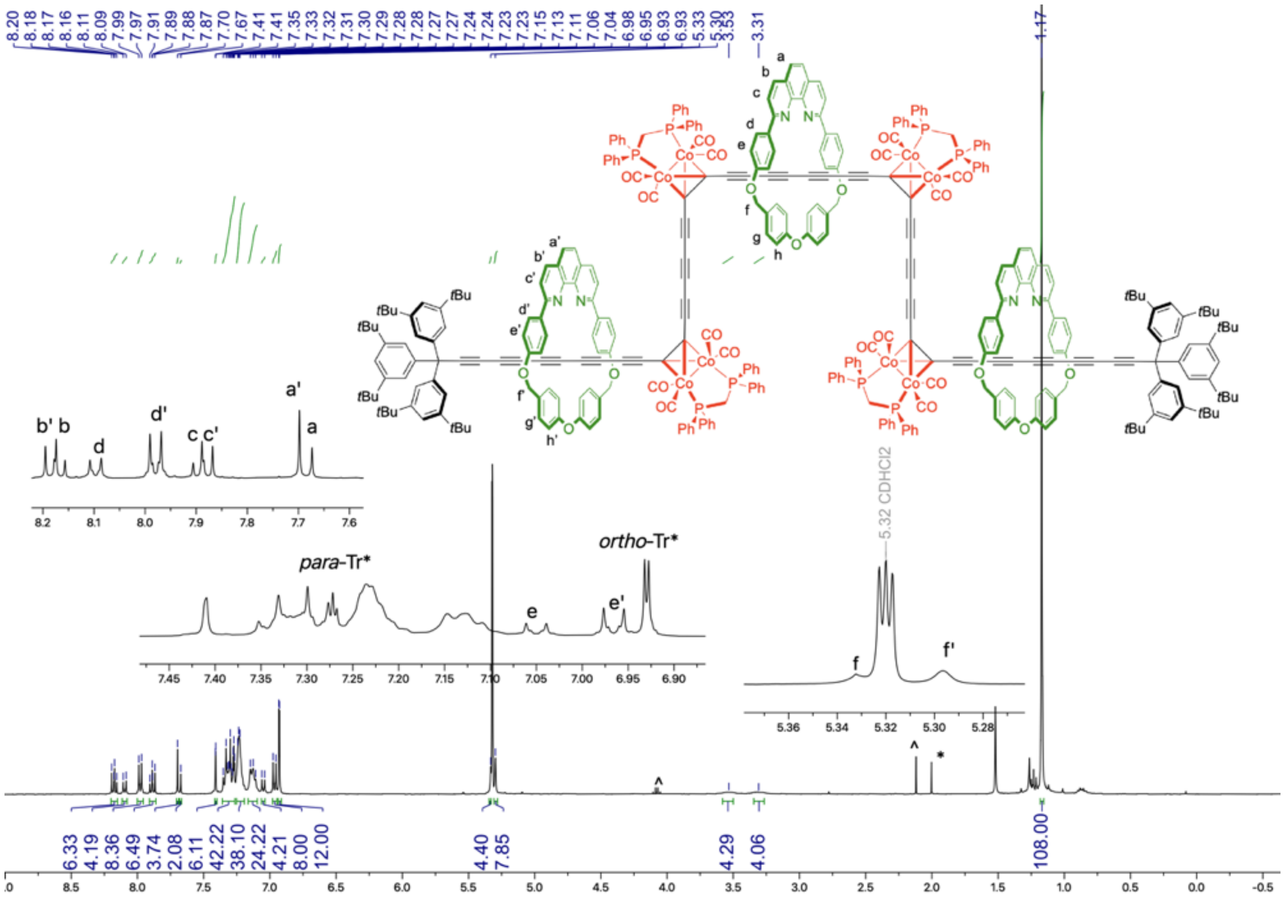

Figure 62. ^{1}H NMR (400 MHz) spectrum of compound ***m*C48·(M$_b$)$_3$** in CD_2Cl_2 (^and *indicate ethyl acetate and acetone, respectively).

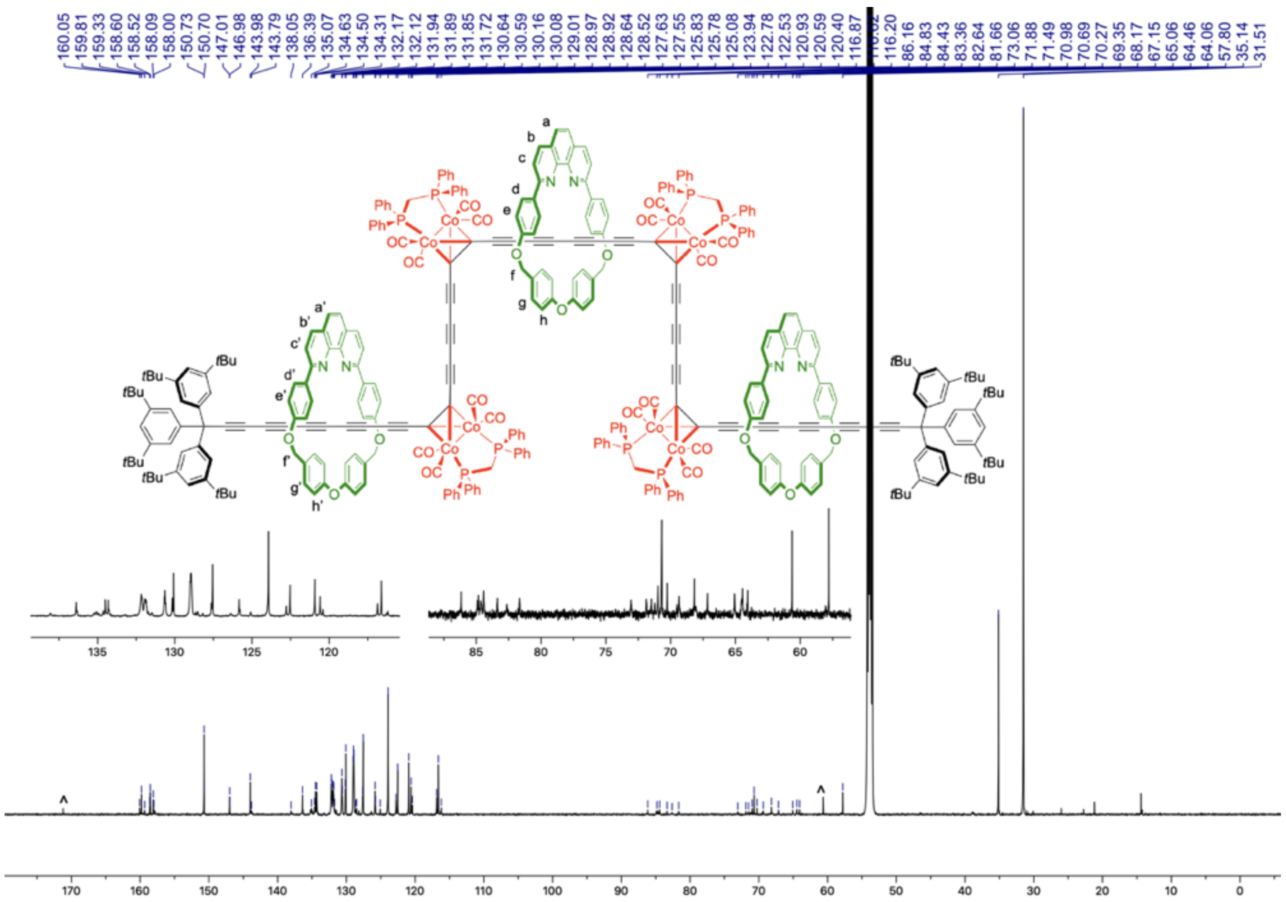

Figure 63. $^{13}C\{^1H\}$ NMR (151 MHz) spectrum of compound ***m*C48·(M$_b$)$_3$** in CD_2Cl_2 (^indicates ethyl acetate).

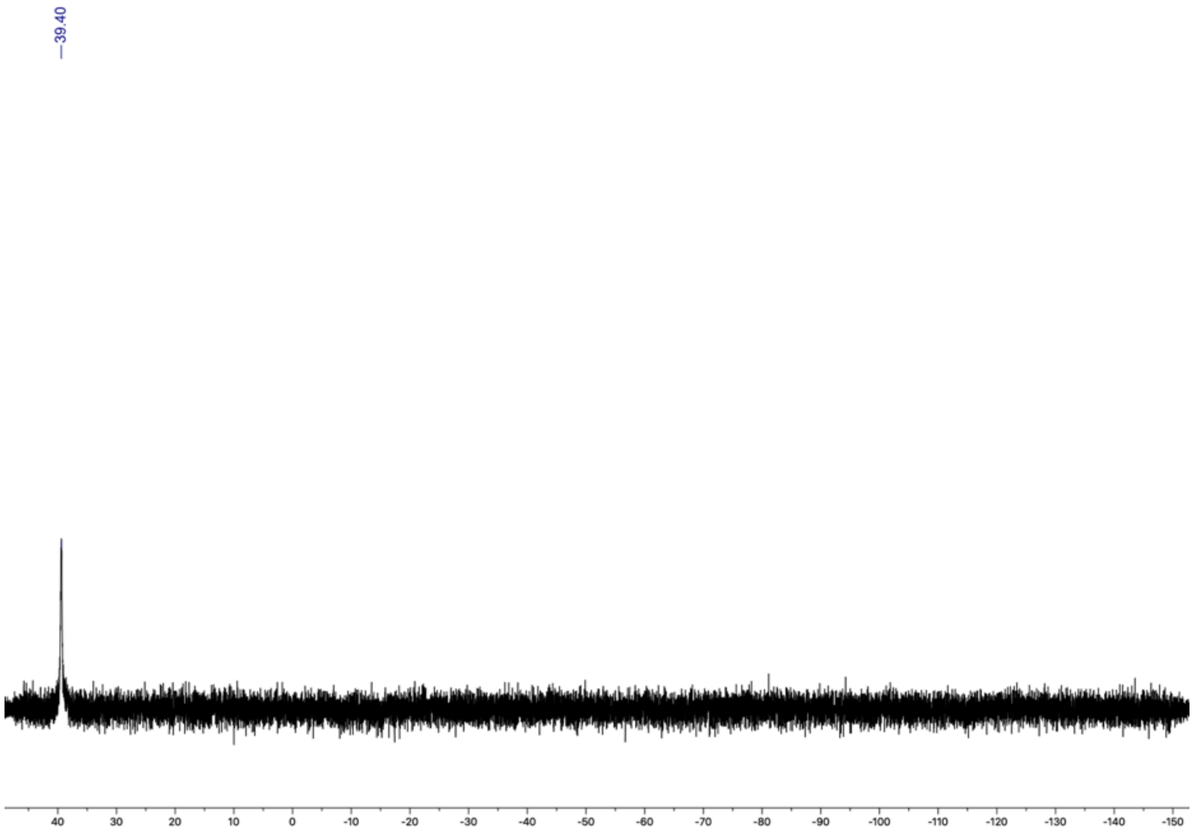

Figure 64. $^{31}P\{^1H\}$ NMR (202 MHz) spectrum of compound ***m*C48·(M$_b$)$_3$** in CD_2Cl_2.

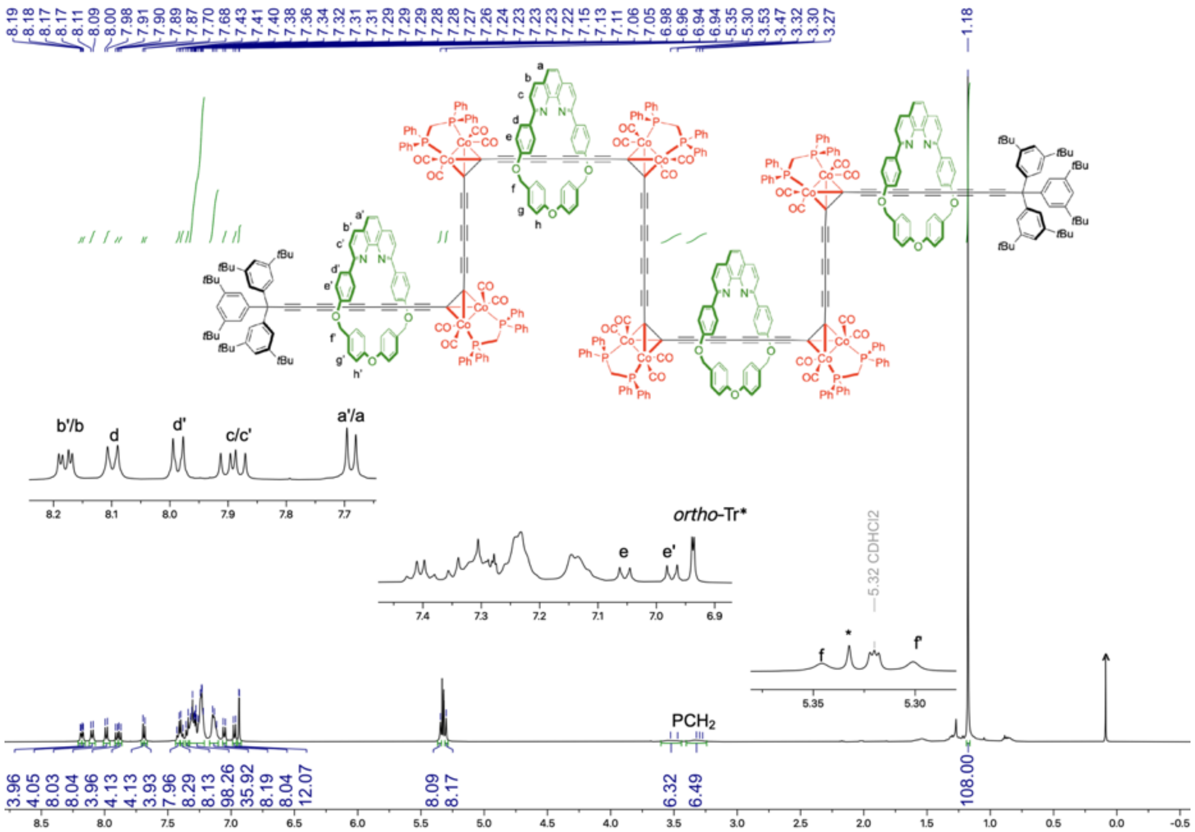

Figure 65. ^{1}H NMR (500 MHz) spectrum of compound ***m*C68·(M$_b$)$_4$** in CD_2Cl_2 (^and *indicate silicon grease and residual CH_2Cl_2, respectively).

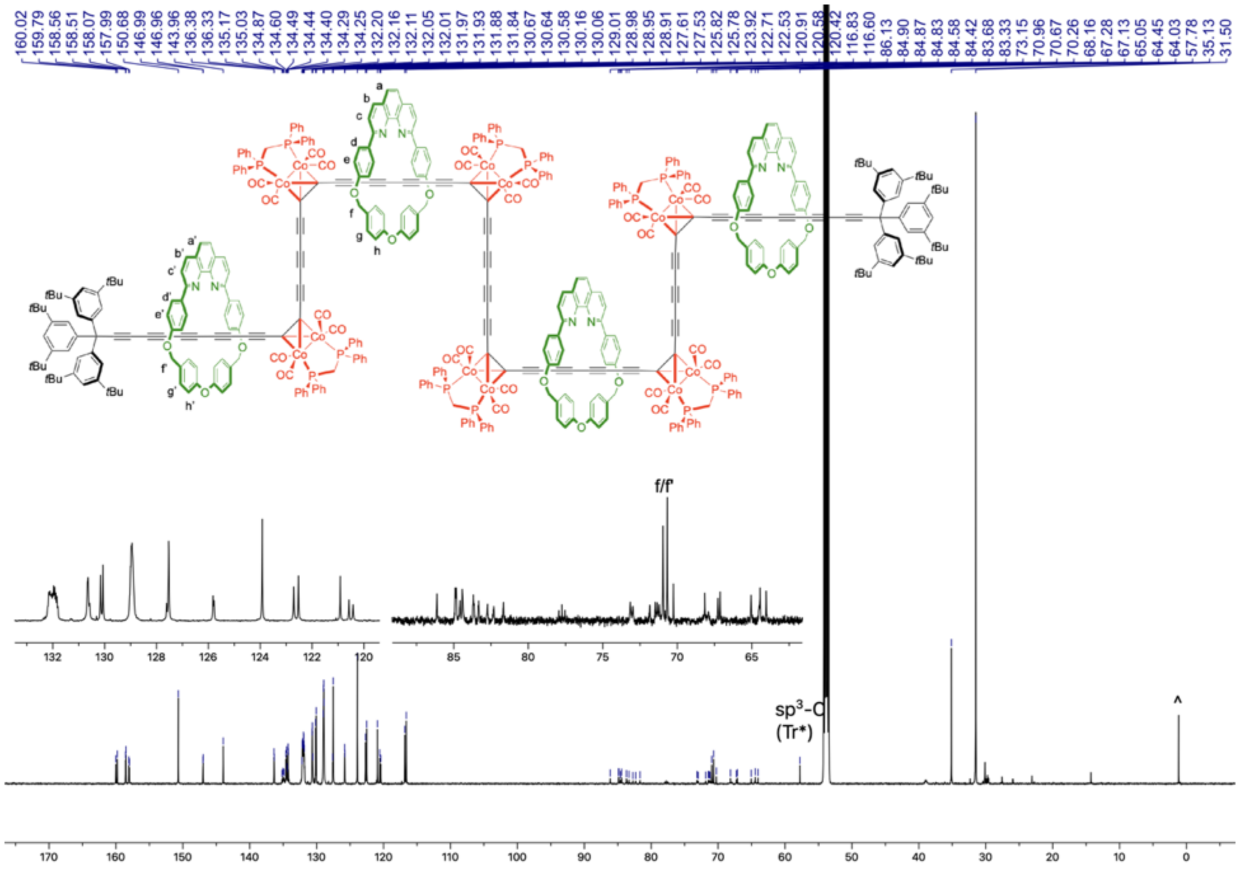

Figure 66. $^{13}C\{^{1}H\}$ NMR (151 MHz) spectrum of compound ***m*C68·(M$_b$)$_4$** in CD_2Cl_2 (^indicates silicone grease).

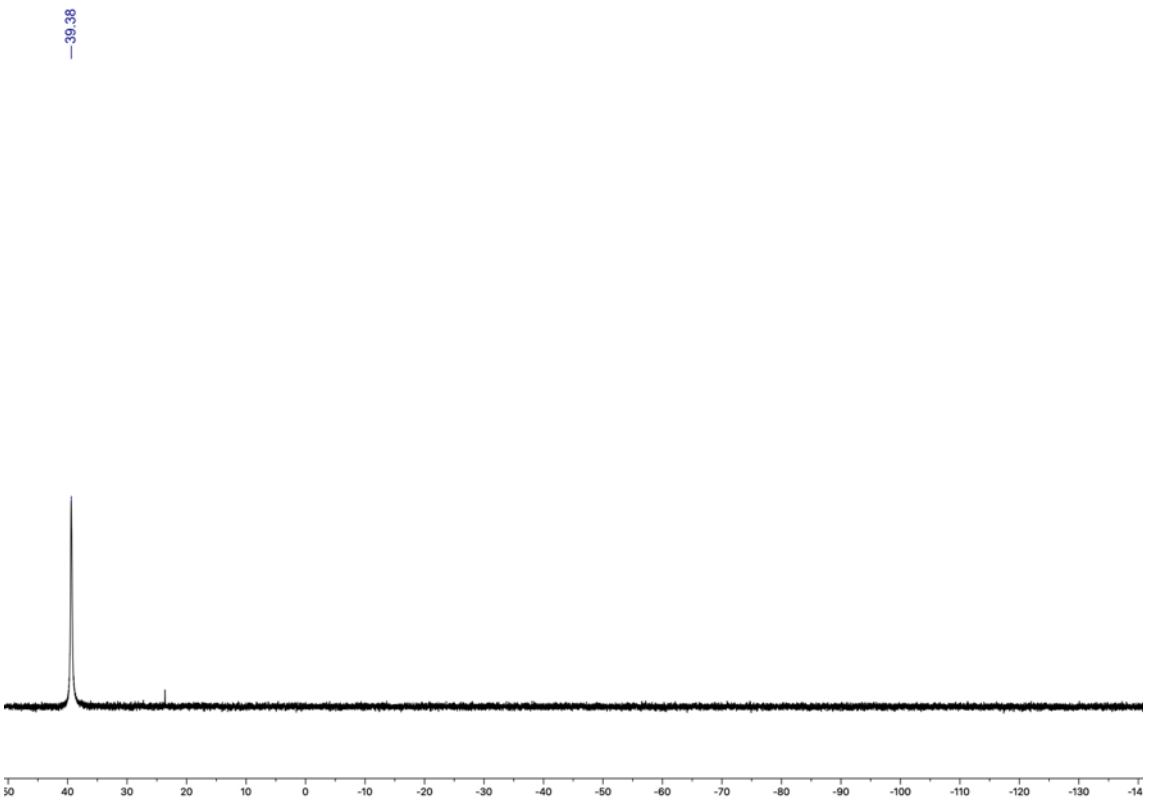

Figure 67. $^{31}P\{^{1}H\}$ NMR (202 MHz) spectrum of compound ***m*C68·(M$_b$)$_4$** in CD_2Cl_2.

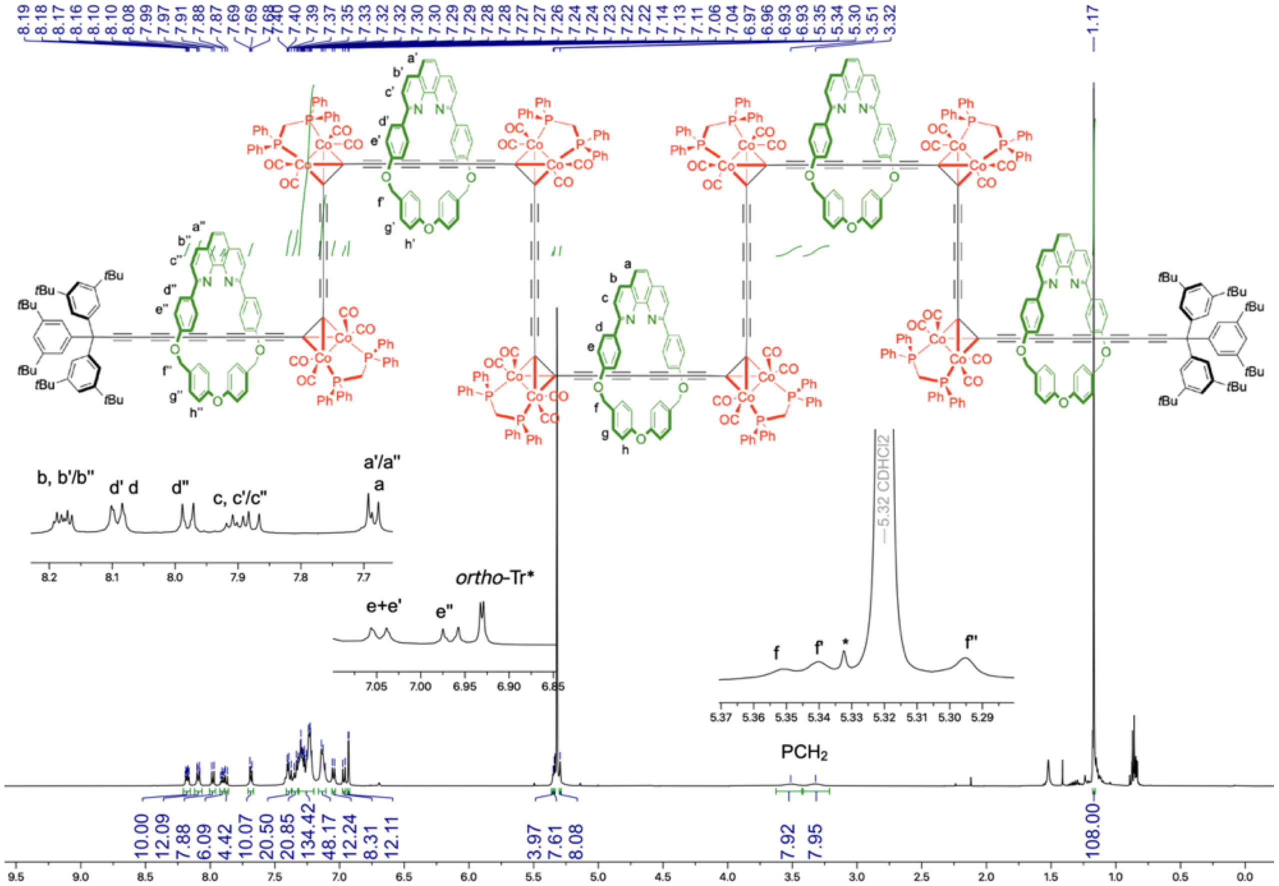

Figure 68. ^{1}H NMR (500 MHz) spectrum of compound ***m*C88·(M$_b$)$_5$** in CD_2Cl_2 (*indicates residual CH_2Cl_2).

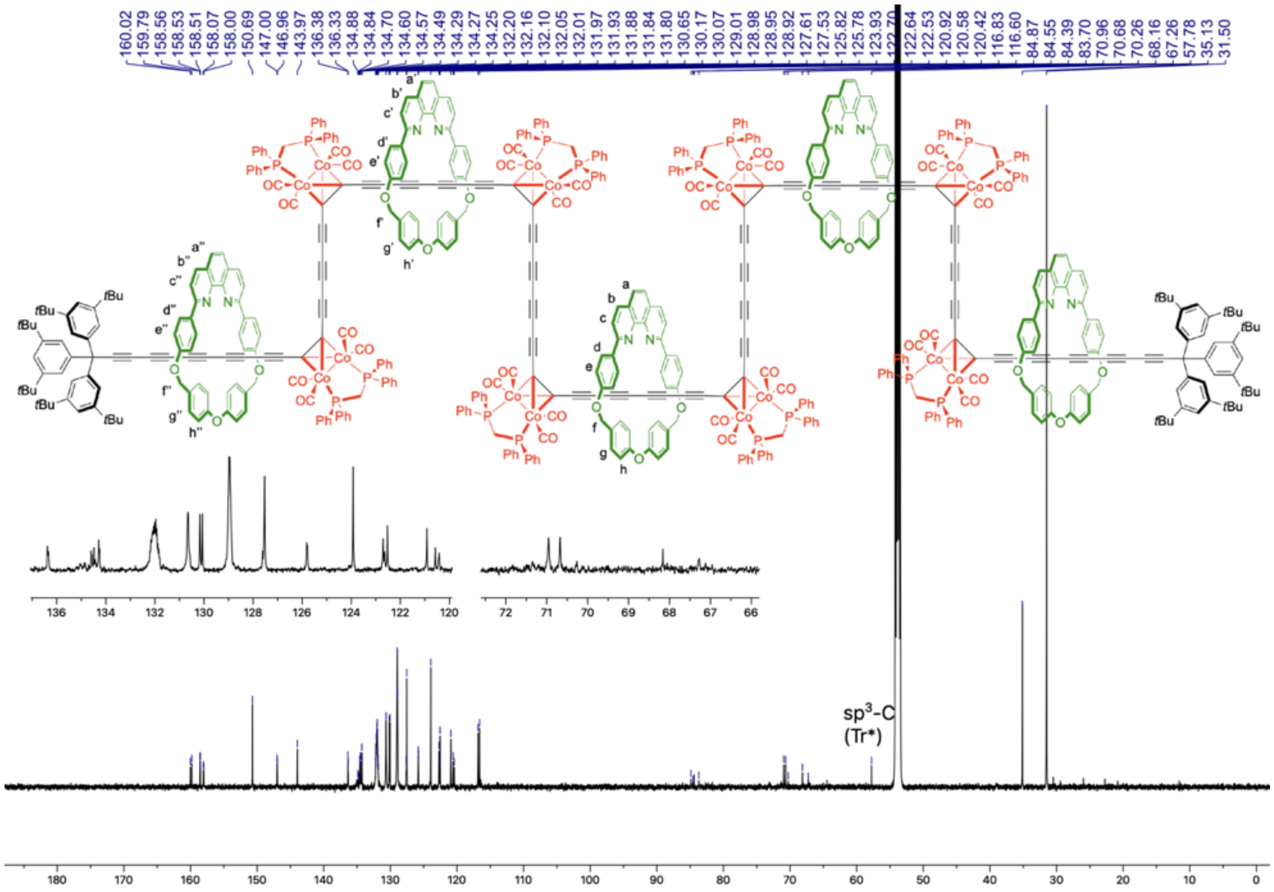

Figure 69. $^{13}C\{^{1}H\}$ NMR (151 MHz) spectrum of compound ***m*C88·(M$_b$)$_5$** in CD_2Cl_2.

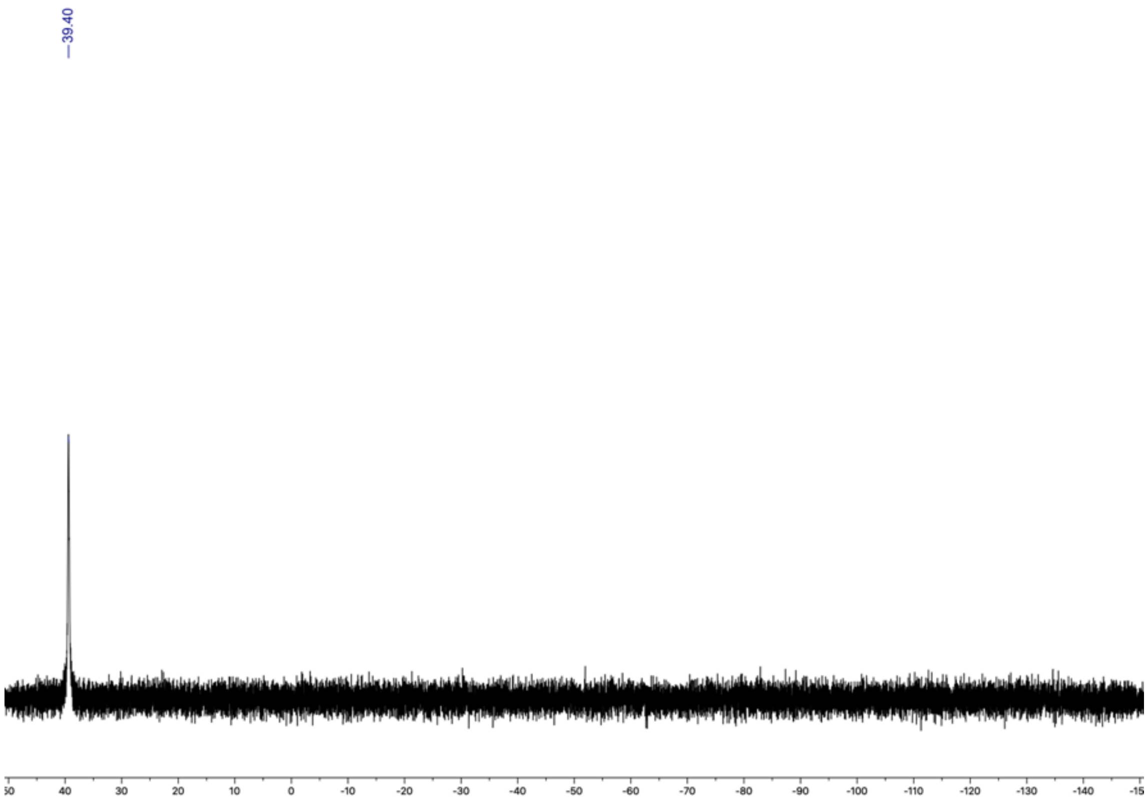

Figure 70. $^{31}P\{^{1}H\}$ NMR (202 MHz) spectrum of compound ***m*C88·(M$_b$)$_5$** in CD_2Cl_2.

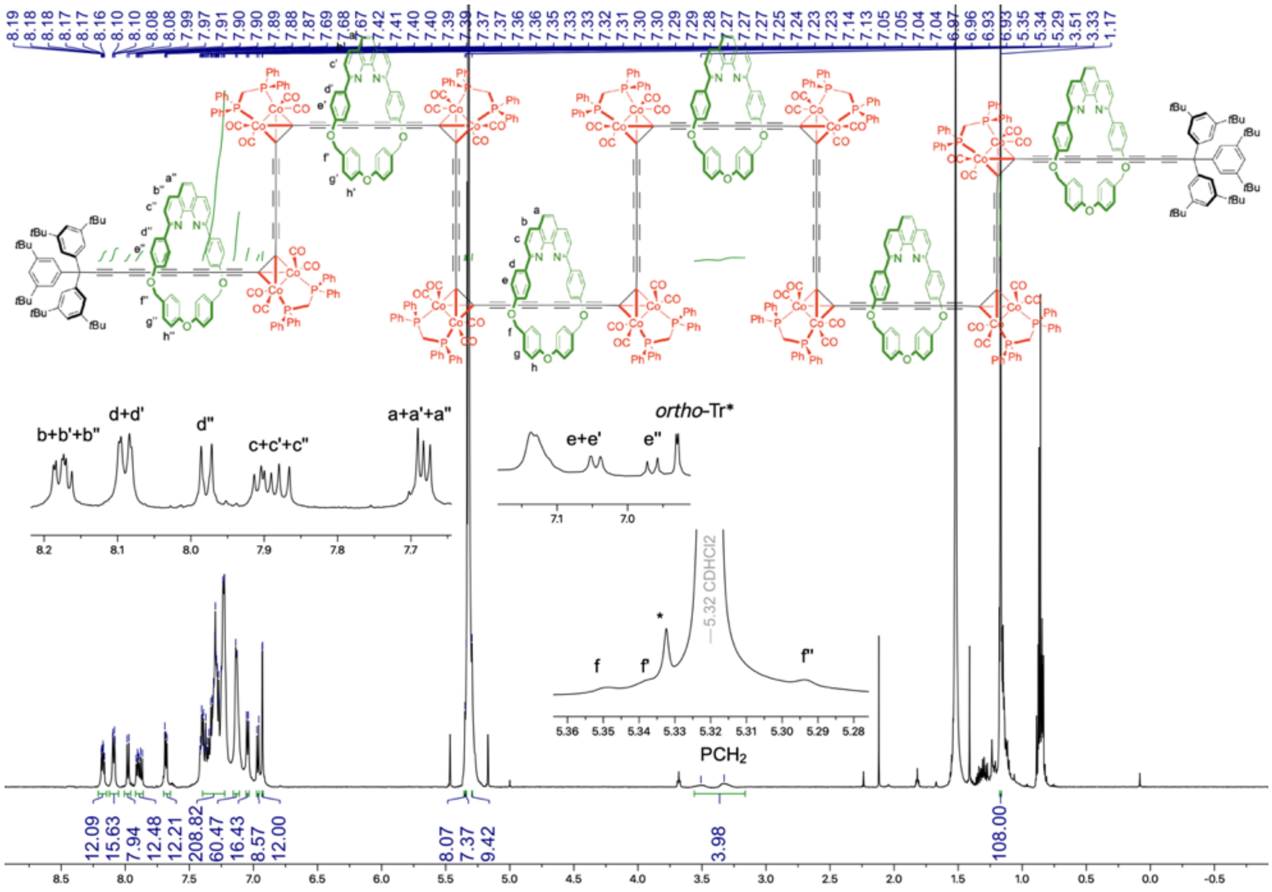

Figure 71. ^{1}H NMR (600 MHz) spectrum of compound ***m*C108·(M$_b$)$_6$** in CD_2Cl_2 (*indicates residual CH_2Cl_2). The integration of PCH_2 resonance was significantly less due to the long relaxing time required and phasing with less accuracy.

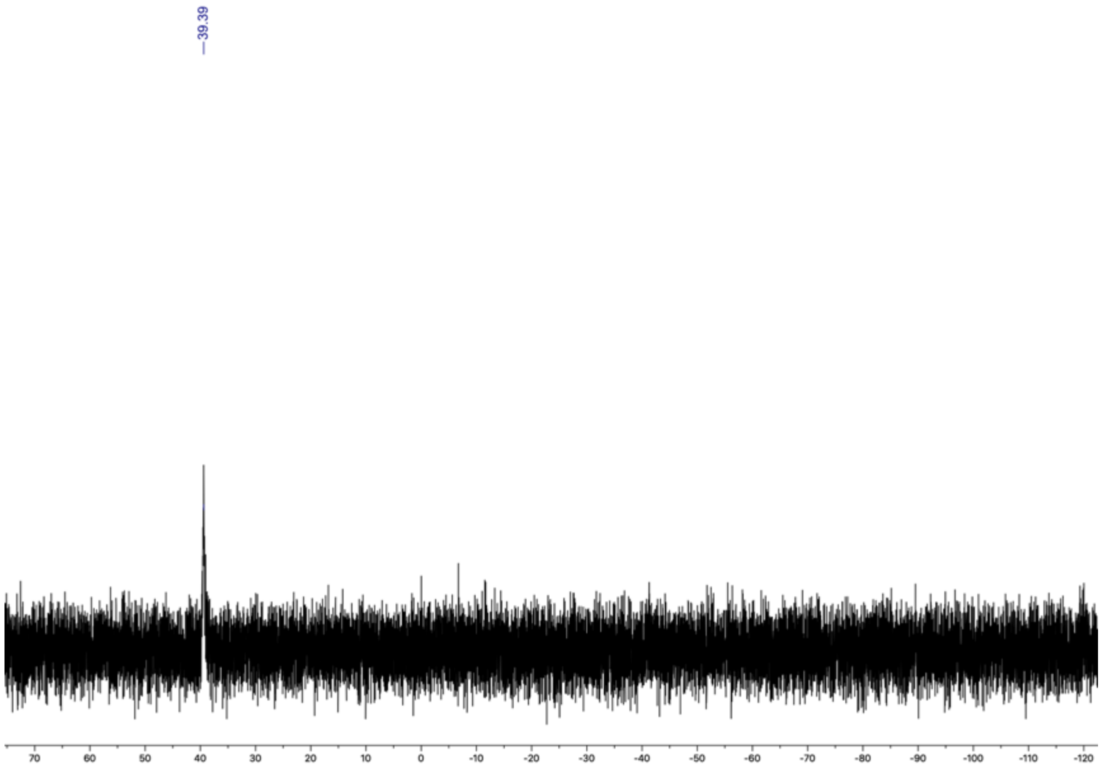

Figure 72. $^{31}P\{^{1}H\}$ NMR (243 MHz) spectrum of compound ***m*C108·(M$_b$)$_6$** in CD_2Cl_2.

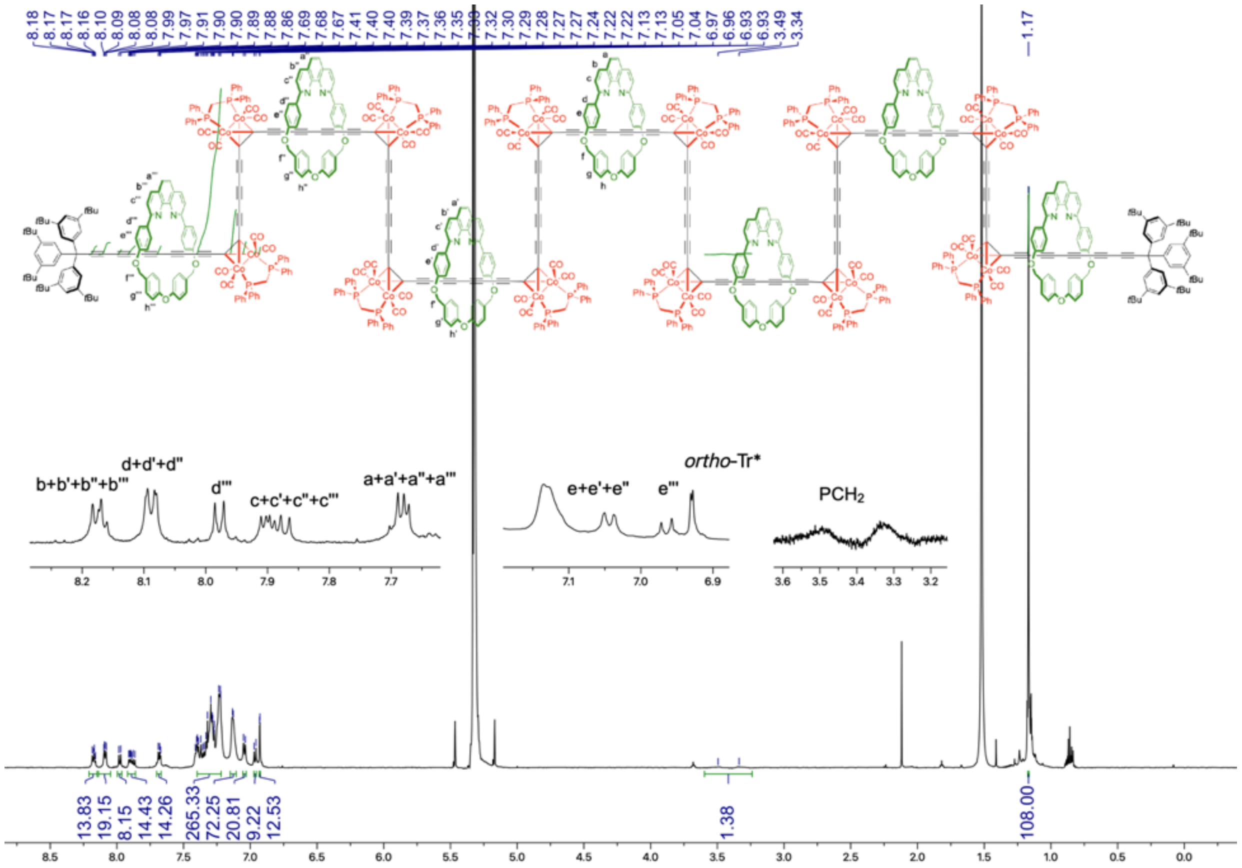

Figure 73. ^{1}H NMR (600 MHz) spectrum of compound ***m*C128·(M$_b$)$_7$** in CD_2Cl_2. The methylene groups from the macrocycles were not resolved due to significant overlap with the residual $CHDCl_2$. The integration of PCH_2 resonance was significantly less due to the long relaxing time required and phasing with less accuracy.

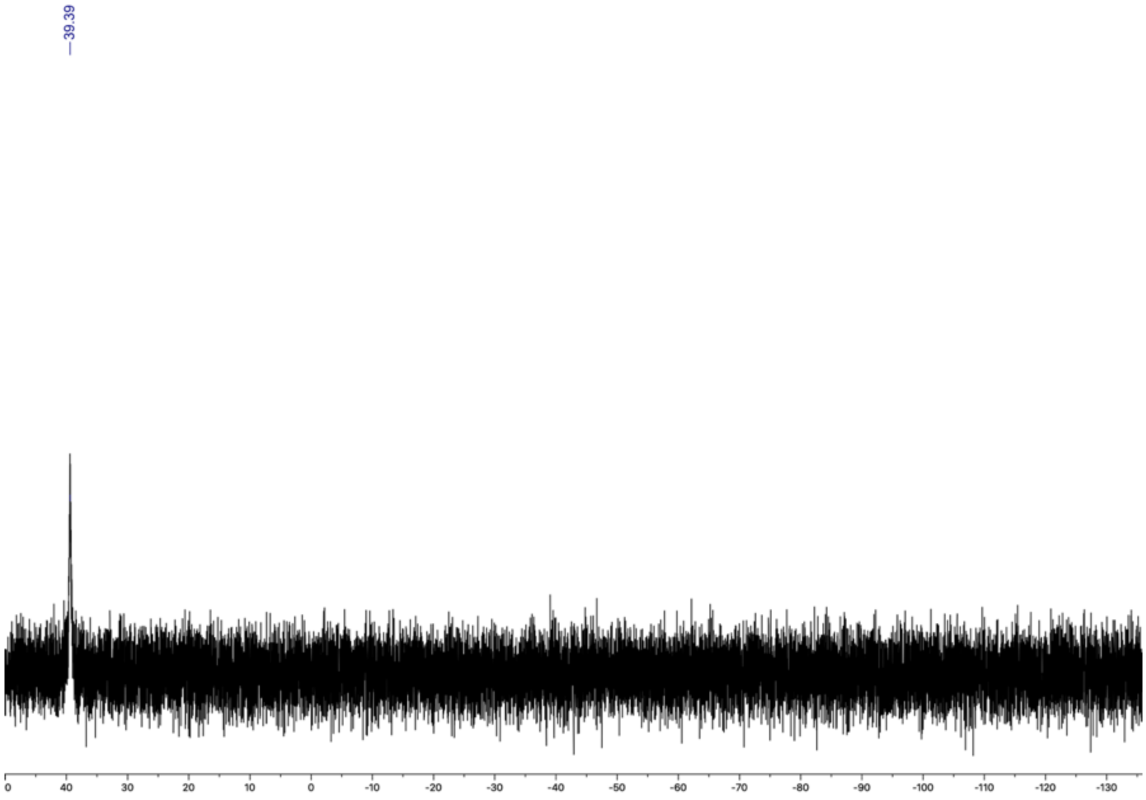

Figure 74. $^{31}P\{^{1}H\}$ NMR (202 MHz) spectrum of compound ***m*C128·(M$_b$)$_7$** in CD_2Cl_2.

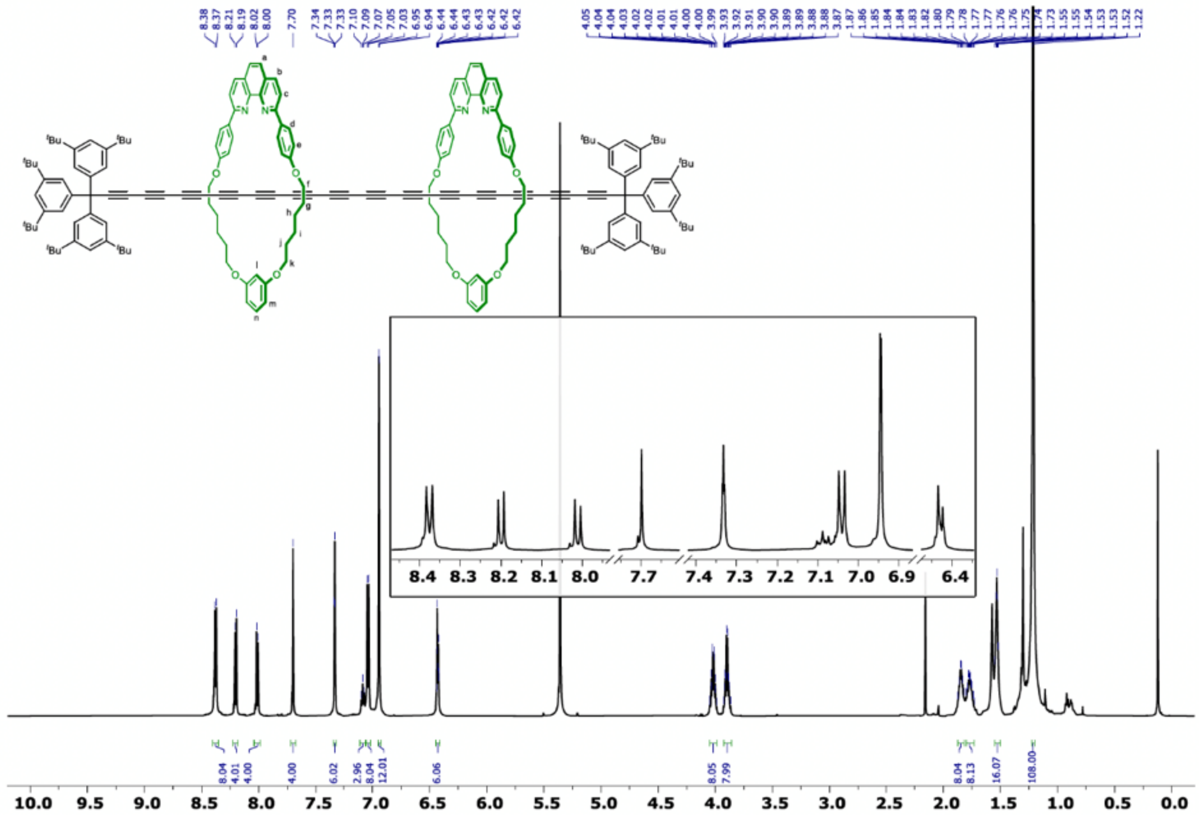

Figure 75. ^{1}H NMR (600 MHz) spectrum of compound **C28·(M$_a$)$_2$** in CD_2Cl_2.

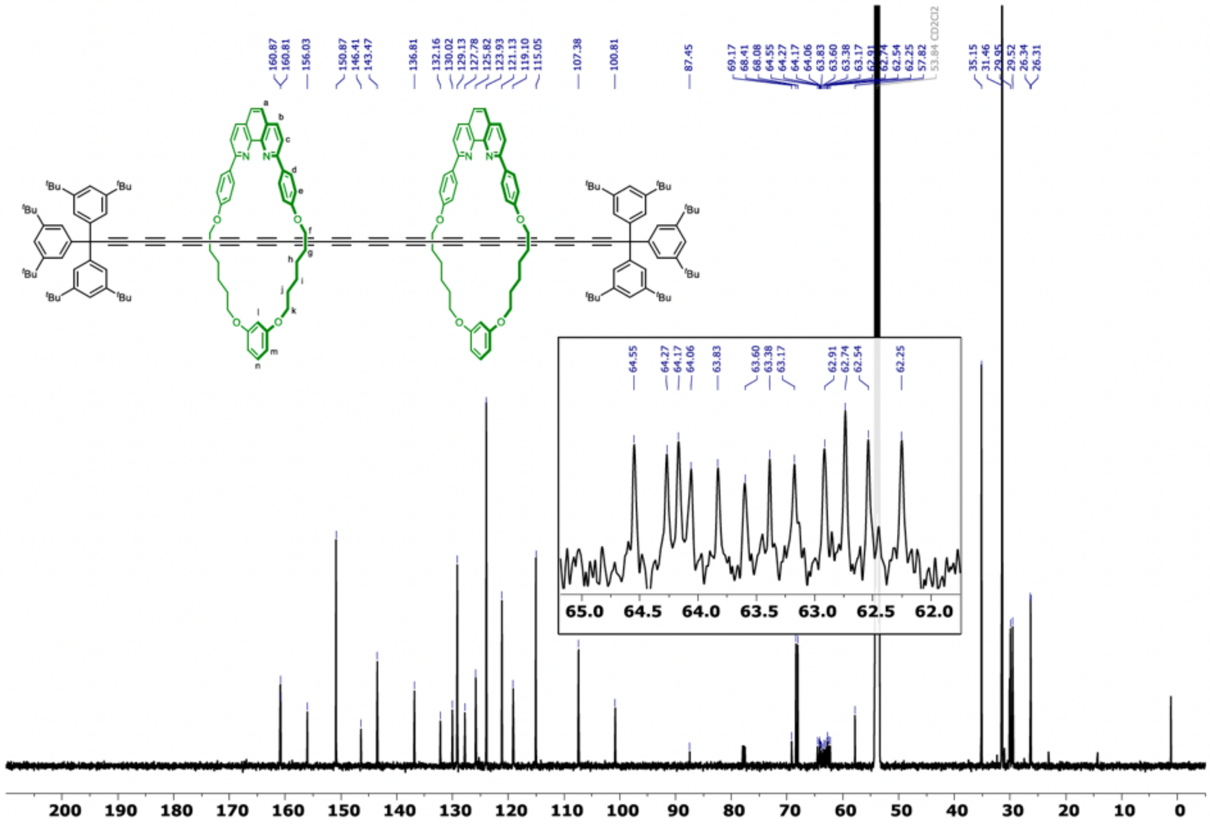

Figure 76. $^{13}C\{^{1}H\}$ NMR (151 MHz) spectrum of compound **C28·(M$_a$)$_2$** in CD_2Cl_2.

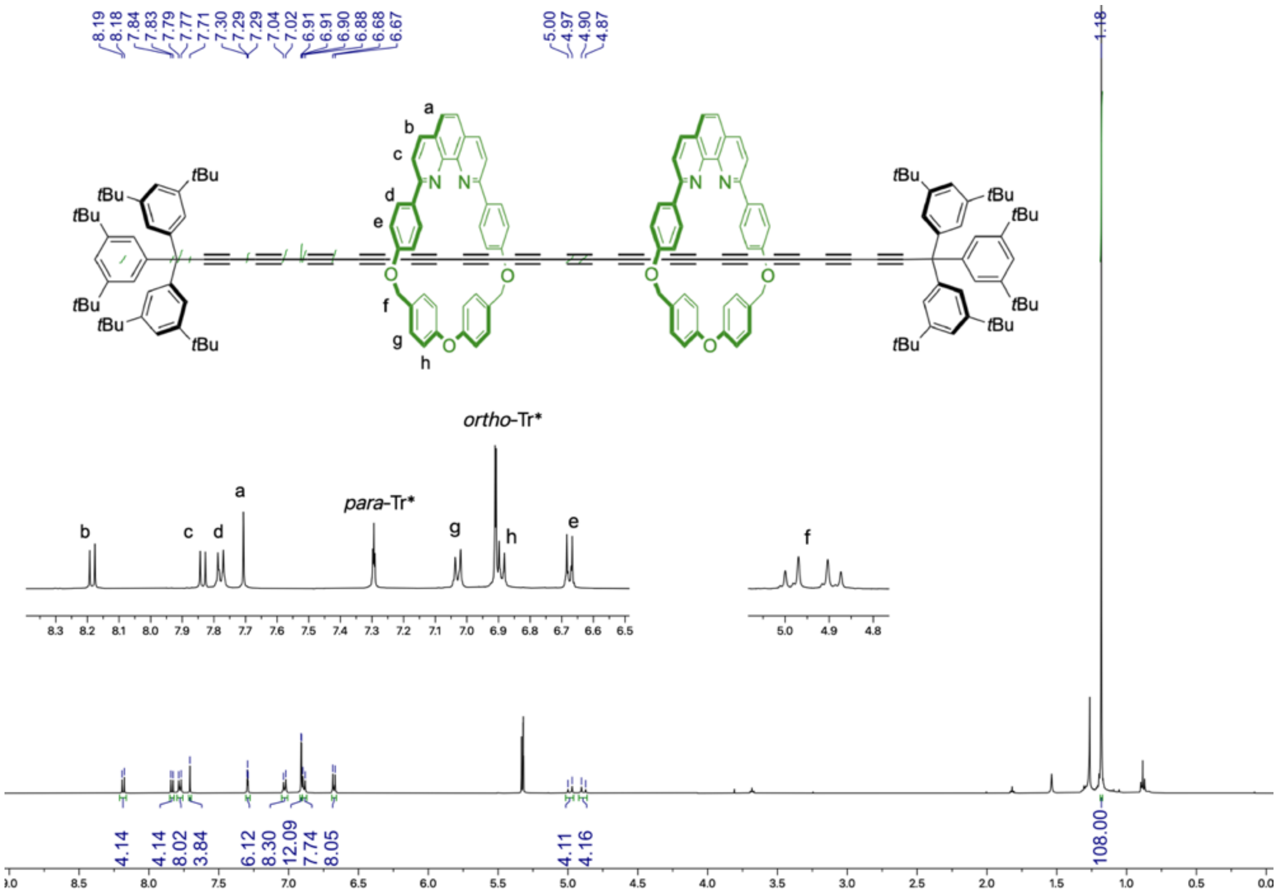

Figure 77. ^{1}H NMR (500 MHz) spectrum of compound **C28·(M$_b$)$_2$** in CD_2Cl_2.

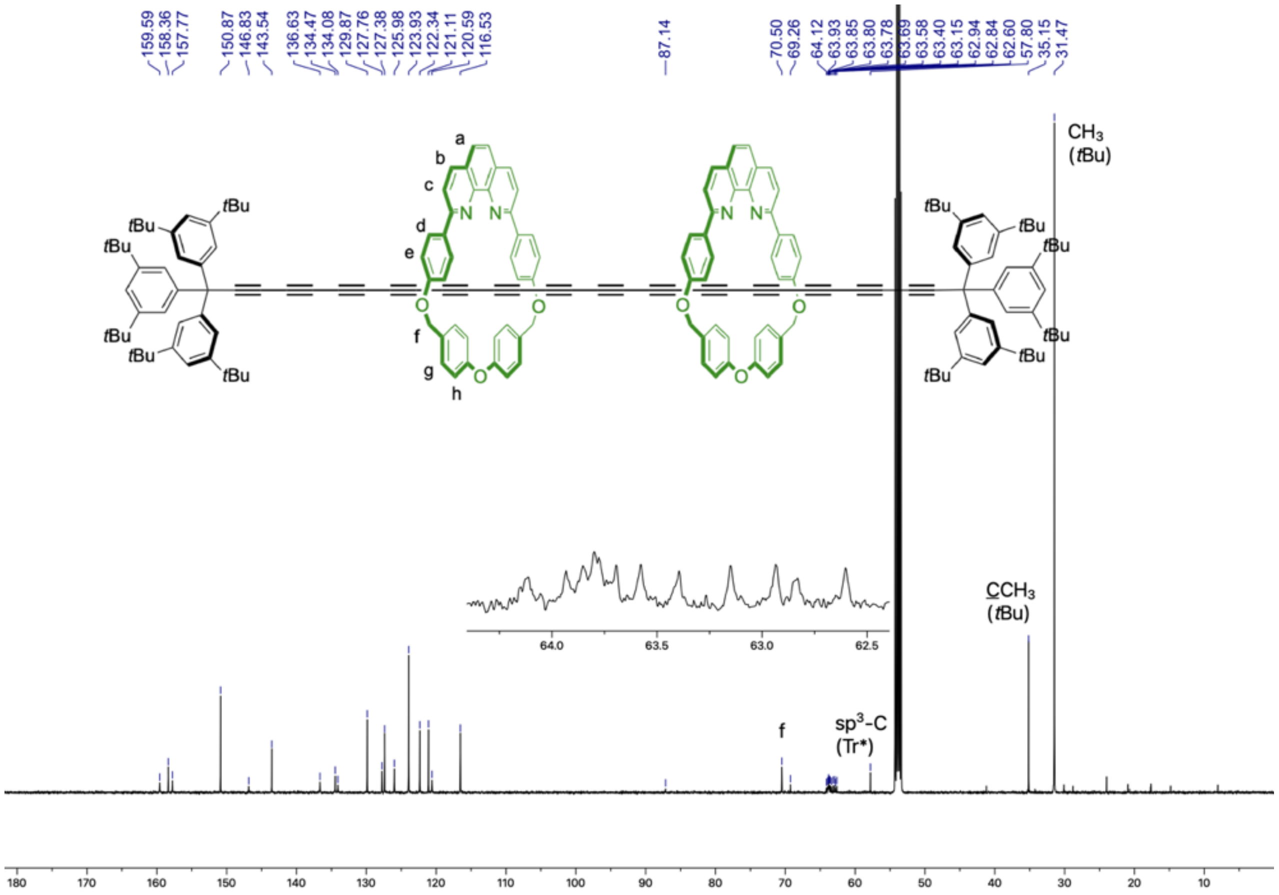

Figure 78. $^{13}C\{^{1}H\}$ NMR (126 MHz) spectrum of compound **C28·(M$_b$)$_2$** in CD_2Cl_2.

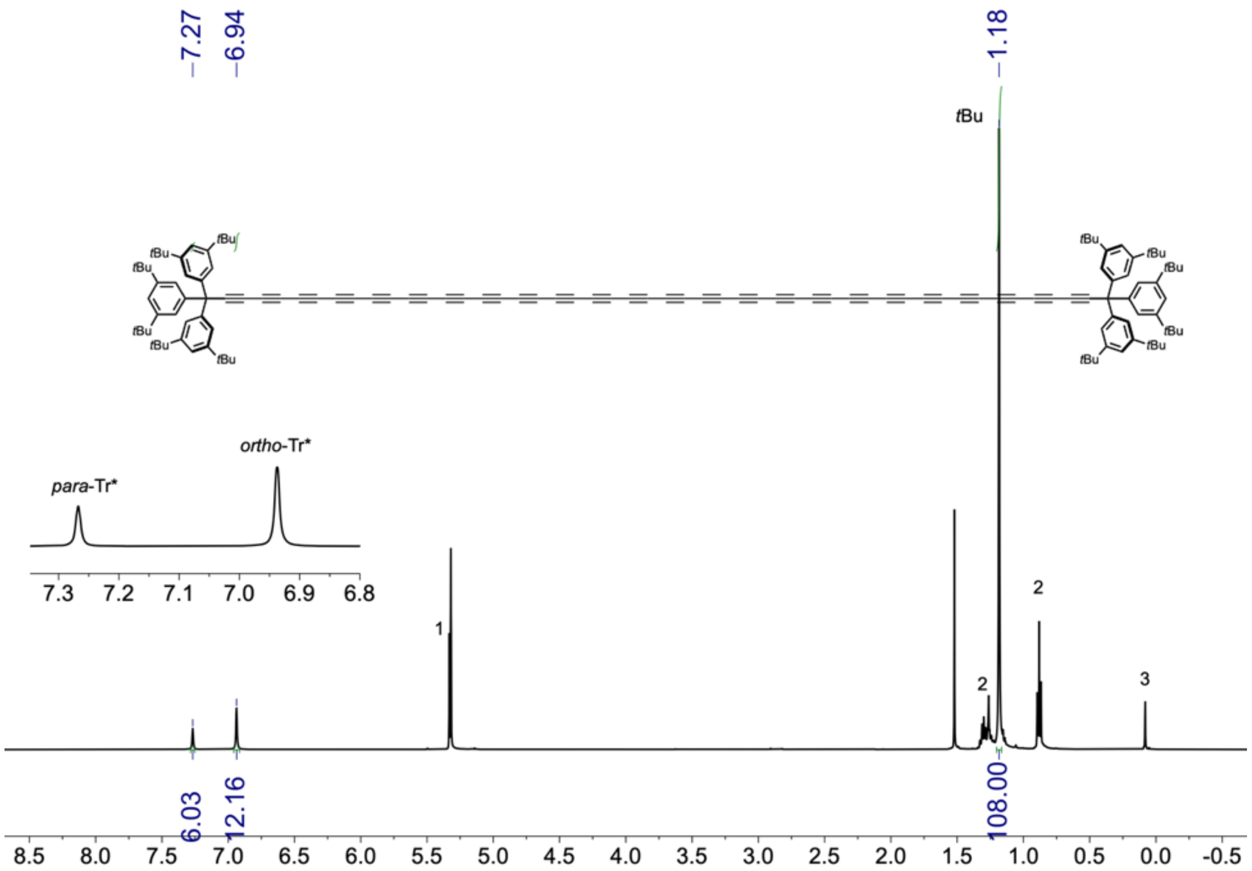

Figure 79. ^{1}H NMR (500 MHz) spectrum of Tr*[24] thread **C48** in CD_2Cl_2 (1, 2, and 3 indicate residual CH_2Cl_2, pentane, and silicon grease, respectively).

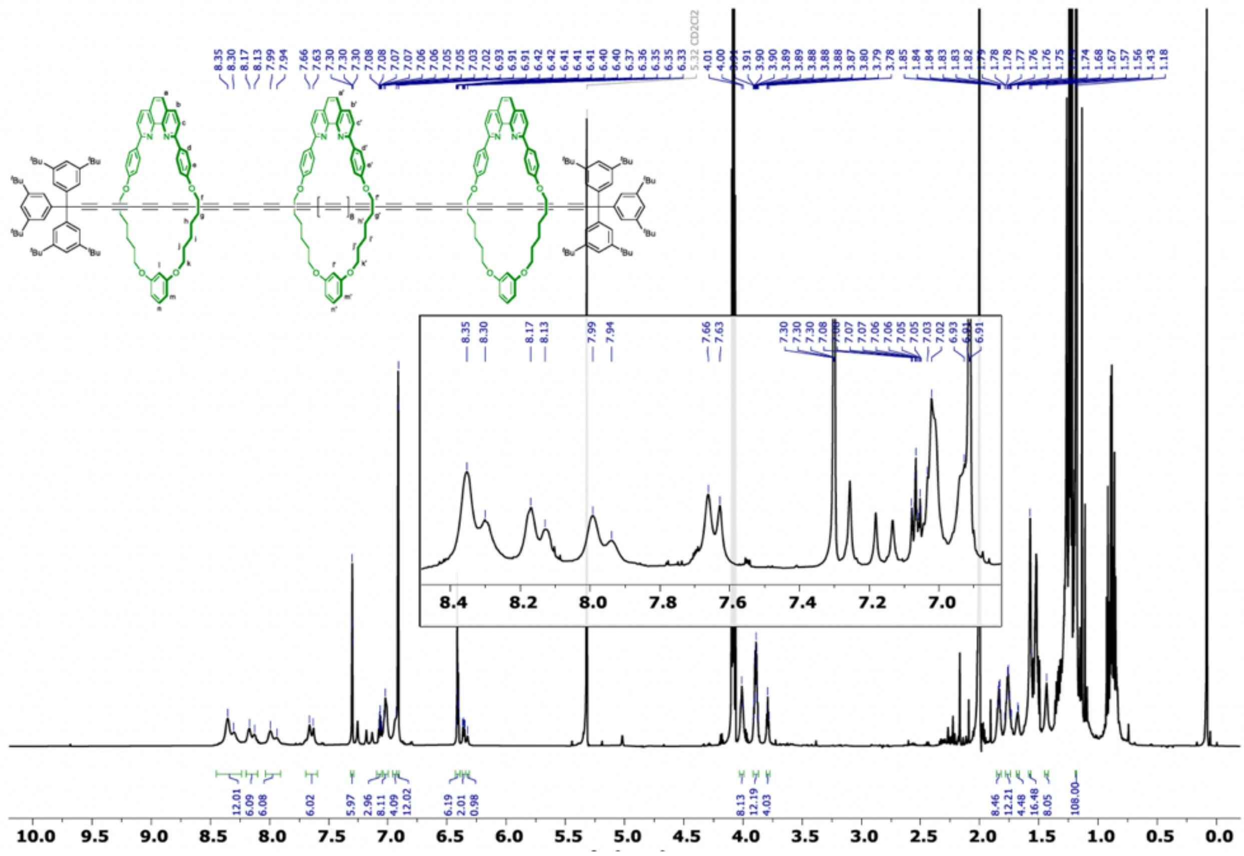

Figure 80. ^{1}H NMR (600 MHz) spectrum of compound **C48·(M$_a$)$_3$** in CD_2Cl_2.

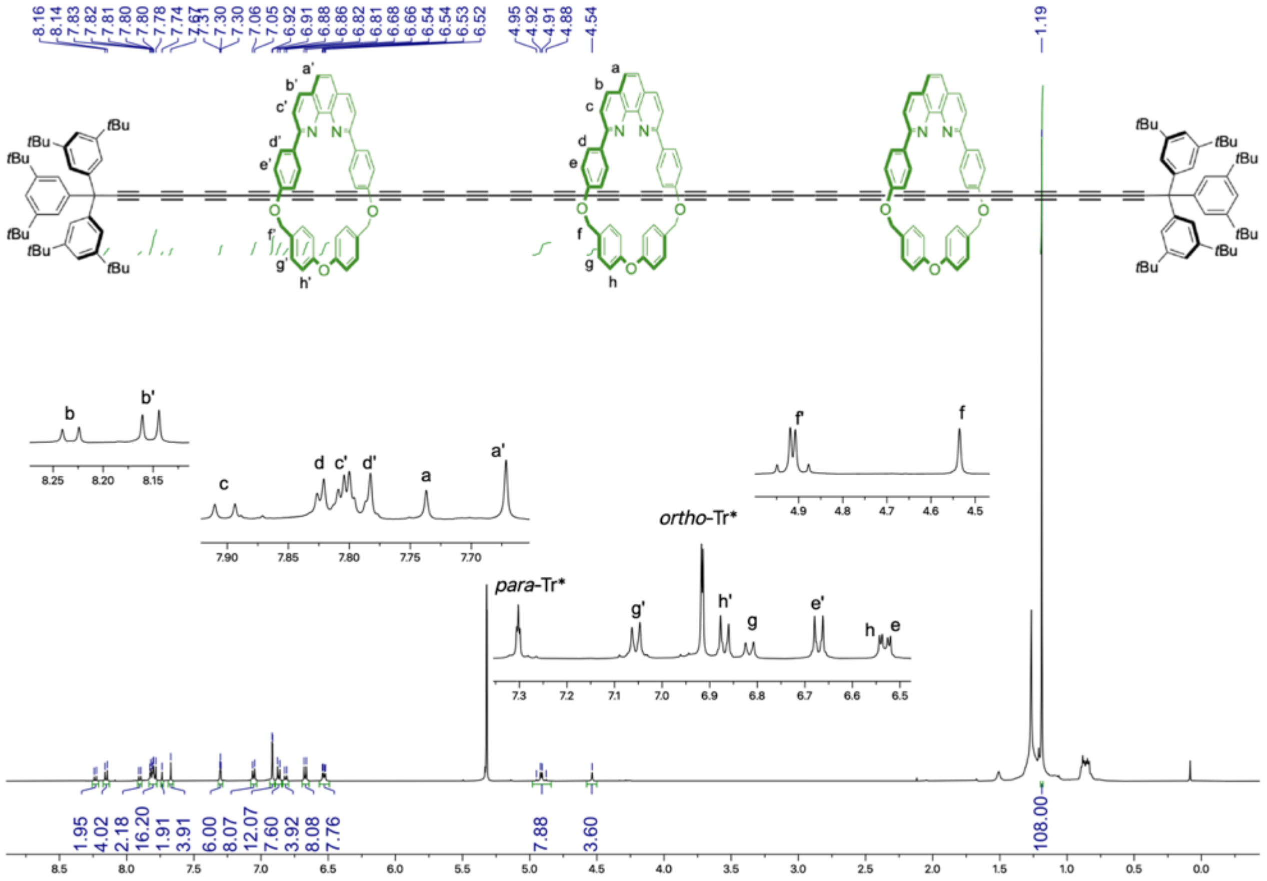

Figure 81. ^{1}H NMR (500 MHz) spectrum of compound **C48·(M$_b$)$_3$** in CD_2Cl_2.

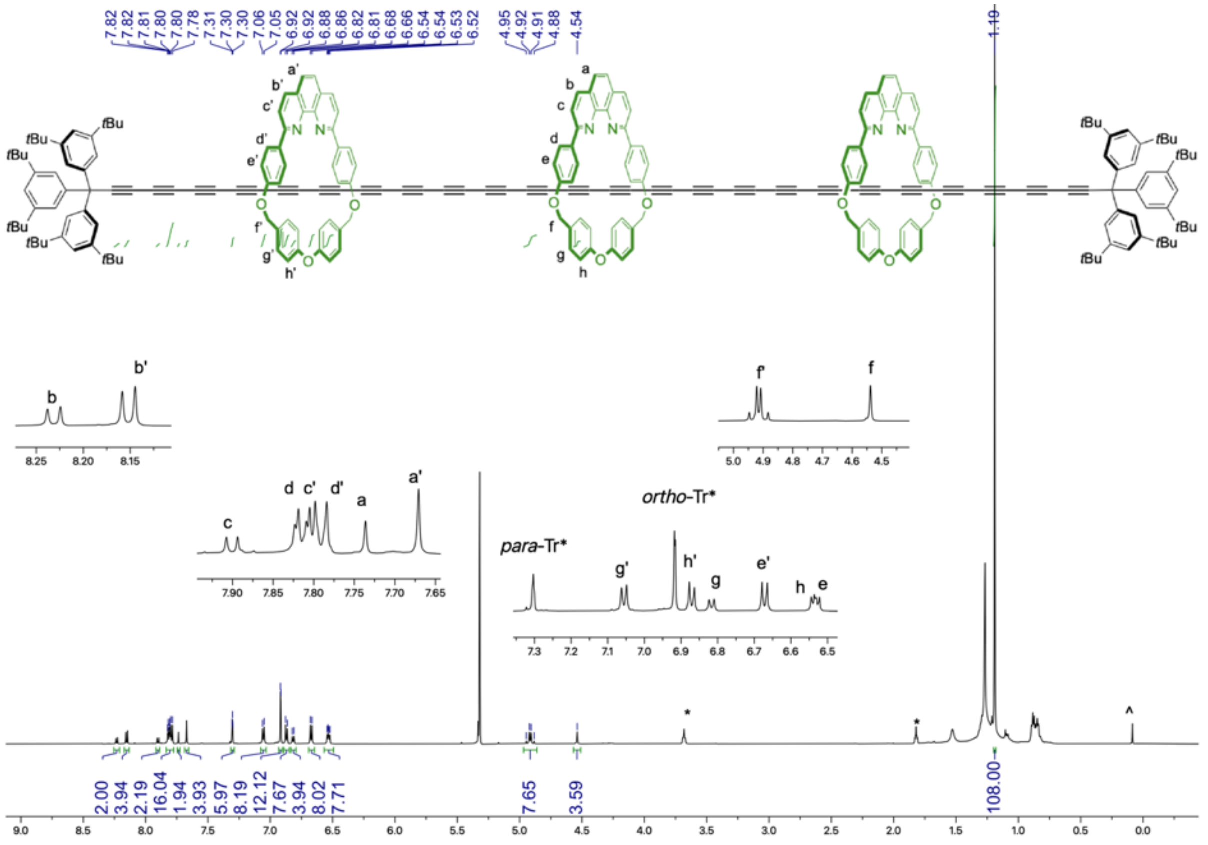

Figure 82. ^{1}H NMR (600 MHz) spectrum of compound **C48·(M$_b$)$_3$** in CD_2Cl_2 (^and *indicate silicon grease and residual THF, respectively).

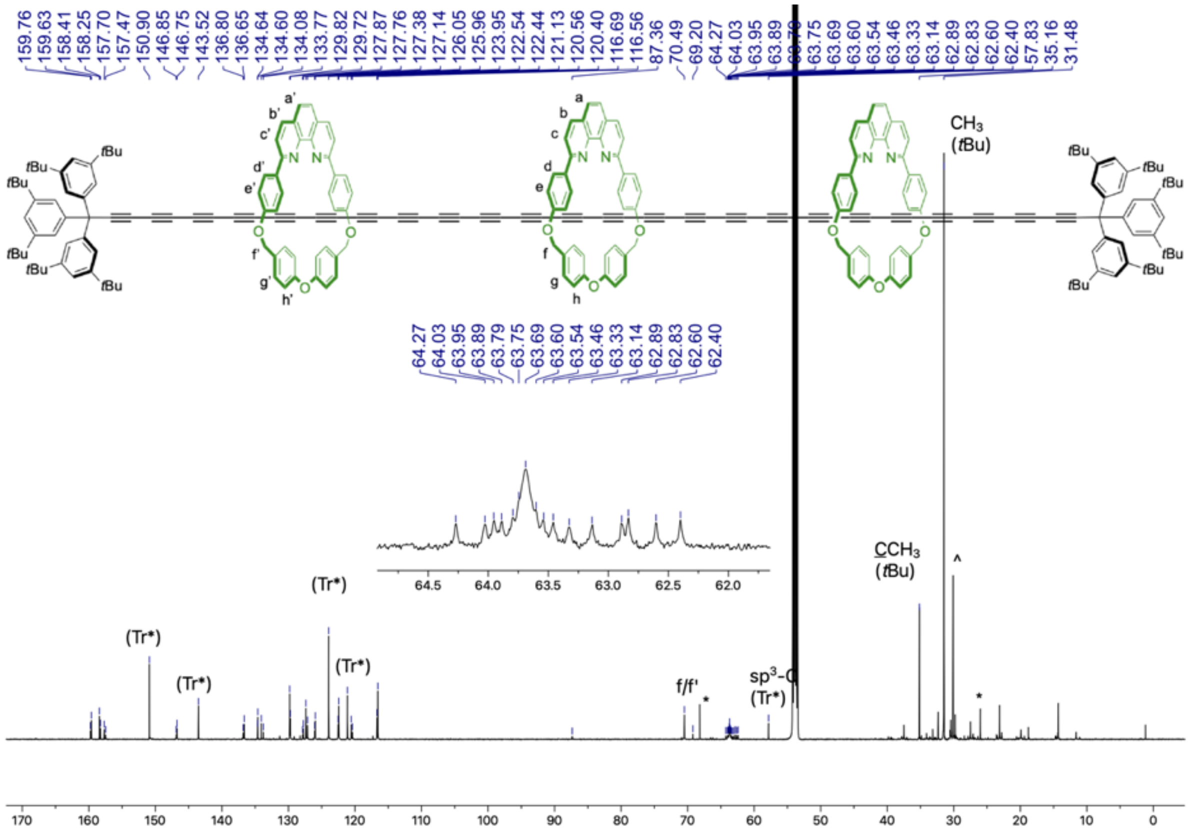

Figure 83. $^{13}C\{^{1}H\}$ NMR (151 MHz) spectrum of compound **C48·(M$_b$)$_3$** in CD_2Cl_2 (^and *indicate silicon grease and residual THF, respectively).

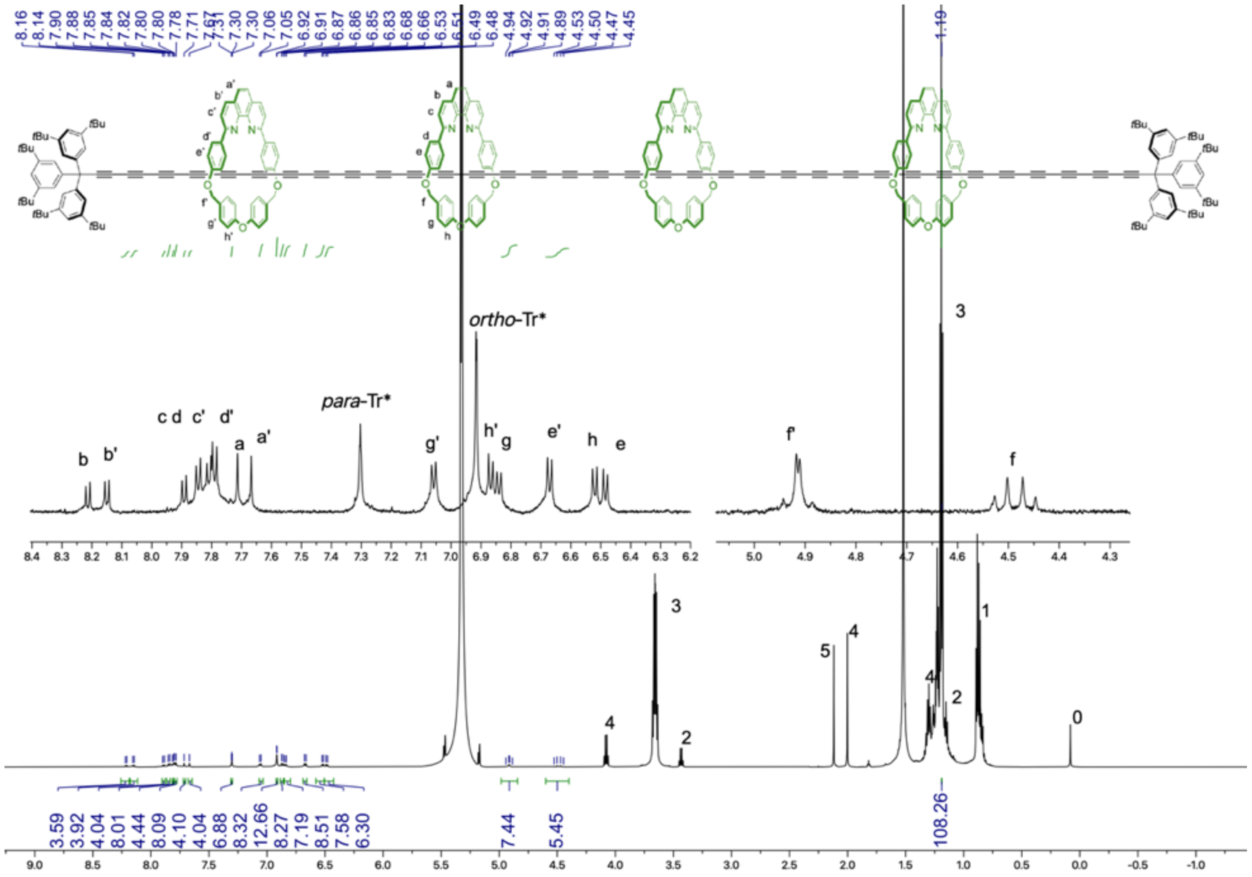

Figure 84. ^{1}H NMR (600 MHz) spectrum of compound **C68·(M$_b$)$_4$** in CD_2Cl_2. 0–5 denote the signals of silicon grease, petrol ether, diethyl ether, ethanol, ethyl acetate, and acetone, respectively.

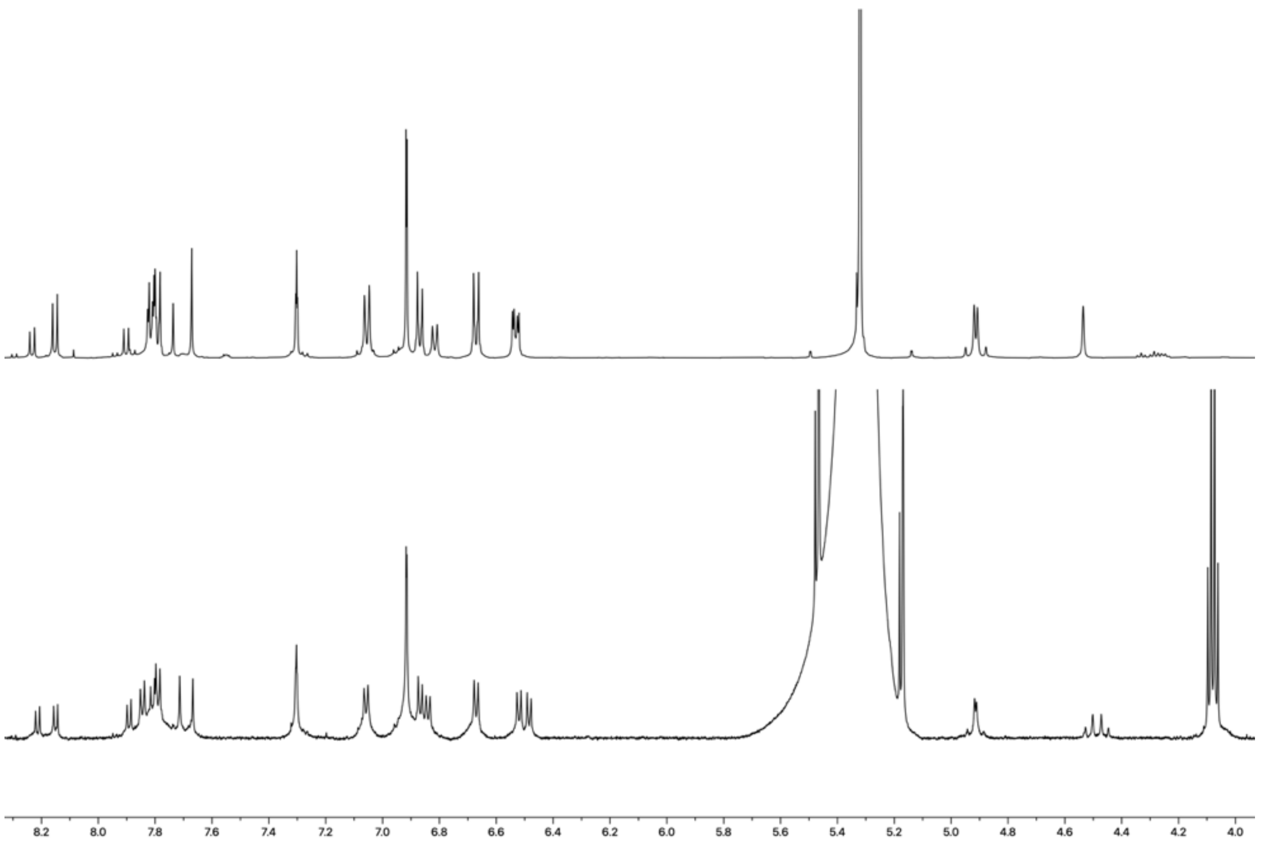

Figure 85. Comparison of ^{1}H NMR (600 MHz) spectrum of compound **C48·(M$_b$)$_3$** (top) and **C68·(M$_b$)$_4$** (bottom) in CD_2Cl_2.

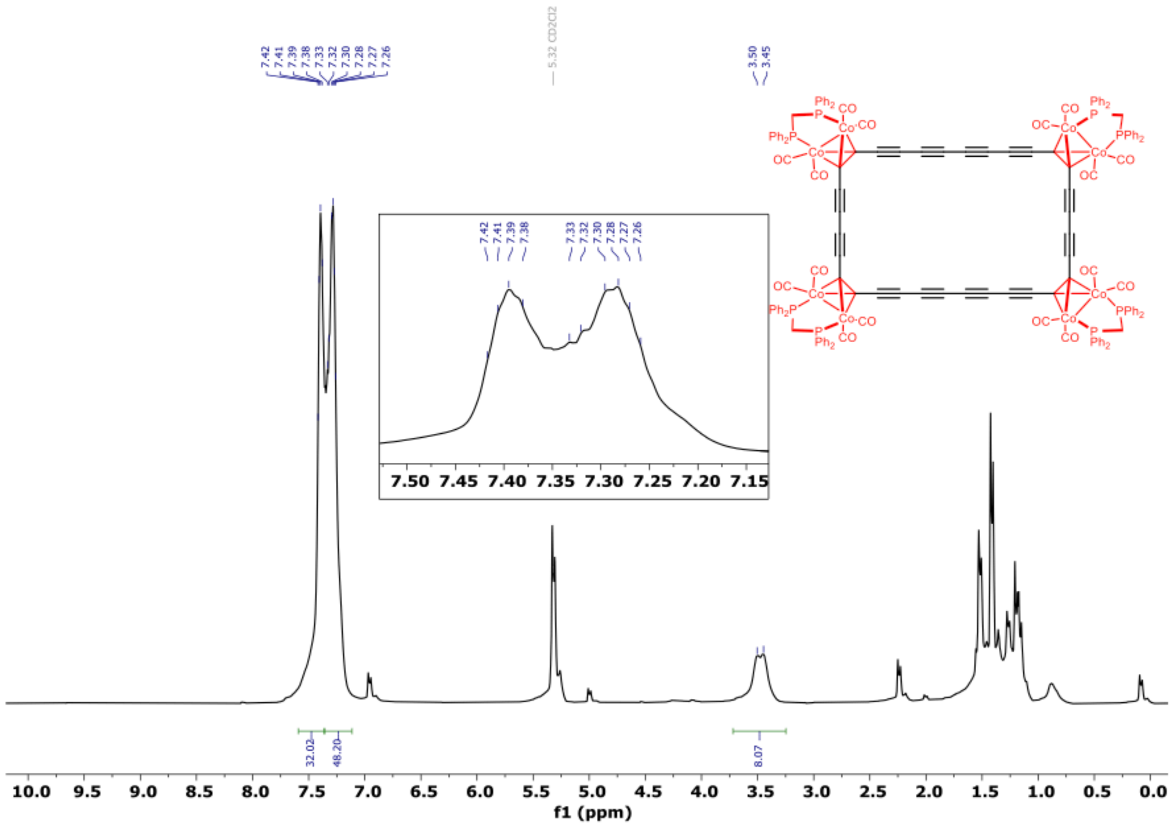

Figure 86. ^{1}H NMR (600 MHz) spectrum of compound ***mc*C32** in CD_2Cl_2.

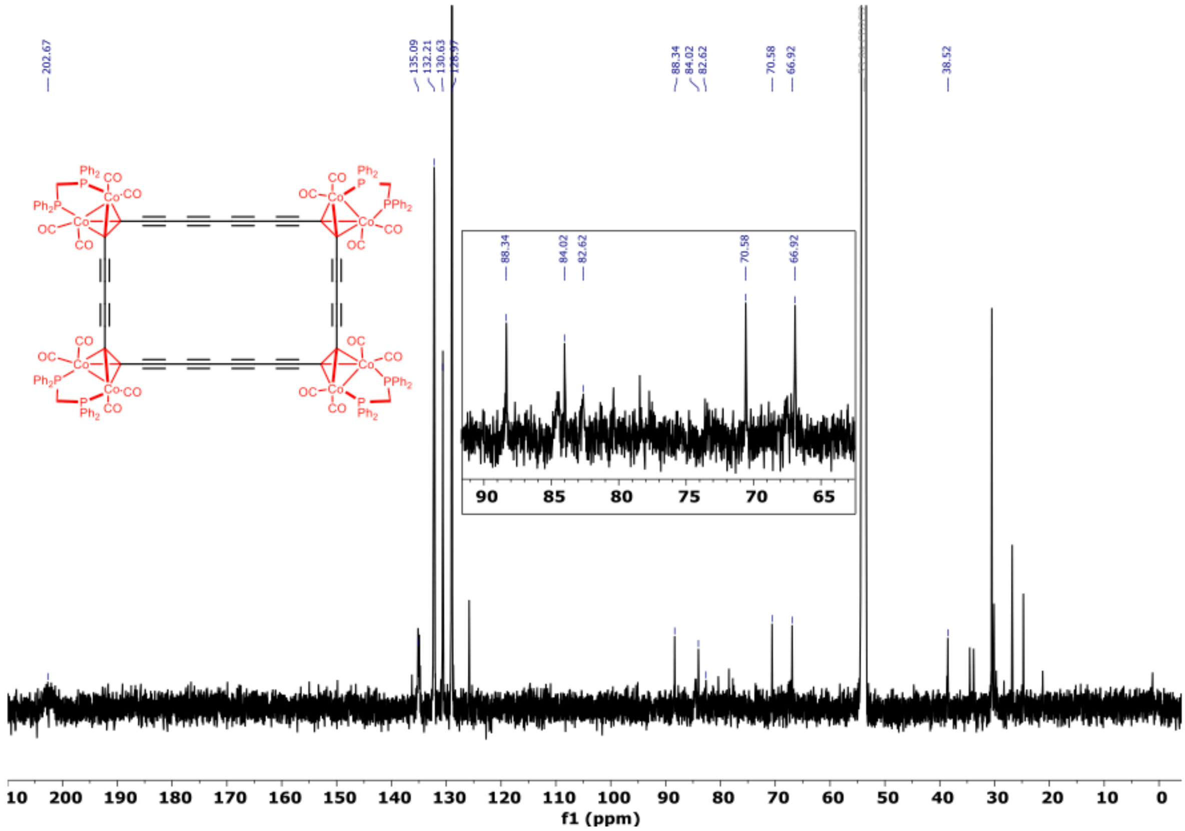

Figure 87. $^{13}C\{^{1}H\}$ NMR (151 MHz) spectrum of compound ***mc*C32** in CD_2Cl_2.

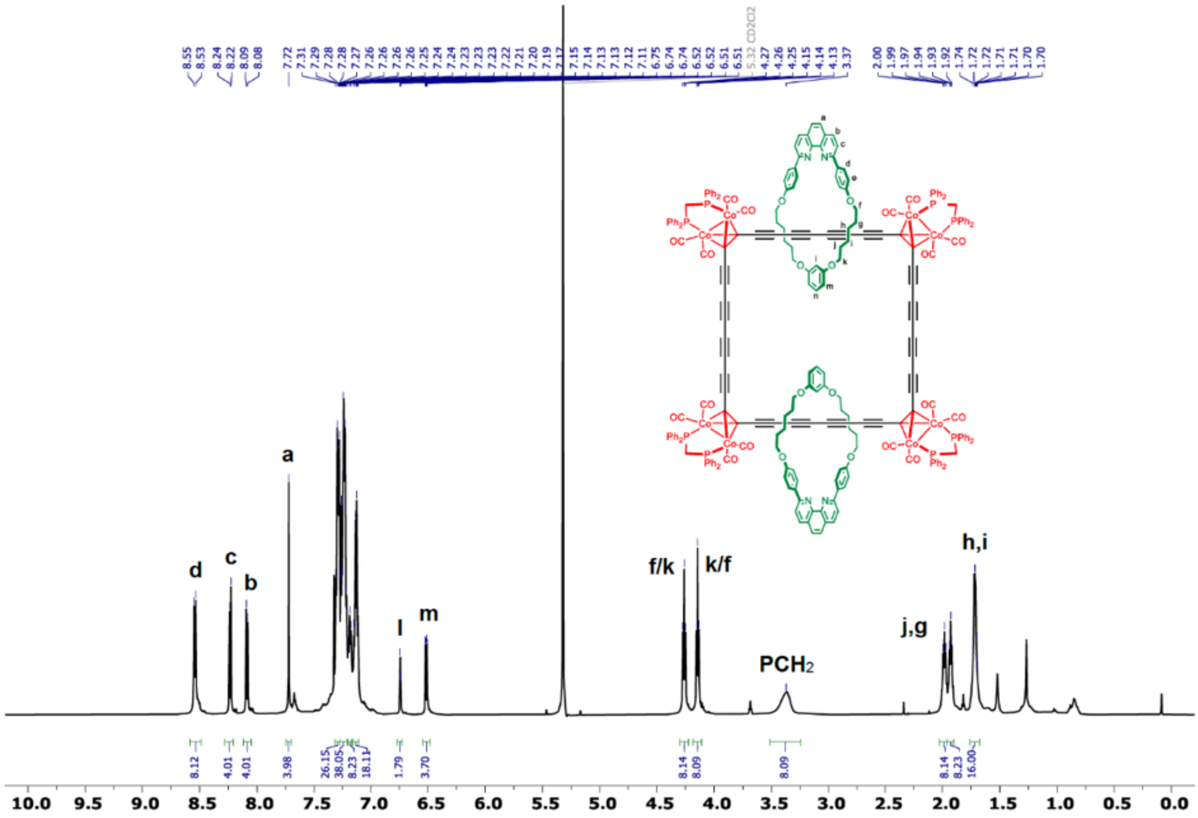

Figure 88. ^{1}H NMR (600 MHz) spectrum of compound ***mc*C40·(Ma)2** in CD_2Cl_2.

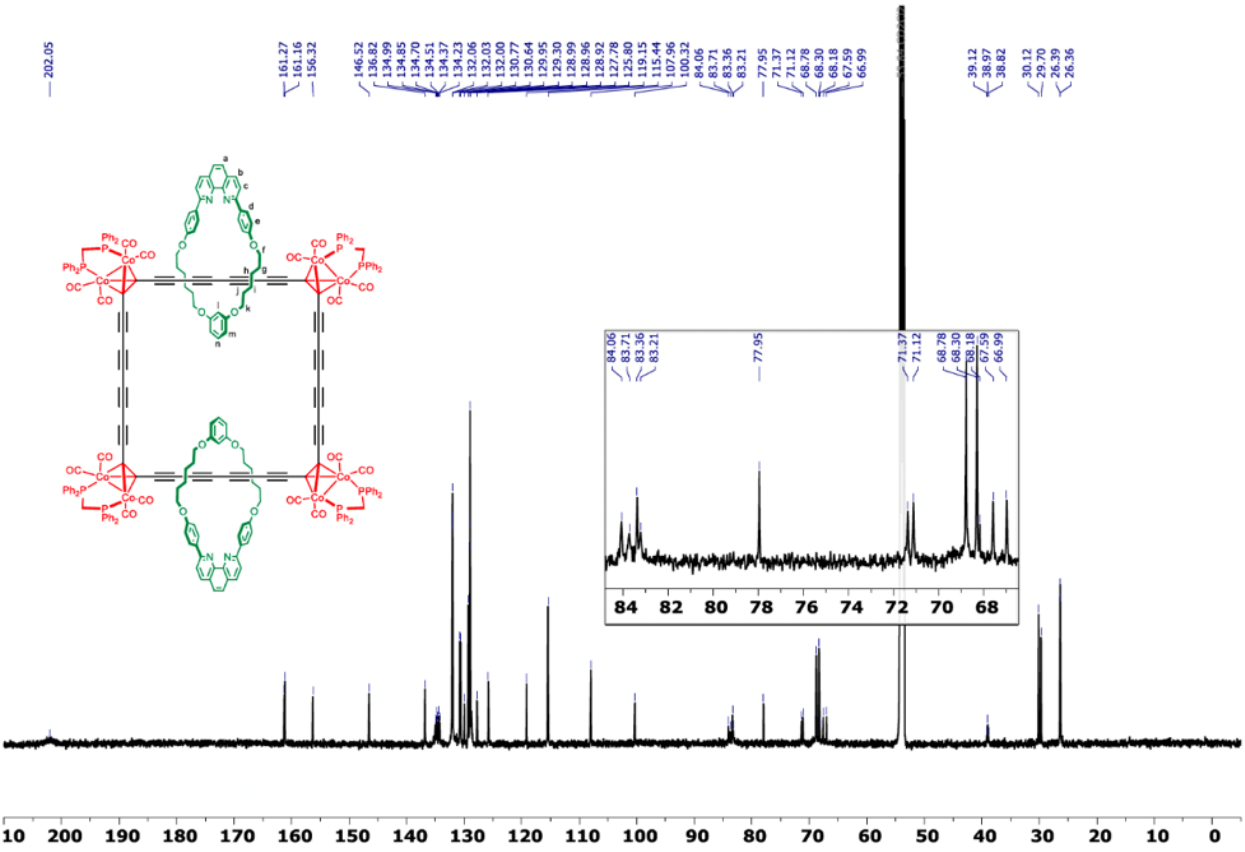

Figure 89. $^{13}C\{^{1}H\}$ NMR (151 MHz) spectrum of compound ***mc*C40·(Ma)2** in CD_2Cl_2.

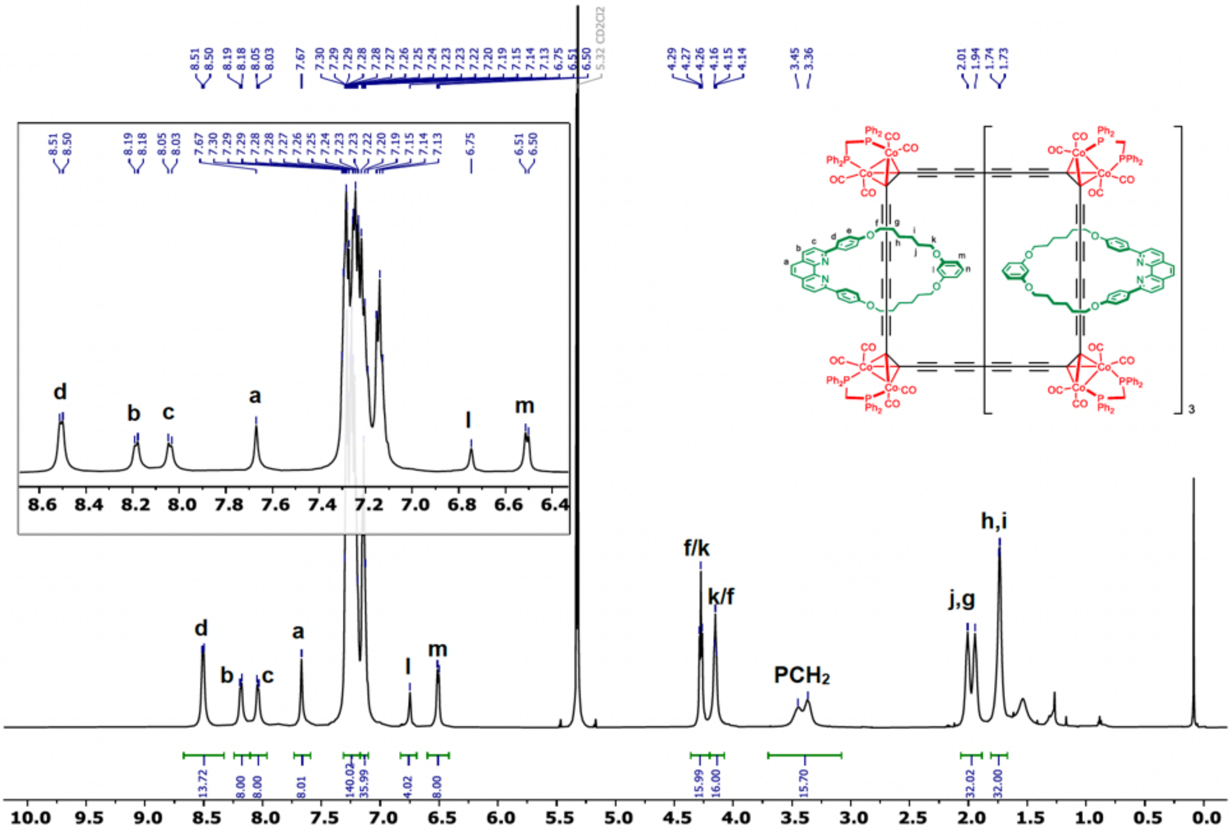

Figure 90. ^{1}H NMR (600 MHz) spectrum of compound ***mc*C80·(M$_a$)$_4$** in CD_2Cl_2.

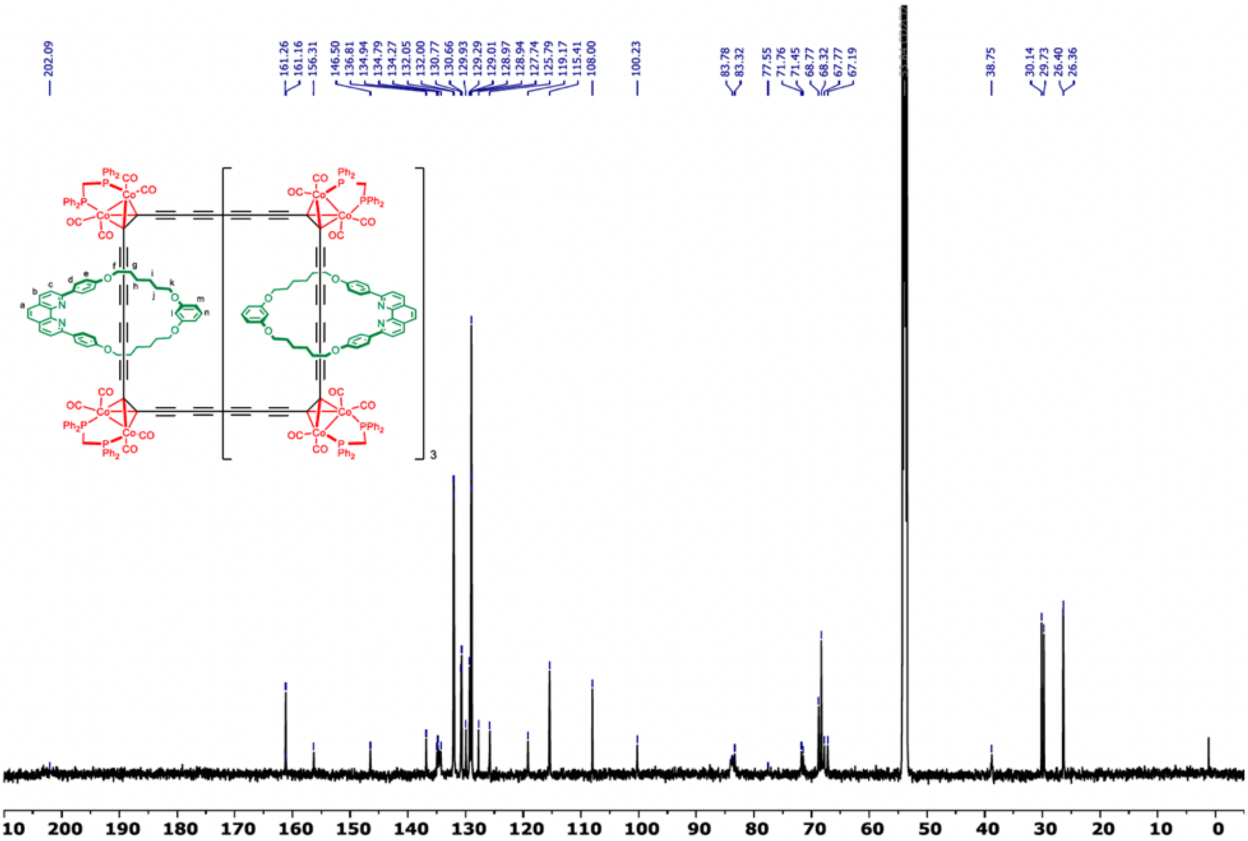

Figure 91. $^{13}C\{^{1}H\}$ NMR (151 MHz) spectrum of compound ***mc*C80·(M$_a$)$_4$** in CD_2Cl_2.

Section 10: Mass Spectra

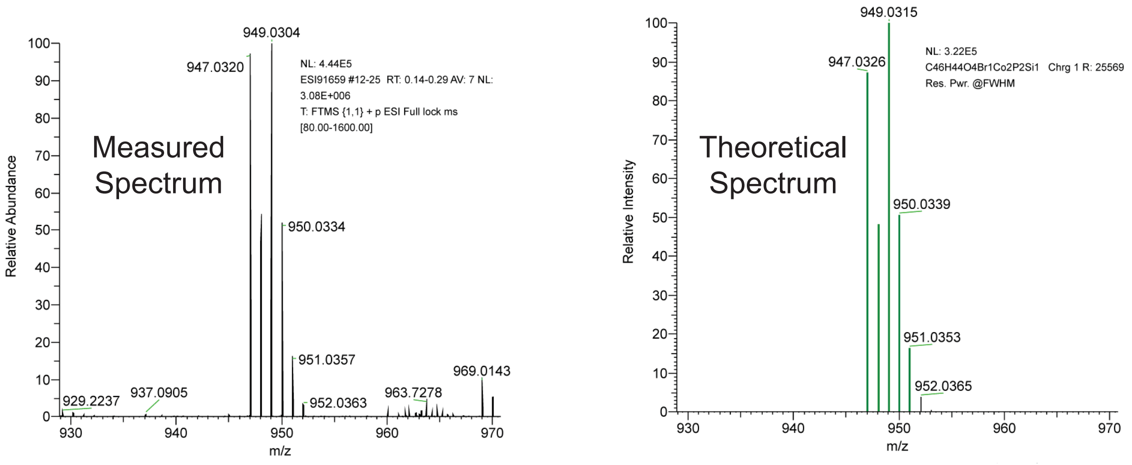

Figure 92. ESI high-resolution mass spectra of compound **2**.

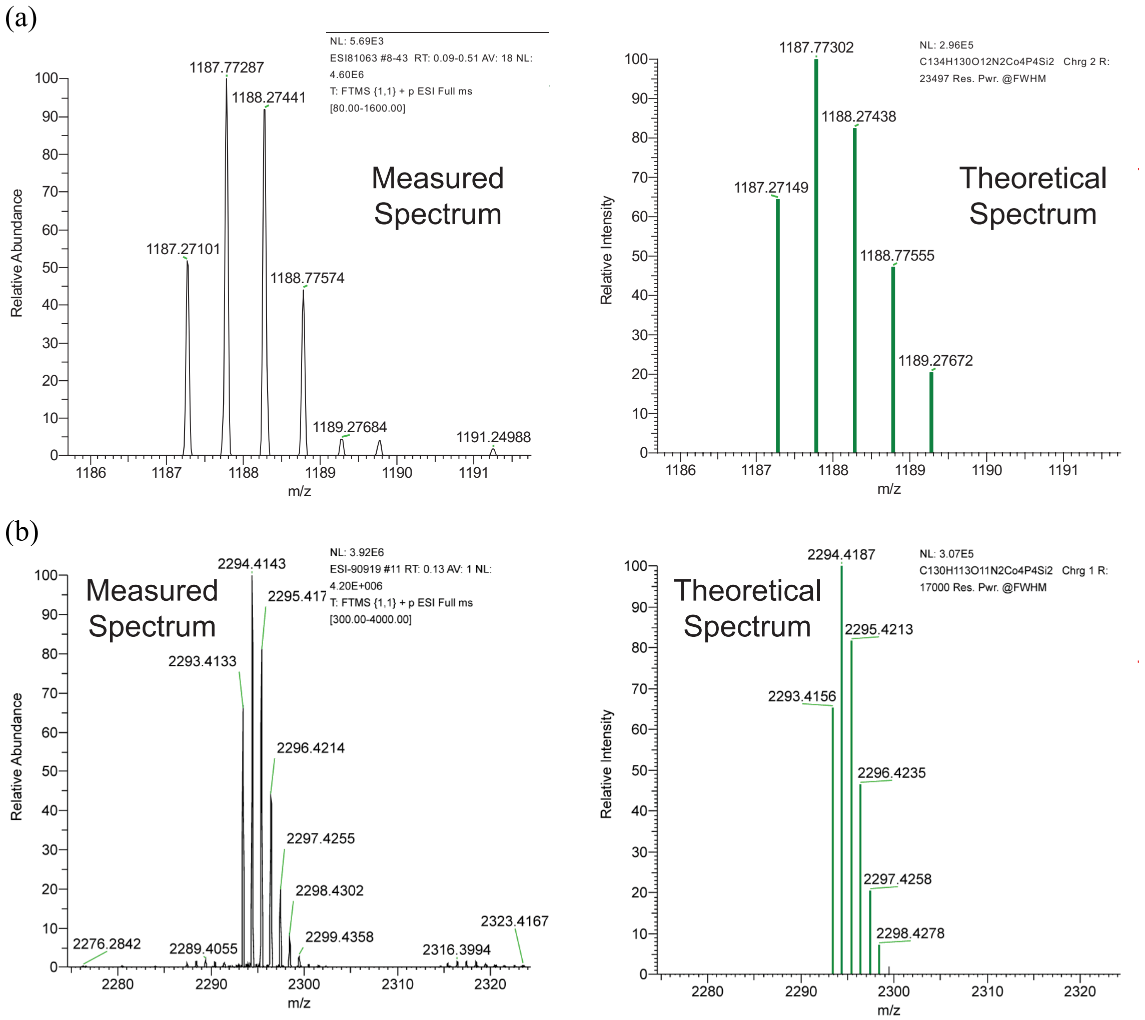

Figure 93. ESI high-resolution mass spectra of (a) **3·M$_a$** and (b) **3·M$_b$**.

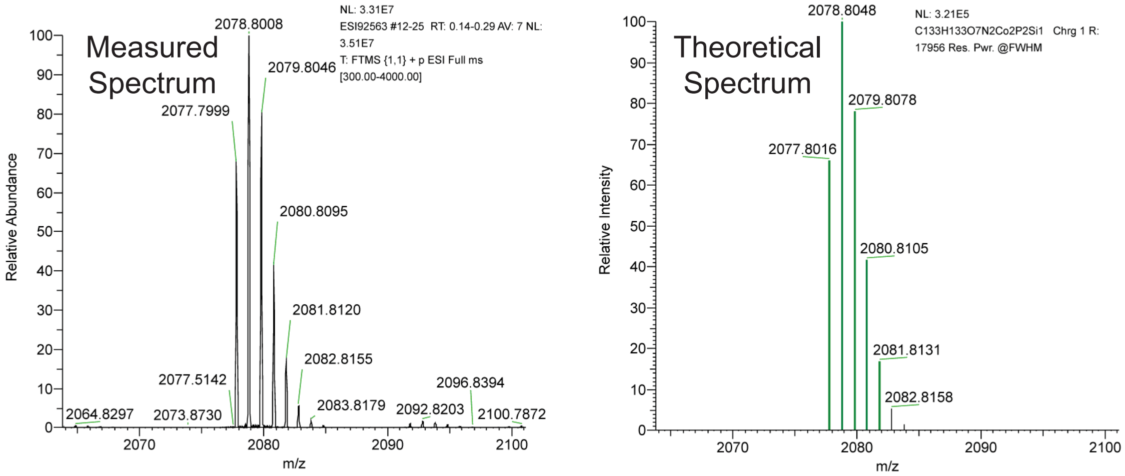

Figure 94. ESI high-resolution mass spectra of **S3·M$_b$**.

(a)

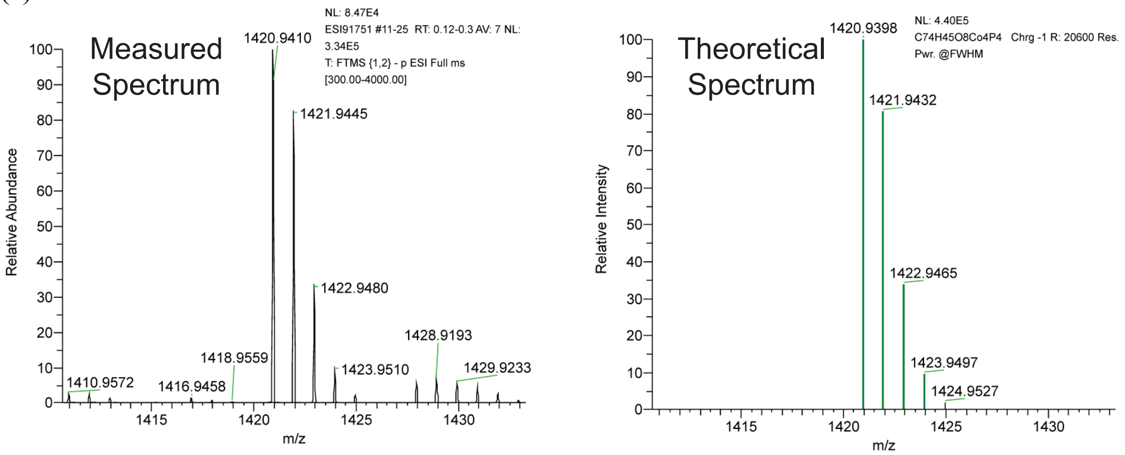

(b)

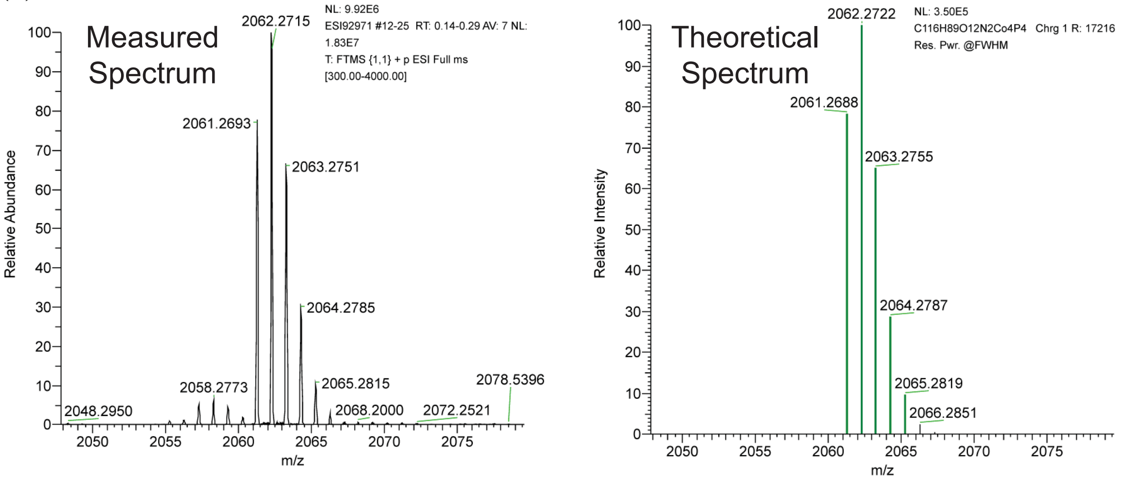

Figure 95. ESI high-resolution mass spectra of (a) compound **4** and (b) **4·M$_a$**.

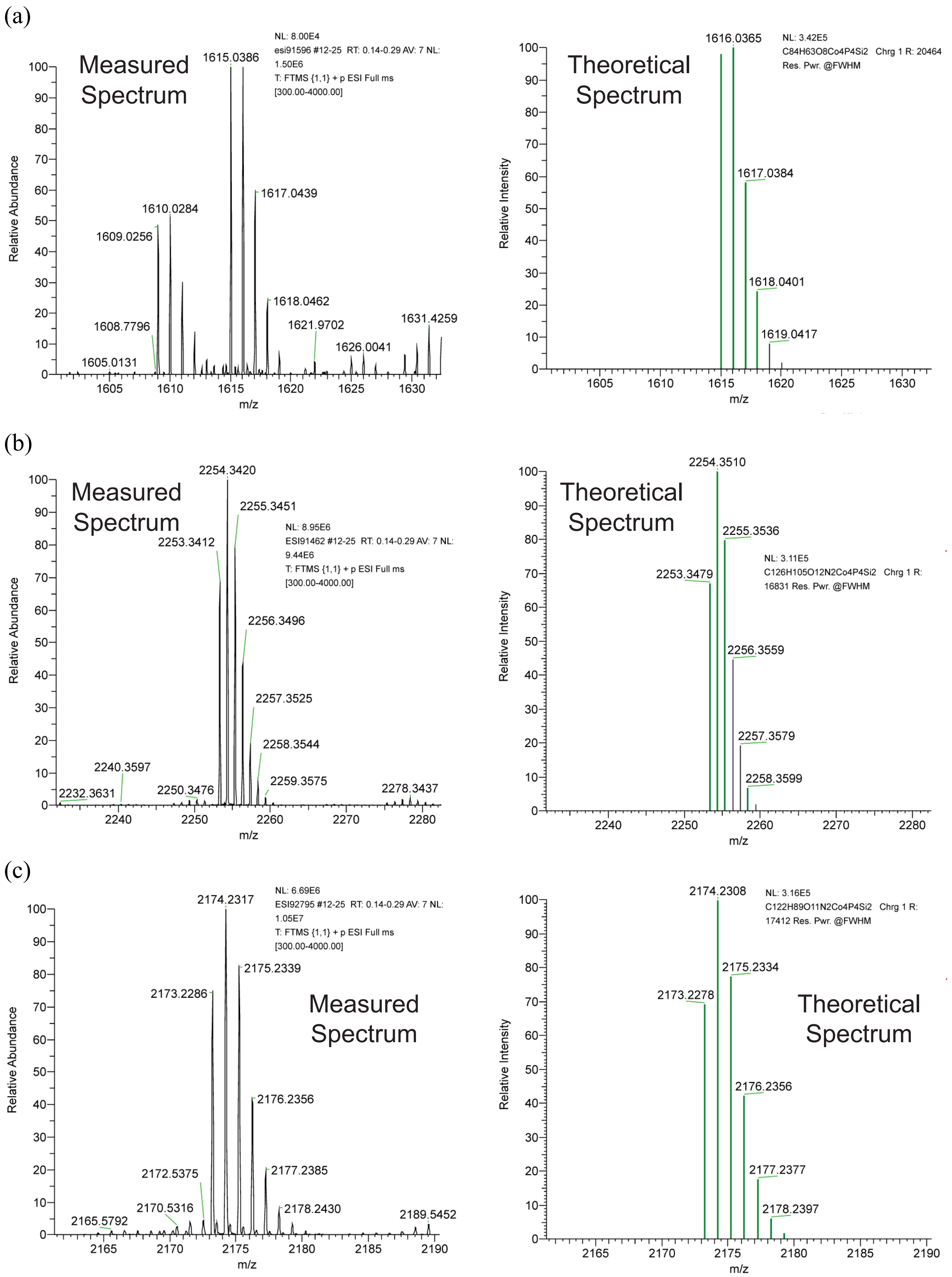

Figure 96. ESI high-resolution mass spectra of (a) **5**, (b) **5·M$_a$**, and (c) **5·M$_b$**.

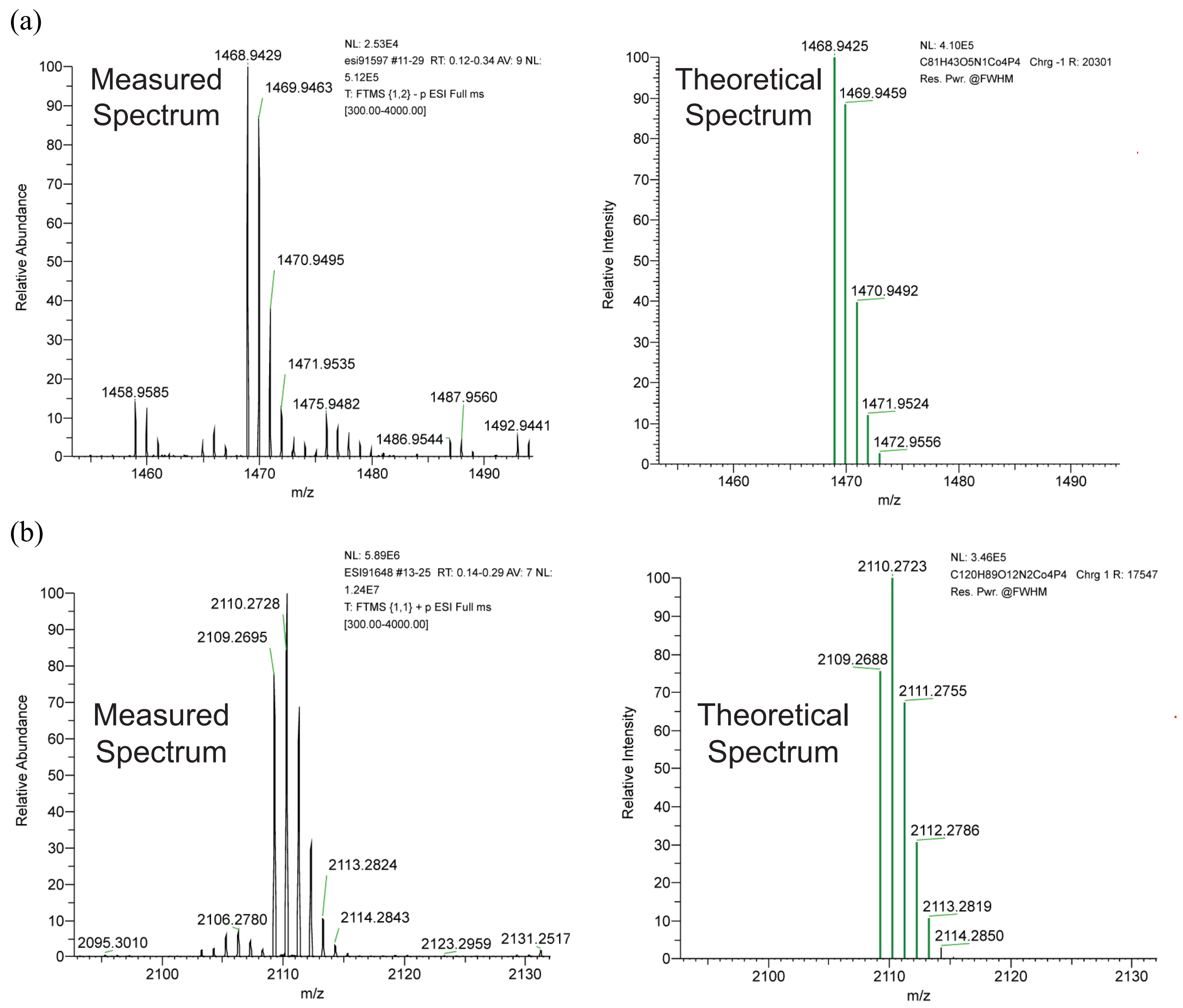

Figure 97. ESI high-resolution mass spectra of (a) **6** and (c) $\mathbf{6{\cdot}M_a}$.

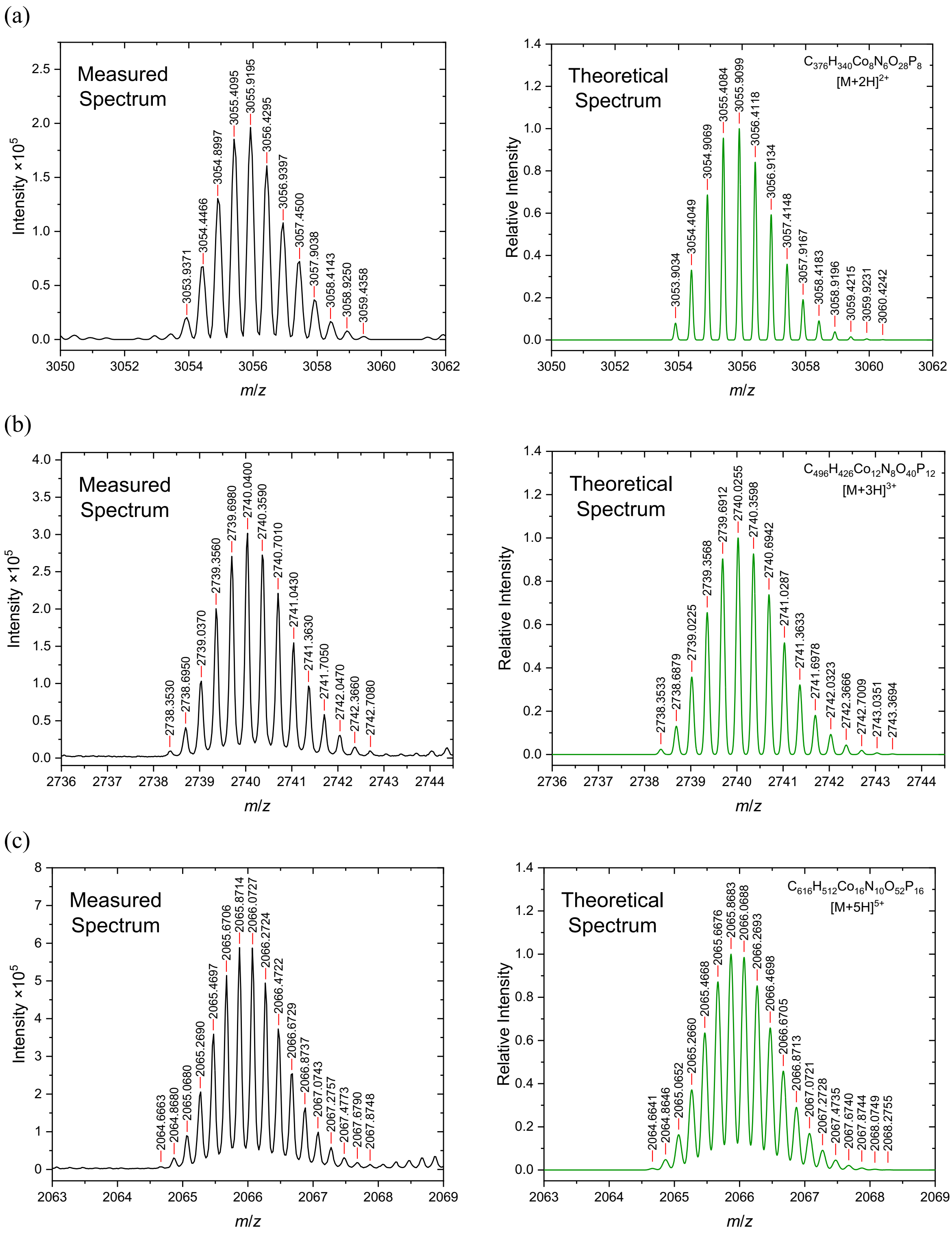

Figure 98. ESI high-resolution mass spectra of (a) ***m*C48·(M$_a$)$_3$**, (b) ***m*C68·(M$_a$)$_4$**, and (c) ***m*C88·(M$_a$)$_5$**.

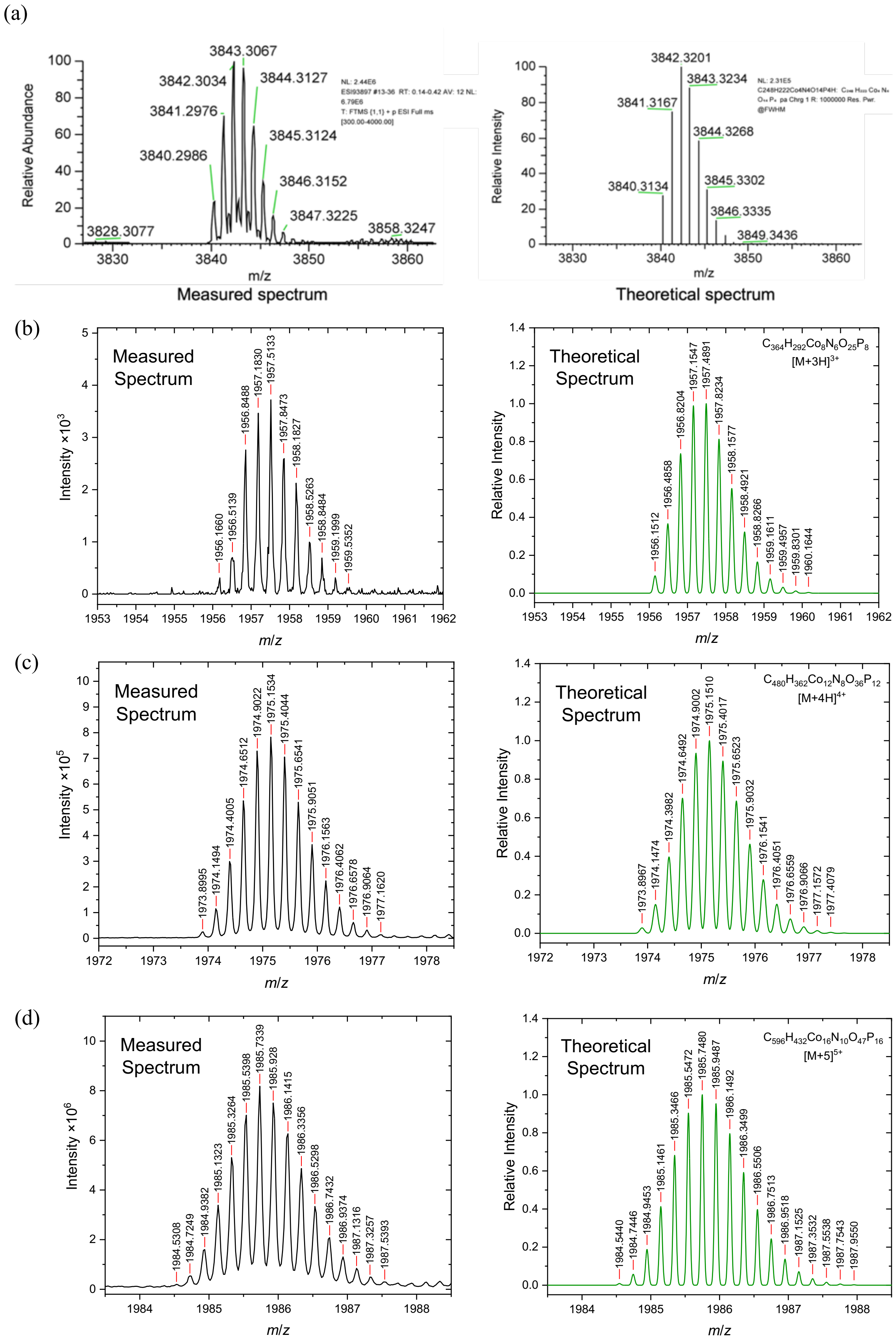

Figure 99. ESI high-resolution mass spectra of (a) ***m*C28·(M$_b$)$_2$**, (b) ***m*C48·(M$_b$)$_3$**, (c) ***m*C68·(M$_b$)$_4$**, and (d) ***m*C88·(M$_b$)$_5$**.

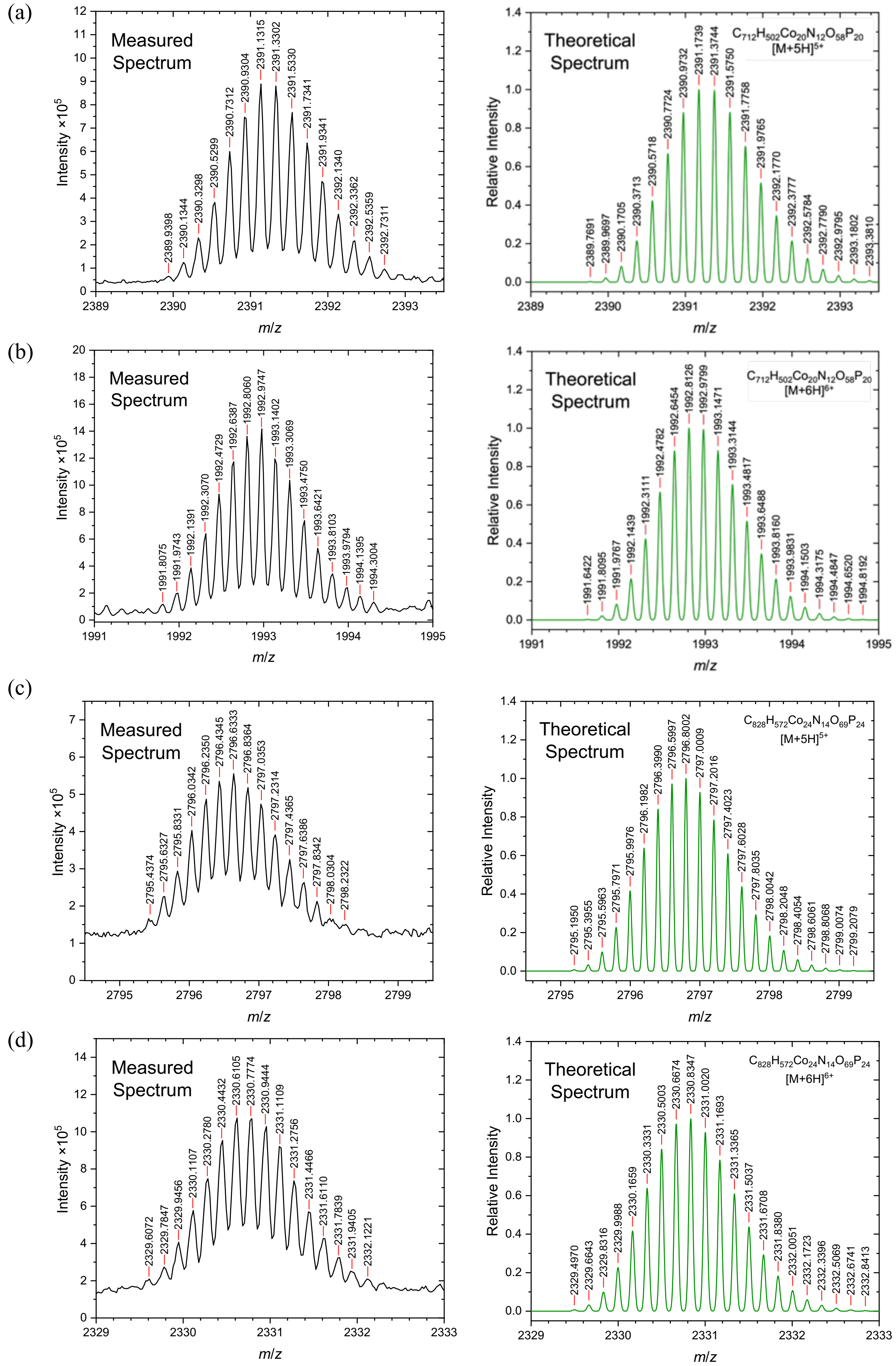

Figure 100. ESI high-resolution mass spectra of [7]rotaxane ***m*C108·(M$_b$)$_6$** in (a) 5$^+$ cation form and (b) 6$^+$ cation form and [8]rotaxane ***m*C128·(M$_b$)$_7$** in (c) 5$^+$ cation form and (d) 6$^+$ cation form.

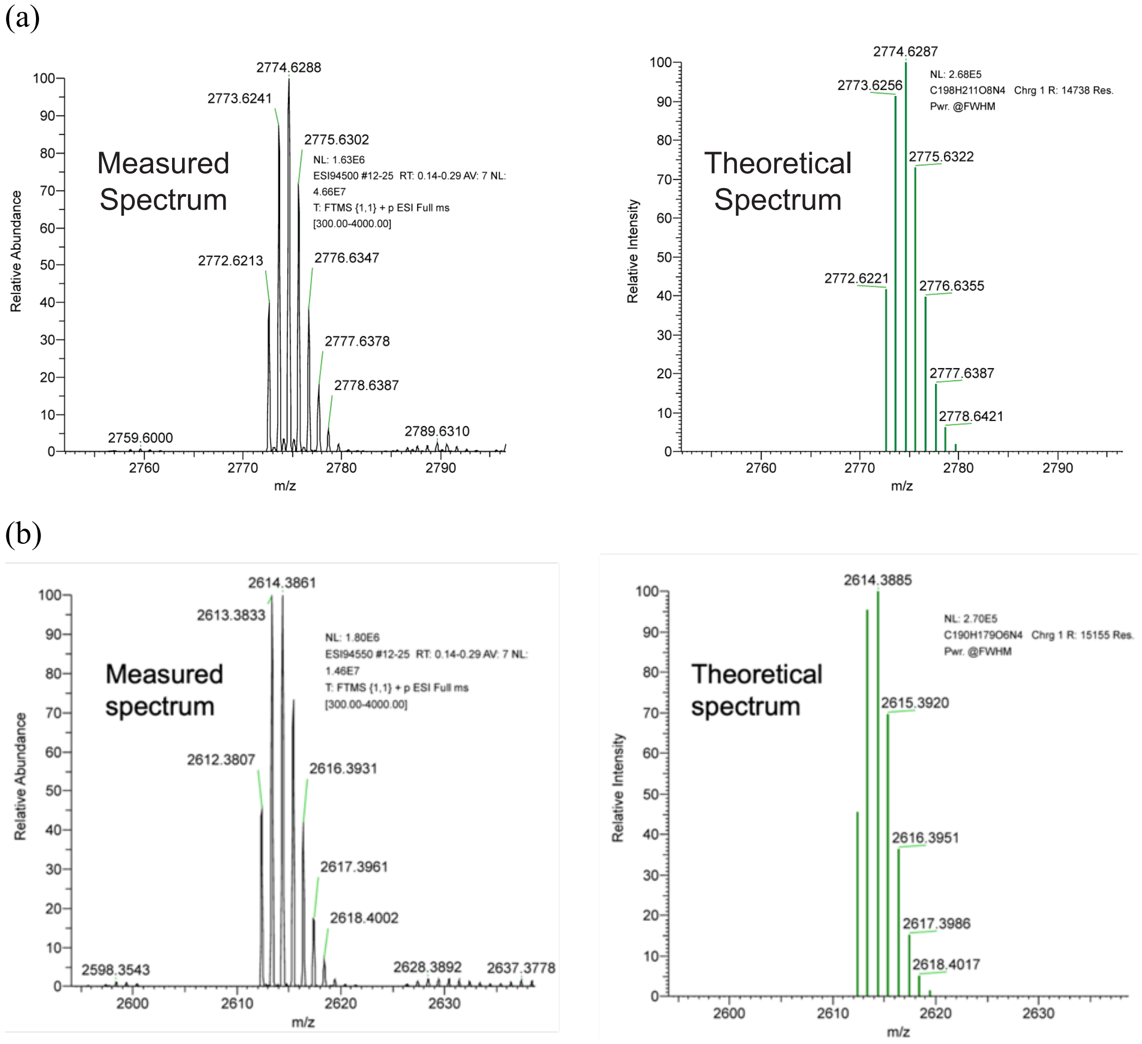

Figure 101. ESI high-resolution mass spectra of (a) **C28·(M$_a$)$_2$** and (b) **C28·(M$_b$)$_2$**.

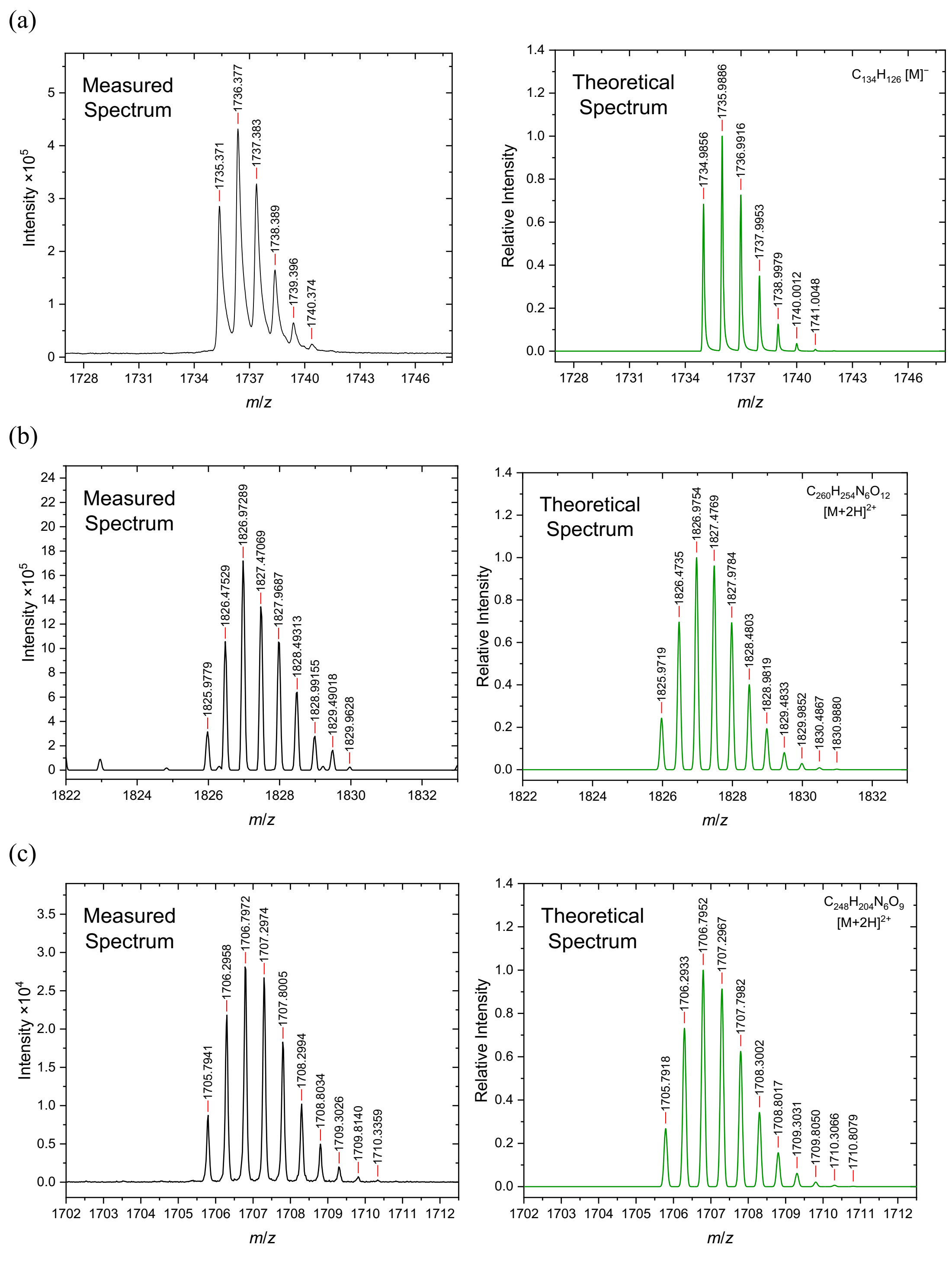

Figure 102. MALDI-ToF mass spectrum high-resolution mass spectra of (a) polyyne dumbbell **C48**, (b) polyyne rotaxanes **C48·(M$_a$)$_3$**, and (c) **C48·(M$_b$)$_3$**.

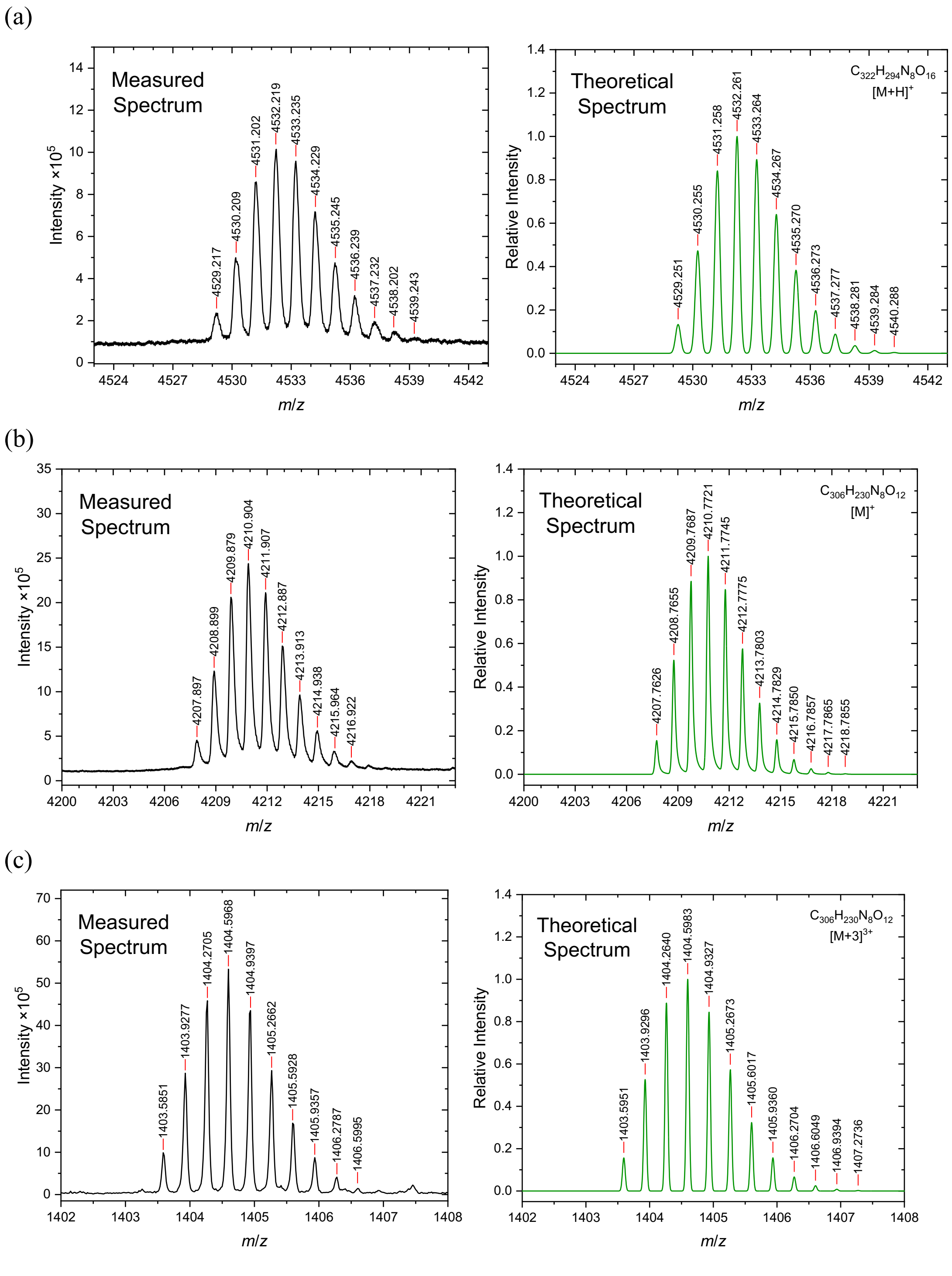

Figure 103. MALDI-ToF mass spectrum high-resolution mass spectra of (a) polyyne rotaxanes **C68·(M$_a$)$_4$** and (b) **C68·(M$_b$)$_4$** as well as (c) ESI high-resolution mass spectra of **C68·(M$_b$)$_4$**.

(a)

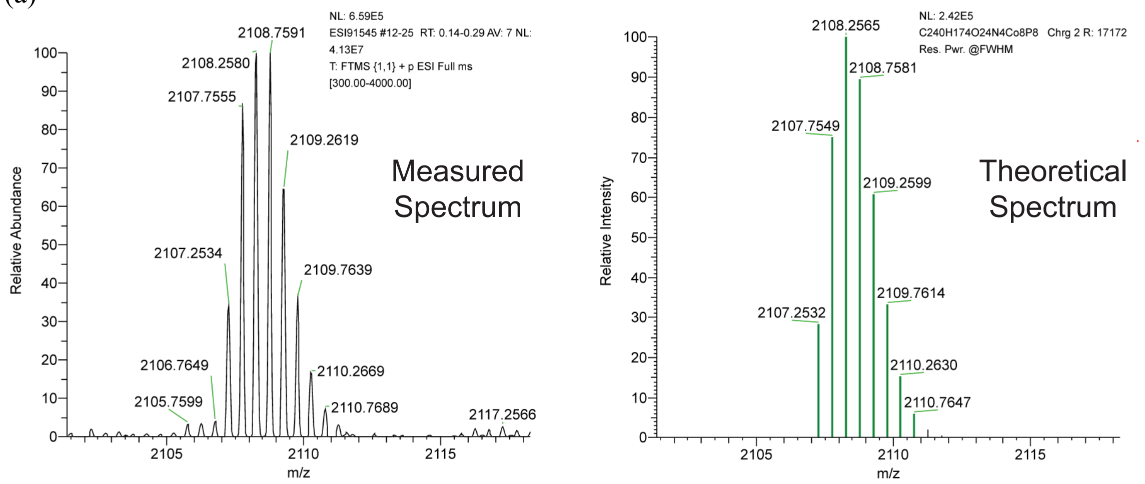

(b)

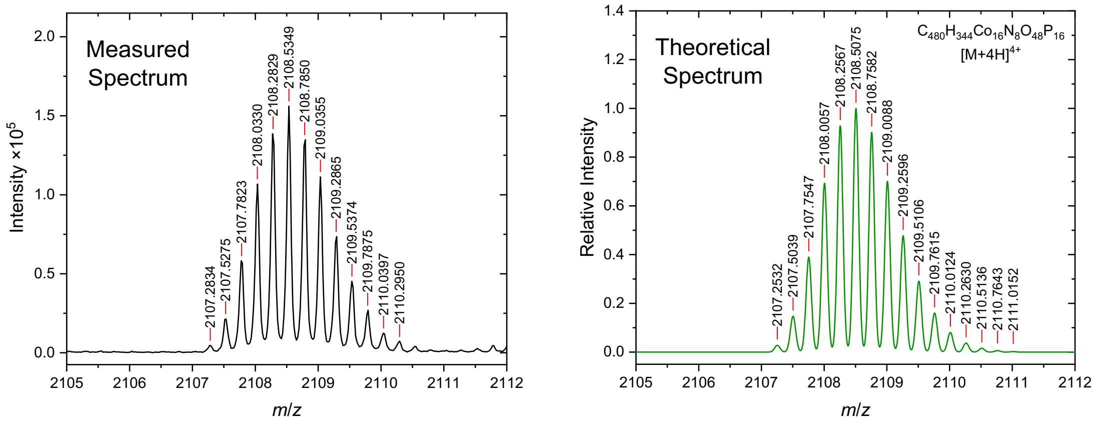

Figure 104. ESI high-resolution mass spectra of (a) ***mc*C40·(M$_a$)$_2$** and (b) ***mc*C80·(M$_a$)$_4$**.

Section 11: References

[1] Keller, R., Wrcoff, H. & Marchi, L. E. Copper(I) chloride. *Inorg. Synth.* **2**, 1–4 (1946).

[2] Chalifoux, W. A. & Tykwinski, R. R. Synthesis of polyynes to model the *sp*-carbon allotrope carbyne. *Nat. Chem.* **2**, 967–971 (2010).

[3] Saito, S., Nakazono, K. & Takahashi, E. Template synthesis of [2]rotaxanes with large ring components and tris(biphenyl)methyl group as the blocking group. The relationship between the ring size and the stability of the rotaxanes. *J. Org. Chem.* **71**, 7477–7480 (2006).

[4] Franz, M., Januszewski, J. A., Wendinger, D., Neiss, C., Movsisyan, L. D., Hampel, F., Anderson, H. L., Gorling, A. & Tykwinski, R. R. Cumulene rotaxanes: stabilization and study of [9]cumulenes. *Angew. Chem. Int. Ed.* **54**, 6645–6649 (2015).

[5] Kohn, D. R., Gawel, P., Xiong, Y.; Christensen, K. E. & Anderson, H. L. Synthesis of polyynes using dicobalt masking groups. *J. Org. Chem.* **83**, 2077–2086 (2018).

[6] Patrick, C. W., Woods, J. F., Gawel, P., Otteson, C. E., Thompson, A. L., Claridge, T. D. W., Jasti, R. & Anderson, H. L. Polyyne [3]rotaxanes: Synthesis via dicobalt carbonyl complexes and enhanced stability. *Angew. Chem. Int. Ed.* **61**, e202116897 (2022).

[7] Movsisyan, L. D., Franz, M., Hampel, F., Thompson, A. L., Tykwinski, R. R. & Anderson, H. L. Polyyne rotaxanes: Stabilization by encapsulation. *J. Am. Chem. Soc.* **138**, 1366–1376 (2016).

[8] Gawel, P., Woltering, S. L., Xiong, Y., Christensen, K. E. & Anderson, H. L. Masked alkyne equivalents for the synthesis of mechanically interlocked polyynes. *Angew. Chem. Int. Ed.* **60**, 5941–5947 (2021).

[9] Allan, D. R., et al. A novel dual air-bearing fixed-χ diffractometer for small-molecule single-crystal X-ray diffraction on beamline I19 at Diamond Light Source. *Crystals* **7**, 336 (2017).

[10] Winter, G. xia2: an expert system for macromolecular crystallography data reduction. *J. Appl. Cryst.* **43**, 186–190 (2010).

[11] Winter, G. et al. DIALS: implementation and evaluation of a new integration package. *Acta Cryst.* **D74**, 85–97 (2018).

[12] Palatinus, L. & Chapuis, G., SUPERFLIP – a computer program for the solution of crystal structures by charge flipping in arbitrary dimensions. *J. Appl. Cryst.* **40**, 786–790 (2007).

[13] Betteridge, P. W., Carruthers, J. R., Cooper, R. I., Prout, K. & Watkin, D. J. CRYSTALS version 12: software for guided crystal structure analysis. *J. Appl. Cryst.* **36**, 1487 (2003).

[14] Cooper, R. I., Thompson, A. L. & Watkin, D. J. CRYSTALS enhancements: dealing with hydrogen atoms in refinement. *J. Appl. Cryst.* **43**, 1100–1107 (2010).
